# The methyltransferase METTL3 negatively regulates nonalcoholic steatohepatitis (NASH) progression

**DOI:** 10.1038/s41467-021-27539-3

**Published:** 2021-12-10

**Authors:** Xinzhi Li, Bingchuan Yuan, Min Lu, Yuqin Wang, Na Ding, Chunhong Liu, Ming Gao, Zhicheng Yao, Shiyan Zhang, Yujun Zhao, Liwei Xie, Zheng Chen

**Affiliations:** 1 HIT Center for Life Sciences, School of Life Science and Technology, Harbin Institute of Technology, Harbin, 150001 China; 2 Department of General surgery, The third affiliated hospital of Sun Yat-sen university, Guangzhou, 510530 China; 3 State Key Laboratory of Drug Research and Small-Molecule Drug Research Center, Shanghai Institute of Materia Medica, Chinese Academy of Sciences, Shanghai, 201203 China; 4 University of Chinese Academy of Sciences, Beijing, 100049 China; 5 State Key Laboratory of Applied Microbiology Southern China, Guangdong Provincial Key Laboratory of Microbial Culture Collection and Application, Guangdong Open Laboratory of Applied Microbiology, Institute of Microbiology, Guangdong Academy of Sciences, Guangzhou, 510070 China

**Keywords:** Non-alcoholic steatohepatitis, Non-alcoholic steatohepatitis, Epigenetics

## Abstract

Nonalcoholic steatohepatitis (NASH) is a key step in the progression of nonalcoholic fatty liver (NAFL) to cirrhosis. However, the molecular mechanisms of the NAFL-to-NASH transition are largely unknown. Here, we identify methyltransferase like 3 (METTL3) as a key negative regulator of NASH pathogenesis. Hepatocyte-specific deletion of *Mettl3* drives NAFL-to-NASH progression by increasing CD36-mediated hepatic free fatty acid uptake and CCL2-induced inflammation, which is due to increased chromatin accessibility in the promoter region of *Cd36* and *Ccl2*. Antibody blockade of CD36 and CCL2 ameliorates NASH progression in hepatic *Mettl3* knockout mice. Hepatic overexpression of *Mettl3* protects against NASH progression by inhibiting the expression of CD36 and CCL2. Mechanistically, METTL3 directly binds to the promoters of the *Cd36* and *Ccl2* genes and recruits HDAC1/2 to induce deacetylation of H3K9 and H3K27 in  their promoters, thus suppressing *Cd36* and *Ccl2* transcription. Furthermore, METTL3 is translocated from the nucleus to the cytosol in NASH, which is associated with CDK9-mediated phosphorylation of METTL3. Our data reveal a mechanism by which METTL3 negatively regulates hepatic *Cd36* and *Ccl2* gene transcription *via* a histone modification pathway for protection against NASH progression.

## Abstract (web summary)

Mechanisms that underlie the progression of non-alcoholic fatty liver disease to the more severe non-alcoholic steatohepatitis are incompletely understood. Here the authors show that METTL3, a RNA methyltransferase that catalyzes mRNA m6 modifications, negatively regulates NASH progression via inhibiting the transcription of *Cd36* and *Ccl2*.

## Introduction

Nonalcoholic fatty liver disease (NAFLD), which ranges from nonalcoholic fatty liver (NAFL) to nonalcoholic steatohepatitis (NASH), is one of the most common chronic liver diseases in both developed and developing countries owing to an increased rate of obesity^1,2^. Approximately 25% of patients with NAFL develop NASH^3^, which is characterized by hepatic steatosis, liver injury, chronic inflammation, and liver fibrosis and is a key step in the development of cirrhosis and hepatocellular carcinoma (HCC)^3,4^. Several mediators have been shown to regulate NASH initiation and progression, such as lipotoxicity, oxidative stress, mitochondrial dysfunction, cell death, and immune cell activation^5,6^. However, the molecular events that determine whether patients with NAFL develop NASH remain unknown.

A “two-hit” theory has been proposed to explain NASH pathogenesis^7^. The first hit, hepatic steatosis, consists of lipid accumulation in the liver due to increased free fatty acid uptake or de novo lipogenesis^8,9^, which sensitizes the liver to the second hit of oxidative stress, inflammation, and injury^7,10,11^. Approximately 59% of hepatic triglycerides in humans are derived from serum non-esterified fatty acids^9^, which indicates the importance of free fatty-acid uptake in the pathogenesis of NAFL and NASH. Free fatty acid uptake is increased in NAFL and NASH due to increased expression of CD36^12^. Knockout of *Cd36* has been shown to protect against diet-induced steatosis and NASH^13^. The second hit may be inflammation, which drives the progression of NAFL-to-NASH^10^. Chemokines such as CCL2 and its receptor CCR2 are abnormally upregulated during NASH progression^14,15^, and inhibition of CCL2 and CCR2 has been shown to be a therapeutic approach for the treatment of NASH^16,17^. It is possible that the molecular drivers that coordinate steatosis and inflammation mediate the NAFL-to-NASH transition. However, these molecular drivers have not yet been identified.

Methyltransferase like 3 (METTL3) is a key RNA methyltransferase that catalyzes mRNA m^6^A modifications^18^. METTL14 and WTAP both regulate METTL3^18–20^. METTL3-mediated m^6^A modification has been shown to participate in many biological processes, such as neurogenesis^21,22^, spermatogenesis^23^, circadian rhythms^24^, stem cell pluripotency^25,26^, postnatal development of interscapular brown adipose tissue in mice^27^, and islet β-cell function^28^, by regulating mRNA stability, mRNA splicing, and translational efficiency. In addition, METTL14 regulates neurogenesis through the modulation of histone modifications^29^. Recently, METTL3 has also been shown to regulate cancer progression by affecting the expression of multiple genes^30–34^. However, whether METTL3 coordinates steatosis and inflammation to mediate the NAFL-to-NASH transition is largely unknown.

Here, we have demonstrated that METTL3 is a key repressor of the NAFL-to-NASH transition. Hepatocyte-specific deletion of *Mettl3* drives the progression of NAFL-to NASH in HFD-fed mice by promoting CD36-mediated hepatic free fatty acid uptake and CCL2-induced inflammation. *Mettl3-*HKO mice also promote MCD-induced NASH, whereas hepatocyte-specific overexpression of *Mettl3* protects against MCD-induced NASH. Mechanistically, METTL3 directly binds to the promoters of the *Cd36* and *Ccl2* genes and recruits HDAC1/2, which causes deacetylation of H3K9 and H3K27 in their promoters; this, in turn, suppresses the transcription of *Cd36* and *Ccl2*. Furthermore, nuclear METTL3 is decreased in NASH, which is likely owing to CDK9-mediated phosphorylation of METTL3. These data reveal a mechanism by which METTL3 represses the transcription of *Cd36* and *Ccl2* via histone modification. This study also suggests that METTL3 is a negative regulator of NASH pathogenesis and may serve as a drug target for the treatment of NASH.

## Results

### Nuclear METTL3 is decreased in NASH livers

db/db (leptin receptor deficiency) mice exhibit severe NAFL but not NASH, whereas a NASH or MCD diet is able to induce NASH in mice. To identify potential regulators that are responsible for the NAFL-to-NASH transition, we assessed two previously published RNA-seq data sets deposited in the Gene Expression Omnibus (GEO) (GEO DataSets: GSE43314 and GSE119340) from WT VS db/db mouse livers and NC VS NASH mouse livers^35–37^. Some genes showed opposite patterns (db/db VS WT fold change >3, and NASH VS NC fold change < 1) in these two data sets, and thus, they may play an important role in the NAFL-to-NASH transition. Among these genes, we noted that *Mettl3*, an RNA methyltransferase-encoding gene, was dramatically upregulated (fold change = 3.945455) in db/db mouse livers but was not upregulated (fold change = 0.88228) in NASH mouse livers, which indicates that METTL3 may regulate NASH progression.

**Fig. 1:**
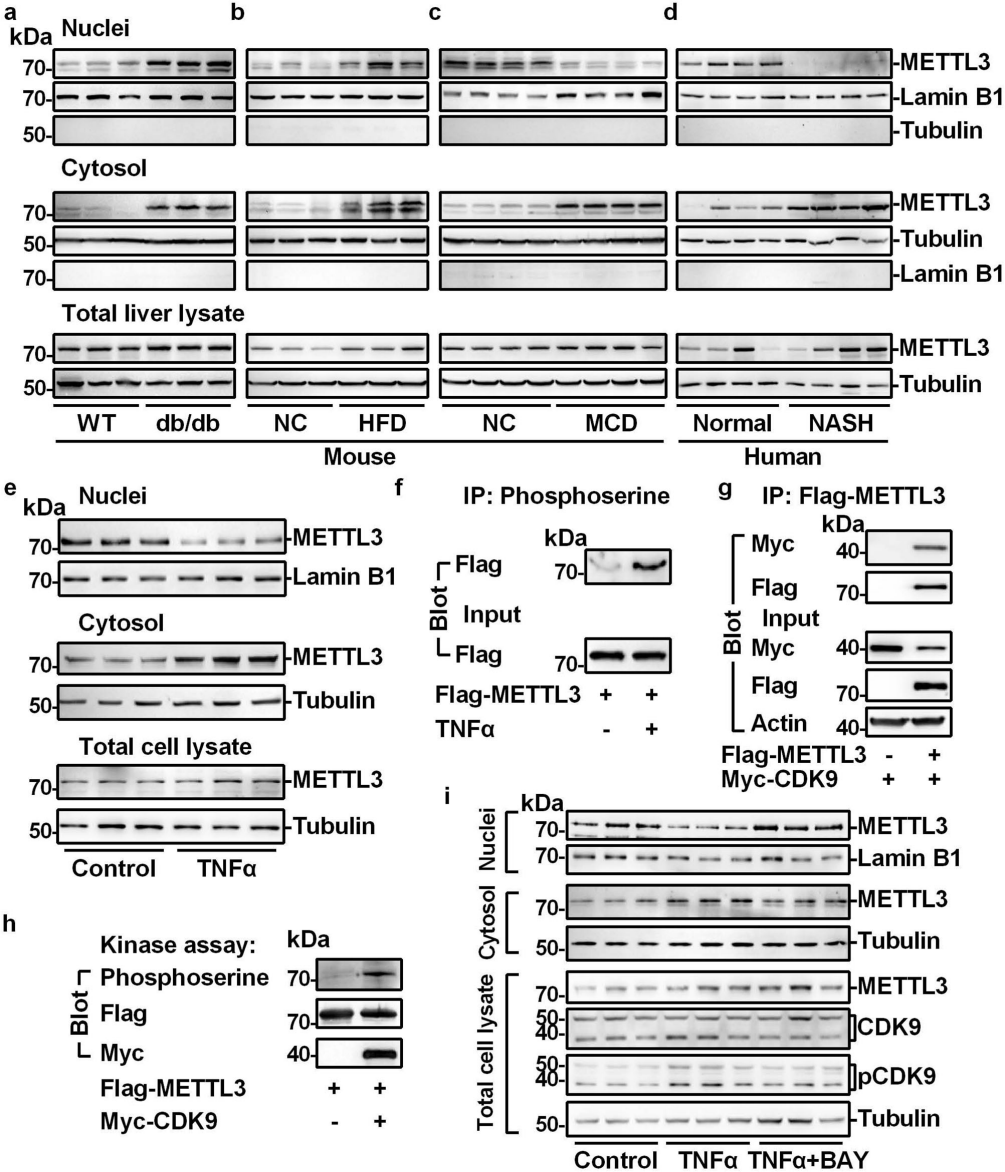
Nuclear METTL3 is decreased in the NASH livers. **a**–**c** METTL3 protein levels in nuclei, cytosol, and total cell lysate from the livers of db/db (11 weeks old) mice and HFD (for 8 weeks)- or MCD (for 3 weeks)-fed mice were measured by immunoblotting. The quantification was shown in Supplementary Fig. 1d–f. **d** Immunoblotting of METTL3 protein levels in nuclei, cytosol, and total cell lysate from human NASH and normal liver tissues. The quantification was shown in Supplementary Fig. 1g. **e** Primary hepatocytes were isolated and treated with TNFα (20 ng/ml) for 2 h. METTL3 protein levels in nuclei, cytosol, and total cell lysate were measured by immunoblotting. The quantification was shown in Supplementary Fig. 1i. **f** Primary hepatocytes were infected with Ad-Flag-METTL3 adenovirus, and then treated with or without TNFα (20 ng/ml) for 2 h. The total cell lysate was immunoprecipitated with anti-phosphoserine antibody and immunoblotted with anti-Flag antibody. **g** Myc-CDK9 expression vector was co-transfected with or without Flag-METTL3 expression vector in HEK293T cells. Total cell lysates were immunoprecipitated with Flag beads and then immunoblotted with anti-Myc or anti-Flag antibodies. **h** In vitro kinase assay. **i** Primary hepatocytes were treated with vehicle, TNFα (20 ng/ml), and TNFα (20 ng/ml) plus BAY-1143572 (2 μM) for 2 h. In TNFα plus BAY-1143572 group, primary hepatocytes were pretreated with BAY-1143572 (2 μM) for 2 h. METTL3 protein levels in nuclei, cytosol, and total cell lysate were measured by immunoblotting. CDK9, p-CDK9, Lamin B1, and Tubulin levels were also measured by immunoblotting. The quantification was shown in Supplementary Fig. 1m–n. The samples were derived from the same experiment and the blots were processed in parallel. *n* was the number of biologically independent mice or cell samples. The cell culture experiments were repeated three times independently with similar results. Source data are provided as a Source Data file.

To confirm the RNA-seq data, we performed qPCR assays. *Mettl3* mRNA levels were increased in livers from both db/db and HFD-fed mice but were not increased in the livers of MCD-fed mice (Supplementary Fig. 1a–c). METTL3 was primarily located in the nucleus^18,38^. To test whether METTL3 displays different subcellular locations in NAFL and NASH, we measured the METTL3 protein levels in the nuclei, cytosol, and total cell lysates from the livers of db/db, HFD-fed, and MCD-fed mice by immunoblotting. As shown in Fig. 1a and Supplementary Fig. 1d, METTL3 protein levels in the nuclei, cytosol, and total cell lysates from the livers of db/db mice were largely increased. Similarly, HFD feeding significantly increased METTL3 protein levels in the nuclei, cytosol, and total cell lysates by more than twofold (Fig. 1b and Supplementary Fig. 1e). This observation is consistent with a previous study^39^. However, METTL3 protein levels in the nuclei of MCD-fed mouse livers were dramatically decreased by 80% but were significantly increased by 10.9-fold in the cytosol (Fig. 1c and Supplementary Fig. 1f). Consistently, in human patients with NASH, we observed similar phenotypes (Fig. 1d and Supplementary Fig. 1g). METTL3 protein levels in the nuclei of NASH livers were dramatically decreased by 71.2% but were significantly increased by 2.84-fold in the cytosol (Fig. 1d and Supplementary Fig. 1g). These data indicate that downregulation of nuclear METTL3 protein levels may contribute to the NAFL-to-NASH transition.

To further test how METTL3 is translocated from the nucleus to the cytosol in NASH, primary hepatocytes were isolated and treated with palmitic acid (PA) or TNFα, as it is known that PA administration can mimic fatty liver disease and that TNFα can induce liver inflammation^40,41^. As shown in Supplementary Fig. 1h, PA did not induce the translocation of METTL3, whereas TNFα was able to increase cytosolic METTL3 accumulation and decrease nuclear METTL3 levels (Fig. 1e and Supplementary Fig. 1i). Phosphorylation has been shown to regulate nuclear localization^42,43^. We observed that TNFα was able to induce the serine phosphorylation of METTL3 (Fig. 1f), which may contribute to the inhibition of METTL3 nuclear localization. Next, we investigated which protein kinase was responsible for the mediation of TNFα-induced phosphorylation of METTL3. Protein interaction data in the neXtProt human protein knowledgebase and IntAct database showed that two protein kinases, CDK9 and PRKDC, might interact with METTL3^44,45^. Co-immunoprecipitation (Co-IP) experiments performed in HEK293T cells showed that CDK9 interacted with METTL3 (Fig. 1g), and an in vitro kinase assay showed that CDK9 was able to phosphorylate METTL3 (Fig. 1h). We observed that TNFα also induced the phosphorylation of CDK9 (Supplementary Fig. 1j and Fig. 1i), and p-CDK9 levels were increased in NASH livers (Supplementary Fig. 1k–l), which further indicates that CDK9 may be involved in the nuclear/cytosolic translocation of METTL3 in NASH. Next, we asked whether inhibition of CDK9 can block the TNFα-induced cytosolic accumulation of METTL3. As shown in Fig. 1i and Supplementary Fig. 1m–n, BAY-1143572, a CDK9 inhibitor, blocked both TNFα-induced p-CDK9 and the nuclear/cytosolic translocation of METTL3. These data indicate that TNFα/CDK9-mediated phosphorylation of METTL3 may contribute to the reduction of nuclear METTL3 levels in NASH livers.

### Hepatic deletion of *Mettl3* exacerbates both HFD and MCD -induced NASH

**Fig. 2:**
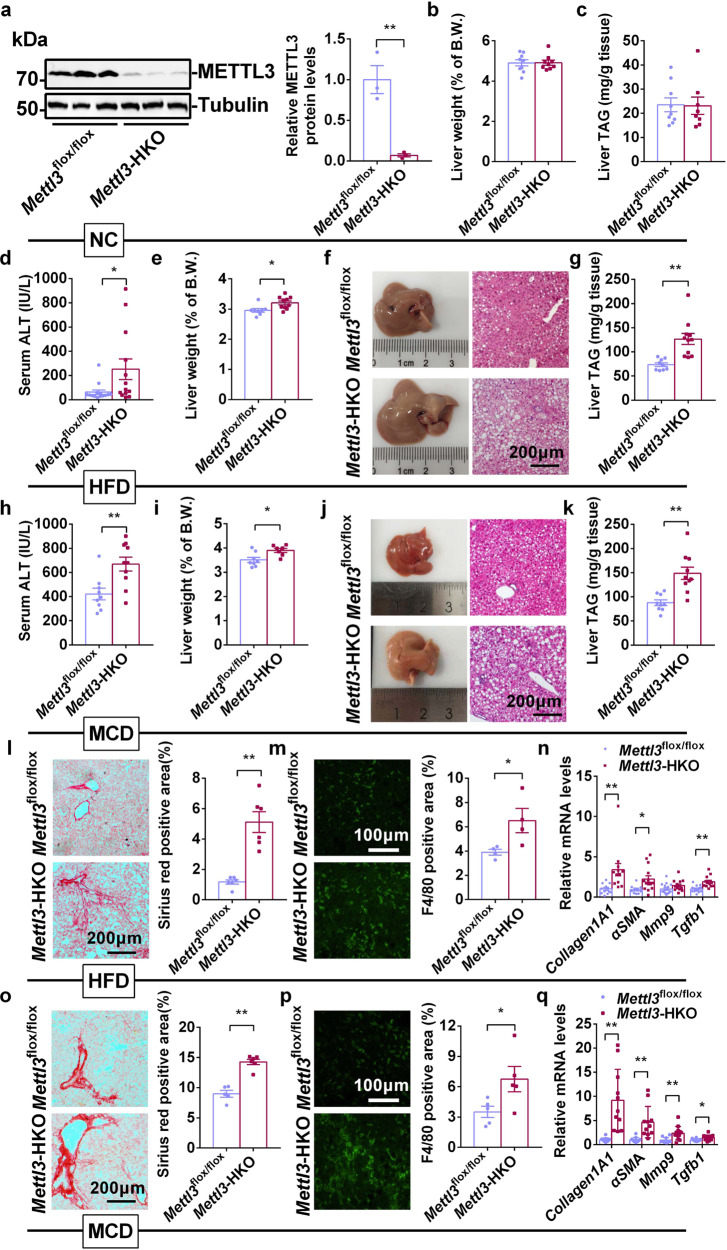
Hepatic deletion of *Mettl3* accelerates diet-induced NASH. **a** METTL3 protein levels in livers of *Mettl3*^flox/flox^ and *Mettl3-*HKO mice at 8 weeks old (*n* = 3 for each group; *P* = 0.0059). The samples were derived from the same experiment and the blots were processed in parallel. **b** Liver weights of *Mettl3*^flox/flox^ and *Mettl3-*HKO mice at 11 weeks old (*n* = 9 for each group). **c** Liver TAG levels in *Mettl3*^flox/flox^ and *Mettl3-*HKO mice at 11 weeks old (*Mettl3*^flox/flox^, *n* = 9; *Mettl3-*HKO, *n* = 8). **d** Serum ALT activity in *Mettl3*^flox/flox^ and *Mettl3-*HKO mice fed an HFD for 12 weeks (*Mettl3*^flox/flox^, *n* = 14; *Mettl3-*HKO, *n* = 13; *P* = 0.0332). **e** Liver weights of *Mettl3*^flox/flox^ and *Mettl3-*HKO mice fed an HFD for 12 weeks (*Mettl3*^flox/flox^, *n* = 8; *Mettl3-*HKO, *n* = 10; *P* = 0.0135). **f** Representative pictures and H&E staining of livers from *Mettl3*^flox/flox^ and *Mettl3-*HKO mice fed an HFD for 12 weeks. **g** Liver TAG levels in *Mettl3*^flox/flox^ and *Mettl3-*HKO mice fed an HFD for 12 weeks (*Mettl3*^flox/flox^, *n* = 9; *Mettl3-*HKO, *n* = 11; *P* = 0.0007). **h** Serum ALT activity in *Mettl3*^flox/flox^ and *Mettl3-*HKO mice fed an MCD for 3 weeks (*Mettl3*^flox/flox^, *n* = 9; *Mettl3-*HKO, *n* = 10; *P* = 0.0046). **i** Liver weights of *Mettl3*^flox/flox^ and *Mettl3-*HKO mice fed an MCD for 3 weeks (*Mettl3*^flox/flox^, *n* = 8; *Mettl3-*HKO, *n* = 7; *P* = 0.0127). **j** Representative pictures and H&E staining of livers from *Mettl3*^flox/flox^ and *Mettl3-*HKO mice fed an MCD for 3 weeks. **k** Liver TAG levels in *Mettl3*^flox/flox^ and *Mettl3-*HKO mice fed an MCD for 3 weeks (*Mettl3*^flox/flox^, *n* = 9; *Mettl3-*HKO, *n* = 10; *P* = 0.0006). **l** Sirius Red staining of liver sections from *Mettl3*^flox/flox^ and *Mettl3-*HKO mice fed an HFD for 12 weeks (*n* = 6 for each group; *P* = 0.0002). **m** F4/80 immunostaining of liver sections from *Mettl3*^flox/flox^ and *Mettl3-*HKO mice fed an HFD for 12 weeks (*n* = 4 for each group; *P* = 0.0439). **n** RT-qPCR analysis of mRNA levels in the livers of *Mettl3*^flox/flox^ and *Mettl3-*HKO mice fed an HFD for 12 weeks (*Mettl3*^flox/flox^, *n* = 10–15; *Mettl3-*HKO, *n* = 12–14; *Collagen1A1*, *P* = 0.0061; *αSMA*, *P* = 0.0163; *Mmp9*, *P* = 0.0884; *Tgfb1*, *P* = 0.0001). **o** Sirius Red staining of liver sections from *Mettl3*^flox/flox^ and *Mettl3-*HKO mice fed an MCD for 3 weeks (*Mettl3*^flox/flox^, *n* = 5; *Mettl3-*HKO, *n* = 6; *P* = 0.00004). **p** F4/80 immunostaining of liver sections from *Mettl3*^flox/flox^ and *Mettl3-*HKO mice fed an MCD for 3 weeks (*n* = 5 for each group; *P* = 0.0446). **q** RT-qPCR analysis of mRNA levels in the livers of *Mettl3*^flox/flox^ and *Mettl3-*HKO mice fed an MCD for 3 weeks (*Mettl3*^flox/flox^, *n* = 10–11; *Mettl3-*HKO, *n* = 11–12; *Collagen1A1*, *P* = 0.0004; *αSMA*, *P* = 0.0015; *Mmp9*, *P* = 0.0062; *Tgfb1*, *P* = 0.0151). *n* was the number of biologically independent mice. Data represent the mean ± SEM. Significance was determined by unpaired two-tailed Student’s *t* test analysis. *P < 0.05. **P < 0.01. Source data are provided as a Source Data file.

To determine whether METTL3 regulates the NAFL-to-NASH transition, we generated hepatocyte-specific *Mettl3* knockout (HKO) mice by crossing *Mettl3*^flox/flox^ mice with *Alb*-*Cre* transgenic mice. The genotype of the *Mettl3*-HKO mice was *Mettl3*^flox/flox^ *Alb*-*Cre*^+/−^. As expected, METTL3 protein levels were dramatically decreased by 93.2% in the livers of *Mettl3*-HKO mice (Fig. 2a). We did not observe any differences in body weights (Supplementary Fig. 2a), serum triacylglycerol (TAG) levels (Supplementary Fig. 2b), liver weights (Supplementary Fig. 2c), or liver TAG levels (Supplementary Fig. 2d) between *Mettl3*^flox/flox^ and *Alb*-*Cre* mice. *Mettl3*^flox/flox^ mice also displayed similar MCD-induced NASH as *Alb*-*Cre* mice, as revealed by similar body weight (Supplementary Fig. 2e), serum TAG levels (Supplementary Fig. 2f), liver weights (Supplementary Fig. 2g), and liver TAG levels (Supplementary Fig. 2h). Therefore, we used *Mettl3*^flox/flox^ mice as the control for *Mettl3*-HKO mice in the following experiments. When normal chow was provided, the liver weights and TAG levels were comparable between *Mettl3*-HKO and *Mettl3*^flox/flox^ mice at 11 weeks old (Fig. 2b, c). However, after they were fed an HFD for 12 weeks, *Mettl3*-HKO mice displayed more severe NAFL, as revealed by higher serum ALT activities (Fig. 2d), higher liver weights (Fig. 2e, f), more hepatic lipid droplets (Fig. 2f), and higher liver TAG levels (Fig. 2g) compared with *Mettl3*^flox/flox^ mice. To further test whether METTL3 is involved in NASH progression, *Mettl3*-HKO and *Mettl3*^flox/flox^ mice were fed an MCD for 3 weeks. Consistently, *Mettl3*-HKO mice also exhibited higher serum ALT activities (Fig. 2h), higher liver weights (Fig. 2i, j), more hepatic lipid droplets (Fig. 2j), and higher liver TAG levels (Fig. 2k), which indicates that *Mettl3*-HKO mice are more sensitive to MCD-induced NASH.

Human patients with NASH have a high risk of developing liver fibrosis^5^. To address whether the increased steatohepatitis in *Mettl3*-HKO mice also promotes liver fibrosis and immune cell infiltration, we measured pathological collagen deposition and immune cell infiltration using Sirius Red staining and F4/80 immunostaining, respectively. Liver sections from *Mettl3*-HKO mice contained significantly larger Sirius Red-positive areas than *Mettl3*^flox/flox^ mice under both HFD- and MCD-feeding conditions (Fig. 2l, o). Liver sections from *Mettl3*-HKO mice contained significantly more F4/80-positive areas than *Mettl3*^flox/flox^ mice under both HFD- and MCD-feeding conditions (Fig. 2m, p). Consistently, the expression of fibrosis markers (*collagen IA1* and *aSMA*) and profibrogenic factor (*Tgfb1*) was significantly increased in *Mettl3*-HKO mice fed either an HFD (Fig. 2n) or MCD (Fig. 2q). These data indicate that hepatic deletion of *Mettl3* accelerates the progression from NASH to liver fibrosis.

### Hepatic deletion of *Mettl3* accelerates free fatty-acid uptake by increasing CD36 expression

**Fig. 3:**
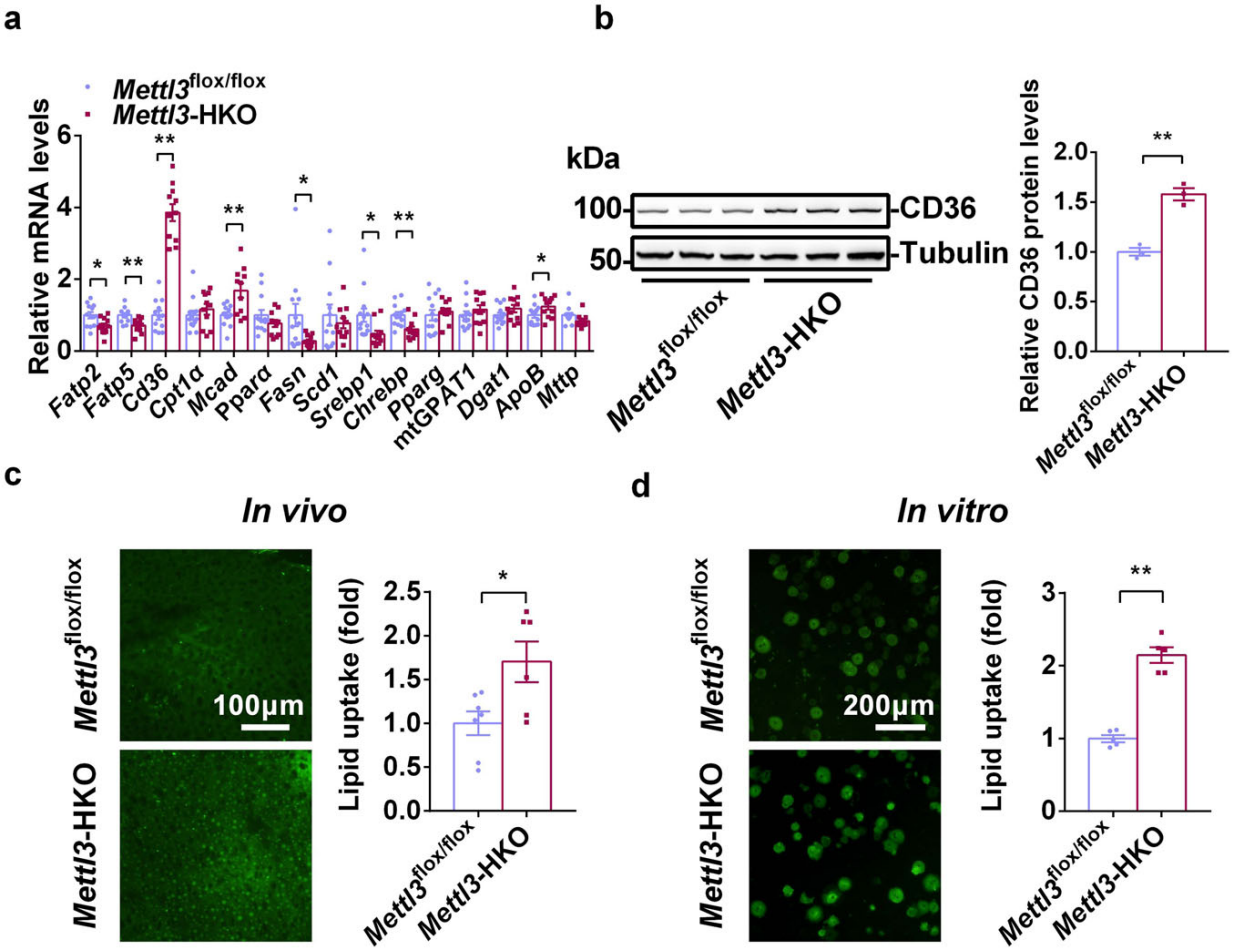
Hepatic deletion of *Mettl3* enhances CD36-mediated free fatty-acid uptake. **a** Relative mRNA levels in livers of *Mettl3*^flox/flox^ and *Mettl3-*HKO mice at 8 weeks old were determined by RT-qPCR (*Mettl3*^flox/flox^, *n* = 12–13; *Mettl3-*HKO, *n* = 11; *Fatp2*, *P* = 0.0171; *Fatp5*, *P* = 0.0029; *Cd36*, *P* < 0.00001; *Cpt1α*, *P* = 0.4018; *Mcad*, *P* = 0.0025; *Pparα*, *P* = 0.2124; *Fasn*, *P* = 0.0393; *Scd1*, *P* = 0.5119; *Srebp1*, *P* = 0.02599; *Chrebp*, *P* = 0.00298; *Pparg*, *P* = 0.6043; *mtGPAT1*, *P* = 0.358; *Dgat1*, *P* = 0.1546; *ApoB*, *P* = 0.0453; *Mttp*, *P* = 0.0594). **b** CD36 protein levels in *Mettl3*^flox/flox^ and *Mettl3-*HKO mice at 8 weeks age were measured by immunoblotting. CD36 protein levels were quantified by ImageJ and normalized to Tubulin (*n* = 3 for each group; *P* = 0.0014). The samples were derived from the same experiment and the blots were processed in parallel. **c** Representative BODIPY FL C16 fluorescence image and relative lipid uptake levels in *Mettl3*^flox/flox^ and *Mettl3-*HKO mice at 8 weeks old (*Mettl3*^flox/flox^, *n* = 7; *Mettl3-*HKO, *n* = 6; *P* = 0.02). **d** Representative BODIPY FL C16 fluorescence image and relative lipid uptake levels in primary hepatocytes isolated from *Mettl3*^flox/flox^ and *Mettl3-*HKO mice at 8 weeks old (*n* = 5 for each group; *P* = 0.00001). *n* was the number of biologically independent mice or cell samples. The cell culture experiments were repeated for three times independently with similar results. Data represent the mean ± SEM. Significance was determined by unpaired two-tailed Student’s *t* test analysis. *P < 0.05. **P < 0.01. Source data are provided as a Source Data file.

Hepatic lipid accumulation contributes to NASH progression^5,11^. Liver steatosis results from an imbalance among free fatty acid uptake, lipogenesis, fatty acid β-oxidation, and very low-density lipoprotein (VLDL) secretion^8,46^. To determine which process is responsible for hepatic lipid accumulation in *Mettl3*-HKO mice, the expression of genes related to free fatty acid uptake (*Fatp2*, *Fatp5,* and *Cd36*), fatty acid β-oxidation (*Cpt1α*, *Mcad,* and *Ppara*), lipogenesis (*Fasn*, *Scd1*, *Srebp1*, *Chrebp*, *Pparg*, *mtGPAT1,* and *Dgat1*) and VLDL secretion (*ApoB* and *Mttp*) was measured by real-time quantitative PCR (RT-qPCR). As shown in Fig. 3a, *Cd36* mRNA levels were dramatically increased whereas those of *Fasn*, *Srebp1*, *Chrebp*, *Fatp2,* and *Fatp5* were decreased, which indicates that CD36-mediated free fatty acid uptake was increased whereas lipogenesis was decreased in the livers of *Mettl3*-HKO mice. *Mcad* and *ApoB* mRNA levels were slightly increased whereas *Cpt1a*, *Ppara,* and *Mttp* mRNA levels were not altered in the livers of *Mettl3*-HKO mice. These data suggest that fatty acid β-oxidation, lipogenesis, and VLDL secretion do not contribute to the increased liver steatosis seen in *Mettl3*-HKO mice and suggest that increased CD36-mediated hepatic free fatty-acid uptake leads to more severe NALF and NASH in *Mettl3*-HKO mice. Consistent with the increased mRNA levels, CD36 protein levels were also significantly increased in the livers of *Mettl3*-HKO mice (Fig. 3b). To further test whether free fatty acid uptake is increased in *Mettl3*-HKO mice, we measured free fatty-acid uptake both in vivo and in vitro. As shown in Fig. 3c, acute injection of the fluorescent palmitate analog BODIPY FL C_16_ into *Mettl3*-HKO and *Mettl3*^flox/flox^ mice resulted in a 1.7-fold increase in free fatty acid uptake by the liver in *Mettl3*-HKO mice. To further verify whether hepatic METTL3 regulates free fatty-acid uptake in a cell-autonomous manner, primary hepatocytes were isolated from *Mettl3*-HKO and *Mettl3*^flox/flox^ mice, and BODIPY FL C_16_ uptake experiments were performed. As shown in Fig. 3d, free fatty-acid uptake was significantly increased by 2.1-fold according to the measurement of intracellular fluorescence of BODIPY FL C_16_. These data demonstrate that increased CD36-mediated free fatty acid uptake contributes to the increased liver steatosis seen in *Mettl3*-HKO mice.

### Liver-specific knockout of *Mettl3* enhances liver injury

**Fig. 4:**
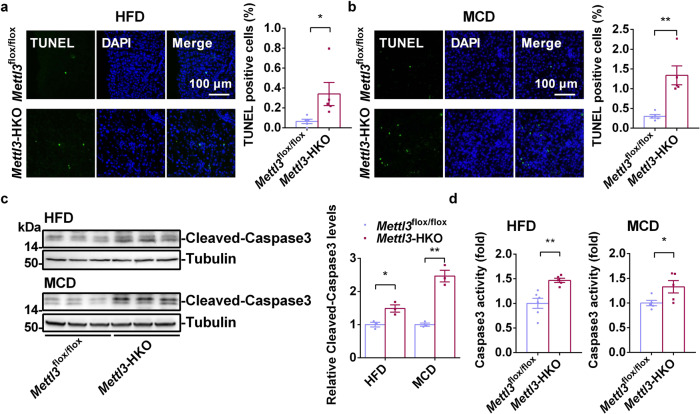
Liver-specific Knockout of *Mettl3* accelerates diet-induced liver injury. **a** The TUNEL-positive cells in *Mettl3*^flox/flox^ and *Mettl3-*HKO mice fed an HFD for 12 weeks (*n* = 5 for each group; *P* = 0.0474). **b** The TUNEL-positive cells in *Mettl3*^flox/flox^ and *Mettl3-*HKO mice fed an MCD for 3 weeks (*n* = 5 for each group; *P* = 0.0027). **c** Cleaved caspase3 levels were measured by immunoblotting in the livers of *Mettl3*^flox/flox^ and *Mettl3-*HKO mice fed either an HFD or MCD. Cleaved caspase3 levels were quantified by ImageJ and normalized to Tubulin (*n* = 3 for each group; HFD, *Mettl3*^flox/flox^ versus *Mettl3-*HKO, *P* = 0.0197; MCD, *Mettl3*^flox/flox^ versus *Mettl3-*HKO, *P* = 0.0013). The samples were derived from the same experiment and the blots were processed in parallel. **d** Caspase3 activity in the livers of *Mettl3*^flox/flox^ and *Mettl3-*HKO mice fed either an HFD or MCD was measured by using a Caspase3 Assay Kit (ab39383, Abcam) following the instruction (*Mettl3*^flox/flox^, *n* = 6 for HFD, *n* = 5 for MCD; *Mettl3-*HKO, *n* = 5; HFD, *Mettl3*^flox/flox^ versus *Mettl3-*HKO, *P* = 0.0034; MCD, *Mettl3*^flox/flox^ versus *Mettl3-*HKO, *P* = 0.0433). *n* was the number of biologically independent mice. Data represent the mean ± SEM. Significance was determined by unpaired two-tailed Student’s *t* test analysis. *P < 0.05. **P < 0.01. Source data are provided as a Source Data file.

Increased hepatic steatosis is not sufficient to induce NASH^5^. For this reason, an HFD alone cannot induce NASH. Surprisingly, HFD feeding was sufficient to induce NASH in *Mettl3*-HKO mice. Liver injury and inflammation have been shown to be key regulators of the NAFL-to-NASH transition^10^. Serum ALT activities were significantly increased in *Mettl3*-HKO mice fed either an HFD or MCD, which suggests that hepatic deletion of *Mettl3* accelerates diet-induced liver injury and inflammation. We measured hepatocyte apoptosis using terminal deoxynucleotidyl transferase dUTP nick end labeling (TUNEL) assays in both HFD- and MCD-fed mice. The number of TUNEL-positive cells was significantly increased in both HFD- and MCD- fed *Mettl3*-HKO mice (Fig. 4a, b). In addition, cleaved caspase3 and its activity were much higher in *Mettl3*-HKO mice (Fig. 4c, d), which indicates that *Mettl3*-HKO mice displayed more severe liver injury after they were fed an HFD or MCD.

### Hepatic deletion of *Mettl3* increases liver inflammation by enhancing CCL2 expression

**Fig. 5:**
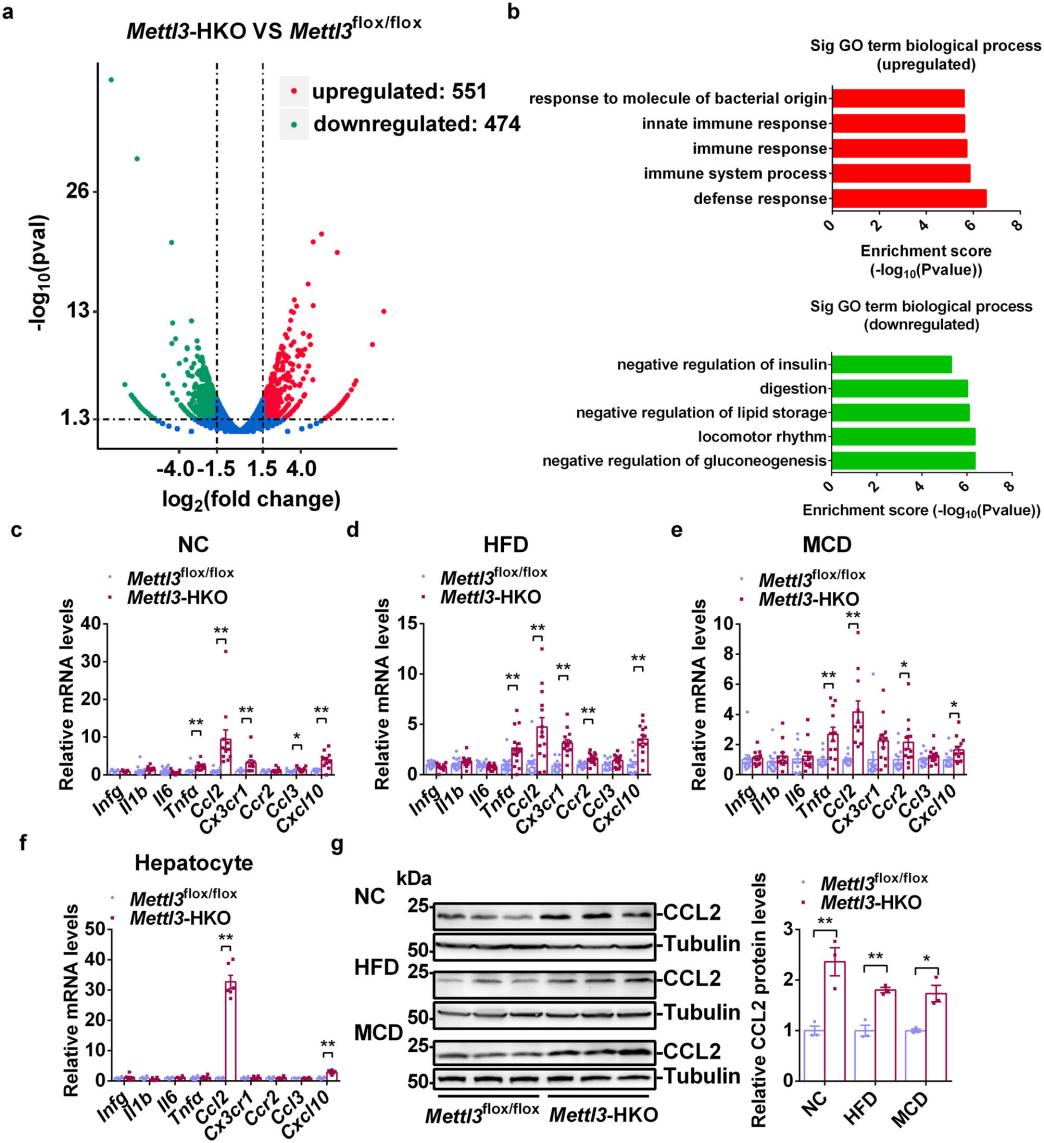
Liver-specific Knockout of *Mettl3* increases liver inflammation. **a** RNA-seq analysis was performed in the livers of *Mettl3*^flox/flox^ and *Mettl3-*HKO mice at 8 weeks old. The differentially expressed genes (DEGs) (HKO VS ff) including 474 downregulated genes and 551 upregulated genes were illustrated in a volcanoplot (|log2foldchange|>1.5 and *p*val < 0.05). **b** Top GO biological process terms enriched in downregulated and upregulated genes. **c** Real-time qPCR analysis of mRNA levels in the livers of *Mettl3*^flox/flox^ and *Mettl3-*HKO mice at 8 weeks old (*Mettl3*^flox/flox^, *n* = 12, 13; *Mettl3-*HKO, *n* = 11; *Infg*, *P* = 0.4526; *Il1b*, *P* = 0.1869; *Il6*, *P* = 0.2102; *Tnfα*, *P* = 0.0056; *Ccl2*, *P* = 0.0024; *Cxcr1*, *P* = 0.0085; *Ccr2*, *P* = 0.5215; *Ccl3*, *P* = 0.0461; *Cxcl10*, *P* = 0.0001). **d** RT-qPCR analysis of mRNA levels in the livers of *Mettl3*^flox/flox^ and *Mettl3-*HKO mice fed an HFD for 12 weeks (*Mettl3*^flox/flox^, *n* = 13–16; *Mettl3-*HKO, *n* = 14; *Infg*, *P* = 0.0237; *Il1b*, *P* = 0.0977; *Il6*, *P* = 0.0353; *Tnfα*, *P* = 0.0042; *Ccl2*, *P* = 0.0008; *Cxcr1*, *P* < 0.0001; *Ccr2*, *P* = 0.0008; *Ccl3*, *P* = 0.0777; *Cxcl10*, *P* < 0.0001). **e** RT-qPCR analysis of mRNA levels in the livers of *Mettl3*^flox/flox^ and *Mettl3-*HKO mice fed an MCD for 3 weeks (*Mettl3*^flox/flox^, *n* = 11, 12; *Mettl3-*HKO, *n* = 11, 12; *Infg*, *P* = 0.7685; *Il1b*, *P* = 0.3087; *Il6*, *P* = 0.6141; *Tnfα*, *P* = 0.0033; *Ccl2*, *P* = 0.0003; *Cxcr1*, *P* = 0.0753; *Ccr2*, *P* = 0.0306; *Ccl3*, *P* = 0.2095; *Cxcl10*, *P* = 0.0381). **f** Real-time qPCR analysis of mRNA levels in primary hepatocytes isolated from *Mettl3*^flox/flox^ and *Mettl3-*HKO mice at 8 weeks old (*n* = 6 for each group; *Infg*, *P* = 0.7077; *Il1b*, *P* = 0.1698; *Il6*, *P* = 0.6845; *Tnfα*, *P* = 0.6021; *Ccl2*, *P* < 0.0001; *Cxcr1*, *P* = 0.6532; *Ccr2*, *P* = 0.7745; *Ccl3*, *P* = 0.484; *Cxcl10*, *P* < 0.0001). **g** CCL2 protein levels were measured by immunoblotting in the livers of *Mettl3*^flox/flox^ and *Mettl3-*HKO mice fed NC, HFD and MCD, respectively. CCL2 protein levels were then quantified by ImageJ and normalized to Tubulin (*n* = 3 for each group; NC, *P* = 0.0096; HFD, *P* = 0.0026; MCD, *P* = 0.0117). The samples were derived from the same experiment and the blots were processed in parallel. *n* was the number of biologically independent mice. Data represent the mean ± SEM. Significance was determined by unpaired two-tailed Student’s *t* test analysis. *P < 0.05. **P < 0.01. Source data are provided as a Source Data file.

To comprehensively compare the gene expression profiles in the livers of *Mettl3*-HKO and *Mettl3*^flox/flox^ mice, we performed RNA-sequencing (RNA-seq) analysis. As shown in Fig. 5a, a total of 551 genes were upregulated, and 474 genes were downregulated. Gene Ontology (GO) analysis showed that genes related to defense response, immune system process, immune response, and innate immune response were significantly increased, whereas those associated with negative regulation of gluconeogenesis, locomotor rhythm, negative regulation of lipid storage, and digestion were downregulated (Fig. 5b). *Cd36* was one of the upregulated genes, which was confirmed above by RT-qPCR and immunoblotting (Fig. 3a, b). RT-qPCR analysis further confirmed other upregulated genes. As shown in Fig. 5c–e, *Ccl2*, *Cx3cr1*, *Ccr2*, *Ccl3*, *Cxcl10,* and *Tnfa* mRNA levels were increased in *Mettl3*-HKO mice, but the expression levels of other inflammatory genes such as *Infg, Il1b,* and *Il6* were unchanged. To further determine whether hepatic METTL3 regulates the expression of inflammatory genes in a cell-autonomous manner, primary hepatocytes were isolated from *Mettl3*-HKO and *Mettl3*^flox/flox^ mice, and gene expression was measured by RT-qPCR. As shown in Fig. 5f, *Ccl2* was the most upregulated gene in primary hepatocytes isolated from *Mettl3-*HKO mice. Furthermore, CCL2 protein levels were also significantly increased in the livers of *Mettl3-*HKO mice fed the three types of diets (normal chow, HFD, and MCD) (Fig. 5g). These data suggest that hepatic upregulation of CCL2 in *Mettl3-*HKO mice contributes to more severe NASH.

### Inhibition of CD36 and CCL2 ameliorates NASH progression in *Mettl3*-HKO mice

**Fig. 6:**
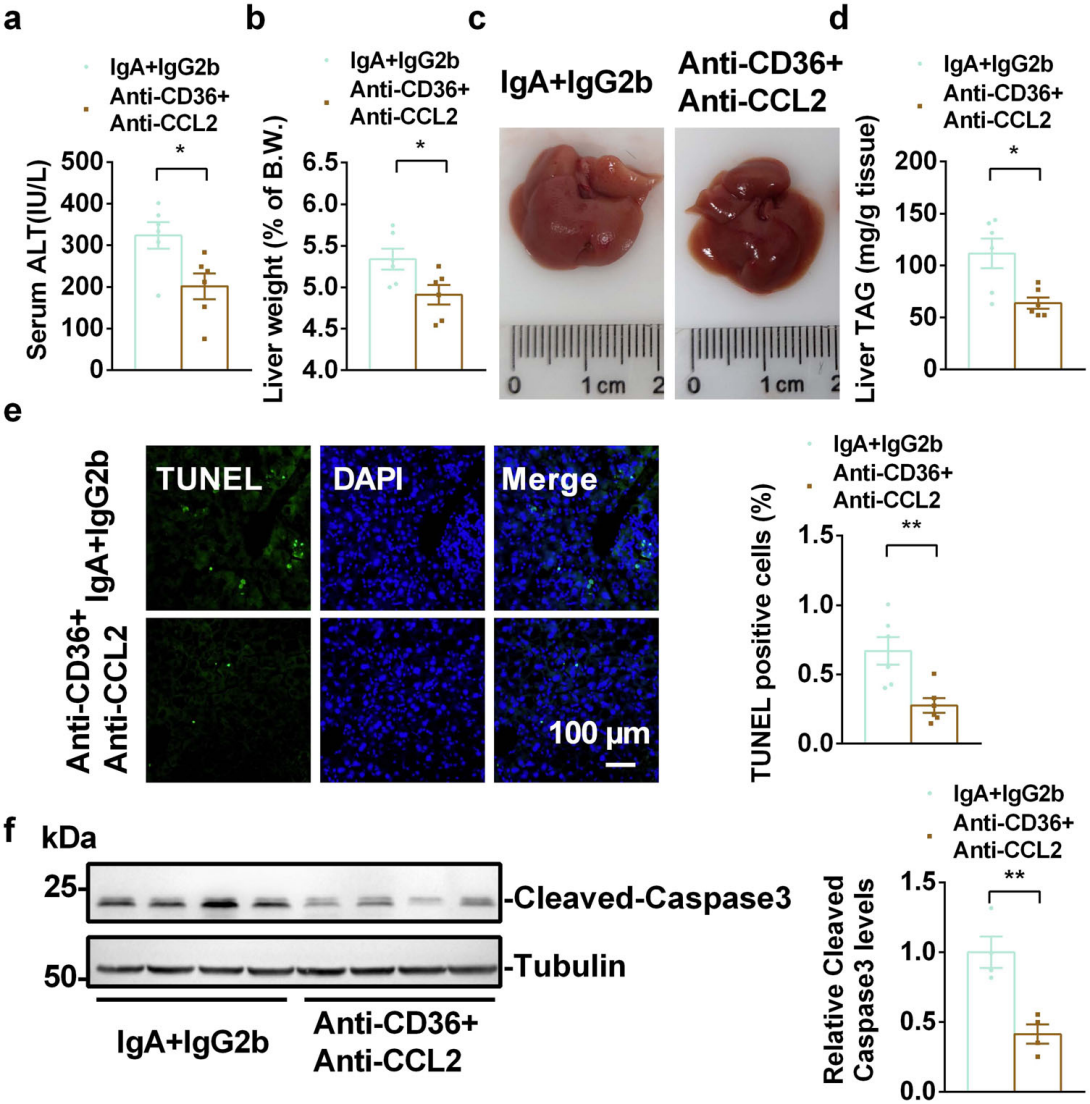
Neutralization of CD36 and CCL2 ameliorates NASH progression in *Mettl3-*HKO mice. *Mettl3-*HKO mice at 8 weeks old were treated with a combination of anti-CD36 and anti-CCL2 antibodies (4 μg/antibody/mouse, i.v.) or an equal amount of control antibodies (IgA and IgG2b) on day 1, 4, 7, and 8 after MCD feeding. Mice were killed on day 9 after MCD feeding. **a** Serum ALT activity (*n* = 6 for each group; *p* = 0.0204). **b** Liver weights (*n* = 6 for each group; *P* = 0.0325). **c** Representative liver pictures. **d** Liver TAG levels (*n* = 6 for each group; *P* = 0.0108). **e** TUNEL-positive cells (*n* = 6 for each group; *P* = 0.0059). **f** The cleavage of caspase3 was measured by immunoblotting, quantified by ImageJ, and normalized to Tubulin (*n* = 4 for each group; *P* = 0.0043). The samples were derived from the same experiment and the blots were processed in parallel. *n* was the number of biologically independent mice. Data represent the mean ± SEM. Significance was determined by unpaired two-tailed Student’s *t* test analysis. *P < 0.05. **P < 0.01. Source data are provided as a Source Data file.

To further confirm that CD36 and CCL2 contribute to NASH progression in *Mettl3-*HKO mice, *Mettl3-*HKO mice were fed an MCD diet and treated with a combination of anti-CD36 and anti-CCL2-neutralizing antibodies. Interestingly, inhibition of CD36 and CCL2 ameliorated NASH progression in *Mettl3-*HKO mice, as revealed by lower serum ALT activity (Fig. 6a), lower liver weights (Fig. 6b), normal morphology of the liver (Fig. 6c), lower liver TAG levels (Fig. 6d), fewer TUNEL-positive cells (Fig. 6e), and lower caspase3 cleavage (Fig. 6f). These data demonstrate that antibody blockade of CD36 and CCL2 prevents the NASH progression in *Mettl3-*HKO mice and further supports the theory that elevated CD36 and CCL2 contribute to NASH progression in *Mettl3-*HKO mice.

### Hepatic overexpression of *Mettl3* ameliorates MCD-induced NASH

**Fig. 7:**
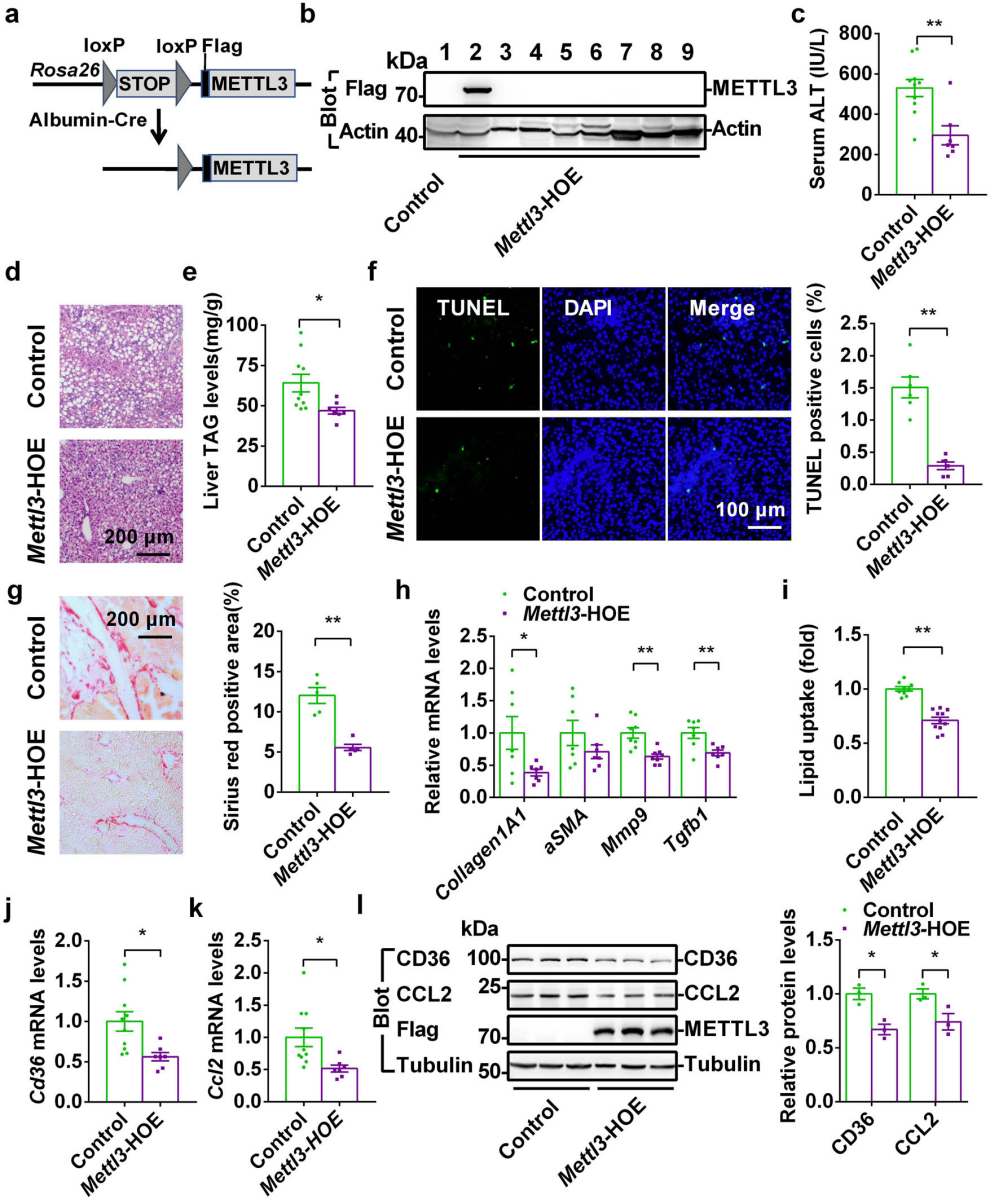
Hepatic overexpression of *Mettl3* ameliorates MCD-induced NASH. **a** A schematic representation of the *Rosa26*-STOP-METTL3 targeting vector and generation of liver-specific overexpression of *Mettl3* mice. **b** Tissue extracts from control and *Mettl3*-HOE mice were immunoblotted with antibodies to Flag and β-actin, respectively. Lane 1 Control liver; lane 2 HOE liver; lane 3 HOE White adipose tissue; lane 4 HOE Skeletal muscle; lane 5 HOE Brown adipose tissue; lane 6 HOE Heart; lane 7 HOE Spleen; lane 8 HOE Kidney, lane 9 HOE Brain. **c** Serum ALT activities (control, *n* = 10; *Mettl3*-HOE, *n* = 7; *P* = 0.0024). **d** H&E staining of liver from control and *Mettl3*-HOE mice fed an MCD for 2 weeks. **e** Liver TAG levels (control, *n* = 10; *Mettl3*-HOE, *n* = 7; *P* = 0.0243). **f** TUNEL-positive cells (*n* = 6 for each group; *P* = 0.00003). **g** Sirius Red staining of livers from control and *Mettl3*-HOE mice fed an MCD for 2 weeks (*n* = 5 for each group). **h** Relative mRNA levels (control, *n* = 7–8; *Mettl3*-HOE, *n* = 7–8; collagen1A1, *P* = 0.0365; αSMA, *P* = 0.2169; Mmp9, *P* = 0.0011; Tgfb1, *P* = 0.0072). **i** The relative lipid uptake in control and *Mettl3*-HOE mice at 8 weeks old (control, *n* = 9; *Mettl3*-HOE, *n* = 11; *P* < 0.0001). **j**, **k** *Cd36* and *Ccl2* mRNA levels (control, *n* = 10; *Mettl3*-HOE, *n* = 7; *Cd36*, *P* = 0.0121; *Ccl2*, *P* = 0.0178). **l** CD36 and CCL2 protein levels were measured by immunoblotting, quantified by ImageJ and normalized to Tubulin (*n* = 3 for each group; CD36, *P* = 0.0101; CCL2, *P* = 0.0427). *n* was the number of biologically independent mice. The samples were derived from the same experiment and the blots were processed in parallel. Data represent the mean ± SEM. Significance was determined by unpaired two-tailed Student’s *t* test analysis. *P < 0.05. **P < 0.01. Source data are provided as a Source Data file.

Next, we asked whether hepatic overexpression of *Mettl3* can ameliorate MCD-induced NASH. We generated *STOP-Mettl3* mice, in which a *STOP-Flag-Mettl3* cassette was inserted in the *Rosa26* allele using the CRISPR-Cas9 technique (Fig. 7a and Supplementary Fig. 3a). Hepatocyte-specific *Mettl3*-overexpressing (HOE) mice were generated by crossing *STOP-Mettl3*^*+/−*^ mice with *Alb-Cre* transgenic mice. The genotype of the *Mettl3-*HOE mice was *STOP-Mettl3*^*+/−*^ *Alb-Cre*^*+/−*^, whereas the genotype of the control mice was *STOP-Mettl3*^*+/−*^ (Supplementary Fig. 3b). As expected, Flag-METTL3 levels were dramatically increased specifically in the livers but not in other tissues (WAT, Skeletal muscle, BAT, Heart, Spleen, Kidney, and Brain) of *Mettl3-*HOE mice (Fig. 7b). When the mice were fed normal chow, the serum TAG levels, liver weights, and liver TAG levels were comparable between *Mettl3-*HOE and their control littermates (Supplementary Fig. 3c–e). We then challenged these mice with an MCD for 2 weeks to determine whether *Mettl3-*HOE mice are resistant to MCD-induced NASH. Interestingly, *Mettl3-*HOE mice displayed resistance to MCD-induced NASH, as revealed by lower serum ALT activity (Fig. 7c), fewer hepatic lipid droplets (Fig. 7d), and lower liver TAG levels (Fig. 7e). *Mettl3-*HOE mice also showed a significant decrease in TUNEL-positive cells (Fig. 7f), which indicates that the hepatocyte-specific overexpression of *Mettl3* could ameliorate MCD-induced liver injury. Moreover, liver sections from *Mettl3-*HOE mice contained fewer Sirius Red-positive areas than their control littermates (Fig. 7g). Consistently, the expression of fibrosis markers (*collagen IA1* and *Mmp9*) and profibrogenic factor (*Tgfb1*) was significantly decreased in *Mettl3-*HOE mice (Fig. 7h). These data indicate that hepatic overexpression of *Mettl3* ameliorates MCD-induced NASH and liver fibrosis.

We then tested whether the protective effects of hepatic METTL3 overexpression against MCD-induced NASH are due to the reduction in CD36-mediated free fatty acid uptake and CCL2-associated liver inflammation. Free fatty acid uptake and the expression of *Cd36* and *Ccl2* were measured in *Mettl3-*HOE and control mice. *Mettl3-*HOE mice showed a significant reduction in hepatic free fatty acid uptake (Fig. 7i), which was due to a decrease in CD36 expression (Fig. 7j, l). We did not observe significant differences in the expression of genes related to TAG synthesis, fatty acid β-oxidation, or VLDL secretion in *Mettl3-*HOE mice (Supplementary Fig. 3f), which suggests that hepatic overexpression of *Mettl3* ameliorates MCD-induced liver steatosis primarily by decreasing CD36-mediated free fatty acid uptake into the liver. In addition, CCL2 expression was significantly decreased in *Mettl3-*HOE mice (Fig. 7k, l). These data suggest that reductions in both CD36-mediated hepatic free fatty acid uptake and CCL2-associated liver inflammation ameliorate MCD-induced NASH in *Mettl3-*HOE mice.

### METTL3 regulates *Cd36* and *Ccl2* transcription

We next sought to investigate the mechanism by which METTL3 affects the expression of *Cd36* and *Ccl2*. METTL3-mediated m^6^A modification has been shown to regulate mRNA processing. To determine whether METTL3-mediated m^6^A modification regulates the expression of *Cd36* and *Ccl2*, m^6^A RNA immunoprecipitation sequencing (m^6^ARIP-seq) analysis was performed in the livers of *Mettl3*-HKO and *Mettl3*^flox/flox^ mice. Consistent with published m^6^ARIP-seq results, the m^6^A peaks identified in the livers of *Mettl3*^flox/flox^ mice were enriched at the stop codon and 3′-UTR and were characterized by the presence of the canonical GGACU motif (Supplementary Fig. 4a, b). However, the m^6^A peaks in the livers of *Mettl3*-HKO mice were dispersed at the transcription start site (TSS), 5′-UTR, and stop codon, and no enriched motif was found (Supplementary Fig. 4a). In the livers of *Mettl3*^flox/flox^ mice, we identified 6556 genes with m^6^A peaks (Supplementary Table 4), and 3489 genes exhibited decreased m^6^A levels in the livers of *Mettl3*-HKO mice (Supplementary Fig. 4c). GO analysis showed that the genes with downregulated m^6^A peaks were related to metabolic process, RNA metabolic process, and cellular macromolecule biosynthetic process (Supplementary Fig. 5). KEGG pathway analysis showed that the genes with downregulated m^6^A peaks were associated with ribosome, metabolic pathways, HIF-1-signaling pathway, pathways in cancer, Epstein-Barr virus infection, Alzheimer’s disease, ubiquitin-mediated proteolysis, glycolysis/gluconeogenesis, protein processing in the endoplasmic reticulum, prolactin signaling pathway, and NAFLD (Supplementary Fig. 6). Among genes related to NAFLD, both m^6^A peaks and mRNA levels of *Srebp1* (also named *Srebf1*) were significantly decreased (Supplementary Fig. 6, Supplementary Table 4 and Fig. 3a), which further indicates that lipogenesis likely contributes little to fatty liver observed in *Mettl3*-HKO mice. However, we did not detect significant m^6^A peaks in either *Cd36* or *Ccl2* transcript in the livers of *Mettl3*^flox/flox^ and *Mettl3*-HKO mice, which indicates that METTL3 regulates the expression of *Cd36* or *Ccl2*, but that it is unlikely due to an m^6^A modification in their transcripts.

**Fig. 8:**
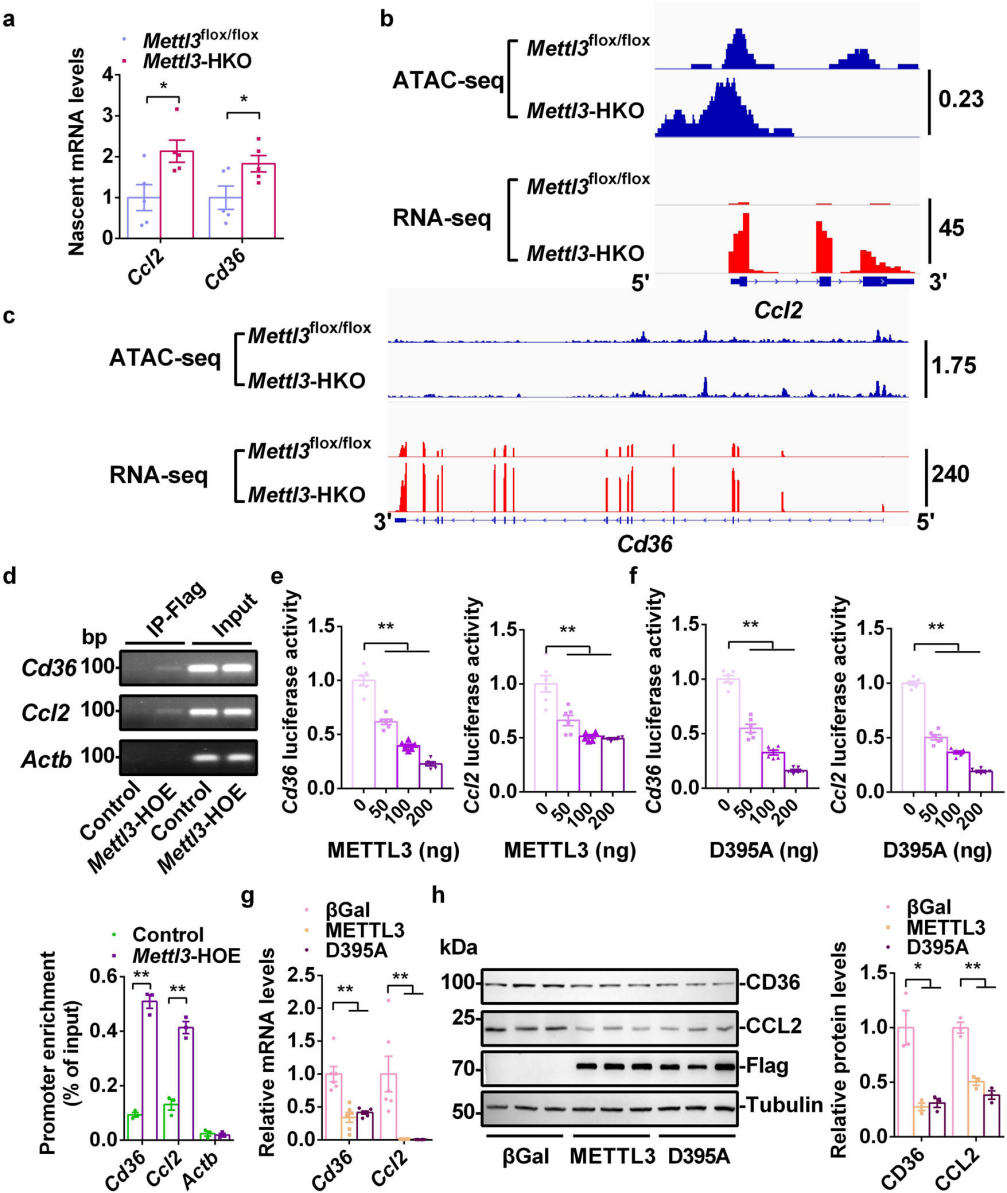
METTL3 regulates *Cd36* and *Ccl2* transcription. **a** Nascent *Cd36* and *Ccl2* mRNA levels in primary hepatocytes isolated from *Mettl3*^flox/flox^ and *Mettl3-*HKO mice (*n* = 5 for each group; *Cd36*, *P* = 0.0261; *Ccl2*, *P* = 0.0439). **b**, **c** ATAC-seq peaks in the promoters of *Cd36* and *Ccl2* genes and RNA-seq peaks of *Cd36* and *Ccl2* transcripts in the livers of *Mettl3*^flox/flox^ and *Mettl3-*HKO at 8 weeks old. **d** Binding of Flag-METTL3 to the promoters of *Cd36* and *Ccl2* were assessed in the livers of control and *Mettl3*-HOE mice at 8 weeks old by ChIP. The liver samples were immunoprecipitated with Flag beads. Immunoprecipitated DNA was extracted for PCR analysis. Representative image and statistic analysis were shown (*n* = 3 for each group; *Cd36*, *P* < 0.0001; *Ccl2*, *P* = 0.0007; *Actb*, *P* = 0.666). The samples were derived from the same experiment and the gels were processed in parallel. **e**, **f** *Cd36* or *Ccl2* luciferase reporter plasmids were co-transfected with METTL3, D395A, or empty expression vector by polyethyleneimine (Sigma) into HEK293T cells. 48 h later, HEK293T cells were lysed in reporter lysis buffer, and luciferase activity was measured and normalized to β-Gal activity (*n* = 6 for each group; *Cd36*, *P* < 0.0001; *Ccl2*, *P* < 0.0001). **g**, **h** Primary hepatocytes were infected with Ad-βGal, Ad-METTL3 and Ad-METTL3 (D395A) adenovirus overnight, *Cd36* and *Ccl2* mRNA levels were measured by qPCR (*n* = 6 for each group; for *Cd36*, βGal versus METTL3, *P* = 0.0008, METTL3 versus D395A, *P* = 0.0006; for *Ccl2*, βGal versus METTL3, *P* = 0.0044, METTL3 versus D395A, *P* = 0.0042). CD36 and CCL2 protein levels were measured by immunoblotting, quantified by ImageJ and normalized to Tubulin (*n* = 3 for each group; for CD36, βGal versus METTL3, *P* = 0.0111, METTL3 versus D395A, *P* = 0.0134; for CCL2, βGal versus METTL3, *P* = 0.0012, METTL3 versus D395A, *P* = 0.0006). The samples were derived from the same experiment and the blots were processed in parallel. *n* was the number of biologically independent mice or cell samples. The cell culture experiments were repeated three times independently with similar results. Data represent the mean ± SEM. Significance was determined by unpaired two-tailed Student’s *t* test analysis. *P < 0.05. **P < 0.01. Source data are provided as a Source Data file.

Nuclear run-on RT-qPCR showed that hepatic deletion of *Mettl3* significantly increased *Cd36* and *Ccl2* nascent mRNA levels (Fig. 8a). These data indicate that METTL3 regulates *Cd36* and *Ccl2* expression at the transcriptional level independent of m^6^A modifications. To further test this hypothesis, we mapped open chromatin in the livers of *Mettl3-*HKO mice using Assay for Transposase-Accessible Chromatin with high-throughput sequencing (ATAC-seq). As expected, we observed strong enrichment of open chromatin in the gene promoters and the ATAC-seq peaks near the TSS (Supplementary Fig. 7), which are associated with the activation of gene transcription. ATAC-seq peaks were significantly increased in the promoters of *Cd36* and *Ccl2* in the livers of *Mettl3-*HKO mice (Fig. 8b, c), which indicates that deletion of *Mettl3* promotes *Cd36* and *Ccl2* transcription. RNA-seq data also showed that both *Cd36* and *Ccl2* transcripts were dramatically increased in the livers of *Mettl3-*HKO mice (Fig. 8b, c). Furthermore, METTL3 directly bound to the *Cd36* and *Ccl2* promoters but did not bind to the *Actb* promoter in the livers of *Mettl3-*HOE mice, as detected by chromatin immunoprecipitation (ChIP) (Fig. 8d). In addition, promoter luciferase assays showed that METTL3 decreased both *Cd36* and *Ccl2* promoter-luciferase activities in a dose-dependent manner (Fig. 8e). The METTL3 enzyme-dead mutation (D395A) also reduced both *Cd36* and *Ccl2* promoter-luciferase activities in a dose-dependent manner (Fig. 8f). Consistently, both METTL3 and its mutation D395A significantly decreased the mRNA and protein levels of CD36 and CCL2 (Fig. 8g, h). METTL3 mutation (D395A) also dramatically decreased the expression of *Cd36* and *Ccl2* in isolated hepatocytes from *Mettl3-*HKO mice (Supplementary Fig. 8). These data demonstrate that METTL3 serves as a transcriptional repressor of both the *Cd36* and *Ccl2* genes independent of its methyltransferase enzyme activity.

### METTL3 interacts with HDAC1/2 and regulates H3K9ac and H3K27ac levels in the promoters of *Cd36* and *Ccl2*

**Fig. 9:**
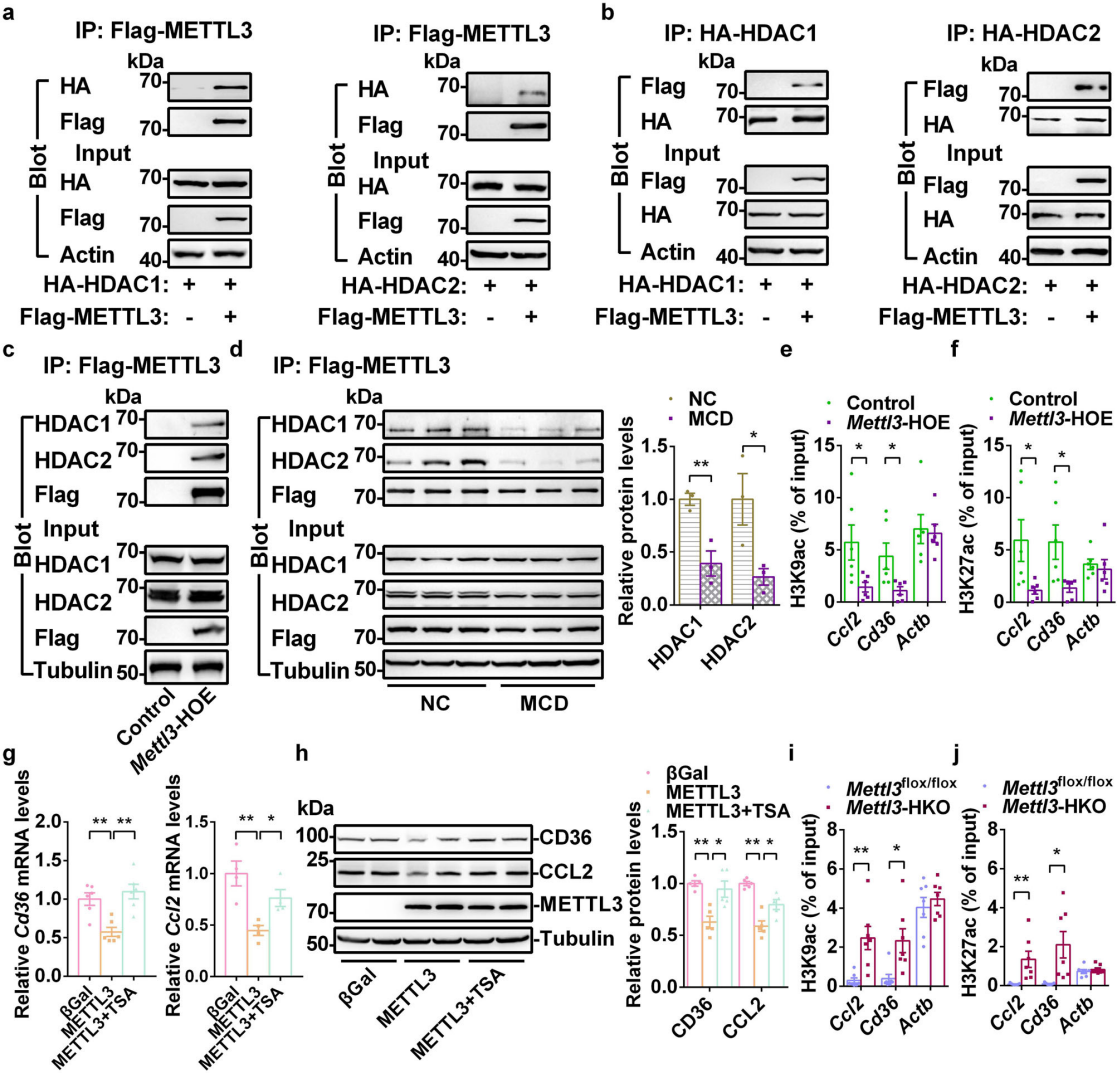
METTL3 interacts with HDAC1/2 and regulates H3K9ac and H3K27ac levels in the promoters of *Cd36* and *Ccl2* genes. **a**, **b** HA-HDAC1/2 expression vector was co-transfected with or without Flag-METTL3 expression vector in HEK293T cells. Total cell lysates were first incubated with DNase1 (200 U/ml) at 37 °C for 30 min. These lysates were immunoprecipitated with Flag or HA beads and then immunoblotted with anti-HA or anti-Flag antibodies. The samples were derived from the same experiment and the blots were processed in parallel. **c** Liver extracts from control and *Mettl3*-HOE mice at 8 weeks old were first incubated with DNase1 (200 U/ml) at 37 °C for 30 min. These lysates were immunoprecipitated with Flag beads and then immunoblotted with anti-HDAC1 or anti-HDAC2 antibodies. The samples were derived from the same experiment and the blots were processed in parallel. **d** *Mettl3*-HOE mice were fed an NC or MCD for 2 weeks. Liver extracts were first incubated with DNase1 (200 U/ml) at 37 °C for 30 min. These lysates were immunoprecipitated with Flag beads and then immunoblotted with anti-HDAC1 or anti-HDAC2 antibodies. The HDAC1/2 protein levels associated with Flag-METTL3 were quantified by ImageJ and normalized to input HDAC1/2 (*n* = 3 for each group; For HDAC1, NC versus MCD, *P* = 0.0096; For HDAC2, NC VS MCD, *P* = 0.0462). The samples were derived from the same experiment and the blots were processed in parallel. **e**, **f** H3K9ac and H3K27ac levels in the promoters of *Ccl2*, *Cd36* and *Actb* genes in *Mettl3-*HOE and control mice fed an MCD for 2 weeks (*n* = 6 for each group; For H3K9ac, *Ccl2*, *P* = 0.0334, *Cd36*, *P* = 0.03, *Actb*, *P* = 0.8045; For H3K27ac, *Ccl2*, *P* = 0.0401, *Cd36*, *P* = 0.02585, *Actb*, *P* = 0.6527). **g**, **h** Primary hepatocytes were infected with Ad-βGal and Ad-METTL3 adenovirus. Ad-METTL3-infected hepatocytes were treated with or without TSA (2 μM) overnight. The relative *Cd36* and *Ccl2* mRNA levels were measured by qPCR (For *Cd36*, *n* = 6 for each group, βGal versus METTL3, *P* = 0.0016, METTL3 versus METTL3+TSA, *P* = 0.0009; For *Ccl2*, *n* = 4 for each group, βGal versus METTL3, *P* = 0.0054, METTL3 versus METTL3+TSA, *P* = 0.0167). CD36 and CCL2 protein levels were measured by immunoblotting, quantified by ImageJ and normalized to Tubulin (n = 5 for each group; For CD36, βGal versus METTL3, *P* = 0.0004, METTL3 versus METTL3+TSA, *P* = 0.0112; For CCL2, βGal versus METTL3, *P* < 0.0001, METTL3 versus METTL3+TSA, *P* = 0.0167). The samples were derived from the same experiment and the blots were processed in parallel. **i**, **j** H3K9ac and H3K27ac levels in the promoters of *Ccl2*, *Cd36* and *Actb* genes in the livers of *Mettl3-*HKO and *Mettl3*^flox/flox^ mice at 8 weeks old (*n* = 7 for each group; for H3K9ac, *Ccl2*, *P* = 0.0037, *Cd36*, *P* = 0.0113, *Actb*, *P* = 0.4987; For H3K27ac, *Ccl2*, *P* = 0.0097, *Cd36*, *P* = 0.0126, *Actb*, *P* = 0.4736). *n* was the number of biologically independent mice or cell samples. The cell culture experiments were repeated three times independently with similar results. Data represent the mean ± SEM. Significance was determined by unpaired two-tailed Student’s *t* test analysis. *P < 0.05. **P < 0.01. Source data are provided as a Source Data file.

We next investigated how METTL3 functions as a transcriptional repressor. Protein interaction data in the neXtProt human protein knowledgebase and IntAct database showed that METTL3 might interact with HDAC1 and HDAC2^44,45^. HDAC1 and HDAC2 are known to repress the expression of target genes by deacetylating histone H3K9 and H3K27. We found that METTL3 coimmunoprecipitated with either HDAC1 or HDAC2 in both HEK293T cells (Fig. 9a, b) and livers from *Mettl3-*HOE mice (Fig. 9c). These interactions were decreased in MCD-induced NASH (Fig. 9d). Immunofluorescence data showed that Flag-METTL3 was translocated from the nucleus to the cytosol in MCD-induced NASH (Supplementary Fig. 9). These data indicate that the decreased interaction between METTL3 and HDAC1/2 in NASH was likely due to the cytosolic translocation of METTL3. To determine whether METTL3 regulates the acetylation of histone H3K9 and H3K27 in the promoters of *Cd36* and *Ccl2*, the acetylation of H3K9 and H3K27 in *Mettl3-*HOE, *Mettl3-*HKO, and corresponding control mice was measured by ChIP. As shown in Fig. 9e, f, both H3K9ac and H3K27ac levels in the promoters of *Cd36* and *Ccl2* were significantly decreased in the livers of *Mettl3-*HOE mice, which indicates that METTL3 was able to elicit deacetylation of H3K9 and H3K27 in the promoters of *Cd36* and *Ccl2*. To confirm that the inhibition of *Cd36* and *Ccl2* expression is mediated by METTL3-elicited histone deacetylation, primary hepatocytes were infected with Ad-METTL3 and treated with or without trichostatin A (TSA), an HDACs inhibitor. As shown in Fig. 9g, h, TSA reversed the suppression of *Cd36* and *Ccl2* expression mediated by METTL3 overexpression. To further test this hypothesis, we used a more specific HDAC1/2 inhibitor (Romidepsin) to repeat this experiment. As shown in Supplementary Fig. 10a–b, Romidepsin completely reversed the suppression of *Cd36* expression and partially rescued the inhibition of *Ccl2* expression mediated by METTL3 overexpression. Furthermore, we measured the acetylation of H3K9 and H3K27 in the promoters of *Cd36* and *Ccl2* in the livers of *Mettl3-*HKO and *Mettl3*^flox/flox^ mice. As shown in Fig. 9i, j, hepatic deletion of *Mettl3* markedly increased both H3K9ac and H3K27ac levels in the *Cd36* and *Ccl2* promoters in the liver. To further determine whether METTL3 regulates HDAC1/2 activity, we measured the activities of HDAC1/2 in the livers of *Mettl3*-HKO and *Mettl3*^flox/flox^ mice. As shown in Supplementary Fig. 11, the activities of HDAC1/2 were significantly decreased in *Mettl3*-HKO mice. These data demonstrate that METTL3 regulates *Cd36* and *Ccl2* transcription by modulating H3K9ac and H3K27ac in their promoters by the involvement of HDAC1/2.

## Discussion

NASH is a major worldwide health problem and is characterized by hepatic steatosis, liver injury, and chronic inflammation. NASH is a key step whereby patients with simple NAFL develop cirrhosis and HCC^3,5^. However, only 25% of patients with NAFL develop NASH^3^. Thus, it is crucial to identify the positive and negative regulators that determine whether patients with simple NAFL develop NASH. In the present study, METTL3 was identified as a previously unrecognized suppressor of the NAFL-to-NASH transition. Hepatic deletion of *Mettl3* promotes NASH progression under either HFD or MCD-feeding conditions due to increased hepatic free fatty acid uptake and inflammation, which are strongly associated with increased expression of *Cd36* and *Ccl2*, respectively. Hepatic overexpression of *Mettl3* protects against MCD-induced NASH by suppressing *Cd36* and *Ccl2* expression. Mechanistically, METTL3 directly binds to the promoters of *Cd36* and *Ccl2* genes and recruits HDAC1/2, which causes deacetylation of H3K9 and H3K27, thus suppressing *Cd36* and *Ccl2* transcription.

The loss of *Mettl3* in the liver leads to more-pronounced steatosis, steatohepatitis, and collagen deposition, which suggests a robust fibrosis progression, whereas overexpression of *Mettl3* in the liver induces the opposite effects. In agreement with these results, we showed that hepatic deletion of *Mettl3* increases free fatty-acid uptake through increased *Cd36* expression, whereas hepatic *Mettl3* overexpression induces the opposite effects. It has been shown that abnormally increased expression of CD36 contributes to the development of steatosis and NASH^12^, whereas knockout of *Cd36* has been shown to protect against MCD-induced NASH^13^. However, factors related to lipogenesis, fatty acid oxidation, and VLDL secretion were not changed by overexpression of *Mettl3*. Gene expression data suggest that fatty acid β-oxidation, lipogenesis, and VLDL secretion do not contribute to the increased liver steatosis seen in *Mettl3*-HKO mice. Thus, the loss of *Mettl3* in the liver promotes hepatic steatosis mainly by increasing *Cd36* expression.

Liver steatosis is considered a first but insufficient hit to induce NASH^5^. This is why an HFD alone cannot induce NASH. Surprisingly, an HFD is sufficient to induce NASH in *Mettl3-*HKO mice. Liver inflammation has been shown to be a key secondary hit for the NAFL-to-NASH transition. We confirmed the expression of cytokines and chemokines and found that CCL2 was strongly upregulated in *Mettl3*-HKO livers and hepatocytes. CCL2 and its receptor CCR2 are strongly associated with NASH^14,15^, and inhibition of CCL2 and CCR2 has been shown to be a therapeutic target for the treatment of NASH^16,17^. Hepatic overexpression of *Mettl3* ameliorates MCD-induced NASH most likely by suppressing CCL2-induced inflammation and CD36-mediated free fatty acid uptake. Blockade of CD36 and CCL2 with neutralizing antibodies ameliorates the NASH progression in *Mettl3-*HKO mice, which further supports the theory that elevated CD36 and CCL2 contribute to the NASH progression in *Mettl3-*HKO mice.

Our focus was on the question of how METTL3 regulates *Cd36* and *Ccl2* expression. METTL3 is considered a key RNA methyltransferase that catalyzes RNA m^6^A modification and regulates most of the RNA processing steps^18^. m^6^ARIP-seq analysis identified 6,556 genes with m^6^A peaks in the livers of *Mettl3*^flox/flox^ mice, and 3,489 genes showed decreased m^6^A levels in the livers of *Mettl3*-HKO mice, which indicates the importance of METTL3-mediated m^6^A modification in liver function. However, no significant m^6^A peaks were detected in either the *Cd36* or the *Ccl2* transcript in the livers of *Mettl3*^flox/flox^ and *Mettl3*-HKO mice, which indicates that METTL3 regulates their expression through some means other than mRNA m^6^A modification. Instead, we observed that METTL3 binds directly to the promoters of *Cd36* and *Ccl2* and represses their promoter luciferase activities. METTL3 also interacts with HDAC1/2 in the liver. This interaction is likely disrupted during NASH progression. Hepatic deletion of *Mettl3* decreases the activities of HDAC1/2, indicating that METTL3 regulates HDAC1/2 activity. Hepatic overexpression of *Mettl3* decreases the levels of H3K9ac and H3K27ac, which are active epigenetic marks, in the promoters of *Cd36* and *Ccl2*, leading to decreased transcription of *Cd36* and *Ccl2*. Conversely, hepatic ablation of *Mettl3* increases H3K9ac and H3K27ac levels in the promoters of *Cd36* and *Ccl2*, which likely results in elevated chromatin accessibility and increased transcription of these genes. Furthermore, inhibition of HDAC1/2 by either TSA or Romidepsin blocks the suppression of *Cd36* and *Ccl2* transcription mediated by METTL3 overexpression. These data support a mechanism by which METTL3 suppresses free fatty acid uptake and inflammation at least in part by recruiting HDAC1/2 to the promoters of *Cd36* and *Ccl2* genes, where HDAC1/2 catalyzes the repressive deacetylation of H3K9 and H3K27. HDAC1/2 is not the only factor regulating H3K9ac/H3K27ac. Other HDACs and histone acetyltransferase (p300/CBP) may also contribute to the regulation of H3K9ac/H3K27ac. We cannot rule out the possibility that other HDACs and CBP/EP300 might be involved in this process, as *Mettl14* deletion increases CBP/EP300 expression by increasing RNA stability due to decreased m^6^A modification^29^.

Several pieces of evidence support the conclusion that METTL3 can regulate the transcription of *Cd36* and *Ccl2* in the liver independent of its methyltransferase enzyme activity. Both METTL3 and METTL3 enzyme-dead mutation (D395A) reduces both *Cd36* and *Ccl2* promoter-luciferase activities in a dose-dependent manner. Consistently, both METTL3 and its mutation D395A significantly decrease the mRNA and protein levels of CD36 and CCL2. METTL3 mutation (D395A) also dramatically decreases the expression of *Cd36* and *Ccl2* in isolated hepatocytes from *Mettl3-*HKO mice. These data demonstrate that METTL3 serves as a transcriptional repressor of both the *Cd36* and *Ccl2* genes independent of its methyltransferase enzyme activity. Although METTL3-mediated m^6^A modification may not be involved in the regulation of *Cd36* and *Ccl2* expression in the pathogenesis of NASH, METTL3-mediated m^6^A modification may regulate the expression of other genes and other liver processes^33^, which requires further study. METTL14 and WTAP both interact with METTL3^18–20^. Whether METTL14 and WTAP also negatively regulate NASH progression by inhibiting the expression of *Cd36* and *Ccl2* also needs future investigation.

Two recent studies show that METTL3 in mouse embryonic stem cells regulates chromatin accessibility depending on its methyltransferase enzyme activity^47,48^. One of the studies shows that *Mettl3* knockout in mouse embryonic stem cells increases chromatin accessibility and activates transcription by increasing expression of the chromosome-associated regulatory RNAs, especially LINE1 elements, due to decreased m^6^A modification^47^. We checked the mRNA and m^6^A levels of LINE1 in the livers of *Mettl3*-HKO and *Mettl3*^flox/flox^ mice from our RNA-seq and m^6^ARIP-seq data. RNA-seq data show that the fpkm value of LINE1(ENSMUSG00000087166) is o (zero) in the livers of *Mettl3*-HKO and *Mettl3*^flox/flox^ mice, which indicates that LINE1 is not expressed in the liver, and unlikely regulates the chromatin state in the livers of *Mettl3*-HKO mice. These data also indicate that METTL3 can regulate chromatin accessibility through different mechanisms in a context-dependent and cell-type-specific manner.

Nuclear METTL3 protein levels are decreased in both human patients and mice with NASH, whereas nuclear METTL3 protein levels are increased in NAFL, which indicates that decreased nuclear METTL3 may contribute to the NAFL-to-NASH transition. Decreased nuclear METTL3 reduces HDAC1/2 activity in the promoters of *Cd36* and *Ccl2*, which results in higher levels of H3K9ac and H3K27ac in their promoters and further increases their transcription. This has been observed in *Mettl3-*HKO mice. With respect to the nuclear/cytosolic translocation of METTL3 in NASH, TNFα/CDK9-mediated serine phosphorylation of METTL3 partially blocks the nuclear localization of METTL3, while inhibition of CDK9 increases the nuclear localization of METTL3. Although phosphorylation of some sites on METTL3 does not lead to nuclear/cytosolic translocation^38^, phosphorylation of other sites on METTL3 mediated by CDK9 may block its nuclear localization. We cannot rule out the possibility that other cytokines and kinases might also contribute to the cytosolic accumulation of METTL3 in NASH, as TNFa did not fully block its nuclear localization and a CDK9 inhibitor only partially blocked TNFa-induced cytosolic accumulation of METTL3.

In conclusion, we have demonstrated that METTL3 is an important repressor of NASH progression. Our data also reveal a mechanism by which METTL3 suppresses the transcription of *Cd36* and *Ccl2* via a histone modification pathway that includes the involvement of HDAC1/2. Decreased nuclear METTL3 may lead to impairment of the METTL3/HDAC1/2 axis, which further increases hepatic free fatty acid uptake and liver inflammation, contributing to NASH progression. Our results also indicate that the METTL3/HDAC1/2 axis may serve as a drug target for the treatment of NASH.

## Methods

### Animal experiments

Animal experiments were carried out in strict accordance with the Guide for the Care and Use of Laboratory Animals (8th edition). Animal experiment protocols were approved by the Institutional Animal Care and Use Committee of Harbin Institute of Technology (HIT/IACUC). The approval number was IACUC-2018004. Mice were housed on a 12-h light/12-h dark cycle, temperature (24 ± 2 °C) and humidity (50% ± 10%) conditions. Mice were fed a normal chow diet with free access to water. For diet-induced obesity, mice were fed an HFD (MD12032, 45% fat, Medicience) for 12 weeks. For diet-induced NASH, mice were fed an MCD (MD12052, Medicience) for 2–3 weeks. *Mettl3*^flox/flox^ mice, in which exon 2 and exon 3 of the *Mettl3* gene were flanked by two loxp sites, were generated using the CRISPR-Cas9 technique as previously reported^27^. *STOP-Mettl3* mice were generated using the CRISPR-Cas9 technique to insert a *STOP-Flag-Mettl3* cassette into the *Rosa26* allele with the help of Shanghai Biomodel Organism. Hepatocyte-specific *Mettl3* knockout (*Mettl3-*HKO) mice were generated by crossing *Mettl3*^flox/flox^ mice with *Alb*-*Cre* mice. Hepatocyte-specific *Mettl3*-overexpressing mice were generated by crossing STOP-*Mettl3*^+/−^ mice with *Alb*-*Cre* mice. For inhibition of CD36 and CCL2 in vivo, 8-week-old *Mettl3-*HKO mice were treated with a combination of anti-CD36 (ab23680, Abcam) and anti-CCL2 (MAB479500, R&D Systems) antibodies (4 μg/antibody/mouse, i.v.) or control antibodies (IgA, bs-0774P, Bioss, and IgG2b, 65211-1-Ig, Proteintech) on days 1, 4, 7, and 8 after MCD feeding. Mice were sacrificed on day 9 after MCD feeding. Blood samples were collected from the orbital sinus. The serum alanine aminotransferase (ALT) activities and TAG levels were measured with an ALT or TAG reagent set, respectively^49^.

### Human liver samples

Human liver samples were collected in the Third Affiliated Hospital of Sun Yat-sen University. The present study was approved by the Research Ethics Committee of the Third Affiliated Hospital of Sun Yat-sen University, and individual permission was obtained using standard informed consent procedures. The investigation conformed to the principles outlined in the Declaration of Helsinki regarding the use of human tissues. Detailed characteristics of patients with or without NASH are listed in Supplementary Table 1.

### Free fatty-acid uptake assays

*Mettl3*^flox/flox^ and *Mettl3*-HKO mice were fasted overnight and then injected intraperitoneally with 20 μM BODIPY FL C16 in 200 μl saline for 20 min. Livers were harvested and homogenized in RIPA buffer. Liver lysates were mixed with three volumes of Dole’s reagent (heptane: 2-propanol: 2N sulfuric acid; 10:40:1) and centrifuged at 18,000 × *g* for 10 min. The BODIPY FL C16 fluorescence in the top organic-phase supernatant was determined using a 488 nm excitation and 515 nm emission filter set (BioTek) and normalized to the protein concentration^50^.

For the in vitro free fatty-acid uptake assay, primary hepatocytes were isolated from *Mettl3*^flox/flox^ and *Mettl3-*HKO mice. Hepatocytes were incubated with BODIPY FL C16 (1 μM) for 30 min in phosphate-buffered saline (PBS), washed with PBS, and fixed with 4% paraformaldehyde (PFA) for 10 min at RT. BODIPY FL C16 fluorescence was observed using a fluorescent microscope (Olympus) and quantified using ImageJ version 1.39 f (National Institutes of Health).

### Primary hepatocyte culture and adenoviral infection

Primary hepatocytes were isolated from C57BL/6 WT, *Mettl3*^flox/flox^, and *Mettl3-*HKO mice by liver perfusion with type II collagenase (Worthington Biochem, Lakewood, NJ) and cultured at 37 °C and 5% CO_2_ in DMEM medium supplemented with 5% FBS. Primary hepatocytes from C57BL/6 WT mice were infected with an equal amount of βGal, Flag-METTL3, or Flag-METTL3(D395A) adenoviruses overnight.

### Nuclear run-on RT-qPCR

Nascent *Cd36* and *Ccl2* mRNA levels were measured by nuclear run-on RT-qPCR^51,52^. In brief, primary hepatocytes were isolated from *Mettl3*^flox/flox^ and *Mettl3-*HKO mice. Nuclei were extracted in NP-40 lysis buffer (10 mM Tris HCl pH 7.4, 10 mM NaCl, 3 mM MgCl2, and 0.5% NP-40), and then incubated with BrUTP and unlabeled ribonucleotides in a transcription reaction buffer supplemented with 100 U RNase OUT, 0.5 mM BrUTP, 1 mM ATP, 1 mM GTP, 1 mM CTP, and 0.5 mM UTP at 30 °C for 30 min. Nuclear RNA was extracted using the TriPure Isolation Reagent (Roche, Mannheim, Germany). These nuclear RNA samples were immunoprecipitated with anti-BrdU antibody (66241-1-Ig, Proteintech). Nascent *Cd36* and *Ccl2* mRNA levels were then measured by RT-qPCR and normalized to 36B4. Primers for real-time RT-qPCR were listed in Supplementary Table 2.

### Luciferase assays

The mouse *Cd36* promoter (from −2001 to −1) and *Ccl2* promoter (from −2001 to −1) were cloned into pGL3 vectors, respectively. Transient transfection and luciferase assays were performed^49,52^. Briefly, HEK293T cells were seeded in 24-well plates 20 h before transfection. *Cd36* or *Ccl2* luciferase reporter plasmids were co-transfected with METTL3, D395A, or empty expression vector by polyethylenimine (Sigma) into HEK293T cells. 48 h later, HEK293T cells were lysed in reporter lysis buffer (Promega, Madison, WI), and luciferase activity was measured and normalized to β-galactosidase (β-Gal) activity. The reagents were listed in Supplementary Table 3.

### Transient transfection, immunoprecipitation, and immunoblotting

HEK293T cells were seeded in 6-well plates 16 h before transfection. The indicated expression vectors (Myc-CDK9, HA-HDAC1, or HA-HDAC2: 1 μg) were co-transfected with or without the Flag-METTL3 expression vector (1 μg) in HEK293T cells. For the Co-IP of HDAC1/2 and METTL3, total cell lysates were first incubated with DNase1 (200 U/ml) at 37 °C for 30 min to digest genomic DNA. These lysates were then immunoprecipitated with Myc, Flag, or HA beads at 4 °C for 2 h. Immunoblotting was performed using the indicated antibodies. Antibody dilutions were as follows: METTL3 (96391, Cell Signaling Technology), 1:2500; Lamin B1 (12987-1-AP, Proteintech), 1:5000; Tubulin (sc-5286, Santa Cruz), 1:5000; β-actin (60008-1-Ig, Proteintech), 1:5000; CDK9 (11705-1-AP, Proteintech), 1:2500; p-CDK9 (2549, Cell Signaling Technology), 1:2500; CD36 (18836-1-AP, Proteintech), 1:5000; Flag (F1804, Sigma), 1:5000; Myc (16286-1-AP, Proteintech), 1:5000; HDAC1 (5356, Cell Signaling Technology), 1:2500; HDAC2 (5113, Cell Signaling Technology), 1:2500; and Caspase3 (9662, Cell Signaling Technology), 1:2500. Other reagents were listed in Supplementary Table 3.

### In vitro kinase assay

Myc-CDK9 was immunopurified using anti-Myc magnetic Beads (B26301, Bimake). Myc-CDK9 was incubated with purified Flag-METTL3 (1.5 μg) in kinase buffer (20 mM HEPES, pH 7.5-7.6, 33 μM ATP, 10 mM MgCl_2_, 50 mM NaCl, 1 mM PMSF and Phosphatase Inhibitor Cocktail 1) at 30 °C for 30 min. Flag-METTL3 (1.5 μg) in the kinase buffer without Myc-CDK9 was served as a control. The reactions were stopped by adding SDS-PAGE loading buffer and boiling for 5 min. Proteins were immunoblotted with antibodies against phosphoserine, Flag, and Myc, respectively. Antibody dilutions were as follows: phosphoserine (AB1603, Sigma), 1:1000; Flag (F1804, Sigma), 1:5000; and Myc (16286-1-AP, Proteintech), 1:5000.

### Real-time quantitative PCR

Total RNA was extracted using the TriPure Isolation Reagent (Roche, Mannheim, Germany), and first-strand cDNA was synthesized using random primers and M-MLV reverse transcriptase (Promega, Madison, WI)^53^. RT-qPCR was performed using a Roche LightCycler 480 real-time PCR system (Roche, Mannheim, Germany)^54^. The data were analyzed using LightCycler 480 Software (v1.5.1). The expression of individual genes was normalized to the expression of 36B4. Primers for real-time RT-qPCR were listed in Supplementary Table 2.

### Chromatin immunoprecipitation assays

The livers were fixed by liver perfusion with 1% PFA. The nuclei were isolated from livers and subjected to sonication (M220, Focused-ultrasonicator; Covaris) to break genomic DNA into 500- to 1000-bp fragments using a chromatin shearing kit (520127, truChIP Chromatin Shearing Kit, Covaris). The samples were immunoprecipitated with anti-FLAG M2 magnetic beads (M8823, Millipore) or antibodies against H3K9ac or H3K27ac. DNA was extracted and used for qPCR analysis. Primers for qPCR were listed in Supplementary Table 2.

### Nuclear extract preparation

Liver tissues were homogenized in lysis buffer (20 mM HEPES, 1 mM EDTA, 250 mM sucrose, 1 mM PMSF, 1 mM Na_3_VO_3_ and 0.5 mM DTT, pH 7.4) and centrifuged sequentially at 1100 × *g* and 4000 × *g* at 4 °C. Nuclear protein was extracted from the pellets using a high-salt solution (20 mM HEPES, 420 mM NaCl, 0.2 mM EDTA, 0.5 mM DTT, 1 mM PMSF and 1 mM Na_3_VO_3_, pH 7.9). The preparation of nuclear and cytosolic proteins from primary hepatocytes was performed using a commercial Nuclear and Cytoplasmic Protein Extraction Kit (P0027, Beyotime).

### RNA-sequencing and m^6^ARIP-sequencing

For RNA-sequencing, total RNA was extracted using Tripure Isolation Reagent (94015120, Roche, Mannheim, Germany) from livers of *Mettl3*^flox/flox^ and *Mettl3-*HKO mice at 8 weeks old. Each sample was pooled from four mice for each group. A total amount of 1 μg RNA per sample was used for the RNA sample preparations. Sequencing libraries were generated using NEBNext® Ultra™ RNA Library Prep Kit for Illumina® (NEB, USA) following the manufacturer’s recommendations and index codes were added to attribute sequences to each sample. The clustering of the index-coded samples was performed on a cBot Cluster Generation System using TruSeq PE Cluster Kit v3-cBot-HS (Illumia) according to the manufacturer’s instructions. After cluster generation, the library preparations were sequenced on an Illumina Hiseq X Ten platform and 125 bp/150 bp paired-end reads were generated. Paired-end clean reads were aligned to the mouse reference genome (Ensemble_GRCm38.90) with Hisat2 (version 2.0.4), and the aligned reads were used to quantify mRNA expression by using HTSeq (version 0.9.1).

For m^6^ARIP-sequencing, total RNA was extracted using Tripure Isolation Reagent (94015120, Roche, Mannheim, Germany) from livers of *Mettl3*^flox/flox^ and *Mettl3-*HKO mice at 8 weeks old. Each sample (300 μg total RNA) was pooled from three mice for each group. Poly(A)+RNA was purified using Dynabeads™ mRNA Purification Kit (61006, Invitrogen). Fragmented poly(A)+RNA was incubated with m^6^A antibody (202003, Synaptic System) for immunoprecipitation using Magna MeRIP^TM^ m^6^A Kit (17-10499, MERCK). Then, immunoprecipitated mRNA or Input was used for library construction with NEBNext ultra RNA library prep kit for Illumina (New England Biolabs). The library preparations were sequenced on an Illumina Hiseq X platform. Sequenced reads were then mapped to Ensemble_GRCm38.90 whole genome using BWA (V0.7.12).

After mapping reads to the reference genome, exomePeak R package (v2.16.0) was used for the m^6^A peak identification in each anti-m^6^A immunoprecipitation group with the corresponding input samples serving as a control, and *q* value threshold of enrichment of 0.05 was used for all data sets. The m^6^A-enriched motifs of each group were identified by HOMER (v4.9.1)^55^. Peak-related genes were confirmed by PeakAnnotator (v7.4), and then GO enrichment analysis of performed to identify the function enrichment results. GO enrichment analysis was implemented by the GOseq R package (v1.26.0), in which gene length bias was corrected. GO terms with corrected *P* value < 0.05 were considered significantly enriched by peak-related genes.

### ATAC-sequencing

ATAC-seq was performed using a standard protocol with some modifications^56,57^. Liver samples were pooled from the livers of *Mettl3*-HKO and *Mettl3*^flox/flox^ mice (*n* = 4 for each group). Nuclei were extracted from liver samples, and the nuclei pellets were resuspended in the Tn5 transposase reaction mix. The transposition reaction was incubated at 37 °C for 30 min. Equimolar adapter1 and adapter2 were added after transposition, and PCR was then performed to amplify the library. After PCR, libraries were purified with the AMPure beads, and library quality was assessed with Qubit. Clustering of the index-coded samples was performed on a cBot Cluster Generation System using TruSeq PE Cluster Kit v3-cBot-HS (Illumina). After cluster generation, the library preparations were sequenced on an Illumina NovaSeq 6000 platform and 150 bp paired-end reads were generated. Nextera adaptor sequences were firstly trimmed from the reads using skewer (V0.2.2). These reads were aligned to a reference genome using BWA (V0.7.12), with standard parameters. These reads were then filtered for high quality (MAPQ 13), non-mitochondrial chromosome, and properly paired reads (longer than 18 nt). All peak calling was performed with macs2 using “macs2 callpeak —nomodel —keepdup all —call-summits”. For simulations of peaks called per input read, aligned and de-duplicated BAM files were used without any additional filtering.

### HDAC1 and HDAC2 activity assays

Liver tissues from *Mettl3*^flox/flox^ and *Mettl3-*HKO mice were homogenized in an L-RIPA lysis buffer (50 mM Tris, pH 7.5, 1% Nonidet P-40, 150 mM NaCl, 2 mM EGTA, 1 mM Na_3_VO_4_, 100 mM NaF, 10 mMNa_4_P_2_O_7_, 1 mM phenylmethylsulfonyl fluoride). HDAC1 or HDAC2 was immunoprecipitated from 200 μg of total lysate with 0.9 μg HDAC1 (10197-1-AP, Proteintech) or HDAC2 (12922-3-AP, Proteintech) antibody. As a negative control, we used 0.9 μg IgG antibody (2729, Cell Signaling Technology) to detect non-specific-bound HDAC activity. Immunoprecipitates were then assayed for HDAC activity using an Epigenase HDAC Activity/Inhibition Direct Assay Kit (Epigentek) according to the manufacturer’s protocol. HDAC1/2 activity values were corrected for non-specific (IgG)-bound HDAC activity and divided by the average HDAC1/2 activity value in *Mettl3*^flox/flox^ mouse livers to determine the relative HDAC1/2 activity.

### Statistical analysis

Data were analyzed using GraphPad Prism 6.02. Data were presented as means ± SEM. Differences between groups were analyzed by unpaired two-tailed Student’s *t* tests. *P* < 0.05 was considered statistically significant. *P < 0.05. **P < 0.01.

### Reporting summary

Further information on research design is available in the Nature Research Reporting Summary linked to this article.

## Supplementary information

Supplementary information

Reporting Summary

## Data Availability

The ATAC-seq and RNA-seq data generated in this study have been deposited in the GEO database under accession code GSE141325. The m^6^ARIP-seq data generated in this study have been deposited in the GEO database under accession code GSE142835. The m^6^ARIP-seq data generated in this study are provided in the Supplementary Information/Source Data file. The published RNA-seq data used in this study are available in the GEO database under accession codes GSE43314 and GSE119340^35–37^. All other data generated or analyzed during this study are included in this published article (and its supplementary information files). Source data are provided with this paper.

## Source data

Source Data
