# Supplementary material for: The methyltransferase METTL3 negatively regulates nonalcoholic steatohepatitis (NASH) progression
Source: Nat Commun. 2021 Dec 10;12:7213. doi: 10.1038/s41467-021-27539-3 (PMC8664922; doi:10.1038/s41467-021-27539-3)
Supplement: Supplementary file 1 — Supplementary information [file 41467_2021_27539_MOESM1_ESM.pdf]

The methyltransferase METTL3 negatively regulates nonalcoholic steatohepatitis (NASH) progression

Xinzhi Li, Bingchuan Yuan, Min Lu, Yuqin Wang, Na Ding, Chunhong Liu, Ming Gao, Zhicheng Yao, Shiyan Zhang, Yujun Zhao, Liwei Xie, and Zheng Chen

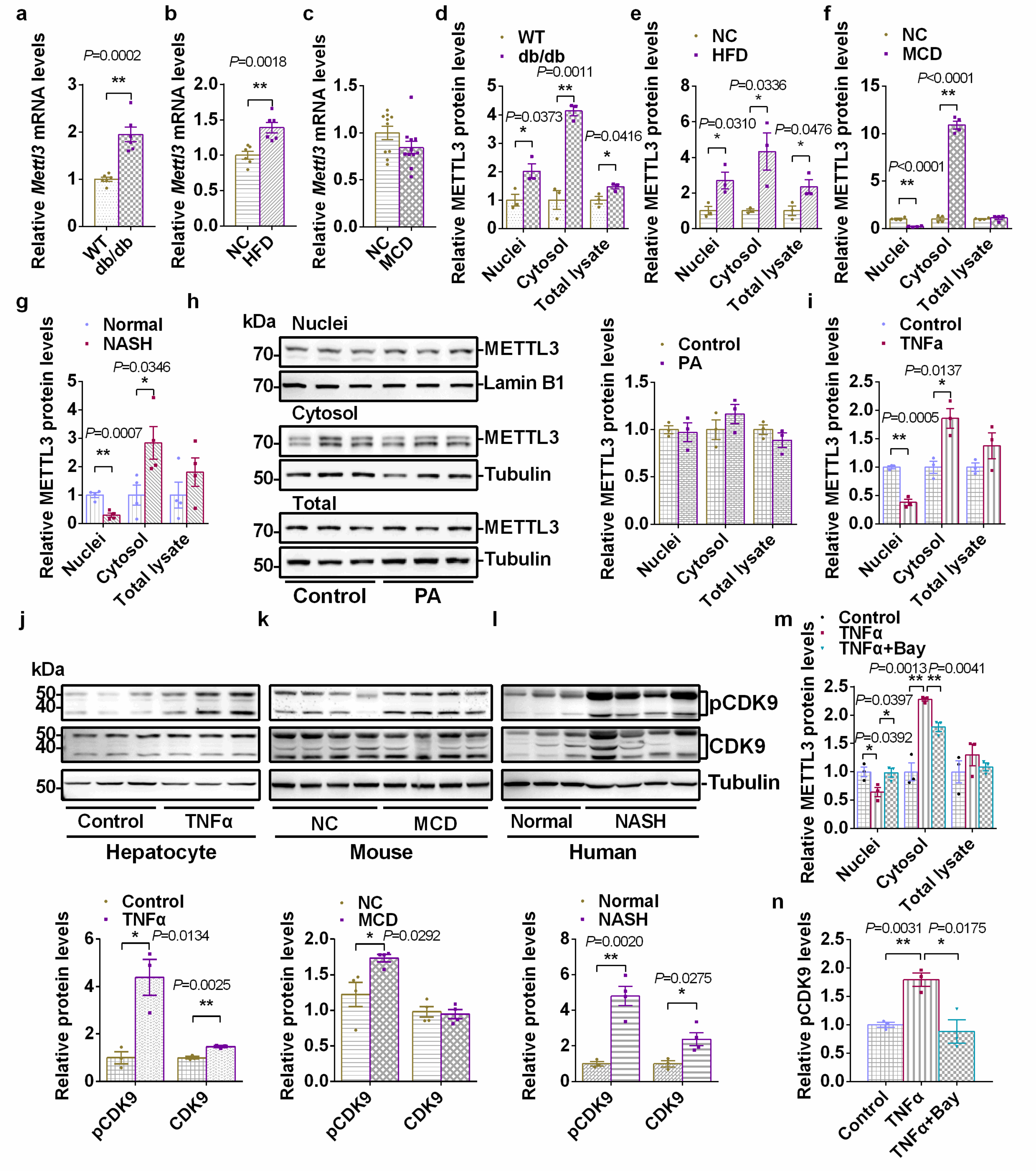

Supplementary Fig. 1. Decreased nuclear METTL3 was associated with CDK9 activation in NASH.

(a-c)*Mettl3* mRNA levels in livers of genetic obese (db/db) mice at 11 weeks age (n=6 for each group), high fat diet (HFD)-induced obese mice (n=6 for each group) and MCD-fed mice (NC, n=10; MCD, n=11).

(d-f) METTL3 protein levels in nuclei, cytosol and total cell lysate from the livers of db/db (11 weeks old, n=3 for each group) mice and HFD (for 8 weeks, n=3 for each group)- or MCD (for 3 weeks, n=4 for each group)-fed mice were measured by immunoblotting as shown in Fig. 1a-c. METTL3 protein levels were quantified by ImageJ and normalized to Tubulin.

(g) Immunoblotting of METTL3 protein levels in nuclei, cytosol and total cell lysate from human NASH and normal liver tissues as shown in Fig. 1d. METTL3 protein levels were quantified by ImageJ and normalized to Tubulin (n=4 for each group).

(h) Primary hepatocytes were isolated and treated with palmitic acid (PA)(0.5mM) overnight. METTL3 protein levels in nuclei, cytosol and total cell lysate were measured by immunoblotting. METTL3 protein levels were quantified by ImageJ and normalized to Tubulin (n=3 for each group).

(i) Primary hepatocytes were isolated and treated with TNFα (20 ng/ml) for 2 hours. METTL3 protein levels in nuclei, cytosol, and total cell lysate were measured by immunoblotting as shown in Fig. 1e. METTL3 protein levels were quantified by ImageJ and normalized to Tubulin (n=3 for each group).

(j) Primary hepatocytes were treated with TNFα (20ng/ml) for 2 hours. P-CDK9 and CDK9 protein levels were measured by immunoblotting, quantified by ImageJ and normalized to Tubulin (n=3 for each group).

(k-l) P-CDK9 and CDK9 protein levels in the livers of both mice (n=4 for each group) and human patients with NASH (Normal, n=3; NASH, n=4) were measured by immunoblotting, quantified by ImageJ and normalized to Tubulin.

(m-n) Primary hepatocytes were treated with vehicle, TNFα (20 ng/ml), and TNFα (20 ng/ml) plus BAY-1143572 (2 μM) for 2 hours. In TNFα plus BAY-1143572 group, primary hepatocytes were pretreated with BAY-1143572 (2 μM) for 2 hours. METTL3 protein levels in nuclei, cytosol, and total cell lysate were measured by immunoblotting. CDK9, p-CDK9, Lamin B1, and Tubulin levels were also measured by

immunoblotting as shown in Fig. 1i. METTL3 and p-CDK9 protein levels were quantified by ImageJ and normalized to Tubulin (n=3 for each group). The samples were derived from the same experiment and the blots were processed in parallel. n was the number of biologically independent mice or cell samples. The cell culture experiments were repeated for three times independently. Data represent the mean ± SEM. Significance was determined by unpaired two-tailed Student's *t* test analysis. *, $P< 0.05$. **, $P< 0.01$. Source data are provided as a Source Data file.

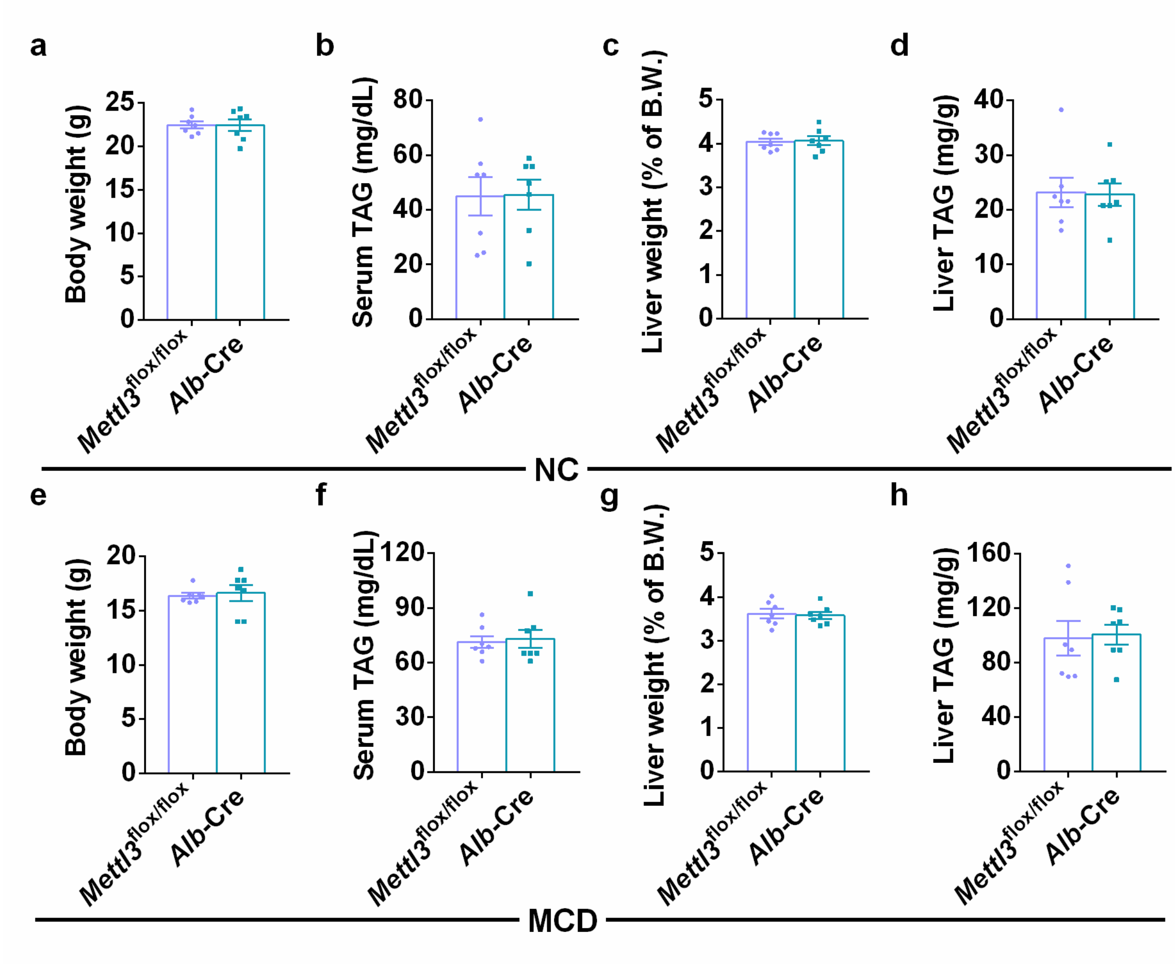

Supplementary Fig. 2. ***Mettl3*$^{\text{flox/flox}}$ mice display similar body weight, liver weights and liver TAG levels as *Alb*-Cre mice.**

(a-d) The body weight, plasma TAG levels, relative liver weights and liver TAG levels were measured in 8-week-old *Mettl3*$^{\text{flox/flox}}$ and *Alb*-Cre mice fed a normal chow diet (n=7 for each group).

(e-h) The body weight, plasma TAG levels, relative liver weights and liver TAG levels were measured in *Mettl3*$^{flox/flox}$ and *Alb*-Cre mice fed an MCD for 2 weeks (n=7 for each group)**.** n was the number of biologically independent mice. Data represent the mean ± SEM. Source data are provided as a Source Data file.

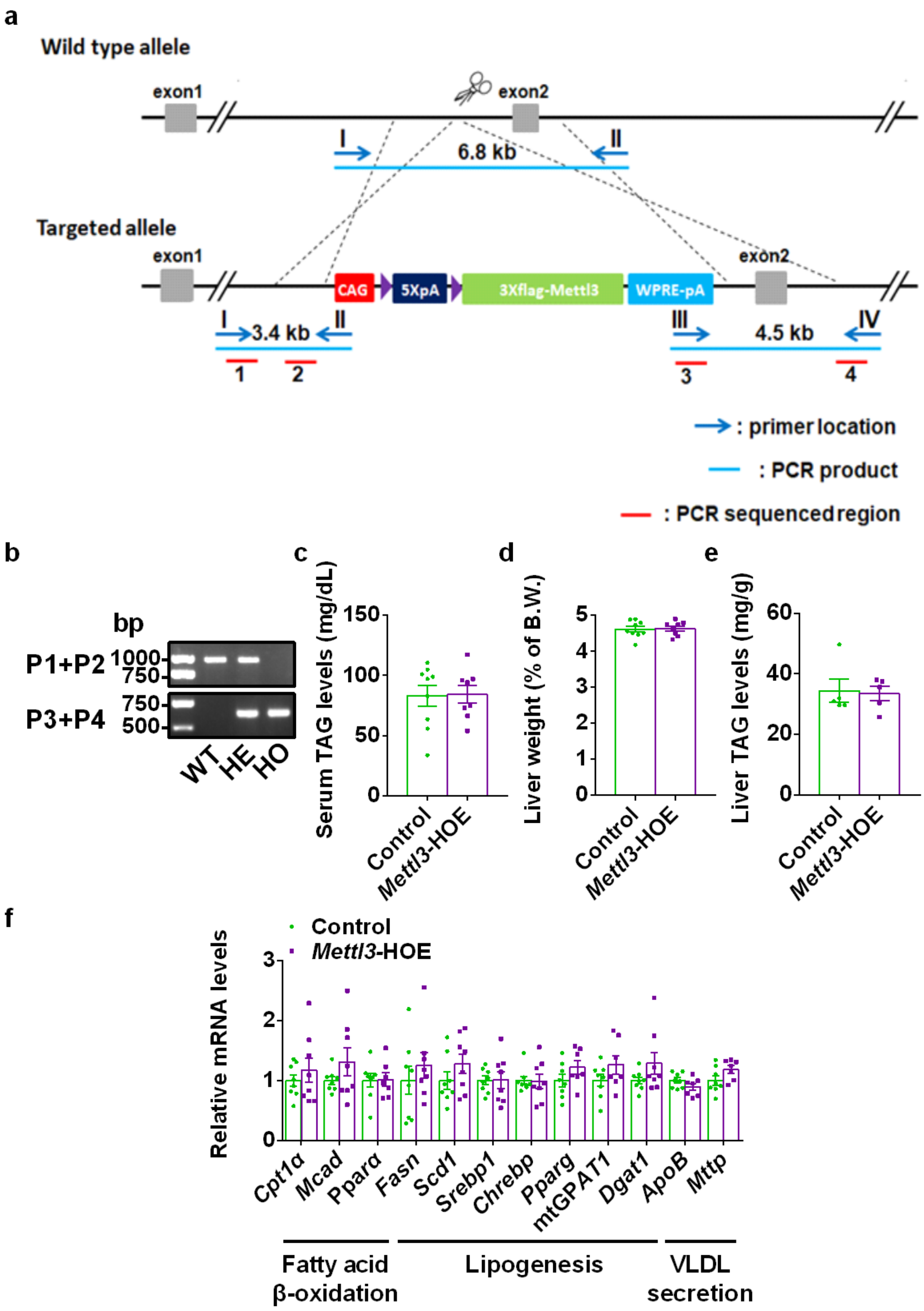

Supplementary Fig. 3. Generation of liver-specific *Mettl3*-overexpressing (HOE) mice.

(a)Generation of *STOP-Mettl3* mice.

(b) Genotyping of PCR products of *STOP-Mettl3* (homozygous), *STOP-Mettl3*$^{+/-}$(heterozygous) and WT mice. This experiment was repeated at least three times independently with similar results.

(c-e) Serum TAG levels (Control, n=9; *Mettl3*-HOE, n=8), liver weight (Control, n=9; *Mettl3*-HOE, n=8) and liver TAG levels (n=5 for each group) were measured in 8-week-old *Mettl3*-HOE and their control littermates fed a normal chow diet**.**

(f)Relative mRNA levels in the livers of Control and *Mettl3*-HOE mice at 8 weeks old were measured by RT-qPCR (Control, n=8; *Mettl3*-HOE, n=7-8). n was the number of biologically independent mice. Data represent the mean ± SEM. Source data are provided as a Source Data file.

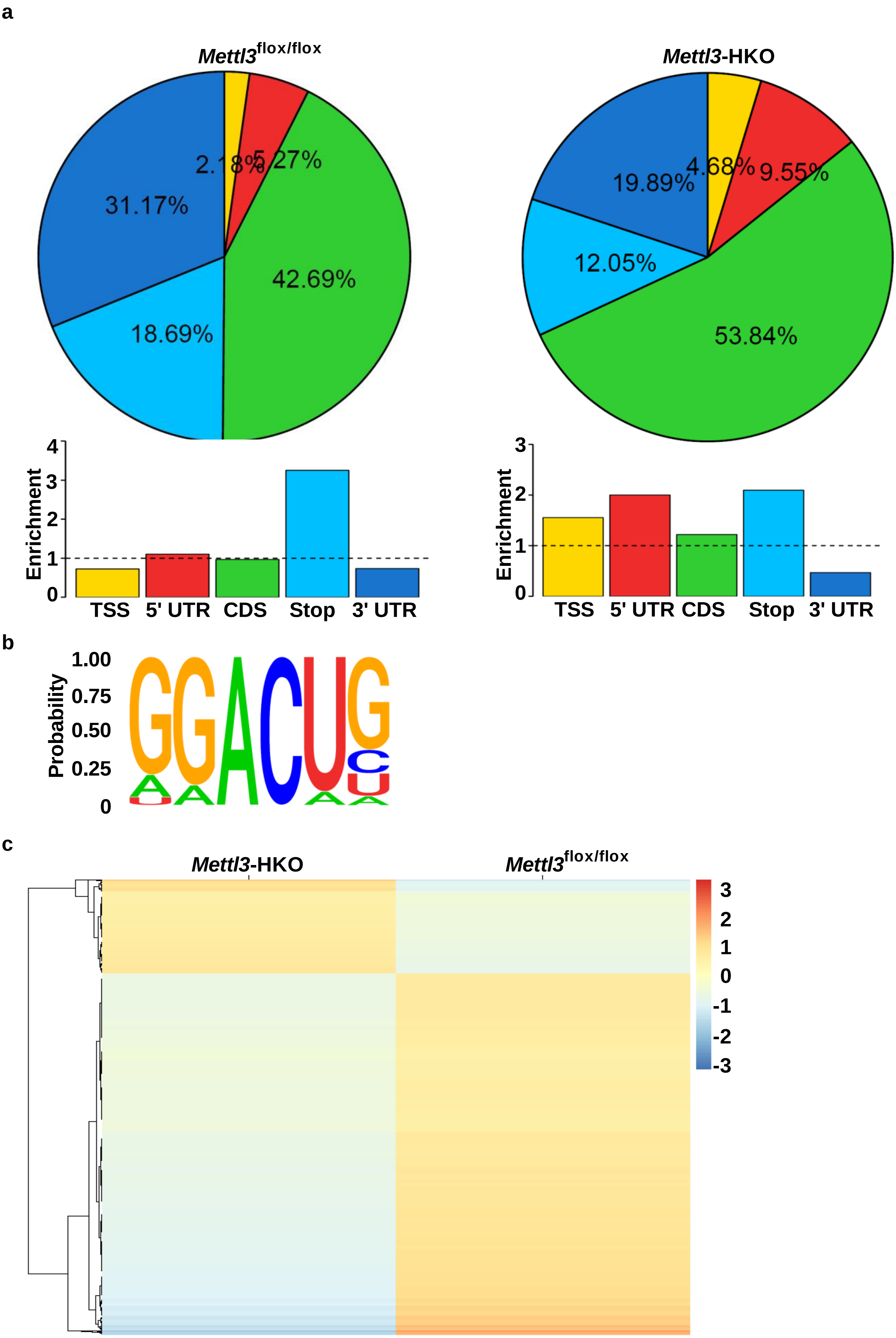
a
Mettl3flox/flox
Mettl3-HKO
2.18%
5.27%
42.69%
18.69%
31.17%
4.68%
9.55%
19.89%
12.05%
53.84%
Enrichment
TSS
5' UTR
CDS
Stop
3' UTR
b
Probability
1.00
0.75
0.50
0.25
0
c
Mettl3-HKO
Mettl3flox/flox
3
2
1
0
-1
-2
-3

Supplementary Fig. 4. METTL3 regulates RNA m^{6}A modification in the liver

The m^{6}A RNA immunoprecipitation sequencing(m^{6}ARIP-seq) analysis was performed in the livers of 8-week-old *Mettl3*$^{flox/flox}$ and *Mettl3*-HKO mice. (a) The enrichment of m^{6}ARIP-seq peaks in the livers of 8-week-old *Mettl3*$^{flox/flox}$ and *Mettl3*-HKO mice. (b) Consensus motif of m^{6}A sites in livers of 8-week-old *Mettl3*$^{flox/flox}$. (c) Heatmap of FoldEnrich in m^{6}A peaks (*Mettl3*-HKO versus *Mettl3*$^{flox/flox}$).

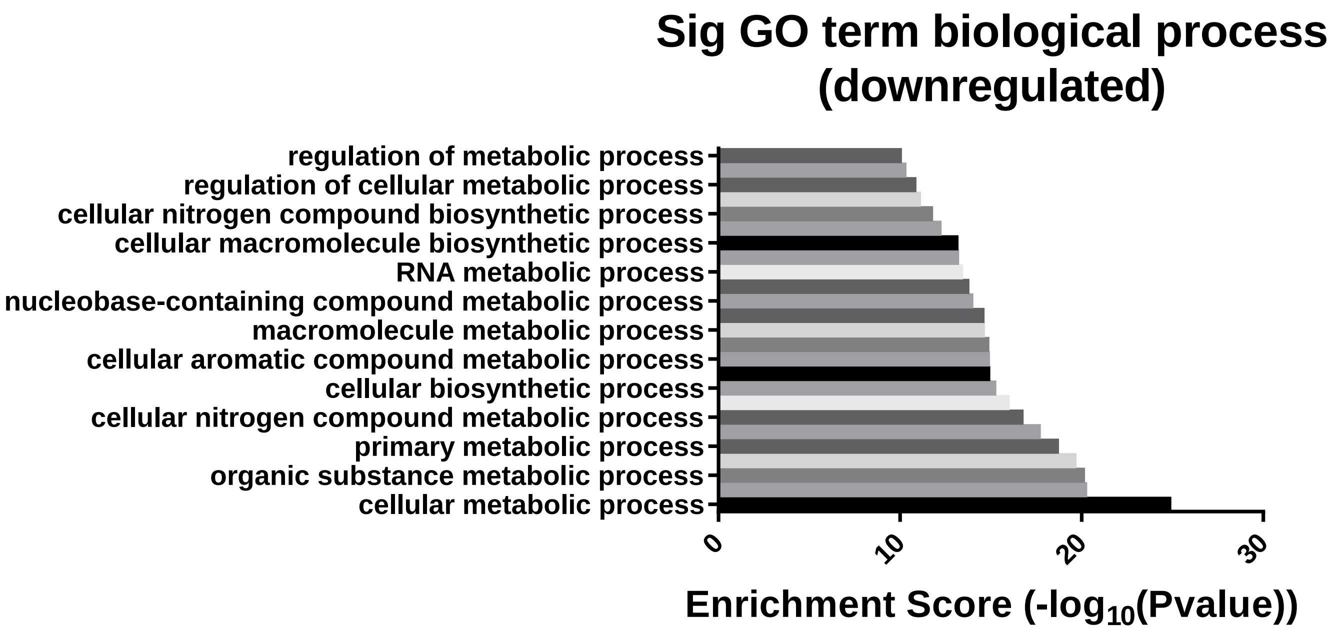

Supplementary Fig. 5. Genes with downregulated m^{6}A peaks are associated with metabolism.

The m^{6}A RNA immunoprecipitation sequencing(m^{6}ARIP-seq) analysis was performed in the livers of 8-week-old *Mettl3*$^{flox/flox}$ and *Mettl3*-HKO mice. GO analysis of the genes with downregulated m^{6}A peaks.

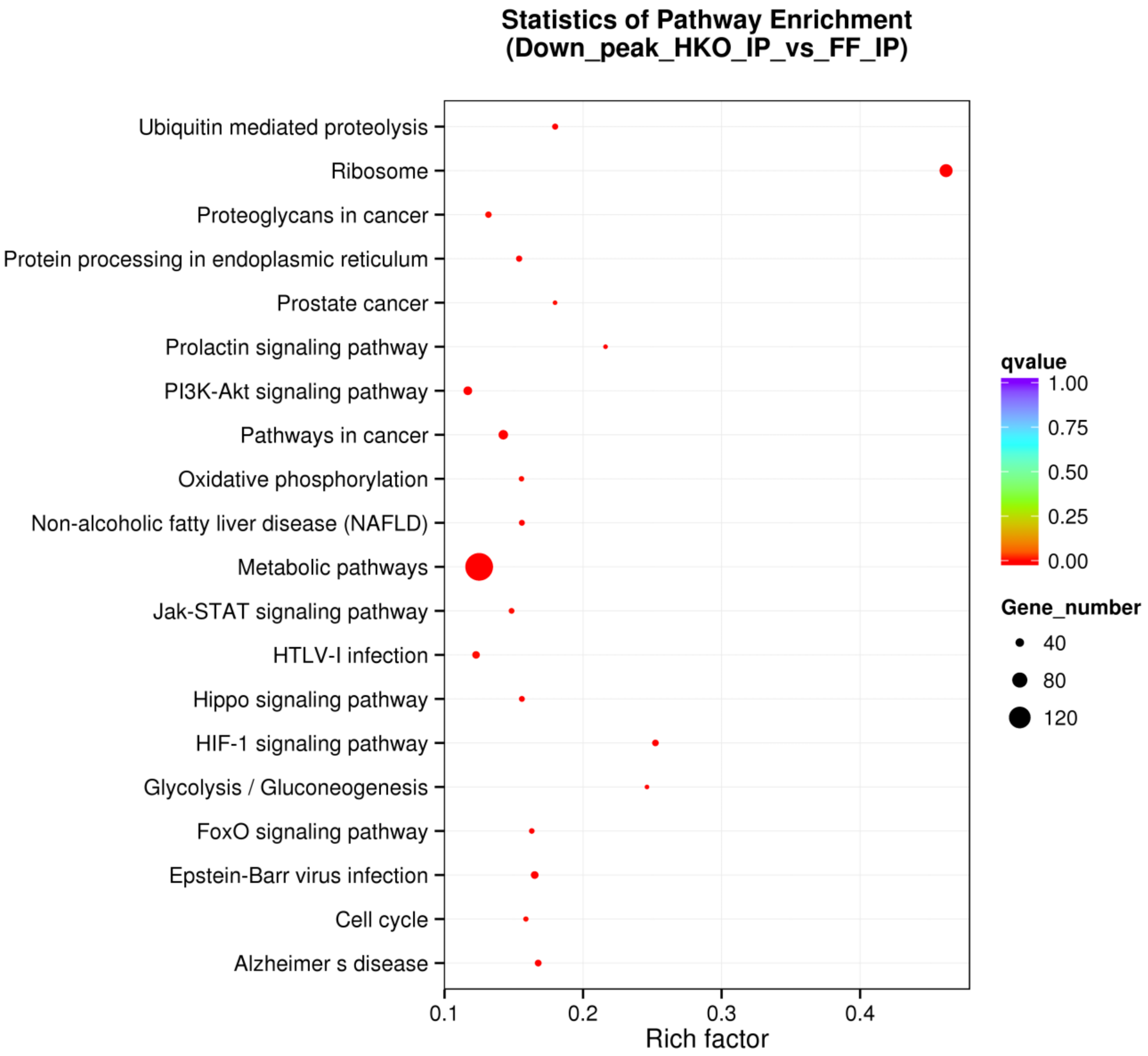

Supplementary Fig. 6. Genes with downregulated m^{6}A peaks are associated with signaling pathways.

The m^{6}A RNA immunoprecipitation sequencing(m^{6}ARIP-seq) analysis was performed in the livers of 8-week-old *Mettl3*$^{flox/flox}$ and *Mettl3*-HKO mice. KEGG analysis of the genes with downregulated m^{6}A peaks.

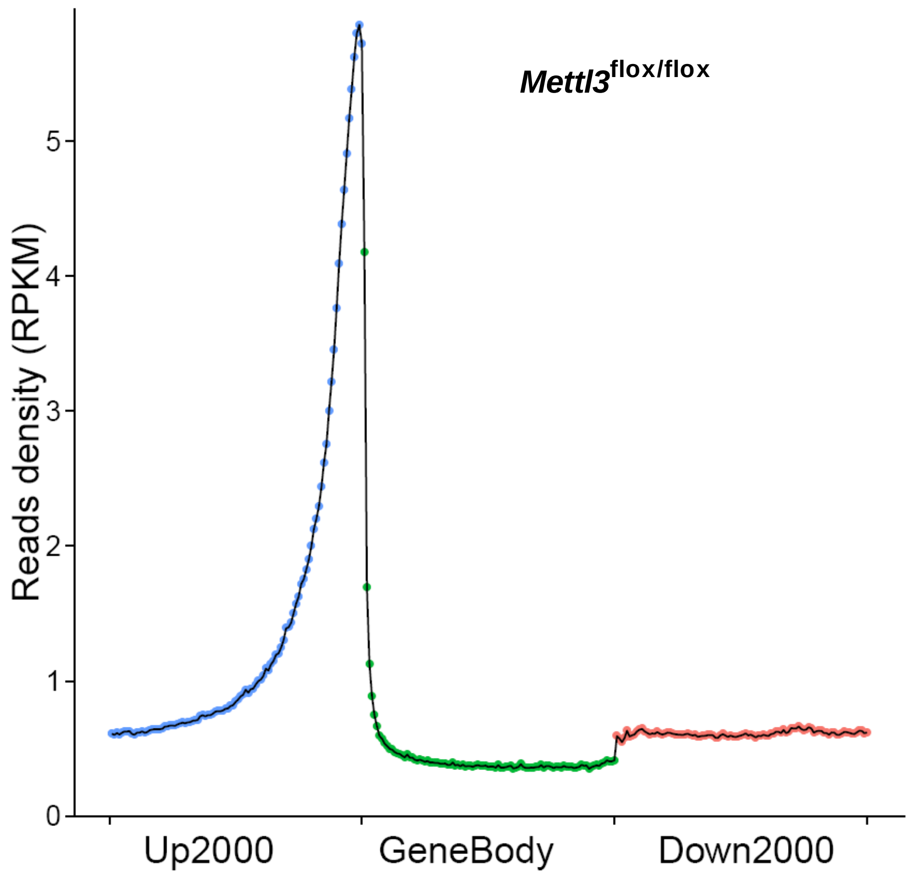
Mettl3flox/flox
Reads density (RPKM)
0
1
2
3
4
5
Up2000
GeneBody
Down2000

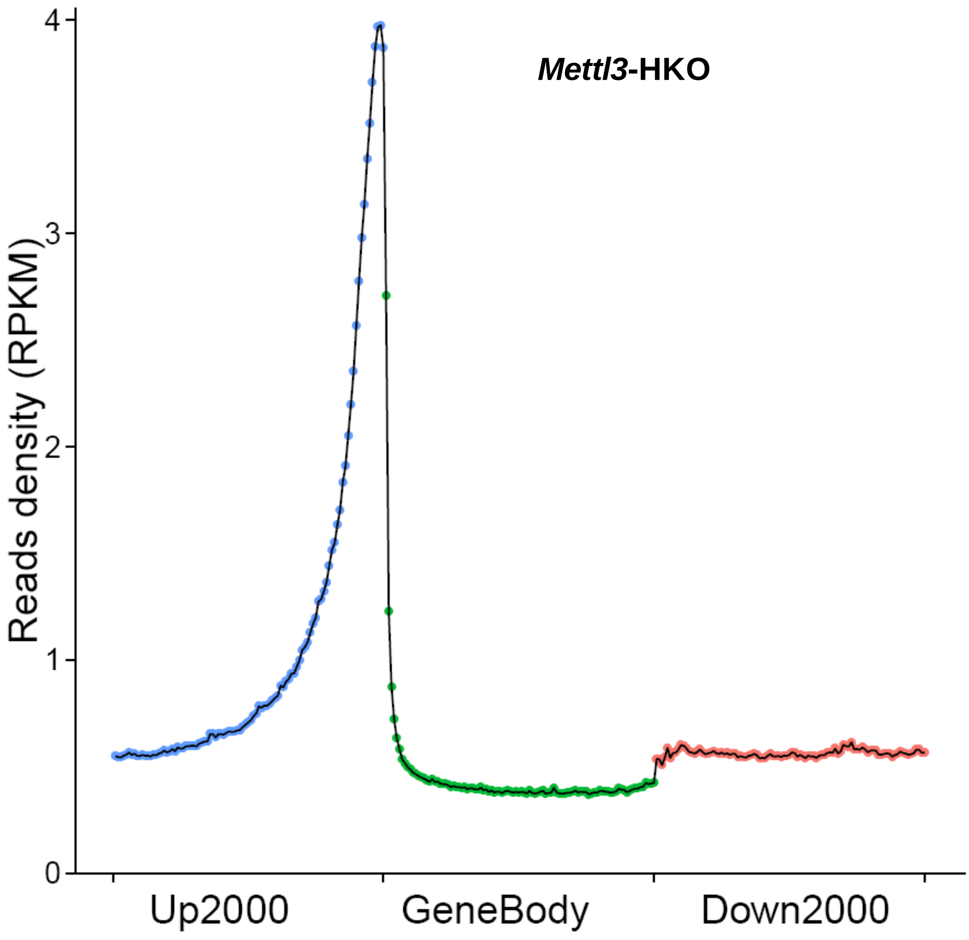
Mettl3-HKO
Reads density (RPKM)
0
1
2
3
4
Up2000
GeneBody
Down2000

Supplementary Fig. 7. The ATAC-seq peaks are located near a gene transcription start site in the livers of *Mettl3*$^{flox/flox}$ and *Mettl3*-HKO mice

The ATAC-seq reads density in Up2000, GeneBody and Down2000 were calculated in the livers of *Mettl3*$^{flox/flox}$ and *Mettl3*-HKO mice at 8 weeks old.

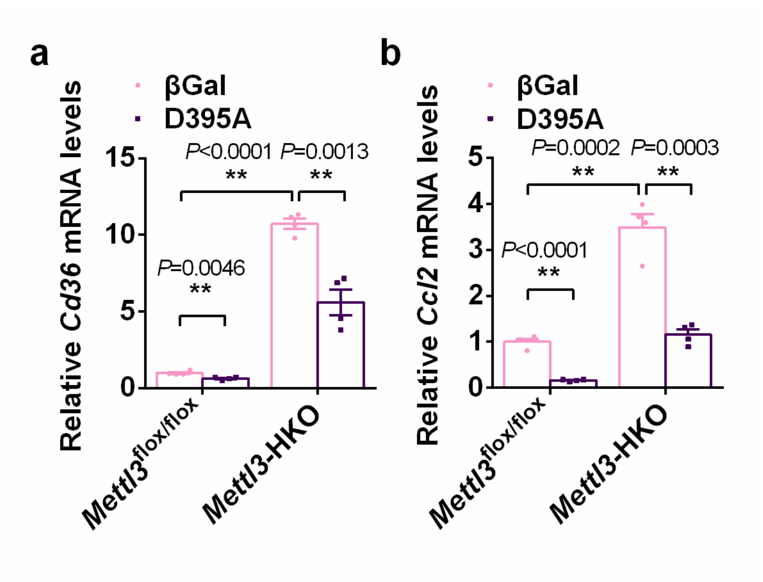

Supplementary Fig. 8. METTL3 mutation D395A suppresses the expression of *Cd36* and *Ccl2* in isolated hepatocytes from *Mettl3*-HKO mice

Primary hepatocytes were isolated from *Mettl3*-HKO mice at 8 weeks old and infected with Ad-βGal and Ad-METTL3 (D395A) adenovirus overnight. Relative *Cd36* and *Ccl2* mRNA levels were measured by qPCR (n=4 for each group). n was the number of biologically independent cell samples. The cell culture experiments were repeated for three times independently.

Data represent the mean ± SEM. Significance was determined by unpaired two-tailed Student's *t* test analysis. *, *P*< 0.05. **, *P*< 0.01. Source data are provided as a Source Data file.

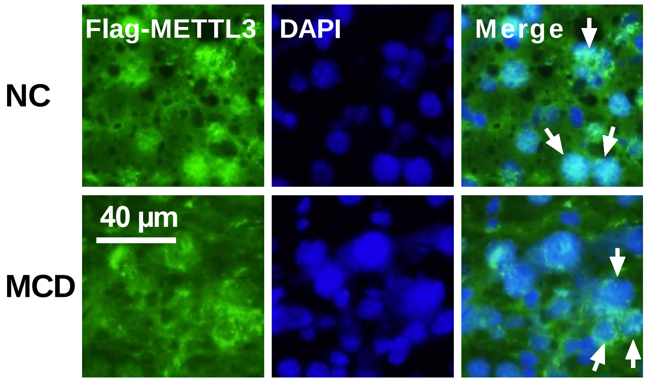

Supplementary Fig. 9. Flag-METTL3 in hepatocytes is translocated from nucleus to cytosol in *Mettl3*-HOE mice fed with an MCD for three weeks.

Mettl3-HOE mice were fed with an normal chow diet or an MCD for three weeks (n=4 for each group). Frozen liver sections were immunostained for Flag-METTL3. DAPI was used to identidy the nucleus. Representative liver sections were shown. Similar results were observed in all tested *Mettl3*-HOE mice(n=4 for each group). n was the number of biologically independent mice.

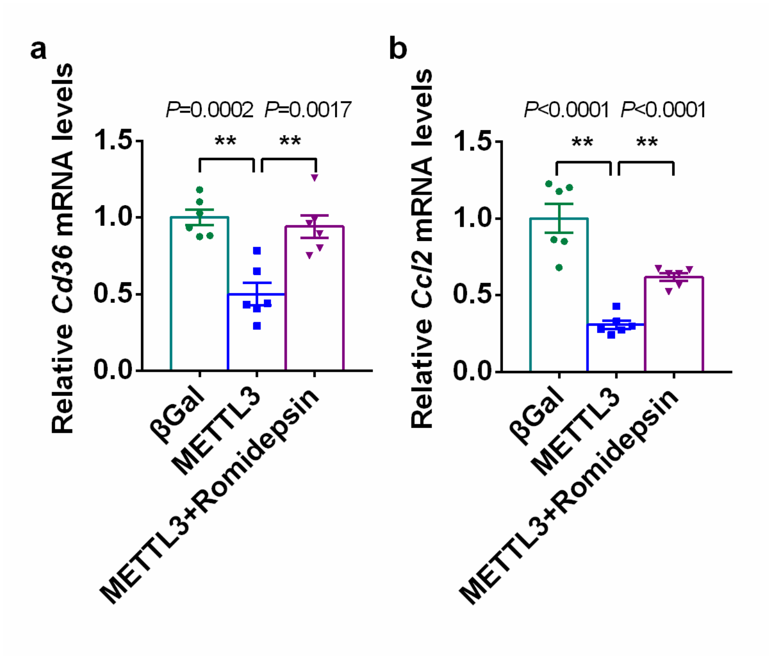

Supplementary Fig. 10. Romidepsin rescues the inhibition of *Cd36* and *Ccl2* expression mediated by METTL3 overexpression in primary hepatocytes.

Primary hepatocytes were isolated and infected with Ad-βGal and Ad-METTL3 adenovirus. Ad-METTL3-infected hepatocytes were treated with or without Romidepsin (5 nM) overnight.

(a, b) Relative *Cd36* and *Ccl2* mRNA levels were measured by qPCR (n=6 for each group). n was the number of biologically independent cell samples. The cell culture experiments were repeated for three times with similar results independently.

Data represent the mean ± SEM. Significance was determined by unpaired two-tailed Student's *t* test analysis. *, *P*< 0.05. **, *P*< 0.01. Source data are provided as a Source Data file.

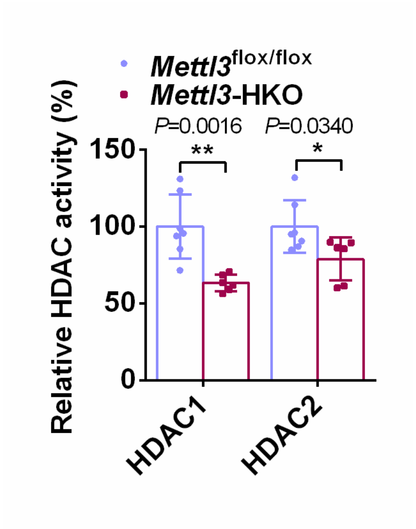

Supplementary Fig. 11. The activities of HDAC1 and HDAC2 are decreased in the livers of *Mettl3*-HKO mice.

HDAC1 or HDAC2 was immunoprecipitated from total lysate of *Mettl3*$^{flox/flox}$ (n=7) and *Mettl3*-HKO (n=6) mouse livers with HDAC1 or HDAC2 antibody. As a negative control, we used equal amount of IgG antibody to detect non-specific-bound HDAC activity. Immunoprecipitates were then assayed for HDAC activity using a commercial kit. HDAC1/2 activity values were corrected for non-specific (IgG)-bound HDAC activity and divided by the average HDAC1/2 activity value in *Mettl3*$^{flox/flox}$ mouse livers to determine the relative HDAC1/2 activity. n was the number of biologically independent mice. Data

represent the mean ± SEM. Significance was determined by unpaired two-tailed Student's *t* test analysis. *, $P < 0.05$. **, $P < 0.01$. Source data are provided as a Source Data file.

Supplementary Fig. 1h. Uncropped immunoblotting images.

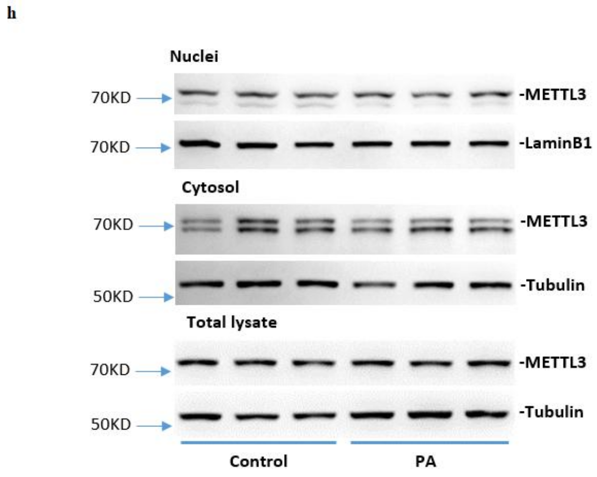

Relative protein levels

	Control			PA		
Nuclei	0.92998	0.99291	1.0771	1.00744	0.78451	1.12167
Cytosol	0.81626	1.17795	1.00579	1.21897	1.31015	0.96704
Total lys	0.9045	1.08366	1.01184	0.9394	0.74024	0.98601

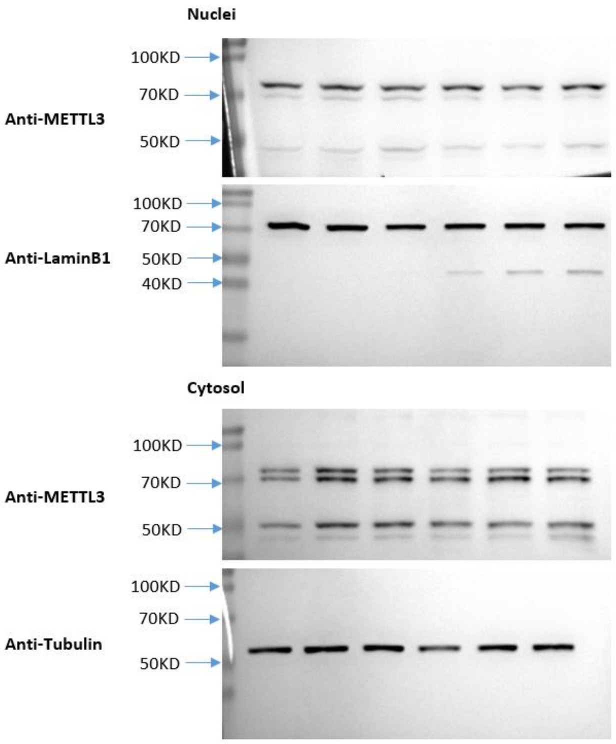

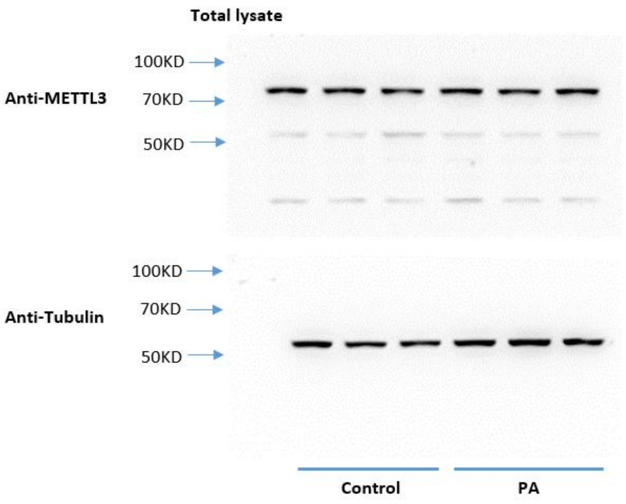

Supplementary Fig. 1j. Uncropped immunoblotting images.

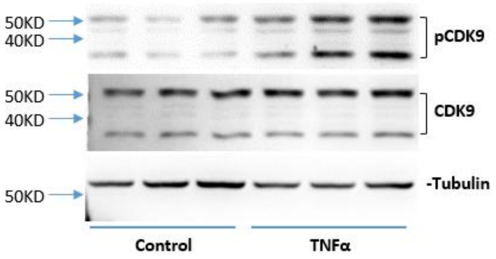

Relative protein levels

	Control			TNFα		
pCDK9	1.4459	0.5799	0.9742	2.893	5.3799	4.891
CDK9	1.1063	0.97	0.9238	1.5086	1.5279	1.386

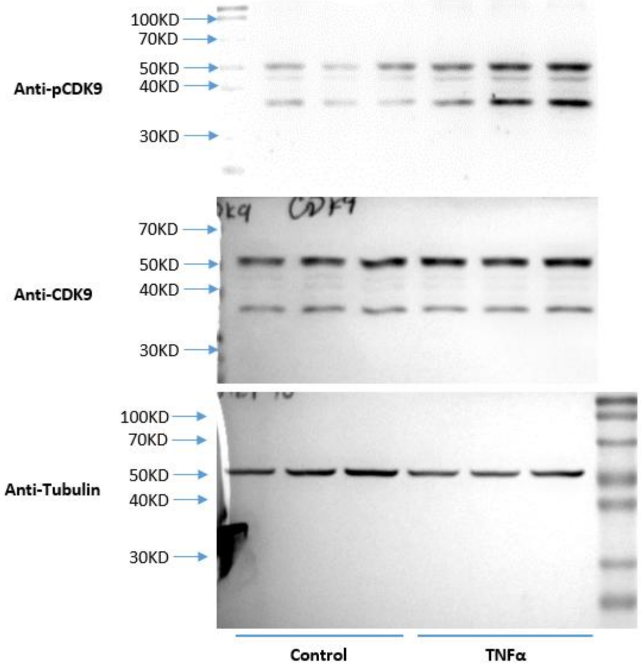

Supplementary Fig. 1k. Uncropped immunoblotting images.

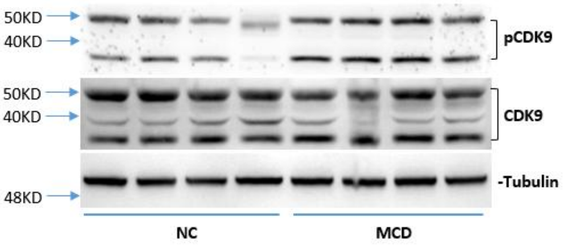

Relative protein levels

	NC				MCD			
pCDK9	1.4679	1.4212	1.2745	0.723	1.5717	1.783	1.799	1.7796
CDK9	0.8581	0.8543	1.159	1.0453	1.0885	0.8689	1.0156	0.8102

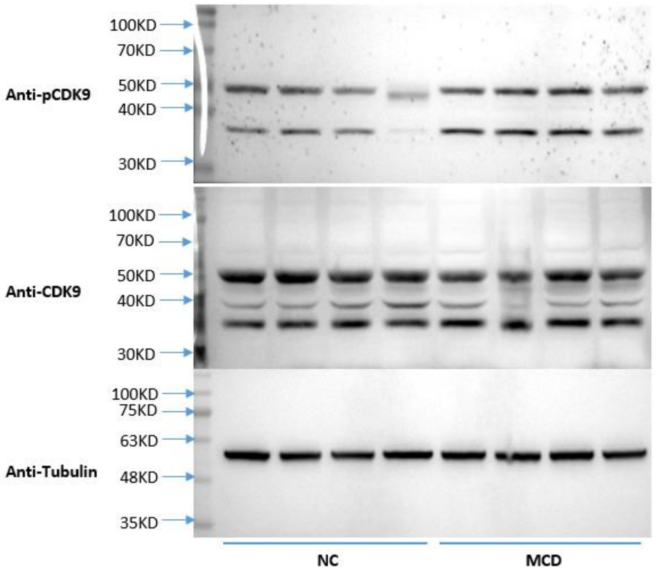

Supplementary Fig. 1l. Uncropped immunoblotting images.

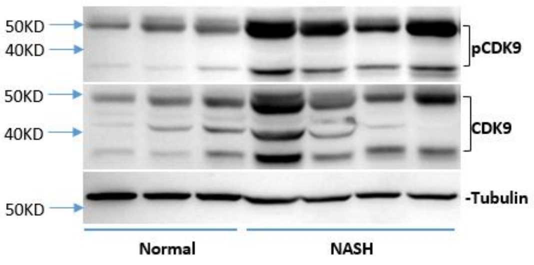

Relative protein levels

	Normal			NASH			
pCDK9	0.8185	0.9372	1.2443	4.7735	5.0776	3.3731	5.9653
CDK9	0.6775	1.0299	1.2926	3.3516	2.4682	1.7671	1.9315

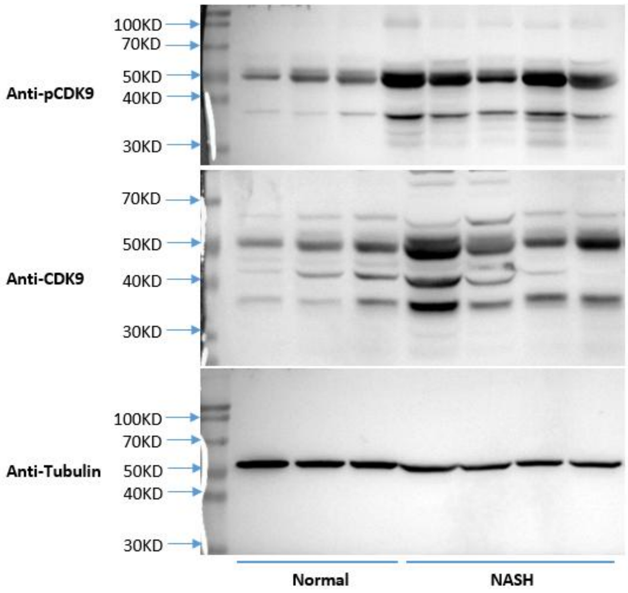

Supplementary Table 1. The clinical information and histologic features of subjects included in this study.

	**Non-steatosis**	**NASH**
All	9	10
Male Gender	3(33.3%)	8(80%)
Age (years)	41.78±4.36	39.6±3.63
BMI(kg/m^2)	21.83±0.85	23.03±0.4
ALT(U/L)	20±4.49	131.2±43.24
AST(U/L)	20±1.55	65.3±17.57
Cholesterol (mM)	3.95±0.25	4.97±0.5
Triglycerides (mM)	2.09±0.45	2.07±0.46
HDL(mM)	1.65±0.27	1.38±0.2
LDL(mM)	2.22±0.31	2.96±0.47
FBG(mM)	4.47±0.15	7.51±1.49
Steatosis grade (1/2/3)	0	3/4/3
Lobular inflammation (1/2/3)	0	3/5/2
Ballooning (0/1/2)	0	6/2/2
Fibrosis (0/1)	0	3/7

Data are expressed as mean±SEM. BMI, body mass index; ALT, alanine amino transferase; HDL, high-density lipoprotein; LDL, low-density lipoprotein; FBG, fasting blood glucose.

Supplementary Table 2. Primers for qPCR.

Genes	Forward	Reverse
Pparg	5'-CCAGAGTCTGCTGATCTGCG-3'	5'-GCCACCTCTTTGCTCTGATC-3'
Fasn	5'-TTGACGGCTCACACACCTAC-3'	5'-CGATCTTCCAGGCTCTTCAG-3'
Srebp1	5'-AACGTCACTTCCAGCTAGAC-3'	5'-CCACTAAGGTGCCTACAGAGC-3'
Scd1	5'-AGGTGCCTCTTAGCCACTGA-3'	5'-CCAGGAGTTTCTTGGGTTGA-3'
Cpt1α	5'-CTGATGACGGCTATGGTGTTT-3'	5'-GTGAGGCCAAACAAGGTGATA-3'
Ppara	5'-CCTGAACATCGAGTGTCGAATA-3'	5'-GGTCTTCTTCTGAATCTTGCAGCT-3'
Chrebp	5'-CTGGGGACCTAAACAGGAGC-3'	5'-GAAGCCACCCTATAGCTCCC-3'
mtGPAT1	5'-ACGCTGAGAGTGCCACATACT-3'	5'-GAGAGATCGCTACAGCACCAC-3'
Dgat1	5'-CGTGGTATCCTGAATTGGTG-3'	5'-GGCGCTTCTCAATCTGAAAT-3'
ApoB	5'-CCAGAGTGTGGAGCTGAATGT-3'	5'-TTGCTTTTTAGGGAGCCTAGC-3'
Mttp	5'-CTCCACAGTGCAGTTCTCACA-3'	5'-AGAGACATATCCCCTGCCTGT-3'
Mcad	5'-ACCCTGTGGAGAAGCTGATG-3'	5'-AGCAACAGTGCTTGGAGCTT-3'
Ccl2	5'-ACTGAAGCCAGCTCTCTCTTCCTC-3'	5'-TTCCTTCTTGGGGTCAGCACAGAC-3'
Tnfα	5'-CATCTTCTCAAAATTCGAGTGACAA-3'	5'-TGGGAGTAGACAAGGTACAACCC-3'
Il1b	5'-GCCTTGGGCCTCAAAGGAAAGAATC-3'	5'-GGAAGACACGGATTCCATGGTGAAG-3'
36B4	5'-AAGCGCGTCCTGGCATTGTCT-3'	5'-CCGCAGGGGCAGCAGTGGT-3'
Il6	5'-AGCCAGAGTCCTTCAGA-3'	5'-GGTCCTTAGCCACTCCT-3'
Ifng	5'-GCTACACACTGCATCTTGGC-3'	5'-CATGTCACCATCCTTTTGCCAG-3'
Cd36	5'-GGAGTGGTGATGTTTGTTGCT-3'	5'-GCACACACCACCATTTCTTCT-3'
Fatp2	5'-CCAAAAGCGGCAACCATCAA-3'	5'-AAGTAGCCCCAACCACGATG-3'
Fatp5	5'-TCCTTGGATTCCTTGGCTGC-3'	5'-CTGGTTGCTCAGGGACGTTA-3'
Mettl3	5'-CTTGCCATCTCTACGCCAGA-3'	5'-TCATGGCAGACAGCTTGGAG-3'
Tgfb1	5'-TTGCTTCAGCTCCACAGAGA-3'	5'-TGGTTGTAGAGGGCAAGGAC-3'
Mmp9	5'-CGTCGTGATCCCCACTTACT-3'	5'-AACACACAGGGTTTGCCTTC-3'
αSMA	5'-GGAGAAGCCCAGCCAGTCGC-3'	5'-AGCCGGCCTTACAGAGCCCA-3'
Collagen1a1	5'-TCACCTACAGCACCCTTGTG-3'	5'-GGTGGAGGGAGTTTACACGA-3'
Ccl2-ChIP	5'-AGCAGAGCCACTCCATTCAC-3'	5'-CCACTCCTGGGAGCAAATGA-3'
Cd36-ChIP	5'-CACCTGAGCCAAATGAAATGAA-3'	5'-CAGCAGAAAGAGGATTAAGGGT-3'
Actb-ChIP	5'-AATAGCCTCCGCCCTTGTG-3'	5'-CGTGACATCCACACCCAGA-3'

Supplementary Table 3

REAGENT or RESOURCE	SOURCE	IDENTIFIER
Chemicals		
TNFα	CUSABIO	Cat#CSB-AP002141HU
BAY-1143572	Selleck	Cat#S8727
Trichostatin A (TSA)	Selleck	Cat#S1045
Romidepsin	Selleck	Cat#S3020
3×Flag peptides	Sigma	Cat#F4799
Protein A Agarose	Pierce	Cat#20333
Anti-HA Magnetic Beads	Bimake	Cat#B26201
Anti-Myc Magnetic Beads	Bimake	Cat#B26301
Anti-FLAG M2 Magnetic Beads	Millipore	Cat#M8823
Glycerol Reagent	Sigma	Cat#F6428
DAPI	Solarbio	Cat#C0060
Palmitic acid	Sigma	Cat#P5585
Blue Plus II Protein Marker	Transgen Biotech	Cat#L50824
High Pure dNTPs	Transgen Biotech	Cat#20201025
GoTaq Green Master Mix	Promega	Cat#M7122
M-MLV reverse transcriptase	Promega	Cat#M1701
Random Primers	Promega	Cat#C1181

FBS	TRINTY TEK	Cat#01010102
Collagenase Type2	Worthington	Cat#LS004176
SYBR™ Green Mix	Roche	Cat#4913914001
TriPure Isolation Reagent	Roche	Cat#94015120
Recombinant DNase I	TaKaRa	Cat#2270A
MCD	Medicience	Cat#MD12052
HFD	Medicience	Cat#MD12032

Assay Kit		
Triglycerides Assay Kit	Nanjing Jiancheng	Cat#F001-1-1
TUNEL Assay Kit	Roche Applied Science	Cat#11684795910
Nuclear and Cytoplasmic Protein Extraction Kit	Beyotime	Cat#P0027
Caspase3 Assay Kit	abcam	Cat#ab39383
Alanine aminotransferase Assay Kit	Nanjing Jiancheng	Cat#C009-2-1
Epigenase™HDAC Activity/Inhibition Direct Assay Kit (Colorimetric)	Epigentek	Cat#P-4034

Supplementary Table 4. m^6ARIP-seq identifies 6,556 genes with m^6A peaks in the liver of *Mettl3*$^{flox/flox}$ mice.

ENSMUSG00000000001	Gnai3
ENSMUSG00000000056	Narf
ENSMUSG00000000078	Klf6
ENSMUSG00000000088	Cox5a
ENSMUSG00000000093	Tbx2
ENSMUSG00000000120	Ngfr
ENSMUSG00000000134	Tfe3
ENSMUSG00000000149	Gna12
ENSMUSG00000000171	Sdhd
ENSMUSG00000000194	Gpr107
ENSMUSG00000000247	Lhx2
ENSMUSG00000000275	Trim25
ENSMUSG00000000278	Scpep1
ENSMUSG00000000282	Mnt
ENSMUSG00000000301	Pemt
ENSMUSG00000000303	Cdh1
ENSMUSG00000000326	Comt
ENSMUSG00000000340	Dbt
ENSMUSG00000000346	Dazap2
ENSMUSG00000000355	Mcts1
ENSMUSG00000000374	Trappc10
ENSMUSG00000000378	Ccm2
ENSMUSG00000000420	Galnt1
ENSMUSG00000000439	Mkrn2
ENSMUSG00000000441	Raf1
ENSMUSG00000000531	Grasp
ENSMUSG00000000532	Acvr1b
ENSMUSG00000000538	Toml12
ENSMUSG00000000555	Itga5
ENSMUSG00000000561	Wdr77
ENSMUSG00000000567	Sox9
ENSMUSG00000000568	Hnrnpd
ENSMUSG00000000605	Clcn4
ENSMUSG00000000631	Myo18a
ENSMUSG00000000673	Haao
ENSMUSG00000000686	Abhd15
ENSMUSG00000000693	Loxl3
ENSMUSG00000000711	Rab5b
ENSMUSG00000000739	Sult5a1
ENSMUSG00000000740	Rpl13

ENSMUSG00000000743	Chmp1a
ENSMUSG00000000751	Rpa1
ENSMUSG00000000753	Serpinf1
ENSMUSG00000000776	Polr3d
ENSMUSG00000000787	Ddx3x
ENSMUSG00000000791	Il12rb1
ENSMUSG00000000804	Usp32
ENSMUSG00000000823	Zfp512b
ENSMUSG00000000827	Tpd52l2
ENSMUSG00000000876	Pxmp4
ENSMUSG00000000881	Dlg3
ENSMUSG00000000915	Hip1r
ENSMUSG00000000957	Mmp14
ENSMUSG00000000959	Oxa1l
ENSMUSG00000000976	Heatr6
ENSMUSG00000001017	Chtop
ENSMUSG00000001029	Icam2
ENSMUSG00000001034	Mapk7
ENSMUSG00000001036	Epn2
ENSMUSG00000001052	Sec24b
ENSMUSG00000001056	Nhp2
ENSMUSG00000001065	Zfp276
ENSMUSG00000001089	Luzp1
ENSMUSG00000001100	Poldip2
ENSMUSG00000001105	Ift20
ENSMUSG00000001119	Col6a1
ENSMUSG00000001123	Lgals9
ENSMUSG00000001138	Cnnm3
ENSMUSG00000001143	Lman2l
ENSMUSG00000001158	Snrnp27
ENSMUSG00000001175	Calm1
ENSMUSG00000001211	Agpat3
ENSMUSG00000001229	Dpp9
ENSMUSG00000001240	Ramp2
ENSMUSG00000001247	Lsr
ENSMUSG00000001249	Hpn
ENSMUSG00000001280	Sp1
ENSMUSG00000001288	Rarg
ENSMUSG00000001289	Pfdn5
ENSMUSG00000001300	Efnb2
ENSMUSG00000001323	Srr
ENSMUSG00000001348	Acp5
ENSMUSG00000001380	Hars
ENSMUSG00000001383	Zmat2

ENSMUSG00000001424	Snd1	
ENSMUSG00000001435	Col18a1	
ENSMUSG00000001436	Slc19a1	
ENSMUSG00000001440	Kpnb1	
ENSMUSG00000001445	Mrpl10	
ENSMUSG00000001467	Cyp51	
ENSMUSG00000001472	Tcf25	
ENSMUSG00000001482	Def8	
ENSMUSG00000001507	Itga3	
ENSMUSG00000001542	Ell2	
ENSMUSG00000001569	Nom1	
ENSMUSG00000001604	Tcea3	
ENSMUSG00000001630	Stk38l	
ENSMUSG00000001632	Brpf1	
ENSMUSG00000001666	Ddt	
ENSMUSG00000001670	Tat	
ENSMUSG00000001674	Ddx18	
ENSMUSG00000001751	Naglu	
ENSMUSG00000001755	Coasy	
ENSMUSG00000001768	Rin2	
ENSMUSG00000001783	Rtcb	
ENSMUSG00000001794	Capns1	
ENSMUSG00000001802	Lrp3	
ENSMUSG00000001833		7-Sep
ENSMUSG00000001847	Rac1	
ENSMUSG00000001855	Nup214	
ENSMUSG00000001910	Nacc1	
ENSMUSG00000001911	Nfix	
ENSMUSG00000001924	Uba1	
ENSMUSG00000001946	Esam	
ENSMUSG00000001962	Fam50a	
ENSMUSG00000001983	Taco1	
ENSMUSG00000002015	Bcap31	
ENSMUSG00000002017	Fam98a	
ENSMUSG00000002028	Kmt2a	
ENSMUSG00000002052	Supt6	
ENSMUSG00000002064	Sdf2	
ENSMUSG00000002083	Bbc3	
ENSMUSG00000002102	Psmc3	
ENSMUSG00000002103	Acp2	
ENSMUSG00000002108	Nr1h3	
ENSMUSG00000002111	Spi1	
ENSMUSG00000002147	Stat6	
ENSMUSG00000002221	Paxip1	

ENSMUSG00000002222	Rmnd5a
ENSMUSG00000002280	Narfl
ENSMUSG00000002289	Angptl4
ENSMUSG00000002307	Daxx
ENSMUSG00000002308	Cd320
ENSMUSG00000002320	Tm9sf1
ENSMUSG00000002325	Irf9
ENSMUSG00000002329	Mdp1
ENSMUSG00000002332	Dhrs1
ENSMUSG00000002346	Slc25a42
ENSMUSG00000002365	Snx9
ENSMUSG00000002393	Nr2f6
ENSMUSG00000002396	Ocel1
ENSMUSG00000002409	Dyrk1b
ENSMUSG00000002416	Ndufb2
ENSMUSG00000002455	Prpf6
ENSMUSG00000002475	Abhd3
ENSMUSG00000002496	Tsc2
ENSMUSG00000002504	Slc9a3r2
ENSMUSG00000002524	Puf60
ENSMUSG00000002550	Uck1
ENSMUSG00000002580	Mien1
ENSMUSG00000002602	Axl
ENSMUSG00000002608	Ccdc97
ENSMUSG00000002625	Akap8l
ENSMUSG00000002635	Pdcd2l
ENSMUSG00000002658	Gtf2f1
ENSMUSG00000002679	Med6
ENSMUSG00000002718	Csel1
ENSMUSG00000002741	Ykt6
ENSMUSG00000002748	Baz1b
ENSMUSG00000002768	Mea1
ENSMUSG00000002769	Gnmt
ENSMUSG00000002778	Kdelr1
ENSMUSG00000002797	Ggct
ENSMUSG00000002803	Btbd6
ENSMUSG00000002814	Top3a
ENSMUSG00000002820	Atg4d
ENSMUSG00000002831	Plin4
ENSMUSG00000002833	Hdgfl2
ENSMUSG00000002881	Nab1
ENSMUSG00000002885	Adgre5
ENSMUSG00000002897	Il17ra
ENSMUSG00000002908	Kcnn1

ENSMUSG00000002910	Arrdc2
ENSMUSG00000002948	Map2k7
ENSMUSG00000002949	Timm44
ENSMUSG00000002957	Ap2a2
ENSMUSG00000002968	Med25
ENSMUSG00000002981	Clptm1
ENSMUSG00000002985	Apoe
ENSMUSG00000002996	Hbp1
ENSMUSG00000003031	Cdkn1b
ENSMUSG00000003032	Klf4
ENSMUSG00000003037	Rab8a
ENSMUSG00000003039	Fam32a
ENSMUSG00000003053	Cyp2c29
ENSMUSG00000003068	Stk11
ENSMUSG00000003119	Cdk12
ENSMUSG00000003123	Lipe
ENSMUSG00000003134	Tbc1d8
ENSMUSG00000003161	Sri
ENSMUSG00000003166	Dgcr2
ENSMUSG00000003184	Irf3
ENSMUSG00000003198	Zfp959
ENSMUSG00000003199	Mpnd
ENSMUSG00000003200	Sh3gl1
ENSMUSG00000003226	Ranbp2
ENSMUSG00000003228	Grk5
ENSMUSG00000003233	Dvl3
ENSMUSG00000003234	Abcf3
ENSMUSG00000003299	Mrpl4
ENSMUSG00000003308	Keap1
ENSMUSG00000003316	Glg1
ENSMUSG00000003344	Btbd2
ENSMUSG00000003345	Csnk1g2
ENSMUSG00000003346	Abhd17a
ENSMUSG00000003360	Ddx23
ENSMUSG00000003378	Grik5
ENSMUSG00000003382	Etv3
ENSMUSG00000003402	Prkcsh
ENSMUSG00000003420	Fcgrt
ENSMUSG00000003421	Nosip
ENSMUSG00000003423	Pih1d1
ENSMUSG00000003435	Supt5
ENSMUSG00000003437	Paf1
ENSMUSG00000003438	Timm50
ENSMUSG00000003444	Med29

ENSMUSG00000003458	Ncstn
ENSMUSG00000003464	Pex19
ENSMUSG00000003477	Inmt
ENSMUSG00000003518	Dusp3
ENSMUSG00000003527	Ess2
ENSMUSG00000003531	Dgcr6
ENSMUSG00000003546	Klc4
ENSMUSG00000003585	Sec14l2
ENSMUSG00000003604	Aven
ENSMUSG00000003617	Cp
ENSMUSG00000003623	Crot
ENSMUSG00000003644	Rps6ka1
ENSMUSG00000003662	Ciao1
ENSMUSG00000003721	Insig2
ENSMUSG00000003731	Kpna6
ENSMUSG00000003746	Man1a
ENSMUSG00000003752	Itpkc
ENSMUSG00000003778	Brd8
ENSMUSG00000003809	Gcdh
ENSMUSG00000003810	Mast2
ENSMUSG00000003812	Dnase2a
ENSMUSG00000003814	Calr
ENSMUSG00000003824	Syce2
ENSMUSG00000003848	Nob1
ENSMUSG00000003873	Bax
ENSMUSG00000003923	Tfam
ENSMUSG00000003949	Hlf
ENSMUSG00000003970	Rpl8
ENSMUSG00000004018	Fancl
ENSMUSG00000004044	Cavin1
ENSMUSG00000004054	Map3k11
ENSMUSG00000004056	Akt2
ENSMUSG00000004069	Dnaja3
ENSMUSG00000004070	Hmox2
ENSMUSG00000004100	Ppan
ENSMUSG00000004105	Angptl2
ENSMUSG00000004221	Ikbkg
ENSMUSG00000004263	Atn1
ENSMUSG00000004264	Phb2
ENSMUSG00000004270	Lpcat3
ENSMUSG00000004319	Clcn3
ENSMUSG00000004356	Utp20
ENSMUSG00000004364	Cul3
ENSMUSG00000004383	Large1

ENSMUSG00000004393	Ddx56
ENSMUSG00000004394	Tmed4
ENSMUSG00000004446	Bid
ENSMUSG00000004455	Ppp1cc
ENSMUSG00000004460	Dnajb11
ENSMUSG00000004462	Tbccd1
ENSMUSG00000004500	Zfp324
ENSMUSG00000004558	Ndrg2
ENSMUSG00000004568	Arhgef18
ENSMUSG00000004591	Pkn2
ENSMUSG00000004610	Etfb
ENSMUSG00000004633	Chn2
ENSMUSG00000004655	Aqp1
ENSMUSG00000004677	Myo9b
ENSMUSG00000004768	Rab23
ENSMUSG00000004771	Rab11a
ENSMUSG00000004788	Eif2b2
ENSMUSG00000004789	Dlst
ENSMUSG00000004798	Ulk2
ENSMUSG00000004837	Grap
ENSMUSG00000004865	Srpk1
ENSMUSG00000004895	Prcc
ENSMUSG00000004897	Hdgf
ENSMUSG00000004931	Apba3
ENSMUSG00000004934	Pias4
ENSMUSG00000004937	Sgta
ENSMUSG00000004945	Tmem242
ENSMUSG00000004980	Hnrnpa2b1
ENSMUSG00000004994	Ccdc130
ENSMUSG00000005043	Sgsh
ENSMUSG00000005078	Jkamp
ENSMUSG00000005089	Slc1a2
ENSMUSG00000005107	Slc2a9
ENSMUSG00000005198	Polr2a
ENSMUSG00000005204	Senp3
ENSMUSG00000005251	Ripk4
ENSMUSG00000005268	Prlr
ENSMUSG00000005299	Letm1
ENSMUSG00000005370	Msh6
ENSMUSG00000005371	Fbxo11
ENSMUSG00000005373	Mlxipl
ENSMUSG00000005374	Tbl2
ENSMUSG00000005417	Mprip
ENSMUSG00000005442	Cic

ENSMUSG00000005469	Prkaca
ENSMUSG00000005481	Ddx39
ENSMUSG00000005483	Dnajb1
ENSMUSG00000005501	Usp40
ENSMUSG00000005505	Kbtbd4
ENSMUSG00000005514	Por
ENSMUSG00000005534	Insr
ENSMUSG00000005547	Cyp2a5
ENSMUSG00000005575	Ube2m
ENSMUSG00000005580	Adcy9
ENSMUSG00000005609	Ctr9
ENSMUSG00000005610	Eif4g2
ENSMUSG00000005615	Pcyt1a
ENSMUSG00000005621	Zfp592
ENSMUSG00000005625	Psmd4
ENSMUSG00000005674	Tomm40l
ENSMUSG00000005677	Nr1i3
ENSMUSG00000005687	Bcas2
ENSMUSG00000005698	Ctcf
ENSMUSG00000005705	Agrp
ENSMUSG00000005732	Ranbp1
ENSMUSG00000005774	Rfx5
ENSMUSG00000005779	Psmb4
ENSMUSG00000005802	Slc30a4
ENSMUSG00000005803	Sqor
ENSMUSG00000005846	Rsl1d1
ENSMUSG00000005871	Apc
ENSMUSG00000005873	Reep5
ENSMUSG00000005881	Ergic3
ENSMUSG00000005886	Ncoa2
ENSMUSG00000005893	Nr2c2
ENSMUSG00000005897	Nr2c1
ENSMUSG00000005936	Kctd20
ENSMUSG00000005949	Ctns
ENSMUSG00000005951	Shpk
ENSMUSG00000005973	Rcn1
ENSMUSG00000005981	Trap1
ENSMUSG00000005982	Naa60
ENSMUSG00000006005	Tpr
ENSMUSG00000006010	Odr4
ENSMUSG00000006014	Prg4
ENSMUSG00000006024	Napa
ENSMUSG00000006050	Sra1
ENSMUSG00000006127	Inpp5k

ENSMUSG00000006134	Crkl
ENSMUSG00000006169	Clint1
ENSMUSG00000006299	Aamp
ENSMUSG00000006301	Tmbim1
ENSMUSG00000006304	Arpc2
ENSMUSG00000006307	Kmt2b
ENSMUSG00000006344	Ggt5
ENSMUSG00000006378	Gcat
ENSMUSG00000006386	Tek
ENSMUSG00000006390	Elovl1
ENSMUSG00000006392	Med8
ENSMUSG00000006412	Pfdn2
ENSMUSG00000006445	Epha2
ENSMUSG00000006456	Rbm14
ENSMUSG00000006471	Ndor1
ENSMUSG00000006494	Pdk1
ENSMUSG00000006522	Itih3
ENSMUSG00000006526	Stimate
ENSMUSG00000006527	Sfmbt1
ENSMUSG00000006529	Itih1
ENSMUSG00000006538	Ihh
ENSMUSG00000006599	Gtf2h1
ENSMUSG00000006611	Hfe
ENSMUSG00000006638	Abhd1
ENSMUSG00000006673	Qrich1
ENSMUSG00000006711	D130043K22Rik
ENSMUSG00000006717	Acot13
ENSMUSG00000006728	Cdk4
ENSMUSG00000006731	B4galnt1
ENSMUSG00000006732	Mettl1
ENSMUSG00000006736	Tspan31
ENSMUSG00000006800	Sulf2
ENSMUSG00000006932	Ctnnb1
ENSMUSG00000006941	Eif1b
ENSMUSG00000006998	Psmd2
ENSMUSG00000007036	Abhd16a
ENSMUSG00000007038	Neu1
ENSMUSG00000007041	Clic1
ENSMUSG00000007415	Gatad1
ENSMUSG00000007476	Lrrc8a
ENSMUSG00000007570	Fance
ENSMUSG00000007594	Hapln4
ENSMUSG00000007656	Arpp19
ENSMUSG00000007659	Bcl2l1

ENSMUSG00000007670	Khsrp
ENSMUSG00000007721	Ccdc124
ENSMUSG00000007777	0610009B22Rik
ENSMUSG00000007812	Zfp655
ENSMUSG00000007817	Zmiz1
ENSMUSG00000007833	Aldh16a1
ENSMUSG00000007836	Hnrnpa0
ENSMUSG00000007872	Id3
ENSMUSG00000007880	Arid1a
ENSMUSG00000007891	Ctsd
ENSMUSG00000007944	Ttc9b
ENSMUSG00000008035	Mid1ip1
ENSMUSG00000008090	Fgfrl1
ENSMUSG00000008200	Fnbp4
ENSMUSG00000008206	Cers4
ENSMUSG00000008226	Scrn3
ENSMUSG00000008301	Phax
ENSMUSG00000008305	Tle1
ENSMUSG00000008348	Ubc
ENSMUSG00000008373	Prpf31
ENSMUSG00000008384	Sertad1
ENSMUSG00000008393	Carhsp1
ENSMUSG00000008398	Elk3
ENSMUSG00000008429	Herpud2
ENSMUSG00000008475	Arpc5
ENSMUSG00000008540	Mgst1
ENSMUSG00000008575	Nfib
ENSMUSG00000008604	Ubqln4
ENSMUSG00000008690	Ncaph2
ENSMUSG00000008763	Man1a2
ENSMUSG00000008855	Hdac5
ENSMUSG00000008859	Rala
ENSMUSG00000008976	Gabpa
ENSMUSG00000009013	Dynll1
ENSMUSG00000009030	Pdcl
ENSMUSG00000009035	Tmem184b
ENSMUSG00000009079	Ewsr1
ENSMUSG00000009092	Derl3
ENSMUSG00000009112	Bcl2l13
ENSMUSG00000009145	Dqx1
ENSMUSG00000009281	Rarres2
ENSMUSG00000009291	Pttg1ip
ENSMUSG00000009293	Ube2g2
ENSMUSG00000009376	Met

ENSMUSG00000009378	Slc16a12
ENSMUSG00000009418	Nav1
ENSMUSG00000009470	Tnpo1
ENSMUSG00000009549	Srp14
ENSMUSG00000009555	Cdk9
ENSMUSG00000009563	Tor2a
ENSMUSG00000009569	Mrtfb
ENSMUSG00000009630	Ppp2cb
ENSMUSG00000009633	G0s2
ENSMUSG00000009646	Pla2g12b
ENSMUSG00000009654	Oit3
ENSMUSG00000009681	Bcr
ENSMUSG00000009741	Ubp1
ENSMUSG00000009772	Nuak2
ENSMUSG00000009894	Snap47
ENSMUSG00000009905	Kdsr
ENSMUSG00000010025	Aldh3a2
ENSMUSG00000010045	Tmem115
ENSMUSG00000010047	Hyal2
ENSMUSG00000010051	Hyal1
ENSMUSG00000010054	Tusc2
ENSMUSG00000010095	Slc3a2
ENSMUSG00000010122	Slc47a1
ENSMUSG00000010175	Prox1
ENSMUSG00000010277	2610507B11Rik
ENSMUSG00000010307	Tmem86a
ENSMUSG00000010376	Nedd8
ENSMUSG00000010392	Gosr1
ENSMUSG00000010453	Kansl3
ENSMUSG00000010554	Mettl16
ENSMUSG00000010601	Apol7a
ENSMUSG00000010607	Pigyl
ENSMUSG00000010608	Rbm25
ENSMUSG00000010609	Psen2
ENSMUSG00000010651	Acaa1b
ENSMUSG00000010755	Cars
ENSMUSG00000010797	Wnt2
ENSMUSG00000010914	Pdhx
ENSMUSG00000010936	Vac14
ENSMUSG00000011148	Adssl1
ENSMUSG00000011257	Pabpc4
ENSMUSG00000011263	Exoc3l2
ENSMUSG00000011305	Plin5
ENSMUSG00000011382	Dhdh

ENSMUSG00000011427	Zfp790
ENSMUSG00000011831	Evi5
ENSMUSG00000011837	Snapc2
ENSMUSG00000011877	Git1
ENSMUSG00000011960	Ccnt1
ENSMUSG00000012017	Scarf2
ENSMUSG00000012117	Dhdds
ENSMUSG00000012296	Tjap1
ENSMUSG00000012422	Tmem167
ENSMUSG00000012428	Steap4
ENSMUSG00000012429	Mplkip
ENSMUSG00000012640	Zfp715
ENSMUSG00000012819	Cdh23
ENSMUSG00000013150	Gfod2
ENSMUSG00000013593	Ndufs2
ENSMUSG00000013622	Atraid
ENSMUSG00000013646	Sh3bp5l
ENSMUSG00000013663	Pten
ENSMUSG00000013701	Timm23
ENSMUSG00000013707	Tnfaip8l2
ENSMUSG00000013736	Trnt1
ENSMUSG00000013787	Ehmt2
ENSMUSG00000013822	Elof1
ENSMUSG00000013846	St3gal1
ENSMUSG00000013858	Tmem259
ENSMUSG00000013997	Nit1
ENSMUSG00000014074	Rnf168
ENSMUSG00000014164	Klhl3
ENSMUSG00000014195	Dnajc7
ENSMUSG00000014226	Cacybp
ENSMUSG00000014245	Pigl
ENSMUSG00000014294	Ndufa2
ENSMUSG00000014353	Tmem87b
ENSMUSG00000014355	Anapc1
ENSMUSG00000014361	Mertk
ENSMUSG00000014426	Map3k4
ENSMUSG00000014496	Ankrd28
ENSMUSG00000014504	Srp19
ENSMUSG00000014542	Clec4f
ENSMUSG00000014547	Wdfy2
ENSMUSG00000014550	Rbsn
ENSMUSG00000014551	Mrps25
ENSMUSG00000014599	Csf1
ENSMUSG00000014601	Strip1

ENSMUSG00000014606	Slc25a11
ENSMUSG00000014633	Cmc2
ENSMUSG00000014763	Fam120b
ENSMUSG00000014773	Dll1
ENSMUSG00000014850	Msh3
ENSMUSG00000014867	Surf4
ENSMUSG00000014905	Dnajb9
ENSMUSG00000014932	Yes1
ENSMUSG00000014956	Ppp1cb
ENSMUSG00000015027	Galns
ENSMUSG00000015112	Slc25a13
ENSMUSG00000015126	Tsr3
ENSMUSG00000015133	Lrrk1
ENSMUSG00000015143	Actn1
ENSMUSG00000015165	Hnrnpl
ENSMUSG00000015176	Nolc1
ENSMUSG00000015189	Casd1
ENSMUSG00000015202	Cnksr3
ENSMUSG00000015243	Abca1
ENSMUSG00000015289	Lage3
ENSMUSG00000015291	Gdi1
ENSMUSG00000015305	Sash1
ENSMUSG00000015312	Gadd45b
ENSMUSG00000015357	Clpx
ENSMUSG00000015363	Trabd
ENSMUSG00000015396	Cd83
ENSMUSG00000015478	Rnf5
ENSMUSG00000015488	Cacfd1
ENSMUSG00000015501	Hivep2
ENSMUSG00000015542	Nat9
ENSMUSG00000015568	Lpl
ENSMUSG00000015597	Zfp318
ENSMUSG00000015605	Srf
ENSMUSG00000015672	Mrpl32
ENSMUSG00000015697	Setdb1
ENSMUSG00000015711	Prune1
ENSMUSG00000015714	Cers2
ENSMUSG00000015745	Plekho1
ENSMUSG00000015749	Anp32e
ENSMUSG00000015750	Aph1a
ENSMUSG00000015755	Tab2
ENSMUSG00000015757	Ppil4
ENSMUSG00000015776	Med22
ENSMUSG00000015839	Nfe2l2

ENSMUSG00000015846	Rxra
ENSMUSG00000015854	Cd5l
ENSMUSG00000015890	Amdhd1
ENSMUSG00000015937	H2afy
ENSMUSG00000015947	Fcgr1
ENSMUSG00000015961	Adss
ENSMUSG00000015966	Il17rb
ENSMUSG00000015970	Chdh
ENSMUSG00000015994	Fnta
ENSMUSG00000016018	Mtrex
ENSMUSG00000016024	Lbp
ENSMUSG00000016028	Celsr1
ENSMUSG00000016087	Fli1
ENSMUSG00000016128	Stard13
ENSMUSG00000016181	Diexf
ENSMUSG00000016256	Ctsz
ENSMUSG00000016257	Prelid3b
ENSMUSG00000016319	Slc25a5
ENSMUSG00000016344	Ppdpf
ENSMUSG00000016477	E2f3
ENSMUSG00000016487	Ppfibp1
ENSMUSG00000016503	Gtf3a
ENSMUSG00000016510	Mtif3
ENSMUSG00000016520	Lnx2
ENSMUSG00000016619	Nup50
ENSMUSG00000016664	Pacsin2
ENSMUSG00000016756	Cmah
ENSMUSG00000016831	Tox4
ENSMUSG00000016833	Mrps18c
ENSMUSG00000016921	Srsf6
ENSMUSG00000016933	Plcg1
ENSMUSG00000016940	Kctd2
ENSMUSG00000017009	Sdc4
ENSMUSG00000017057	Il13ra1
ENSMUSG00000017119	Nbr1
ENSMUSG00000017132	Cyth1
ENSMUSG00000017144	Rnd3
ENSMUSG00000017188	Coa3
ENSMUSG00000017221	Psmd3
ENSMUSG00000017286	Glod4
ENSMUSG00000017309	Cd300lg
ENSMUSG00000017386	Traf4
ENSMUSG00000017412	Cacnb4
ENSMUSG00000017421	Zfp207

ENSMUSG00000017446	C1qtnf1
ENSMUSG00000017453	Pipox
ENSMUSG00000017478	Zc3h18
ENSMUSG00000017548	Suz12
ENSMUSG00000017615	Tnfaip1
ENSMUSG00000017631	Abr
ENSMUSG00000017670	Elmo2
ENSMUSG00000017679	Ttpal
ENSMUSG00000017715	Pgs1
ENSMUSG00000017718	Afmid
ENSMUSG00000017747	Ghdc
ENSMUSG00000017756	Slc12a7
ENSMUSG00000017760	Ctsa
ENSMUSG00000017776	Crk
ENSMUSG00000017781	Pitpna
ENSMUSG00000017802	Retreg3
ENSMUSG00000017831	Rab5a
ENSMUSG00000017858	Ift52
ENSMUSG00000017868	Sgk2
ENSMUSG00000017929	B4galt5
ENSMUSG00000017950	Hnf4a
ENSMUSG00000017999	Ddx27
ENSMUSG00000018076	Med13l
ENSMUSG00000018102	Hist1h2bc
ENSMUSG00000018143	Mafk
ENSMUSG00000018160	Med1
ENSMUSG00000018166	Erbb3
ENSMUSG00000018167	Stard3
ENSMUSG00000018171	Vmp1
ENSMUSG00000018287	Spag7
ENSMUSG00000018293	Pfn1
ENSMUSG00000018322	Tomm34
ENSMUSG00000018326	Ywhab
ENSMUSG00000018347	Zkscan6
ENSMUSG00000018363	Smurf2
ENSMUSG00000018379	Srsf1
ENSMUSG00000018381	Abi3
ENSMUSG00000018387	Shroom1
ENSMUSG00000018401	Mtmr4
ENSMUSG00000018412	Kansl1
ENSMUSG00000018415	Gid4
ENSMUSG00000018425	Dhx40
ENSMUSG00000018428	Akap1
ENSMUSG00000018433	Nol11

ENSMUSG00000018442	Derl2
ENSMUSG00000018459	Slc13a3
ENSMUSG00000018474	Chd3
ENSMUSG00000018476	Kdm6b
ENSMUSG00000018481	Appbp2
ENSMUSG00000018501	Ncor1
ENSMUSG00000018509	Cenpv
ENSMUSG00000018537	Pcgf2
ENSMUSG00000018541	Cwc25
ENSMUSG00000018547	Pip4k2b
ENSMUSG00000018559	Ctdnep1
ENSMUSG00000018567	Gabarap
ENSMUSG00000018572	Phf23
ENSMUSG00000018574	Acadvl
ENSMUSG00000018583	G3bp1
ENSMUSG00000018585	Atox1
ENSMUSG00000018593	Sparc
ENSMUSG00000018599	Mief2
ENSMUSG00000018604	Tbx3
ENSMUSG00000018661	Cog1
ENSMUSG00000018707	Dync1h1
ENSMUSG00000018727	Cpsf4l
ENSMUSG00000018750	Zbtb4
ENSMUSG00000018752	Tnfsfm13
ENSMUSG00000018761	Mpdu1
ENSMUSG00000018765	Fxr2
ENSMUSG00000018770	Atp5g3
ENSMUSG00000018774	Cd68
ENSMUSG00000018796	Acsl1
ENSMUSG00000018821	Avpi1
ENSMUSG00000018849	Wwc1
ENSMUSG00000018858	Mrpl58
ENSMUSG00000018861	Fdxr
ENSMUSG00000018882	Mrpl45
ENSMUSG00000018900	Slc22a5
ENSMUSG00000018921	Pelp1
ENSMUSG00000018923	Med11
ENSMUSG00000018932	Map2k3
ENSMUSG00000018965	Ywhah
ENSMUSG00000018974	Sart3
ENSMUSG00000018995	Nars2
ENSMUSG00000018999	Slc35b4
ENSMUSG00000019082	Slc25a22
ENSMUSG00000019087	Atp6ap1

ENSMUSG00000019122	Ccl9
ENSMUSG00000019132	BC005537
ENSMUSG00000019143	Hars2
ENSMUSG00000019158	Tmem160
ENSMUSG00000019188	H13
ENSMUSG00000019232	Etnppl
ENSMUSG00000019254	Ppp1r12c
ENSMUSG00000019256	Ahr
ENSMUSG00000019297	Nop9
ENSMUSG00000019312	Grb7
ENSMUSG00000019338	Zfp687
ENSMUSG00000019362	D8Ertd738e
ENSMUSG00000019368	Sec14l4
ENSMUSG00000019370	Calm3
ENSMUSG00000019428	Fkbp8
ENSMUSG00000019433	Gipc1
ENSMUSG00000019437	Tlcd1
ENSMUSG00000019464	Ptger1
ENSMUSG00000019471	Cdc37
ENSMUSG00000019518	Ap4m1
ENSMUSG00000019577	Pdk4
ENSMUSG00000019647	Sema6a
ENSMUSG00000019689	Fmc1
ENSMUSG00000019715	Gle1
ENSMUSG00000019726	Lyst
ENSMUSG00000019731	Slc35e1
ENSMUSG00000019738	Polr2i
ENSMUSG00000019762	Iyd
ENSMUSG00000019763	Rmnd1
ENSMUSG00000019774	Mtrf1l
ENSMUSG00000019790	Stxbp5
ENSMUSG00000019795	Pcmt1
ENSMUSG00000019802	Sec63
ENSMUSG00000019810	Fuca2
ENSMUSG00000019814	Ltv1
ENSMUSG00000019818	Cd164
ENSMUSG00000019822	Smpd2
ENSMUSG00000019826	Zbtb24
ENSMUSG00000019832	Rab32
ENSMUSG00000019837	Gtf3c6
ENSMUSG00000019838	Slc16a10
ENSMUSG00000019843	Fyn
ENSMUSG00000019849	Prep
ENSMUSG00000019857	Asf1a

ENSMUSG00000019864	Rtn4ip1
ENSMUSG00000019866	Crybg1
ENSMUSG00000019877	Serinc1
ENSMUSG00000019880	Rspo3
ENSMUSG00000019883	Echdc1
ENSMUSG00000019897	Ccdc59
ENSMUSG00000019907	Ppp1r12a
ENSMUSG00000019917	10-Sep
ENSMUSG00000019923	Zwint
ENSMUSG00000019935	Slc17a8
ENSMUSG00000019943	Atp2b1
ENSMUSG00000019944	Rhobtb1
ENSMUSG00000019947	Arid5b
ENSMUSG00000019951	Uhrf1bp1l
ENSMUSG00000019952	Poc1b
ENSMUSG00000019960	Dusp6
ENSMUSG00000019969	Psen1
ENSMUSG00000019970	Sgk1
ENSMUSG00000019977	Hbs1l
ENSMUSG00000019978	Epb41l2
ENSMUSG00000019987	Arg1
ENSMUSG00000019996	Map7
ENSMUSG00000019997	Ccn2
ENSMUSG00000020009	Ifngr1
ENSMUSG00000020010	Vnn3
ENSMUSG00000020015	Cdk17
ENSMUSG00000020017	Hal
ENSMUSG00000020019	Ntn4
ENSMUSG00000020021	Fgd6
ENSMUSG00000020023	Tmcc3
ENSMUSG00000020027	Socs2
ENSMUSG00000020029	Nudt4
ENSMUSG00000020048	Hsp90b1
ENSMUSG00000020051	Pah
ENSMUSG00000020053	Igf1
ENSMUSG00000020059	Sycp3
ENSMUSG00000020063	Sirt1
ENSMUSG00000020072	Pbld2
ENSMUSG00000020074	Ccar1
ENSMUSG00000020075	Ddx21
ENSMUSG00000020079	Supv3l1
ENSMUSG00000020085	Aifm2
ENSMUSG00000020087	Tysnd1
ENSMUSG00000020088	Sar1a

ENSMUSG00000020091	Eif4ebp2
ENSMUSG00000020097	Sgpl1
ENSMUSG00000020101	Vsir
ENSMUSG00000020102	Slc16a7
ENSMUSG00000020105	Lrig3
ENSMUSG00000020108	Ddit4
ENSMUSG00000020109	Dnajb12
ENSMUSG00000020114	Cand1
ENSMUSG00000020116	Pno1
ENSMUSG00000020122	Egfr
ENSMUSG00000020123	Avpr1a
ENSMUSG00000020124	Usp15
ENSMUSG00000020128	Vps54
ENSMUSG00000020130	Tbc1d15
ENSMUSG00000020132	Rab21
ENSMUSG00000020133	2310011J03Rik
ENSMUSG00000020134	Peli1
ENSMUSG00000020137	Thap2
ENSMUSG00000020149	Rab1a
ENSMUSG00000020153	Ndufs7
ENSMUSG00000020154	Ptprb
ENSMUSG00000020160	Meis1
ENSMUSG00000020166	Cnot2
ENSMUSG00000020167	Tcf3
ENSMUSG00000020170	Frs2
ENSMUSG00000020171	Yeats4
ENSMUSG00000020173	Cobl
ENSMUSG00000020175	Rab36
ENSMUSG00000020178	Adora2a
ENSMUSG00000020180	Snrpd3
ENSMUSG00000020184	Mdm2
ENSMUSG00000020186	Csrp2
ENSMUSG00000020189	Osbpl8
ENSMUSG00000020190	Mknk2
ENSMUSG00000020198	Ap3d1
ENSMUSG00000020205	Phlda1
ENSMUSG00000020219	Timm13
ENSMUSG00000020220	Vps13d
ENSMUSG00000020224	Llph
ENSMUSG00000020228	Helb
ENSMUSG00000020234	4930404N11Rik
ENSMUSG00000020235	Fzr1
ENSMUSG00000020246	Hcfc2
ENSMUSG00000020250	Txnrd1

ENSMUSG00000020255	D10Wsu102e
ENSMUSG00000020257	Wdr82
ENSMUSG00000020258	Glyctk
ENSMUSG00000020260	Pofut2
ENSMUSG00000020261	Slc36a1
ENSMUSG00000020262	Adarb1
ENSMUSG00000020265	Sumo3
ENSMUSG00000020267	Hint1
ENSMUSG00000020271	Fbxw11
ENSMUSG00000020283	Pex13
ENSMUSG00000020288	Ahsa2
ENSMUSG00000020305	Asb3
ENSMUSG00000020307	Cdc34
ENSMUSG00000020308	Tpgs1
ENSMUSG00000020309	Chac2
ENSMUSG00000020315	Sptbn1
ENSMUSG00000020346	Mgat1
ENSMUSG00000020349	Ppp2ca
ENSMUSG00000020357	Flt4
ENSMUSG00000020358	Hnrnpab
ENSMUSG00000020361	Hspa4
ENSMUSG00000020362	Cnot6
ENSMUSG00000020366	Mapk9
ENSMUSG00000020368	Canx
ENSMUSG00000020375	Rufy1
ENSMUSG00000020380	Rad50
ENSMUSG00000020386	Sar1b
ENSMUSG00000020387	Jade2
ENSMUSG00000020390	Ube2b
ENSMUSG00000020392	Cdkn2aipnl
ENSMUSG00000020393	Kremen1
ENSMUSG00000020397	Med7
ENSMUSG00000020400	Tnip1
ENSMUSG00000020402	Vdac1
ENSMUSG00000020409	Slu7
ENSMUSG00000020422	Tns3
ENSMUSG00000020423	Btg2
ENSMUSG00000020427	Igfbp3
ENSMUSG00000020430	Pes1
ENSMUSG00000020431	Adcy1
ENSMUSG00000020432	Tcn2
ENSMUSG00000020448	Rnf185
ENSMUSG00000020451	Limk2
ENSMUSG00000020454	Eif4enif1

ENSMUSG00000020455	Trim11
ENSMUSG00000020456	Ogdh
ENSMUSG00000020457	Drg1
ENSMUSG00000020458	Rtn4
ENSMUSG00000020459	Mtif2
ENSMUSG00000020462	Cfap36
ENSMUSG00000020463	Ppp4r3b
ENSMUSG00000020472	Zkscan17
ENSMUSG00000020474	Polm
ENSMUSG00000020476	Dbnl
ENSMUSG00000020477	Mrps24
ENSMUSG00000020482	Ccdc117
ENSMUSG00000020483	Dynll2
ENSMUSG00000020484	Xbp1
ENSMUSG00000020495	Smg8
ENSMUSG00000020516	Rps6kb1
ENSMUSG00000020519	Sap30l
ENSMUSG00000020522	Mfap3
ENSMUSG00000020523	Fam114a2
ENSMUSG00000020525	Ppm1d
ENSMUSG00000020526	Znhit3
ENSMUSG00000020530	Ggnbp2
ENSMUSG00000020532	Acaca
ENSMUSG00000020534	Shmt1
ENSMUSG00000020544	Cox11
ENSMUSG00000020553	Pctp
ENSMUSG00000020561	Twistnb
ENSMUSG00000020564	Atxn7l1
ENSMUSG00000020572	Nampt
ENSMUSG00000020580	Rock2
ENSMUSG00000020585	Laptm4a
ENSMUSG00000020590	Snx13
ENSMUSG00000020592	Sdc1
ENSMUSG00000020593	Lpin1
ENSMUSG00000020594	Pum2
ENSMUSG00000020600	Slc7a15
ENSMUSG00000020604	Arsg
ENSMUSG00000020605	Hs1bp3
ENSMUSG00000020608	Smc6
ENSMUSG00000020609	Apob
ENSMUSG00000020610	Amz2
ENSMUSG00000020611	Gna13
ENSMUSG00000020612	Prkar1a
ENSMUSG00000020614	Fam20a

ENSMUSG00000020620	Abca8b
ENSMUSG00000020621	Rdh14
ENSMUSG00000020623	Map2k6
ENSMUSG00000020629	Adi1
ENSMUSG00000020634	Ubxn2a
ENSMUSG00000020638	Cmpk2
ENSMUSG00000020640	Itsn2
ENSMUSG00000020641	Rsad2
ENSMUSG00000020644	Id2
ENSMUSG00000020647	Ncoa1
ENSMUSG00000020653	Klf11
ENSMUSG00000020659	Cbll1
ENSMUSG00000020661	Dnmt3a
ENSMUSG00000020664	Dld
ENSMUSG00000020671	Rab10
ENSMUSG00000020677	Ddx52
ENSMUSG00000020679	Hnf1b
ENSMUSG00000020680	Taf15
ENSMUSG00000020687	Cdc27
ENSMUSG00000020694	Tlk2
ENSMUSG00000020696	Rffl
ENSMUSG00000020697	Lig3
ENSMUSG00000020700	Map3k3
ENSMUSG00000020701	Tmem132e
ENSMUSG00000020705	Ddx42
ENSMUSG00000020706	Ftsj3
ENSMUSG00000020707	Rnf135
ENSMUSG00000020708	Psmc5
ENSMUSG00000020709	Adap2
ENSMUSG00000020715	Ern1
ENSMUSG00000020716	Nf1
ENSMUSG00000020717	Pecam1
ENSMUSG00000020719	Ddx5
ENSMUSG00000020720	Psmd12
ENSMUSG00000020721	Helz
ENSMUSG00000020733	Slc9a3r1
ENSMUSG00000020737	Jpt1
ENSMUSG00000020741	Cluh
ENSMUSG00000020743	Mif4gd
ENSMUSG00000020745	Pafah1b1
ENSMUSG00000020755	Sap30bp
ENSMUSG00000020775	Mrpl38
ENSMUSG00000020777	Acox1
ENSMUSG00000020780	Srp68

ENSMUSG00000020790	Ankfy1
ENSMUSG00000020794	Ube2g1
ENSMUSG00000020802	Ube2o
ENSMUSG00000020803	Txndc17
ENSMUSG00000020806	Rhbdf2
ENSMUSG00000020817	Rabep1
ENSMUSG00000020818	Mfsd11
ENSMUSG00000020821	Kif1c
ENSMUSG00000020823	Sec14l1
ENSMUSG00000020827	Mink1
ENSMUSG00000020828	Pld2
ENSMUSG00000020829	Slc46a1
ENSMUSG00000020831	0610010K14Rik
ENSMUSG00000020832	Eral1
ENSMUSG00000020841	Cpd
ENSMUSG00000020843	Timm22
ENSMUSG00000020849	Ywhae
ENSMUSG00000020859	Spag9
ENSMUSG00000020863	Luc7l3
ENSMUSG00000020864	Ankrd40
ENSMUSG00000020865	Abcc3
ENSMUSG00000020868	Xylt2
ENSMUSG00000020869	Lrrc59
ENSMUSG00000020873	Slc35b1
ENSMUSG00000020883	Fbxl20
ENSMUSG00000020884	Asgr1
ENSMUSG00000020889	Nr1d1
ENSMUSG00000020893	Per1
ENSMUSG00000020900	Myh10
ENSMUSG00000020910	Adprm
ENSMUSG00000020918	Kat2a
ENSMUSG00000020923	Ubtf
ENSMUSG00000020925	Ccdc43
ENSMUSG00000020935	Dcakd
ENSMUSG00000020936	Nmt1
ENSMUSG00000020946	Gosr2
ENSMUSG00000020952	Scfd1
ENSMUSG00000020956	Dtd2
ENSMUSG00000020964	Sel1l
ENSMUSG00000020973	Dnaaf2
ENSMUSG00000020982	Nemf
ENSMUSG00000020988	L2hgdh
ENSMUSG00000020993	Trappc6b
ENSMUSG00000020994	Pnn

ENSMUSG00000021009	Ptpn21
ENSMUSG00000021012	Zc3h14
ENSMUSG00000021024	Psma6
ENSMUSG00000021025	Nfkbia
ENSMUSG00000021036	Sptlc2
ENSMUSG00000021037	Ahsa1
ENSMUSG00000021038	Vipas39
ENSMUSG00000021039	Snw1
ENSMUSG00000021040	Slirp
ENSMUSG00000021051	Ppp2r5e
ENSMUSG00000021054	Sgpp1
ENSMUSG00000021055	Esr2
ENSMUSG00000021067	Sav1
ENSMUSG00000021072	Tmx1
ENSMUSG00000021079	Timm9
ENSMUSG00000021091	Serpina3n
ENSMUSG00000021096	Ppm1a
ENSMUSG00000021097	Clmn
ENSMUSG00000021101	4930408O17Rik
ENSMUSG00000021102	Glrx5
ENSMUSG00000021109	Hif1a
ENSMUSG00000021111	Papola
ENSMUSG00000021112	Mpp5
ENSMUSG00000021114	Atp6v1d
ENSMUSG00000021116	Eif2s1
ENSMUSG00000021124	Vti1b
ENSMUSG00000021127	Zfp36l1
ENSMUSG00000021133	Susd6
ENSMUSG00000021134	Srsf5
ENSMUSG00000021135	Slc10a1
ENSMUSG00000021136	Smoc1
ENSMUSG00000021139	Gm20498
ENSMUSG00000021140	Pcnx
ENSMUSG00000021143	Pacs2
ENSMUSG00000021144	Mta1
ENSMUSG00000021149	Gtpbp4
ENSMUSG00000021156	Zmynd11
ENSMUSG00000021179	Nrde2
ENSMUSG00000021186	Fbln5
ENSMUSG00000021188	Trip11
ENSMUSG00000021189	Atxn3
ENSMUSG00000021192	Golga5
ENSMUSG00000021210	Akr1c6
ENSMUSG00000021211	Akr1c12

ENSMUSG00000021213	Akr1c13
ENSMUSG00000021215	Net1
ENSMUSG00000021228	Acot3
ENSMUSG00000021236	Entpd5
ENSMUSG00000021238	Aldh6a1
ENSMUSG00000021242	Npc2
ENSMUSG00000021248	Tmed10
ENSMUSG00000021250	Fos
ENSMUSG00000021258	Ccnk
ENSMUSG00000021264	Yy1
ENSMUSG00000021271	Zfp839
ENSMUSG00000021273	Fdft1
ENSMUSG00000021275	Tecpr2
ENSMUSG00000021276	Cinp
ENSMUSG00000021277	Traf3
ENSMUSG00000021279	Cdc42bpb
ENSMUSG00000021282	Eif5
ENSMUSG00000021285	Ppp1r13b
ENSMUSG00000021302	Ggps1
ENSMUSG00000021326	Trim27
ENSMUSG00000021336	Slc17a4
ENSMUSG00000021360	Gcnt2
ENSMUSG00000021361	Tmem14c
ENSMUSG00000021364	Elovl2
ENSMUSG00000021365	Nedd9
ENSMUSG00000021366	Hivep1
ENSMUSG00000021374	Nup153
ENSMUSG00000021375	Kif13a
ENSMUSG00000021377	Dek
ENSMUSG00000021391	Cenpp
ENSMUSG00000021392	Nol8
ENSMUSG00000021395	Spin1
ENSMUSG00000021400	Wrnip1
ENSMUSG00000021408	Ripk1
ENSMUSG00000021411	Pxdc1
ENSMUSG00000021413	Prpf4b
ENSMUSG00000021417	Eci2
ENSMUSG00000021427	Ssr1
ENSMUSG00000021431	Snrnp48
ENSMUSG00000021432	Slc35b3
ENSMUSG00000021453	Gadd45g
ENSMUSG00000021457	Syk
ENSMUSG00000021458	Aopep
ENSMUSG00000021460	Auh

ENSMUSG00000021466	Ptch1
ENSMUSG00000021468	Sptlc1
ENSMUSG00000021470	Ercc6l2
ENSMUSG00000021476	Habp4
ENSMUSG00000021477	Ctsl
ENSMUSG00000021484	Lman2
ENSMUSG00000021486	Prelid1
ENSMUSG00000021488	Nsd1
ENSMUSG00000021495	Fam193b
ENSMUSG00000021496	Pcbd2
ENSMUSG00000021500	Ddx46
ENSMUSG00000021501	Caml
ENSMUSG00000021504	B4galt7
ENSMUSG00000021509	Slc25a48
ENSMUSG00000021518	Ptdss1
ENSMUSG00000021537	Cetn3
ENSMUSG00000021539	Lect2
ENSMUSG00000021540	Smad5
ENSMUSG00000021546	Hnrnpk
ENSMUSG00000021549	Rasa1
ENSMUSG00000021550	2210016F16Rik
ENSMUSG00000021559	Dapk1
ENSMUSG00000021573	Tppp
ENSMUSG00000021577	Sdha
ENSMUSG00000021578	Ccdc127
ENSMUSG00000021579	Lrrc14b
ENSMUSG00000021595	Nsun2
ENSMUSG00000021596	Mctp1
ENSMUSG00000021606	Ndufs6
ENSMUSG00000021610	Clptm1l
ENSMUSG00000021620	Acot12
ENSMUSG00000021621	Zcchc9
ENSMUSG00000021636	Marveld2
ENSMUSG00000021638	Ocln
ENSMUSG00000021650	Ptcd2
ENSMUSG00000021666	Gfm2
ENSMUSG00000021670	Hmgcr
ENSMUSG00000021676	Iqgap2
ENSMUSG00000021681	Aggf1
ENSMUSG00000021686	Ap3b1
ENSMUSG00000021687	Scamp1
ENSMUSG00000021690	Jmy
ENSMUSG00000021701	Plk2
ENSMUSG00000021709	Erbin

ENSMUSG00000021710	Nln
ENSMUSG00000021711	Trappc13
ENSMUSG00000021716	Srek1ip1
ENSMUSG00000021737	Psmd6
ENSMUSG00000021738	Atxn7
ENSMUSG00000021745	Ptprg
ENSMUSG00000021751	Acox2
ENSMUSG00000021752	Kctd6
ENSMUSG00000021754	Map3k1
ENSMUSG00000021756	Il6st
ENSMUSG00000021759	Plpp1
ENSMUSG00000021764	Ndufs4
ENSMUSG00000021767	Kat6b
ENSMUSG00000021770	Samd8
ENSMUSG00000021772	Nkiras1
ENSMUSG00000021775	Nr1d2
ENSMUSG00000021779	Thrb
ENSMUSG00000021786	Oxsm
ENSMUSG00000021796	Bmpr1a
ENSMUSG00000021807	Rtraf
ENSMUSG00000021809	Nudt13
ENSMUSG00000021816	Ppp3cb
ENSMUSG00000021819	Zswim8
ENSMUSG00000021820	Camk2g
ENSMUSG00000021830	Txndc16
ENSMUSG00000021832	Psmc6
ENSMUSG00000021835	Bmp4
ENSMUSG00000021840	Mapk1ip1l
ENSMUSG00000021846	Peli2
ENSMUSG00000021868	Ppif
ENSMUSG00000021870	Slmap
ENSMUSG00000021871	Gm49342
ENSMUSG00000021876	Rnase4
ENSMUSG00000021884	Hacl1
ENSMUSG00000021886	Gpr65
ENSMUSG00000021891	Mettl6
ENSMUSG00000021892	Sh3bp5
ENSMUSG00000021893	Capn7
ENSMUSG00000021895	Arhgef3
ENSMUSG00000021900	Btd
ENSMUSG00000021902	Phf7
ENSMUSG00000021905	Dph3
ENSMUSG00000021910	Nisch
ENSMUSG00000021916	Glt8d1

ENSMUSG00000021917	Spcs1
ENSMUSG00000021922	Itih4
ENSMUSG00000021929	Kpna3
ENSMUSG00000021939	Ctsb
ENSMUSG00000021943	Gdf10
ENSMUSG00000021944	Gata4
ENSMUSG00000021945	Zmym2
ENSMUSG00000021959	Lats2
ENSMUSG00000021962	Dcp1a
ENSMUSG00000021967	Mrpl57
ENSMUSG00000021969	Zdhhc20
ENSMUSG00000021973	Micu2
ENSMUSG00000021978	Extl3
ENSMUSG00000021982	Cdadc1
ENSMUSG00000021983	Atp8a2
ENSMUSG00000021990	Spata13
ENSMUSG00000021998	Lcp1
ENSMUSG00000022000	Zc3h13
ENSMUSG00000022009	Nufip1
ENSMUSG00000022010	Tsc22d1
ENSMUSG00000022013	Dnajc15
ENSMUSG00000022016	Akap11
ENSMUSG00000022019	Tdrd3
ENSMUSG00000022023	Wbp4
ENSMUSG00000022025	Cnmd
ENSMUSG00000022032	Scara5
ENSMUSG00000022035	Ccdc25
ENSMUSG00000022037	Clu
ENSMUSG00000022051	Bnip3l
ENSMUSG00000022052	Ppp2r2a
ENSMUSG00000022075	Rhobtb2
ENSMUSG00000022089	Bin3
ENSMUSG00000022091	Sorbs3
ENSMUSG00000022095	Fam160b2
ENSMUSG00000022105	Rb1
ENSMUSG00000022108	Itm2b
ENSMUSG00000022124	Fbxl3
ENSMUSG00000022125	Cln5
ENSMUSG00000022131	Gpr180
ENSMUSG00000022136	Dnajc3
ENSMUSG00000022139	Mbnl2
ENSMUSG00000022141	Nipbl
ENSMUSG00000022149	C9
ENSMUSG00000022150	Dab2

ENSMUSG00000022151	Ttc33
ENSMUSG00000022175	Lrp10
ENSMUSG00000022177	Haus4
ENSMUSG00000022181	C6
ENSMUSG00000022184	Fbxo4
ENSMUSG00000022185	Acin1
ENSMUSG00000022191	Drosha
ENSMUSG00000022194	Pabpn1
ENSMUSG00000022195	6030458C11Rik
ENSMUSG00000022200	Golph3
ENSMUSG00000022201	Zfr
ENSMUSG00000022205	Sub1
ENSMUSG00000022210	Dhrs4
ENSMUSG00000022216	Psme1
ENSMUSG00000022218	Tgm1
ENSMUSG00000022219	Cideb
ENSMUSG00000022228	Zscan26
ENSMUSG00000022237	Ankrd33b
ENSMUSG00000022241	Tars
ENSMUSG00000022244	Amacr
ENSMUSG00000022246	Rai14
ENSMUSG00000022247	Brix1
ENSMUSG00000022253	Nadk2
ENSMUSG00000022255	Mtdh
ENSMUSG00000022261	Sdc2
ENSMUSG00000022263	Trio
ENSMUSG00000022265	Ank
ENSMUSG00000022270	Retreg1
ENSMUSG00000022272	Myo10
ENSMUSG00000022280	Rnf19a
ENSMUSG00000022283	Pabpc1
ENSMUSG00000022297	Fzd6
ENSMUSG00000022299	Slc25a32
ENSMUSG00000022304	Dpys
ENSMUSG00000022305	Lrp12
ENSMUSG00000022307	Oxr1
ENSMUSG00000022312	Eif3h
ENSMUSG00000022313	Utp23
ENSMUSG00000022314	Rad21
ENSMUSG00000022323	Rida
ENSMUSG00000022338	Eny2
ENSMUSG00000022339	Ebag9
ENSMUSG00000022346	Myc
ENSMUSG00000022351	Sqle

ENSMUSG00000022353	Mtss1
ENSMUSG00000022354	Ndufb9
ENSMUSG00000022358	Fbxo32
ENSMUSG00000022361	Zhx1
ENSMUSG00000022362	Gm29394
ENSMUSG00000022365	Derl1
ENSMUSG00000022370	Mrpl13
ENSMUSG00000022377	Asap1
ENSMUSG00000022383	Ppara
ENSMUSG00000022387	Brd1
ENSMUSG00000022389	Tef
ENSMUSG00000022391	Rangap1
ENSMUSG00000022394	L3mbtl2
ENSMUSG00000022401	Xpnpep3
ENSMUSG00000022403	St13
ENSMUSG00000022412	Mief1
ENSMUSG00000022419	Deptor
ENSMUSG00000022420	Dnal4
ENSMUSG00000022426	Josd1
ENSMUSG00000022437	Samm50
ENSMUSG00000022443	Myh9
ENSMUSG00000022451	Twf1
ENSMUSG00000022453	Naga
ENSMUSG00000022462	Slc38a2
ENSMUSG00000022464	Slc38a4
ENSMUSG00000022472	Desi1
ENSMUSG00000022498	Txndc11
ENSMUSG00000022507	1810013L24Rik
ENSMUSG00000022512	Cldn1
ENSMUSG00000022514	Il1rap
ENSMUSG00000022516	Nudt16l1
ENSMUSG00000022517	Mgrn1
ENSMUSG00000022521	Crebbp
ENSMUSG00000022528	Hes1
ENSMUSG00000022529	Zfp263
ENSMUSG00000022538	Lsg1
ENSMUSG00000022545	Ercc4
ENSMUSG00000022551	Cyc1
ENSMUSG00000022554	Hgh1
ENSMUSG00000022559	Fbxl6
ENSMUSG00000022560	Slc52a2
ENSMUSG00000022564	Grina
ENSMUSG00000022565	Plec
ENSMUSG00000022579	Gpihbp1

ENSMUSG00000022587	Ly6e
ENSMUSG00000022601	Zbtb11
ENSMUSG00000022610	Mapk12
ENSMUSG00000022615	Tymp
ENSMUSG00000022620	Arsa
ENSMUSG00000022623	Shank3
ENSMUSG00000022629	Kif21a
ENSMUSG00000022634	Yaf2
ENSMUSG00000022636	Alcam
ENSMUSG00000022637	Cblb
ENSMUSG00000022656	Nectin3
ENSMUSG00000022663	Atg3
ENSMUSG00000022664	Slc35a5
ENSMUSG00000022665	Ccdc80
ENSMUSG00000022671	Mzt2
ENSMUSG00000022678	Nde1
ENSMUSG00000022679	Mpv17l
ENSMUSG00000022682	Rrn3
ENSMUSG00000022684	Bfar
ENSMUSG00000022698	Naa50
ENSMUSG00000022702	Hira
ENSMUSG00000022704	Qtrt2
ENSMUSG00000022706	Mrpl40
ENSMUSG00000022708	Zbtb20
ENSMUSG00000022710	Usp7
ENSMUSG00000022721	Trmt2a
ENSMUSG00000022748	Cmss1
ENSMUSG00000022752	Tomm70a
ENSMUSG00000022757	Tfg
ENSMUSG00000022760	Thap7
ENSMUSG00000022765	Snap29
ENSMUSG00000022766	Serpind1
ENSMUSG00000022769	Sdf2l1
ENSMUSG00000022770	Dlg1
ENSMUSG00000022772	Senp5
ENSMUSG00000022781	Pak2
ENSMUSG00000022787	Wdr53
ENSMUSG00000022791	Tnk2
ENSMUSG00000022799	Arhgap31
ENSMUSG00000022800	Fyttd1
ENSMUSG00000022801	Lrch3
ENSMUSG00000022807	Osbpl11
ENSMUSG00000022809	Nr1i2
ENSMUSG00000022811	Zfp148

ENSMUSG00000022812	Gsk3b
ENSMUSG00000022814	Umps
ENSMUSG00000022817	Itgb5
ENSMUSG00000022821	Hgd
ENSMUSG00000022828	Gtf2e1
ENSMUSG00000022836	Mylk
ENSMUSG00000022847	Thpo
ENSMUSG00000022848	Slc49a4
ENSMUSG00000022853	Ehhadh
ENSMUSG00000022856	Tmem41a
ENSMUSG00000022864	D16Ertd472e
ENSMUSG00000022867	Usp25
ENSMUSG00000022868	Ahsg
ENSMUSG00000022875	Kng1
ENSMUSG00000022877	Hrg
ENSMUSG00000022884	Eif4a2
ENSMUSG00000022885	St6gal1
ENSMUSG00000022887	Masp1
ENSMUSG00000022890	Atp5j
ENSMUSG00000022892	App
ENSMUSG00000022893	Adamts1
ENSMUSG00000022895	Ets2
ENSMUSG00000022897	Dyrk1a
ENSMUSG00000022898	Vps26c
ENSMUSG00000022906	Parp9
ENSMUSG00000022911	Arl13b
ENSMUSG00000022912	Pros1
ENSMUSG00000022914	Brwd1
ENSMUSG00000022946	Dop1b
ENSMUSG00000022951	Rcan1
ENSMUSG00000022957	Itsn1
ENSMUSG00000022961	Son
ENSMUSG00000022965	Ifngr2
ENSMUSG00000022967	Ifnar1
ENSMUSG00000022969	Il10rb
ENSMUSG00000022971	Ifnar2
ENSMUSG00000022972	Cfap298
ENSMUSG00000022973	Synj1
ENSMUSG00000022974	Paxbp1
ENSMUSG00000022982	Sod1
ENSMUSG00000022983	Scaf4
ENSMUSG00000022992	Kansl2
ENSMUSG00000022994	Adcy6
ENSMUSG00000022999	Lmbr1l

ENSMUSG00000023010	Tmbim6
ENSMUSG00000023019	Gpd1
ENSMUSG00000023020	Cox14
ENSMUSG00000023022	Lima1
ENSMUSG00000023025	Larp4
ENSMUSG00000023027	Atf1
ENSMUSG00000023030	Slc11a2
ENSMUSG00000023034	Nr4a1
ENSMUSG00000023043	Krt18
ENSMUSG00000023044	Csad
ENSMUSG00000023045	Soat2
ENSMUSG00000023048	Prr13
ENSMUSG00000023057	Fabp2
ENSMUSG00000023067	Cdkn1a
ENSMUSG00000023068	Nus1
ENSMUSG00000023070	Rgn
ENSMUSG00000023073	Slc10a2
ENSMUSG00000023075	Akirin1
ENSMUSG00000023087	Noct
ENSMUSG00000023089	Ndufa5
ENSMUSG00000023094	Msrb2
ENSMUSG00000023104	Rfc2
ENSMUSG00000023110	Prmt5
ENSMUSG00000023143	Nagpa
ENSMUSG00000023150	Ivns1abp
ENSMUSG00000023156	Rpp14
ENSMUSG00000023175	Bsg
ENSMUSG00000023176	Cpn2
ENSMUSG00000023191	P3h3
ENSMUSG00000023224	Serping1
ENSMUSG00000023243	Kcnk5
ENSMUSG00000023262	Acy1
ENSMUSG00000023272	Creld2
ENSMUSG00000023286	Ube2j2
ENSMUSG00000023307	5-Mar
ENSMUSG00000023353	Agap3
ENSMUSG00000023452	Pisd
ENSMUSG00000023460	Rab12
ENSMUSG00000023467	Tulp2
ENSMUSG00000023723	Mrps23
ENSMUSG00000023800	Tiam2
ENSMUSG00000023805	Synj2
ENSMUSG00000023829	Slc22a1
ENSMUSG00000023830	Igf2r

ENSMUSG00000023852	Chd1
ENSMUSG00000023904	Hcfc1r1
ENSMUSG00000023915	Tnfrsf21
ENSMUSG00000023923	Tbc1d5
ENSMUSG00000023932	Cdc5l
ENSMUSG00000023938	Aars2
ENSMUSG00000023939	Mrpl14
ENSMUSG00000023942	Slc29a1
ENSMUSG00000023944	Hsp90ab1
ENSMUSG00000023951	Vegfa
ENSMUSG00000023952	Gtpbp2
ENSMUSG00000023960	Enpp5
ENSMUSG00000023961	Enpp4
ENSMUSG00000023965	Fbxl17
ENSMUSG00000023973	Cnpy3
ENSMUSG00000023977	Ubr2
ENSMUSG00000023980	Taf8
ENSMUSG00000023984	Gm20517
ENSMUSG00000023988	Bysl
ENSMUSG00000023990	Tfeb
ENSMUSG00000023991	Foxp4
ENSMUSG00000023994	Nfya
ENSMUSG00000024002	Brd4
ENSMUSG00000024006	Stk38
ENSMUSG00000024007	Ppil1
ENSMUSG00000024014	Pim1
ENSMUSG00000024026	Glo1
ENSMUSG00000024030	Abcg1
ENSMUSG00000024038	Ndufv3
ENSMUSG00000024039	Cbs
ENSMUSG00000024042	Sik1
ENSMUSG00000024045	Akap8
ENSMUSG00000024052	Lpin2
ENSMUSG00000024054	Smchd1
ENSMUSG00000024065	Ehd3
ENSMUSG00000024066	Xdh
ENSMUSG00000024069	Slc30a6
ENSMUSG00000024070	Prkd3
ENSMUSG00000024074	Crim1
ENSMUSG00000024079	Eif2ak2
ENSMUSG00000024081	Cebpz
ENSMUSG00000024082	Ndufaf7
ENSMUSG00000024085	Man2a1
ENSMUSG00000024096	Ralbp1

ENSMUSG00000024098	Twsg1
ENSMUSG00000024101	Washc1
ENSMUSG00000024104	Washc2
ENSMUSG00000024118	Tedc2
ENSMUSG00000024120	Lrpprc
ENSMUSG00000024127	Prepl
ENSMUSG00000024130	Abca3
ENSMUSG00000024131	Slc3a1
ENSMUSG00000024132	Eci1
ENSMUSG00000024135	Srbd1
ENSMUSG00000024137	E4f1
ENSMUSG00000024140	Epas1
ENSMUSG00000024142	Mlst8
ENSMUSG00000024143	Rhoq
ENSMUSG00000024145	Pigf
ENSMUSG00000024150	Mcfd2
ENSMUSG00000024160	Spsb3
ENSMUSG00000024163	Mapk8ip3
ENSMUSG00000024164	C3
ENSMUSG00000024168	Tmem204
ENSMUSG00000024169	Ift140
ENSMUSG00000024174	Pot1b
ENSMUSG00000024180	Tmem8
ENSMUSG00000024182	Axin1
ENSMUSG00000024190	Dusp1
ENSMUSG00000024201	Kdm4b
ENSMUSG00000024212	Mllt1
ENSMUSG00000024213	Nudt3
ENSMUSG00000024218	Taf11
ENSMUSG00000024219	Anks1
ENSMUSG00000024222	Fkbp5
ENSMUSG00000024228	Nudt12
ENSMUSG00000024231	Cul2
ENSMUSG00000024232	Bambi
ENSMUSG00000024238	Zeb1
ENSMUSG00000024240	Epc1
ENSMUSG00000024241	Sos1
ENSMUSG00000024247	Pkdcc
ENSMUSG00000024248	Cox7a2l
ENSMUSG00000024254	Abcg8
ENSMUSG00000024259	Slc25a46
ENSMUSG00000024273	2700062C07Rik
ENSMUSG00000024276	Zfp397
ENSMUSG00000024277	Mapre2

ENSMUSG00000024283	Wac
ENSMUSG00000024286	Ccny
ENSMUSG00000024287	Thoc1
ENSMUSG00000024290	Rock1
ENSMUSG00000024292	Cyp4f14
ENSMUSG00000024293	Esco1
ENSMUSG00000024298	Zfp871
ENSMUSG00000024304	Cdh2
ENSMUSG00000024308	Tapbp
ENSMUSG00000024309	Pfdn6
ENSMUSG00000024325	Ring1
ENSMUSG00000024327	Slc39a7
ENSMUSG00000024331	Dsc2
ENSMUSG00000024335	Brd2
ENSMUSG00000024339	Tap2
ENSMUSG00000024346	Pfdn1
ENSMUSG00000024354	Slc23a1
ENSMUSG00000024357	Sil1
ENSMUSG00000024359	Hspa9
ENSMUSG00000024360	Etf1
ENSMUSG00000024378	Stard4
ENSMUSG00000024384	Iws1
ENSMUSG00000024386	Proc
ENSMUSG00000024387	Csnk2b
ENSMUSG00000024391	Apom
ENSMUSG00000024393	Prrc2a
ENSMUSG00000024397	Aif1
ENSMUSG00000024400	Wdr33
ENSMUSG00000024414	Mrpl27
ENSMUSG00000024422	Dhx16
ENSMUSG00000024424	Ttc39c
ENSMUSG00000024427	Spry4
ENSMUSG00000024429	Gnl1
ENSMUSG00000024430	Cabyr
ENSMUSG00000024431	Nr3c1
ENSMUSG00000024440	Pcdh12
ENSMUSG00000024446	Rpp21
ENSMUSG00000024451	Arap3
ENSMUSG00000024456	Diaph1
ENSMUSG00000024474	Ik
ENSMUSG00000024477	Pggt1b
ENSMUSG00000024479	Mal2
ENSMUSG00000024483	Ankhd1
ENSMUSG00000024487	Yipf5

ENSMUSG00000024507	Hsd17b4
ENSMUSG00000024513	Mbd2
ENSMUSG00000024515	Smad4
ENSMUSG00000024524	Gnal
ENSMUSG00000024528	Srfbp1
ENSMUSG00000024535	Snx24
ENSMUSG00000024544	Ldlrad4
ENSMUSG00000024560	Cxxc1
ENSMUSG00000024561	Mbd1
ENSMUSG00000024563	Smad2
ENSMUSG00000024566	Atp9b
ENSMUSG00000024570	Rbfa
ENSMUSG00000024571	Gm16286
ENSMUSG00000024576	Csnk1a1
ENSMUSG00000024580	Grpel2
ENSMUSG00000024581	Napg
ENSMUSG00000024587	Nars
ENSMUSG00000024588	Fech
ENSMUSG00000024589	Nedd4l
ENSMUSG00000024592	C330018D20Rik
ENSMUSG00000024594	Prrc1
ENSMUSG00000024610	Cd74
ENSMUSG00000024620	Pdgfrb
ENSMUSG00000024621	Csf1r
ENSMUSG00000024622	Hmgxb3
ENSMUSG00000024646	Cyb5a
ENSMUSG00000024661	Fth1
ENSMUSG00000024663	Rab3il1
ENSMUSG00000024665	Fads2
ENSMUSG00000024683	Mrpl16
ENSMUSG00000024687	Osbp
ENSMUSG00000024695	Zfp91
ENSMUSG00000024712	Rfk
ENSMUSG00000024732	Ccdc86
ENSMUSG00000024737	Slc15a3
ENSMUSG00000024747	Aldh1a7
ENSMUSG00000024750	Zfand5
ENSMUSG00000024754	Cemip2
ENSMUSG00000024759	Atl3
ENSMUSG00000024772	Ehd1
ENSMUSG00000024773	Atg2a
ENSMUSG00000024778	Fas
ENSMUSG00000024780	Cdc37l1
ENSMUSG00000024785	Rcl1

ENSMUSG00000024789	Jak2
ENSMUSG00000024790	Sac3d1
ENSMUSG00000024799	Tm7sf2
ENSMUSG00000024805	Pcgf5
ENSMUSG00000024807	Syvn1
ENSMUSG00000024812	Tjp2
ENSMUSG00000024816	Frmd8
ENSMUSG00000024827	Gldc
ENSMUSG00000024830	Rps6kb2
ENSMUSG00000024835	Coro1b
ENSMUSG00000024841	Eif1ad
ENSMUSG00000024843	Chka
ENSMUSG00000024844	Banf1
ENSMUSG00000024853	Sf3b2
ENSMUSG00000024856	Cdk2ap2
ENSMUSG00000024863	Mbl2
ENSMUSG00000024866	Acy3
ENSMUSG00000024867	Pip5k1b
ENSMUSG00000024892	Pcx
ENSMUSG00000024896	Minpp1
ENSMUSG00000024900	Cpt1a
ENSMUSG00000024902	Mrpl11
ENSMUSG00000024913	Lrp5
ENSMUSG00000024914	Drap1
ENSMUSG00000024921	Smarca2
ENSMUSG00000024925	Rnaseh2c
ENSMUSG00000024927	Rela
ENSMUSG00000024937	Ehbp1l1
ENSMUSG00000024941	Scyl1
ENSMUSG00000024947	Men1
ENSMUSG00000024949	Sf1
ENSMUSG00000024953	Prdx5
ENSMUSG00000024959	Bad
ENSMUSG00000024960	Plcb3
ENSMUSG00000024969	Mark2
ENSMUSG00000024975	Pdcd4
ENSMUSG00000024976	Shoc2
ENSMUSG00000024985	Tcf7l2
ENSMUSG00000024986	Hhex
ENSMUSG00000024987	Cyp26a1
ENSMUSG00000024991	Eif3a
ENSMUSG00000024998	Plce1
ENSMUSG00000024999	Noc3l
ENSMUSG00000025006	Sorbs1

ENSMUSG00000025017	Pik3ap1
ENSMUSG00000025025	Mxi1
ENSMUSG00000025034	Trim8
ENSMUSG00000025036	Sfxn2
ENSMUSG00000025040	Fundc1
ENSMUSG00000025041	Nt5c2
ENSMUSG00000025044	Msr1
ENSMUSG00000025047	Pdcd11
ENSMUSG00000025059	Gk
ENSMUSG00000025060	Slk
ENSMUSG00000025066	Sfr1
ENSMUSG00000025068	Gsto1
ENSMUSG00000025075	Habp2
ENSMUSG00000025076	Casp7
ENSMUSG00000025078	Nhlrc2
ENSMUSG00000025085	Ablim1
ENSMUSG00000025086	Trub1
ENSMUSG00000025089	Gfra1
ENSMUSG00000025127	Gcgr
ENSMUSG00000025130	P4hb
ENSMUSG00000025132	Arhgdia
ENSMUSG00000025137	Pcyt2
ENSMUSG00000025138	Sirt7
ENSMUSG00000025139	Tollip
ENSMUSG00000025142	Aspscr1
ENSMUSG00000025144	Cenpx
ENSMUSG00000025153	Fasn
ENSMUSG00000025156	Gps1
ENSMUSG00000025157	Zdhhc16
ENSMUSG00000025158	Rfng
ENSMUSG00000025159	Mms19
ENSMUSG00000025162	Csnk1d
ENSMUSG00000025171	Ubtd1
ENSMUSG00000025176	Hoga1
ENSMUSG00000025178	Pi4k2a
ENSMUSG00000025184	R3hcc1l
ENSMUSG00000025190	Got1
ENSMUSG00000025193	Cutc
ENSMUSG00000025194	Abcc2
ENSMUSG00000025195	Dnmbp
ENSMUSG00000025197	Cyp2c23
ENSMUSG00000025198	Erlin1
ENSMUSG00000025200	Cwf19l1
ENSMUSG00000025201	Bloc1s2-ps

ENSMUSG00000025204	Ndufb8
ENSMUSG00000025207	Sema4g
ENSMUSG00000025208	Mrpl43
ENSMUSG00000025209	Twnk
ENSMUSG00000025218	Poll
ENSMUSG00000025220	Oga
ENSMUSG00000025223	Ldb1
ENSMUSG00000025224	Gbf1
ENSMUSG00000025226	Fbxl15
ENSMUSG00000025228	Actr1a
ENSMUSG00000025234	Arih1
ENSMUSG00000025236	Adpgk
ENSMUSG00000025239	Limd1
ENSMUSG00000025246	Tbl1x
ENSMUSG00000025255	Zfhx4
ENSMUSG00000025260	Hsd17b10
ENSMUSG00000025261	Huwe1
ENSMUSG00000025269	Apex2
ENSMUSG00000025277	Abhd6
ENSMUSG00000025278	Flnb
ENSMUSG00000025279	Dnase1l3
ENSMUSG00000025280	Polr3a
ENSMUSG00000025314	Ptprj
ENSMUSG00000025316	Banp
ENSMUSG00000025326	Ube3a
ENSMUSG00000025332	Kdm5c
ENSMUSG00000025337	Sbds
ENSMUSG00000025340	Rabgef1
ENSMUSG00000025347	Mettl7b
ENSMUSG00000025354	Dnajc14
ENSMUSG00000025355	Mmp19
ENSMUSG00000025364	Pa2g4
ENSMUSG00000025369	Smarcc2
ENSMUSG00000025371	Chmp6
ENSMUSG00000025373	Rnf41
ENSMUSG00000025374	Nabp2
ENSMUSG00000025377	Tepsin
ENSMUSG00000025381	Cnpy2
ENSMUSG00000025396	Hsd17b6
ENSMUSG00000025402	Nab2
ENSMUSG00000025404	R3hdm2
ENSMUSG00000025405	Inhbc
ENSMUSG00000025410	Dctn2
ENSMUSG00000025413	Ttc4

ENSMUSG00000025417	Pip4k2c
ENSMUSG00000025421	Hdhd2
ENSMUSG00000025423	Pias2
ENSMUSG00000025437	Usp33
ENSMUSG00000025451	Paip1
ENSMUSG00000025453	Nnt
ENSMUSG00000025470	Zfp511
ENSMUSG00000025479	Cyp2e1
ENSMUSG00000025484	Bet1l
ENSMUSG00000025487	Psmd13
ENSMUSG00000025492	Ifitm3
ENSMUSG00000025495	Ptdss2
ENSMUSG00000025499	Hras
ENSMUSG00000025504	Eps8l2
ENSMUSG00000025509	Pnpla2
ENSMUSG00000025510	Cd151
ENSMUSG00000025512	Chid1
ENSMUSG00000025532	Crcp
ENSMUSG00000025533	Asl
ENSMUSG00000025534	Gusb
ENSMUSG00000025538	Sumf2
ENSMUSG00000025571	Tnrc6c
ENSMUSG00000025574	Tk1
ENSMUSG00000025575	Cant1
ENSMUSG00000025577	Cbx2
ENSMUSG00000025578	Cbx8
ENSMUSG00000025583	Rptor
ENSMUSG00000025608	Podxl
ENSMUSG00000025609	Mkln1
ENSMUSG00000025612	Bach1
ENSMUSG00000025613	Cct8
ENSMUSG00000025616	Usp16
ENSMUSG00000025645	Ccdc51
ENSMUSG00000025646	Atrip
ENSMUSG00000025647	Shisa5
ENSMUSG00000025702	8-Mar
ENSMUSG00000025722	Wdr73
ENSMUSG00000025726	Slc28a1
ENSMUSG00000025730	Rab40c
ENSMUSG00000025736	Jmjd8
ENSMUSG00000025743	Sdc3
ENSMUSG00000025745	Hadha
ENSMUSG00000025757	Hspa4l
ENSMUSG00000025764	Jade1

ENSMUSG00000025780	Itih5
ENSMUSG00000025782	Taf3
ENSMUSG00000025786	Zdhhc3
ENSMUSG00000025791	Pgm1
ENSMUSG00000025792	Slc25a10
ENSMUSG00000025795	Rassf3
ENSMUSG00000025809	Itgb1
ENSMUSG00000025812	Pard3
ENSMUSG00000025815	Dhtkd1
ENSMUSG00000025821	Zfp282
ENSMUSG00000025823	Pdia4
ENSMUSG00000025856	Pdgfa
ENSMUSG00000025858	Get4
ENSMUSG00000025860	Xiap
ENSMUSG00000025868	Higd2a
ENSMUSG00000025869	Nop16
ENSMUSG00000025872	Thoc3
ENSMUSG00000025873	Faf2
ENSMUSG00000025878	Uimc1
ENSMUSG00000025880	Smad7
ENSMUSG00000025885	Myo5b
ENSMUSG00000025902	Sox17
ENSMUSG00000025907	Rb1cc1
ENSMUSG00000025917	Cops5
ENSMUSG00000025921	Rdh10
ENSMUSG00000025936	Gm4956
ENSMUSG00000025937	Lactb2
ENSMUSG00000025940	Tmem70
ENSMUSG00000025950	Idh1
ENSMUSG00000025956	Mettl21a
ENSMUSG00000025958	Creb1
ENSMUSG00000025962	Fastkd2
ENSMUSG00000025964	Adam23
ENSMUSG00000025967	Eef1b2
ENSMUSG00000025969	Nrp2
ENSMUSG00000025980	Hspd1
ENSMUSG00000026003	Acadl
ENSMUSG00000026004	Kansl1l
ENSMUSG00000026014	Raph1
ENSMUSG00000026020	Nop58
ENSMUSG00000026021	Sumo1
ENSMUSG00000026024	Als2
ENSMUSG00000026028	Trak2
ENSMUSG00000026029	Casp8

ENSMUSG00000026031	Cflar
ENSMUSG00000026032	Ndufb3
ENSMUSG00000026034	Clk1
ENSMUSG00000026036	Nif3l1
ENSMUSG00000026048	Ercc5
ENSMUSG00000026064	Ptp4a1
ENSMUSG00000026072	Il1r1
ENSMUSG00000026078	Pdcl3
ENSMUSG00000026082	Rev1
ENSMUSG00000026083	Eif5b
ENSMUSG00000026088	Mitd1
ENSMUSG00000026095	Asnsd1
ENSMUSG00000026102	Inpp1
ENSMUSG00000026104	Stat1
ENSMUSG00000026111	Unc50
ENSMUSG00000026112	Coa5
ENSMUSG00000026116	Tmem131
ENSMUSG00000026121	Sema4c
ENSMUSG00000026131	Dst
ENSMUSG00000026135	Zfp142
ENSMUSG00000026142	Rhbdd1
ENSMUSG00000026153	Fam135a
ENSMUSG00000026155	Smap1
ENSMUSG00000026158	Ogfrl1
ENSMUSG00000026163	Sphkap
ENSMUSG00000026170	Cyp27a1
ENSMUSG00000026171	Rnf25
ENSMUSG00000026173	Plcd4
ENSMUSG00000026174	Cnot9
ENSMUSG00000026176	Ctdsp1
ENSMUSG00000026177	Slc11a1
ENSMUSG00000026185	Igfbp5
ENSMUSG00000026193	Fn1
ENSMUSG00000026197	Zfand2b
ENSMUSG00000026199	Ankzf1
ENSMUSG00000026201	Stk16
ENSMUSG00000026202	Tuba4a
ENSMUSG00000026219	Trip12
ENSMUSG00000026227	2810459M11Rik
ENSMUSG00000026229	Psmd1
ENSMUSG00000026234	Ncl
ENSMUSG00000026238	Ptma
ENSMUSG00000026239	Pde6d
ENSMUSG00000026248	Mrpl44

ENSMUSG00000026254	Eif4e2
ENSMUSG00000026260	Ndufa10
ENSMUSG00000026269	Rnpepl1
ENSMUSG00000026272	Agxt
ENSMUSG00000026273	Mterf4
ENSMUSG00000026275	Ppp1r7
ENSMUSG00000026277	Stk25
ENSMUSG00000026280	Atg4b
ENSMUSG00000026283	Ing5
ENSMUSG00000026288	Inpp5d
ENSMUSG00000026295	Spp2
ENSMUSG00000026304	Rab17
ENSMUSG00000026305	Lrrfip1
ENSMUSG00000026317	Cln8
ENSMUSG00000026333	Gin1
ENSMUSG00000026339	Ccdc93
ENSMUSG00000026343	Gpr39
ENSMUSG00000026344	Lypd1
ENSMUSG00000026348	Acmsd
ENSMUSG00000026349	Ccnt2
ENSMUSG00000026353	Ubxn4
ENSMUSG00000026356	Dars
ENSMUSG00000026358	Rgs1
ENSMUSG00000026360	Rgs2
ENSMUSG00000026365	Cfh
ENSMUSG00000026377	Nifk
ENSMUSG00000026383	Epb41l5
ENSMUSG00000026389	Steap3
ENSMUSG00000026398	Nr5a2
ENSMUSG00000026405	C4bp
ENSMUSG00000026409	Pfkfb2
ENSMUSG00000026417	Pigr
ENSMUSG00000026425	Srgap2
ENSMUSG00000026434	Nucks1
ENSMUSG00000026435	Slc45a3
ENSMUSG00000026436	Elk4
ENSMUSG00000026439	Rbbp5
ENSMUSG00000026457	Adipor1
ENSMUSG00000026466	Tor1aip1
ENSMUSG00000026469	Xpr1
ENSMUSG00000026470	Stx6
ENSMUSG00000026473	Glul
ENSMUSG00000026475	Rgs16
ENSMUSG00000026478	Lamc1

ENSMUSG00000026482	Rgl1
ENSMUSG00000026489	Coq8a
ENSMUSG00000026490	Cdc42bpa
ENSMUSG00000026491	Ahctf1
ENSMUSG00000026492	Tfb2m
ENSMUSG00000026510	Trp53bp2
ENSMUSG00000026519	Tmem63a
ENSMUSG00000026542	Apcs
ENSMUSG00000026544	Dusp23
ENSMUSG00000026553	Copa
ENSMUSG00000026554	Dcaf8
ENSMUSG00000026558	Uck2
ENSMUSG00000026568	Mpc2
ENSMUSG00000026571	Dcaf6
ENSMUSG00000026576	Atp1b1
ENSMUSG00000026579	F5
ENSMUSG00000026580	Selp
ENSMUSG00000026584	Scyl3
ENSMUSG00000026589	Sec16b
ENSMUSG00000026594	Ralgps2
ENSMUSG00000026596	Abl2
ENSMUSG00000026608	Kctd3
ENSMUSG00000026614	Slc30a10
ENSMUSG00000026615	Eprs
ENSMUSG00000026618	Iars2
ENSMUSG00000026621	1-Mar
ENSMUSG00000026623	Lpgat1
ENSMUSG00000026628	Atf3
ENSMUSG00000026638	Irf6
ENSMUSG00000026639	Lamb3
ENSMUSG00000026640	Plxna2
ENSMUSG00000026641	Usf1
ENSMUSG00000026648	Dclre1c
ENSMUSG00000026655	Fam107b
ENSMUSG00000026656	Fcgr2b
ENSMUSG00000026657	Frmd4a
ENSMUSG00000026662	Sephs1
ENSMUSG00000026663	Atf6
ENSMUSG00000026664	Phyh
ENSMUSG00000026669	Mcm10
ENSMUSG00000026670	Uap1
ENSMUSG00000026672	Optn
ENSMUSG00000026675	Hsd17b7
ENSMUSG00000026692	Fmo4

ENSMUSG00000026694	Eef1aknmt
ENSMUSG00000026698	Pigc
ENSMUSG00000026705	Klhl20
ENSMUSG00000026708	Cenpl
ENSMUSG00000026712	Mrc1
ENSMUSG00000026715	Serpinc1
ENSMUSG00000026718	Stam
ENSMUSG00000026721	Rabgap1l
ENSMUSG00000026729	4930562F07Rik
ENSMUSG00000026730	Pter
ENSMUSG00000026737	Pip4k2a
ENSMUSG00000026739	Bmi1
ENSMUSG00000026740	Dnajc1
ENSMUSG00000026749	Nek6
ENSMUSG00000026750	Psmb7
ENSMUSG00000026753	Ppp6c
ENSMUSG00000026755	Arpc5l
ENSMUSG00000026768	Itga8
ENSMUSG00000026775	Yme1l1
ENSMUSG00000026781	Acbd5
ENSMUSG00000026788	Zbtb43
ENSMUSG00000026796	Fam129b
ENSMUSG00000026810	Dpm2
ENSMUSG00000026812	Tsc1
ENSMUSG00000026814	Eng
ENSMUSG00000026833	Olfm1
ENSMUSG00000026836	Acvr1
ENSMUSG00000026837	Col5a1
ENSMUSG00000026842	Abl1
ENSMUSG00000026848	Tor1b
ENSMUSG00000026849	Tor1a
ENSMUSG00000026853	Crat
ENSMUSG00000026856	Dolpp1
ENSMUSG00000026857	Ntmt1
ENSMUSG00000026864	Hspa5
ENSMUSG00000026866	Kynu
ENSMUSG00000026867	Gapvd1
ENSMUSG00000026869	Psmd5
ENSMUSG00000026870	Cutal
ENSMUSG00000026872	Zeb2
ENSMUSG00000026874	Hc
ENSMUSG00000026878	Rab14
ENSMUSG00000026883	Dab2ip
ENSMUSG00000026887	Mrrf

ENSMUSG00000026895	Ndufa8
ENSMUSG00000026915	Strbp
ENSMUSG00000026918	Brd3
ENSMUSG00000026922	Agpat2
ENSMUSG00000026923	Notch1
ENSMUSG00000026924	Sec16a
ENSMUSG00000026927	Entr1
ENSMUSG00000026932	Nacc2
ENSMUSG00000026933	Camsap1
ENSMUSG00000026938	Fcna
ENSMUSG00000026942	Traf2
ENSMUSG00000026965	Anapc2
ENSMUSG00000026966	Ssna1
ENSMUSG00000026970	Rbms1
ENSMUSG00000026974	Zmynd19
ENSMUSG00000026975	Dph7
ENSMUSG00000026977	7-Mar
ENSMUSG00000026986	Hnmt
ENSMUSG00000026991	Pkp4
ENSMUSG00000027002	Nckap1
ENSMUSG00000027007	Itprid2
ENSMUSG00000027014	Cwc22
ENSMUSG00000027016	Zfp385b
ENSMUSG00000027048	Abcb11
ENSMUSG00000027067	Ssrp1
ENSMUSG00000027074	Slc43a3
ENSMUSG00000027079	Clp1
ENSMUSG00000027080	Med19
ENSMUSG00000027082	Tfpi
ENSMUSG00000027088	Phospho2
ENSMUSG00000027091	Zc3h15
ENSMUSG00000027109	Sp3
ENSMUSG00000027122	Arl14ep
ENSMUSG00000027131	Emc4
ENSMUSG00000027132	Katnbl1
ENSMUSG00000027164	Traf6
ENSMUSG00000027173	Depdc7
ENSMUSG00000027176	Cstf3
ENSMUSG00000027177	Hipk3
ENSMUSG00000027180	Fbxo3
ENSMUSG00000027185	Nat10
ENSMUSG00000027188	Pamr1
ENSMUSG00000027189	Trim44
ENSMUSG00000027193	Api5

ENSMUSG00000027200	Sema6d
ENSMUSG00000027206	Cops2
ENSMUSG00000027227	Sord
ENSMUSG00000027233	Patl2
ENSMUSG00000027243	Harbi1
ENSMUSG00000027244	Atg13
ENSMUSG00000027245	Hypk
ENSMUSG00000027248	Pdia3
ENSMUSG00000027249	F2
ENSMUSG00000027253	Lrp4
ENSMUSG00000027255	Arfgap2
ENSMUSG00000027257	Pacsin3
ENSMUSG00000027261	Hao1
ENSMUSG00000027274	Mkks
ENSMUSG00000027276	Jag1
ENSMUSG00000027284	Cdan1
ENSMUSG00000027288	Zfp106
ENSMUSG00000027291	Vps39
ENSMUSG00000027293	Ehd4
ENSMUSG00000027300	Ubox5
ENSMUSG00000027304	Rtf1
ENSMUSG00000027305	Ndufaf1
ENSMUSG00000027312	Atrn
ENSMUSG00000027313	Chac1
ENSMUSG00000027314	Dll4
ENSMUSG00000027322	Siglec1
ENSMUSG00000027332	Ivd
ENSMUSG00000027342	Pcna
ENSMUSG00000027346	Gpcpd1
ENSMUSG00000027351	Spred1
ENSMUSG00000027357	Crls1
ENSMUSG00000027358	Bmp2
ENSMUSG00000027359	Slc27a2
ENSMUSG00000027361	Gabpb1
ENSMUSG00000027363	Usp8
ENSMUSG00000027364	Usp50
ENSMUSG00000027367	Stard7
ENSMUSG00000027374	Mrps5
ENSMUSG00000027394	Ttl
ENSMUSG00000027395	Polr1b
ENSMUSG00000027397	Slc20a1
ENSMUSG00000027398	Il1b
ENSMUSG00000027404	Snrpb
ENSMUSG00000027422	Rrbp1

ENSMUSG00000027424	Mgme1
ENSMUSG00000027425	Kat14
ENSMUSG00000027427	Polr3f
ENSMUSG00000027428	Rbbp9
ENSMUSG00000027439	Gzf1
ENSMUSG00000027447	Cst3
ENSMUSG00000027455	Nsfl1c
ENSMUSG00000027459	Fam110a
ENSMUSG00000027465	Tbc1d20
ENSMUSG00000027474	Ccm2l
ENSMUSG00000027479	Mapre1
ENSMUSG00000027495	Fam210b
ENSMUSG00000027498	Cstf1
ENSMUSG00000027502	Rtf2
ENSMUSG00000027508	Pag1
ENSMUSG00000027509	Rae1
ENSMUSG00000027513	Pck1
ENSMUSG00000027519	Rab22a
ENSMUSG00000027531	Impa1
ENSMUSG00000027540	Ptpn1
ENSMUSG00000027546	Atp9a
ENSMUSG00000027551	Zfp64
ENSMUSG00000027559	Car3
ENSMUSG00000027573	Gid8
ENSMUSG00000027575	Arfgap1
ENSMUSG00000027580	Helz2
ENSMUSG00000027582	Zgpat
ENSMUSG00000027589	Pcmtd2
ENSMUSG00000027593	Raly
ENSMUSG00000027598	Itch
ENSMUSG00000027599	Armc1
ENSMUSG00000027602	Map1lc3a
ENSMUSG00000027612	Mmp24
ENSMUSG00000027613	Eif6
ENSMUSG00000027618	Nfs1
ENSMUSG00000027620	Rbm39
ENSMUSG00000027628	Aar2
ENSMUSG00000027636	Sla2
ENSMUSG00000027637	Rab5if
ENSMUSG00000027650	Tti1
ENSMUSG00000027652	Ralgapb
ENSMUSG00000027660	Skil
ENSMUSG00000027665	Pik3ca
ENSMUSG00000027677	Ttc14

ENSMUSG00000027678	Ncoa3
ENSMUSG00000027690	Slc2a2
ENSMUSG00000027694	Gm8325
ENSMUSG00000027695	Pld1
ENSMUSG00000027698	Nceh1
ENSMUSG00000027706	Sec62
ENSMUSG00000027708	Dcun1d1
ENSMUSG00000027710	Acad9
ENSMUSG00000027719	Adad1
ENSMUSG00000027739	Rab33b
ENSMUSG00000027746	Ufm1
ENSMUSG00000027761	Aadac
ENSMUSG00000027762	Sucnr1
ENSMUSG00000027763	Mbnl1
ENSMUSG00000027765	P2ry1
ENSMUSG00000027770	Dhx36
ENSMUSG00000027774	Gfm1
ENSMUSG00000027777	Schip1
ENSMUSG00000027782	Kpna4
ENSMUSG00000027792	Bche
ENSMUSG00000027796	Smad9
ENSMUSG00000027806	Tsc22d2
ENSMUSG00000027809	Etfdh
ENSMUSG00000027822	Slc33a1
ENSMUSG00000027829	Ccnl1
ENSMUSG00000027845	Dclre1b
ENSMUSG00000027848	Olfml3
ENSMUSG00000027859	Ngf
ENSMUSG00000027860	Vangl1
ENSMUSG00000027865	Gdap2
ENSMUSG00000027867	Spag17
ENSMUSG00000027875	Hmgcs2
ENSMUSG00000027878	Notch2
ENSMUSG00000027883	Gpsm2
ENSMUSG00000027884	Clcc1
ENSMUSG00000027893	Ahcyl1
ENSMUSG00000027900	Dram2
ENSMUSG00000027905	Ddx20
ENSMUSG00000027931	Npr1
ENSMUSG00000027933	Ints3
ENSMUSG00000027935	Rab13
ENSMUSG00000027936	Crtc2
ENSMUSG00000027937	Jtb
ENSMUSG00000027947	Il6ra

ENSMUSG00000027951	Adar
ENSMUSG00000027952	Pmvk
ENSMUSG00000027954	Efna1
ENSMUSG00000027961	Lrrc39
ENSMUSG00000027963	Extl2
ENSMUSG00000027984	Hadh
ENSMUSG00000027993	Trim2
ENSMUSG00000027995	Tlr2
ENSMUSG00000028001	Fga
ENSMUSG00000028003	Lrat
ENSMUSG00000028011	Tdo2
ENSMUSG00000028015	Ctso
ENSMUSG00000028019	Pdgfc
ENSMUSG00000028024	Enpep
ENSMUSG00000028030	Tbck
ENSMUSG00000028039	Efna3
ENSMUSG00000028042	Zbtb7b
ENSMUSG00000028051	Hcn3
ENSMUSG00000028053	Ash1l
ENSMUSG00000028057	Rit1
ENSMUSG00000028060	Khdc4
ENSMUSG00000028062	Lamtor2
ENSMUSG00000028064	Sema4a
ENSMUSG00000028069	Gpatch4
ENSMUSG00000028073	Pear1
ENSMUSG00000028082	Sh3d19
ENSMUSG00000028085	Gatb
ENSMUSG00000028086	Fbxw7
ENSMUSG00000028088	Fmo5
ENSMUSG00000028096	Gpr89
ENSMUSG00000028102	Pex11b
ENSMUSG00000028104	Polr3gl
ENSMUSG00000028106	Rprd2
ENSMUSG00000028108	Ecm1
ENSMUSG00000028121	Bcar3
ENSMUSG00000028126	Pip5k1a
ENSMUSG00000028127	Abcd3
ENSMUSG00000028132	Tmem56
ENSMUSG00000028138	Adh5
ENSMUSG00000028140	Mrpl9
ENSMUSG00000028150	Rorc
ENSMUSG00000028156	Eif4e
ENSMUSG00000028161	Ppp3ca
ENSMUSG00000028164	Manba

ENSMUSG00000028179	Cth
ENSMUSG00000028180	Zranb2
ENSMUSG00000028184	Adgrl2
ENSMUSG00000028185	Dnase2b
ENSMUSG00000028187	Rpf1
ENSMUSG00000028195	Ccn1
ENSMUSG00000028207	Asph
ENSMUSG00000028211	Trp53inp1
ENSMUSG00000028224	Nbn
ENSMUSG00000028240	Cyp7a1
ENSMUSG00000028247	Coq3
ENSMUSG00000028248	Pnisr
ENSMUSG00000028251	Tstd3
ENSMUSG00000028261	Ndufaf4
ENSMUSG00000028266	Lmo4
ENSMUSG00000028274	Rngtt
ENSMUSG00000028277	Ube2j1
ENSMUSG00000028284	Map3k7
ENSMUSG00000028291	Akirin2
ENSMUSG00000028293	Slc35a1
ENSMUSG00000028307	Aldob
ENSMUSG00000028309	Rnf20
ENSMUSG00000028329	Xpa
ENSMUSG00000028333	Anp32b
ENSMUSG00000028343	Erp44
ENSMUSG00000028356	Ambp
ENSMUSG00000028367	Txn1
ENSMUSG00000028381	Ugcg
ENSMUSG00000028382	Ptbp3
ENSMUSG00000028385	Snx30
ENSMUSG00000028393	Alad
ENSMUSG00000028394	Pole3
ENSMUSG00000028399	Ptprd
ENSMUSG00000028410	Dnaja1
ENSMUSG00000028412	Slc44a1
ENSMUSG00000028413	B4galt1
ENSMUSG00000028416	Bag1
ENSMUSG00000028419	Chmp5
ENSMUSG00000028420	Tmem38b
ENSMUSG00000028423	Nfx1
ENSMUSG00000028426	Rad23b
ENSMUSG00000028430	Nol6
ENSMUSG00000028433	Ubap2
ENSMUSG00000028434	Epb41l4b

ENSMUSG00000028437	Ubap1
ENSMUSG00000028443	Nudt2
ENSMUSG00000028445	Enho
ENSMUSG00000028447	Dctn3
ENSMUSG00000028452	Vcp
ENSMUSG00000028454	Pigo
ENSMUSG00000028458	Tesk1
ENSMUSG00000028459	Cd72
ENSMUSG00000028461	Ccdc107
ENSMUSG00000028465	Tln1
ENSMUSG00000028467	Gba2
ENSMUSG00000028469	Npr2
ENSMUSG00000028478	Clta
ENSMUSG00000028479	Gne
ENSMUSG00000028484	Psip1
ENSMUSG00000028514	Usp24
ENSMUSG00000028517	Plpp3
ENSMUSG00000028522	Mier1
ENSMUSG00000028525	Pde4b
ENSMUSG00000028530	Jak1
ENSMUSG00000028536	2610528J11Rik
ENSMUSG00000028538	St3gal3
ENSMUSG00000028542	Slc6a9
ENSMUSG00000028550	Atg4c
ENSMUSG00000028552	Eps15
ENSMUSG00000028553	Angptl3
ENSMUSG00000028556	Dock7
ENSMUSG00000028557	Rnf11
ENSMUSG00000028559	Osbpl9
ENSMUSG00000028560	Usp1
ENSMUSG00000028565	Nfia
ENSMUSG00000028567	Txndc12
ENSMUSG00000028577	Plaa
ENSMUSG00000028578	Caap1
ENSMUSG00000028582	Cc2d1b
ENSMUSG00000028599	Tnfrsf1b
ENSMUSG00000028603	Scp2
ENSMUSG00000028607	Cpt2
ENSMUSG00000028608	Czib
ENSMUSG00000028609	Magoh
ENSMUSG00000028614	Ndc1
ENSMUSG00000028617	Lrrc42
ENSMUSG00000028619	Tceanc2
ENSMUSG00000028621	Cyb5rl

ENSMUSG00000028630	Dyrk2
ENSMUSG00000028636	Ppcs
ENSMUSG00000028639	Ybx1
ENSMUSG00000028641	P3h1
ENSMUSG00000028643	Svbp
ENSMUSG00000028645	Slc2a1
ENSMUSG00000028646	Rragc
ENSMUSG00000028649	Macf1
ENSMUSG00000028653	Trit1
ENSMUSG00000028654	Mycl
ENSMUSG00000028655	Mfsd2a
ENSMUSG00000028656	Cap1
ENSMUSG00000028668	Eloa
ENSMUSG00000028673	Fuca1
ENSMUSG00000028675	Pnrc2
ENSMUSG00000028677	Rnf220
ENSMUSG00000028684	Urod
ENSMUSG00000028688	Toe1
ENSMUSG00000028703	Lrrc41
ENSMUSG00000028709	Mob3c
ENSMUSG00000028715	Cyp4a14
ENSMUSG00000028729	Ebna1bp2
ENSMUSG00000028737	Aldh4a1
ENSMUSG00000028741	Mrto4
ENSMUSG00000028744	Pqlc2
ENSMUSG00000028745	Capzb
ENSMUSG00000028755	Cda
ENSMUSG00000028756	Pink1
ENSMUSG00000028757	Ddost
ENSMUSG00000028759	Hp1bp3
ENSMUSG00000028760	Eif4g3
ENSMUSG00000028763	Hspg2
ENSMUSG00000028766	Alpl
ENSMUSG00000028771	Ptpn12
ENSMUSG00000028776	Tinagl1
ENSMUSG00000028779	Pef1
ENSMUSG00000028788	Ptp4a2
ENSMUSG00000028792	Ak2
ENSMUSG00000028793	Rnf19b
ENSMUSG00000028796	Phc2
ENSMUSG00000028797	Tmem234
ENSMUSG00000028799	Zfp362
ENSMUSG00000028809	Srrm1
ENSMUSG00000028811	Yars

ENSMUSG00000028820	Sfpq
ENSMUSG00000028821	Syf2
ENSMUSG00000028822	Tmem50a
ENSMUSG00000028826	Maco1
ENSMUSG00000028847	Trappc3
ENSMUSG00000028848	Gpn2
ENSMUSG00000028849	Map7d1
ENSMUSG00000028850	Gpatch3
ENSMUSG00000028851	Nudc
ENSMUSG00000028868	Wasf2
ENSMUSG00000028869	Gnl2
ENSMUSG00000028882	Ppp1r8
ENSMUSG00000028890	Mtf1
ENSMUSG00000028893	Sesn2
ENSMUSG00000028899	Taf12
ENSMUSG00000028901	Gmeb1
ENSMUSG00000028911	Srsf4
ENSMUSG00000028914	Casp9
ENSMUSG00000028917	Plekhm2
ENSMUSG00000028919	Arhgef19
ENSMUSG00000028920	Fbxo42
ENSMUSG00000028936	Rpl22
ENSMUSG00000028944	Prkag2
ENSMUSG00000028945	Rheb
ENSMUSG00000028948	Nol9
ENSMUSG00000028949	Smarcd3
ENSMUSG00000028952	Zbtb48
ENSMUSG00000028953	Abcf2
ENSMUSG00000028954	Nub1
ENSMUSG00000028958	Tmub1
ENSMUSG00000028960	Ube4b
ENSMUSG00000028964	Park7
ENSMUSG00000028967	Errfi1
ENSMUSG00000028974	Dffa
ENSMUSG00000028975	Pex14
ENSMUSG00000028979	Masp2
ENSMUSG00000028980	H6pd
ENSMUSG00000028982	Slc25a33
ENSMUSG00000028988	Ctnnbip1
ENSMUSG00000028991	Mtor
ENSMUSG00000028992	Nmnat1
ENSMUSG00000028998	Tomm7
ENSMUSG00000028999	Rint1
ENSMUSG00000029004	Kmt2e

ENSMUSG00000029009	Mthfr
ENSMUSG00000029020	Mfn2
ENSMUSG00000029028	Lrrc47
ENSMUSG00000029036	Atad3a
ENSMUSG00000029038	Ssu72
ENSMUSG00000029048	Rer1
ENSMUSG00000029050	Ski
ENSMUSG00000029059	Prxl2b
ENSMUSG00000029062	Cdk11b
ENSMUSG00000029063	Nadk
ENSMUSG00000029064	Gnb1
ENSMUSG00000029066	Mrpl20
ENSMUSG00000029068	Ccnl2
ENSMUSG00000029071	Dvl1
ENSMUSG00000029073	Cptp
ENSMUSG00000029076	Sdf4
ENSMUSG00000029090	Adgra3
ENSMUSG00000029098	Acox3
ENSMUSG00000029103	Lrpap1
ENSMUSG00000029110	Rnf4
ENSMUSG00000029119	Man2b2
ENSMUSG00000029125	Stx18
ENSMUSG00000029131	Dnajb6
ENSMUSG00000029135	Fosl2
ENSMUSG00000029145	Eif2b4
ENSMUSG00000029147	Ppm1g
ENSMUSG00000029153	Ociad2
ENSMUSG00000029162	Khk
ENSMUSG00000029163	Emilin1
ENSMUSG00000029165	Agbl5
ENSMUSG00000029167	Ppargc1a
ENSMUSG00000029169	Dhx15
ENSMUSG00000029173	Sepsecs
ENSMUSG00000029174	Tbc1d1
ENSMUSG00000029176	Anapc4
ENSMUSG00000029178	Klf3
ENSMUSG00000029185	Fam114a1
ENSMUSG00000029195	Klb
ENSMUSG00000029196	Tada2b
ENSMUSG00000029198	Grpel1
ENSMUSG00000029199	Lias
ENSMUSG00000029202	Pds5a
ENSMUSG00000029203	Ube2k
ENSMUSG00000029207	Apbb2

ENSMUSG00000029213	Commd8
ENSMUSG00000029217	Tec
ENSMUSG00000029221	Slc30a9
ENSMUSG00000029228	Lnx1
ENSMUSG00000029231	Pdgfra
ENSMUSG00000029238	Clock
ENSMUSG00000029246	Ppat
ENSMUSG00000029247	Paics
ENSMUSG00000029249	Rest
ENSMUSG00000029260	Ugt2b34
ENSMUSG00000029263	Pigg
ENSMUSG00000029265	Dr1
ENSMUSG00000029267	Mtf2
ENSMUSG00000029270	Dipk1a
ENSMUSG00000029287	Tgfbr3
ENSMUSG00000029290	Zfp326
ENSMUSG00000029304	Spp1
ENSMUSG00000029309	Sparcl1
ENSMUSG00000029310	Nudt9
ENSMUSG00000029313	Aff1
ENSMUSG00000029328	Hnrnpdl
ENSMUSG00000029338	Antxr2
ENSMUSG00000029344	Tpst2
ENSMUSG00000029345	Tfip11
ENSMUSG00000029346	Srrd
ENSMUSG00000029369	Afm
ENSMUSG00000029385	Ccng2
ENSMUSG00000029388	Eif2b1
ENSMUSG00000029392	Rilpl1
ENSMUSG00000029394	Cdk2ap1
ENSMUSG00000029397	Rchy1
ENSMUSG00000029402	Snrnp35
ENSMUSG00000029404	Arl6ip4
ENSMUSG00000029405	G3bp2
ENSMUSG00000029406	Pitpnm2
ENSMUSG00000029407	Uso1
ENSMUSG00000029422	Rsrc2
ENSMUSG00000029427	Zcchc8
ENSMUSG00000029428	Stx2
ENSMUSG00000029434	Vps33a
ENSMUSG00000029438	Bcl7a
ENSMUSG00000029439	Sfswap
ENSMUSG00000029440	Psmd9
ENSMUSG00000029445	Hpd

ENSMUSG00000029449	Rhof
ENSMUSG00000029454	Mapkapk5
ENSMUSG00000029455	Aldh2
ENSMUSG00000029458	Brap
ENSMUSG00000029461	Fam168a
ENSMUSG00000029464	Gpn3
ENSMUSG00000029465	Arpc3
ENSMUSG00000029467	Atp2a2
ENSMUSG00000029472	Anapc5
ENSMUSG00000029474	Rnf34
ENSMUSG00000029478	Ncor2
ENSMUSG00000029480	Dhx37
ENSMUSG00000029482	Aacs
ENSMUSG00000029499	Pxmp2
ENSMUSG00000029501	Ankle2
ENSMUSG00000029504	Ddx51
ENSMUSG00000029505	Ep400
ENSMUSG00000029507	Pus1
ENSMUSG00000029512	Ulk1
ENSMUSG00000029513	Prkab1
ENSMUSG00000029518	Rab35
ENSMUSG00000029524	Sirt4
ENSMUSG00000029528	Pxn
ENSMUSG00000029535	Triap1
ENSMUSG00000029536	Gatc
ENSMUSG00000029545	Acads
ENSMUSG00000029550	Sppl3
ENSMUSG00000029556	Hnf1a
ENSMUSG00000029557	Mrm2
ENSMUSG00000029559	2210016L21Rik
ENSMUSG00000029561	Oasl2
ENSMUSG00000029569	Tmem168
ENSMUSG00000029571	Tmem106b
ENSMUSG00000029575	Mmab
ENSMUSG00000029578	Wipi2
ENSMUSG00000029580	Actb
ENSMUSG00000029587	Zfp12
ENSMUSG00000029592	Usp30
ENSMUSG00000029594	Rbm19
ENSMUSG00000029599	Ddx54
ENSMUSG00000029613	Eif2ak1
ENSMUSG00000029616	Erp29
ENSMUSG00000029622	Arpc1b
ENSMUSG00000029623	Pdap1

ENSMUSG00000029624	Ptcd1
ENSMUSG00000029625	Cpsf4
ENSMUSG00000029630	Cyp3a25
ENSMUSG00000029632	Ndufa4
ENSMUSG00000029634	Rnf6
ENSMUSG00000029640	Usp12
ENSMUSG00000029642	Polr1d
ENSMUSG00000029647	Pan3
ENSMUSG00000029648	Flt1
ENSMUSG00000029649	Pomp
ENSMUSG00000029650	Slc46a3
ENSMUSG00000029651	Mtus2
ENSMUSG00000029655	N4bp2l2
ENSMUSG00000029656	C8b
ENSMUSG00000029661	Col1a2
ENSMUSG00000029669	Tspan12
ENSMUSG00000029670	Ing3
ENSMUSG00000029672	Fam3c
ENSMUSG00000029681	Bcl7b
ENSMUSG00000029684	Wasl
ENSMUSG00000029686	Cul1
ENSMUSG00000029695	Aass
ENSMUSG00000029701	Rbm28
ENSMUSG00000029708	Gcc1
ENSMUSG00000029710	Ephb4
ENSMUSG00000029711	Epo
ENSMUSG00000029714	Gigyf1
ENSMUSG00000029715	Pop7
ENSMUSG00000029716	Tfr2
ENSMUSG00000029720	Gm20605
ENSMUSG00000029722	Agfg2
ENSMUSG00000029723	Tsc22d4
ENSMUSG00000029726	Mepce
ENSMUSG00000029727	Cyp3a13
ENSMUSG00000029729	Zkscan1
ENSMUSG00000029761	Cald1
ENSMUSG00000029763	Exoc4
ENSMUSG00000029767	Calu
ENSMUSG00000029771	Irf5
ENSMUSG00000029775	Klhdc10
ENSMUSG00000029776	Hibadh
ENSMUSG00000029781	Fkbp9
ENSMUSG00000029810	Tmem176b
ENSMUSG00000029823	Luc7l2

ENSMUSG00000029833	Trim24
ENSMUSG00000029864	Gstk1
ENSMUSG00000029917	C130060K24Rik
ENSMUSG00000029920	Smarcad1
ENSMUSG00000029922	Mkrn1
ENSMUSG00000029924	Slc37a3
ENSMUSG00000029998	Pcyox1
ENSMUSG00000029999	Tgfa
ENSMUSG00000030002	Dusp11
ENSMUSG00000030008	Pradc1
ENSMUSG00000030016	Zfp638
ENSMUSG00000030019	Fbxl14
ENSMUSG00000030029	Lrig1
ENSMUSG00000030032	Wdr54
ENSMUSG00000030035	Wbp1
ENSMUSG00000030036	Mogs
ENSMUSG00000030037	Mrpl53
ENSMUSG00000030042	Pole4
ENSMUSG00000030045	Mrpl19
ENSMUSG00000030046	Bmp10
ENSMUSG00000030047	Arhgap25
ENSMUSG00000030055	Rab43
ENSMUSG00000030056	Isy1
ENSMUSG00000030057	Cnbp
ENSMUSG00000030059	Tmf1
ENSMUSG00000030061	Uba3
ENSMUSG00000030064	Frmd4b
ENSMUSG00000030067	Foxp1
ENSMUSG00000030083	Abtb1
ENSMUSG00000030087	Klf15
ENSMUSG00000030088	Aldh1l1
ENSMUSG00000030091	Nup210
ENSMUSG00000030094	Xpc
ENSMUSG00000030095	Tmem43
ENSMUSG00000030101	Sumf1
ENSMUSG00000030103	Bhlhe40
ENSMUSG00000030109	Slc6a12
ENSMUSG00000030122	Ptms
ENSMUSG00000030123	Plxnd1
ENSMUSG00000030126	Tmcc1
ENSMUSG00000030131	Mug2
ENSMUSG00000030138	Bms1
ENSMUSG00000030159	Clec1b
ENSMUSG00000030161	Gabarapl1

ENSMUSG00000030166	Rad52
ENSMUSG00000030168	Adipor2
ENSMUSG00000030170	Wnt5b
ENSMUSG00000030172	Erc1
ENSMUSG00000030180	Kdm5a
ENSMUSG00000030189	Ybx3
ENSMUSG00000030199	Etv6
ENSMUSG00000030201	Lrp6
ENSMUSG00000030203	Dusp16
ENSMUSG00000030204	Ddx47
ENSMUSG00000030213	Atf7ip
ENSMUSG00000030216	Wbp11
ENSMUSG00000030217	Art4
ENSMUSG00000030224	Strap
ENSMUSG00000030231	Plekha5
ENSMUSG00000030232	Aebp2
ENSMUSG00000030236	Slco1b2
ENSMUSG00000030243	Recql
ENSMUSG00000030247	Kcnj8
ENSMUSG00000030256	Bhlhe41
ENSMUSG00000030259	Rassf8
ENSMUSG00000030265	Kras
ENSMUSG00000030278	Cidec
ENSMUSG00000030287	Itpr2
ENSMUSG00000030291	Med21
ENSMUSG00000030301	Ccdc91
ENSMUSG00000030304	Ergic2
ENSMUSG00000030306	Tmtc1
ENSMUSG00000030313	Dennd5b
ENSMUSG00000030315	Vgll4
ENSMUSG00000030335	Mrpl51
ENSMUSG00000030339	Ltbr
ENSMUSG00000030340	Scnn1a
ENSMUSG00000030341	Tnfrsf1a
ENSMUSG00000030352	Tspan9
ENSMUSG00000030359	Pzp
ENSMUSG00000030364	Clec2h
ENSMUSG00000030378	Sult2a8
ENSMUSG00000030397	Mark4
ENSMUSG00000030400	Ercc2
ENSMUSG00000030407	Qpctl
ENSMUSG00000030409	Dmpk
ENSMUSG00000030410	Dmwd
ENSMUSG00000030421	Uri1

ENSMUSG00000030423	Pop4
ENSMUSG00000030431	Tmem238
ENSMUSG00000030435	U2af2
ENSMUSG00000030451	Herc2
ENSMUSG00000030452	Nipa2
ENSMUSG00000030471	Zdhhc13
ENSMUSG00000030483	Cyp2b10
ENSMUSG00000030494	Rhpn2
ENSMUSG00000030499	Kctd15
ENSMUSG00000030509	Asb7
ENSMUSG00000030513	Pcsk6
ENSMUSG00000030516	Tjp1
ENSMUSG00000030518	Fam189a1
ENSMUSG00000030522	Mtmr10
ENSMUSG00000030527	Crtc3
ENSMUSG00000030530	Furin
ENSMUSG00000030533	Unc45a
ENSMUSG00000030536	Iqgap1
ENSMUSG00000030538	Cib1
ENSMUSG00000030539	Sema4b
ENSMUSG00000030541	Idh2
ENSMUSG00000030545	Pex11a
ENSMUSG00000030551	Nr2f2
ENSMUSG00000030555	Ttc23
ENSMUSG00000030557	Mef2a
ENSMUSG00000030602	Pak4
ENSMUSG00000030603	Psmc4
ENSMUSG00000030609	Aen
ENSMUSG00000030611	Mrps11
ENSMUSG00000030612	Mrpl46
ENSMUSG00000030615	Tmem126a
ENSMUSG00000030619	Eed
ENSMUSG00000030623	Prss23os
ENSMUSG00000030629	Zfand6
ENSMUSG00000030630	Fah
ENSMUSG00000030643	Rab30
ENSMUSG00000030647	Ndufc2
ENSMUSG00000030653	Gm45837
ENSMUSG00000030655	Smg1
ENSMUSG00000030660	Pik3c2a
ENSMUSG00000030662	Ipo5
ENSMUSG00000030663	1110004F10Rik
ENSMUSG00000030670	Cyp2r1
ENSMUSG00000030671	Pde3b

ENSMUSG00000030674	Qprt
ENSMUSG00000030678	Maz
ENSMUSG00000030680	Pagr1a
ENSMUSG00000030688	Stard10
ENSMUSG00000030691	Fchsd2
ENSMUSG00000030697	Ppp4c
ENSMUSG00000030711	Sult1a1
ENSMUSG00000030714	Sgf29
ENSMUSG00000030718	Ppme1
ENSMUSG00000030725	Lipt2
ENSMUSG00000030727	Rabep2
ENSMUSG00000030733	Sh2b1
ENSMUSG00000030737	Slco2b1
ENSMUSG00000030738	Eif3c
ENSMUSG00000030747	Dgat2
ENSMUSG00000030748	Il4ra
ENSMUSG00000030753	Thap12
ENSMUSG00000030760	Acer3
ENSMUSG00000030761	Myo7a
ENSMUSG00000030781	Slc5a2
ENSMUSG00000030787	Lyve1
ENSMUSG00000030790	Adm
ENSMUSG00000030795	Fus
ENSMUSG00000030800	Prss8
ENSMUSG00000030801	Kat8
ENSMUSG00000030802	Bckdk
ENSMUSG00000030805	Stx4a
ENSMUSG00000030815	Phkg2
ENSMUSG00000030816	Rnf40
ENSMUSG00000030824	Nucb1
ENSMUSG00000030826	Bcat2
ENSMUSG00000030830	Itgal
ENSMUSG00000030846	Tial1
ENSMUSG00000030847	Bag3
ENSMUSG00000030849	Fgfr2
ENSMUSG00000030852	Tacc2
ENSMUSG00000030861	Acadsb
ENSMUSG00000030868	Dctn5
ENSMUSG00000030870	Ubfd1
ENSMUSG00000030871	Ears2
ENSMUSG00000030876	Mettl9
ENSMUSG00000030879	Mrpl17
ENSMUSG00000030880	Polr3e
ENSMUSG00000030894	Tpp1

ENSMUSG00000030909	Anks4b
ENSMUSG00000030929	Eri2
ENSMUSG00000030930	Chst15
ENSMUSG00000030934	Oat
ENSMUSG00000030935	Acsm3
ENSMUSG00000030942	Thumpd1
ENSMUSG00000030946	Lhpp
ENSMUSG00000030956	Fam53b
ENSMUSG00000030965	Abraxas2
ENSMUSG00000030966	Trim21
ENSMUSG00000030967	Zranb1
ENSMUSG00000030968	Pdilt
ENSMUSG00000030970	Ctbp2
ENSMUSG00000030972	Acsm5
ENSMUSG00000030979	Uros
ENSMUSG00000030980	Knop1
ENSMUSG00000030983	Bccip
ENSMUSG00000030986	Dhx32
ENSMUSG00000030987	Stim1
ENSMUSG00000031010	Usp9x
ENSMUSG00000031015	Swap70
ENSMUSG00000031016	Wee1
ENSMUSG00000031021	Tmem9b
ENSMUSG00000031024	St5
ENSMUSG00000031059	Ndufb11
ENSMUSG00000031077	Fadd
ENSMUSG00000031129	Slc9a9
ENSMUSG00000031134	Rbmx
ENSMUSG00000031138	F9
ENSMUSG00000031153	Gripap1
ENSMUSG00000031154	Otud5
ENSMUSG00000031157	Pqbp1
ENSMUSG00000031166	Wdr13
ENSMUSG00000031168	Ebp
ENSMUSG00000031173	Otc
ENSMUSG00000031196	F8
ENSMUSG00000031207	Msn
ENSMUSG00000031216	Stard8
ENSMUSG00000031217	Efnb1
ENSMUSG00000031229	Atrx
ENSMUSG00000031231	Cox7b
ENSMUSG00000031232	Magt1
ENSMUSG00000031245	Hmgn5
ENSMUSG00000031271	Serpina7

ENSMUSG00000031286	Glt28d2
ENSMUSG00000031311	Nono
ENSMUSG00000031352	Hccs
ENSMUSG00000031355	Arhgap6
ENSMUSG00000031357	Syap1
ENSMUSG00000031365	Zfp275
ENSMUSG00000031378	Abcd1
ENSMUSG00000031386	Hcfc1
ENSMUSG00000031392	Irak1
ENSMUSG00000031393	Mecp2
ENSMUSG00000031399	Fam3a
ENSMUSG00000031422	Morf4l2
ENSMUSG00000031429	Psmd10
ENSMUSG00000031431	Tsc22d3
ENSMUSG00000031441	Atp11a
ENSMUSG00000031443	F7
ENSMUSG00000031444	F10
ENSMUSG00000031445	Proz
ENSMUSG00000031482	Slc25a15
ENSMUSG00000031483	Erlin2
ENSMUSG00000031485	Plpbp
ENSMUSG00000031486	Adgra2
ENSMUSG00000031487	Brf2
ENSMUSG00000031489	Adrb3
ENSMUSG00000031490	Eif4ebp1
ENSMUSG00000031504	Rab20
ENSMUSG00000031508	Ankrd10
ENSMUSG00000031513	Leprotl1
ENSMUSG00000031520	Vegfc
ENSMUSG00000031523	Dlc1
ENSMUSG00000031530	Dusp4
ENSMUSG00000031532	Saraf
ENSMUSG00000031534	Smim19
ENSMUSG00000031536	Polb
ENSMUSG00000031537	Ikbkb
ENSMUSG00000031540	Kat6a
ENSMUSG00000031545	Gpat4
ENSMUSG00000031546	Gins4
ENSMUSG00000031549	Ido2
ENSMUSG00000031556	Tm2d2
ENSMUSG00000031561	Tenm3
ENSMUSG00000031565	Fgfr1
ENSMUSG00000031568	Rwdd4a
ENSMUSG00000031570	Plpp5

ENSMUSG00000031577	Tti2
ENSMUSG00000031578	Mak16
ENSMUSG00000031594	Fgl1
ENSMUSG00000031596	Slc7a2
ENSMUSG00000031601	Cnot7
ENSMUSG00000031605	Klhl2
ENSMUSG00000031613	Hpgd
ENSMUSG00000031617	Tmem184c
ENSMUSG00000031618	Nr3c2
ENSMUSG00000031622	Sin3b
ENSMUSG00000031640	Gm45753
ENSMUSG00000031641	Cbr4
ENSMUSG00000031645	F11
ENSMUSG00000031652	N4bp1
ENSMUSG00000031660	Brd7
ENSMUSG00000031661	Nkd1
ENSMUSG00000031665	Sall1
ENSMUSG00000031666	Rbl2
ENSMUSG00000031668	Eif2ak3
ENSMUSG00000031671	Setd6
ENSMUSG00000031672	Got2
ENSMUSG00000031681	Smad1
ENSMUSG00000031684	Slc10a7
ENSMUSG00000031700	Gpt2
ENSMUSG00000031701	Dnaja2
ENSMUSG00000031708	Tecr
ENSMUSG00000031711	Zfp330
ENSMUSG00000031714	Gab1
ENSMUSG00000031722	Hp
ENSMUSG00000031725	Ces1f
ENSMUSG00000031729	Ist1
ENSMUSG00000031731	Ap1g1
ENSMUSG00000031751	Amfr
ENSMUSG00000031753	Cog4
ENSMUSG00000031762	Mt2
ENSMUSG00000031765	Mt1
ENSMUSG00000031767	Nudt7
ENSMUSG00000031770	Herpud1
ENSMUSG00000031774	Fam192a
ENSMUSG00000031782	Coq9
ENSMUSG00000031790	Mmp15
ENSMUSG00000031799	Tpm4
ENSMUSG00000031803	B3gnt3
ENSMUSG00000031812	Map1lc3b

ENSMUSG00000031819	Emc8
ENSMUSG00000031820	Babam1
ENSMUSG00000031822	Gse1
ENSMUSG00000031823	Zdhhc7
ENSMUSG00000031824	6430548M08Rik
ENSMUSG00000031833	Mast3
ENSMUSG00000031835	Mbtps1
ENSMUSG00000031842	Pde4c
ENSMUSG00000031844	Hsd17b2
ENSMUSG00000031845	Bco1
ENSMUSG00000031858	Mau2
ENSMUSG00000031871	Cdh5
ENSMUSG00000031886	Ces2e
ENSMUSG00000031887	Tradd
ENSMUSG00000031902	Nfatc3
ENSMUSG00000031903	Pla2g15
ENSMUSG00000031913	Vps4a
ENSMUSG00000031916	Cog8
ENSMUSG00000031917	Nip7
ENSMUSG00000031918	Mtmr2
ENSMUSG00000031921	Terf2
ENSMUSG00000031922	Cep57
ENSMUSG00000031924	Cyb5b
ENSMUSG00000031925	Maml2
ENSMUSG00000031931	Ankrd49
ENSMUSG00000031939	Taf1d
ENSMUSG00000031954	Cfdp1
ENSMUSG00000031955	Bcar1
ENSMUSG00000031958	Ldhd
ENSMUSG00000031959	Wdr59
ENSMUSG00000031960	Aars
ENSMUSG00000031967	Afg3l1
ENSMUSG00000031974	Abcb10
ENSMUSG00000031976	Urb2
ENSMUSG00000031980	Agt
ENSMUSG00000031984	2810004N23Rik
ENSMUSG00000031986	Sprtn
ENSMUSG00000031987	Egln1
ENSMUSG00000031993	Snx19
ENSMUSG00000031996	Aplp2
ENSMUSG00000032000	Birc3
ENSMUSG00000032009	Sesn3
ENSMUSG00000032010	Usp2
ENSMUSG00000032012	Nectin1

ENSMUSG00000032014	Oaf
ENSMUSG00000032018	Sc5d
ENSMUSG00000032026	Rexo2
ENSMUSG00000032028	Nxpe2
ENSMUSG00000032030	Cul5
ENSMUSG00000032035	Ets1
ENSMUSG00000032040	Dcps
ENSMUSG00000032041	Tirap
ENSMUSG00000032044	Rpusd4
ENSMUSG00000032046	Abhd12
ENSMUSG00000032047	Acat1
ENSMUSG00000032050	Rdx
ENSMUSG00000032058	Ppp2r1b
ENSMUSG00000032059	Alg9
ENSMUSG00000032062	2310030G06Rik
ENSMUSG00000032064	Dixdc1
ENSMUSG00000032066	Bco2
ENSMUSG00000032068	Plet1
ENSMUSG00000032077	Bud13
ENSMUSG00000032078	Zpr1
ENSMUSG00000032079	Apoa5
ENSMUSG00000032080	Apoa4
ENSMUSG00000032083	Apoa1
ENSMUSG00000032086	Bace1
ENSMUSG00000032089	Il10ra
ENSMUSG00000032096	Arcn1
ENSMUSG00000032097	Ddx6
ENSMUSG00000032103	Pus3
ENSMUSG00000032109	Nlrx1
ENSMUSG00000032119	Hinfp
ENSMUSG00000032122	Slc37a2
ENSMUSG00000032125	Robo4
ENSMUSG00000032127	Vps11
ENSMUSG00000032171	Pin1
ENSMUSG00000032180	Tmed1
ENSMUSG00000032185	Carm1
ENSMUSG00000032187	Smarca4
ENSMUSG00000032193	Ldlr
ENSMUSG00000032194	Kank2
ENSMUSG00000032198	Dock6
ENSMUSG00000032199	Polr2m
ENSMUSG00000032204	Aqp9
ENSMUSG00000032207	Lipc
ENSMUSG00000032212	Sltm

ENSMUSG00000032217	Rnf111
ENSMUSG00000032220	Myo1e
ENSMUSG00000032228	Tcf12
ENSMUSG00000032232	Cgnl1
ENSMUSG00000032235	Ice2
ENSMUSG00000032238	Rora
ENSMUSG00000032239	Rp9
ENSMUSG00000032244	Fem1b
ENSMUSG00000032249	Anp32a
ENSMUSG00000032252	Glce
ENSMUSG00000032253	Phip
ENSMUSG00000032261	Sh3bgrl2
ENSMUSG00000032263	Bckdhb
ENSMUSG00000032265	Tent5a
ENSMUSG00000032271	Nnmt
ENSMUSG00000032280	Tle3
ENSMUSG00000032288	Imp3
ENSMUSG00000032290	Ptpn9
ENSMUSG00000032293	Ireb2
ENSMUSG00000032300	1700017B05Rik
ENSMUSG00000032301	Psma4
ENSMUSG00000032309	Fbxo22
ENSMUSG00000032310	Cyp1a2
ENSMUSG00000032312	Csk
ENSMUSG00000032314	Etfa
ENSMUSG00000032316	Clk3
ENSMUSG00000032328	Tmem30a
ENSMUSG00000032329	Hmg20a
ENSMUSG00000032340	Neo1
ENSMUSG00000032348	Gsta4
ENSMUSG00000032349	Elovl5
ENSMUSG00000032350	Gclc
ENSMUSG00000032359	Ctsh
ENSMUSG00000032360	Hcrtr2
ENSMUSG00000032366	Tpm1
ENSMUSG00000032370	Lactb
ENSMUSG00000032372	Plscr2
ENSMUSG00000032376	Usp3
ENSMUSG00000032380	Dapk2
ENSMUSG00000032388	Spg21
ENSMUSG00000032392	Parp16
ENSMUSG00000032393	Dpp8
ENSMUSG00000032396	Dis3l
ENSMUSG00000032398	Snapc5

ENSMUSG00000032399	Rpl4
ENSMUSG00000032402	Smad3
ENSMUSG00000032405	Pias1
ENSMUSG00000032410	Xrn1
ENSMUSG00000032412	Atp1b3
ENSMUSG00000032423	Syncrip
ENSMUSG00000032434	Cmtm6
ENSMUSG00000032435	Dync1li1
ENSMUSG00000032437	Stt3b
ENSMUSG00000032440	Tgfbr2
ENSMUSG00000032456	Nmnat3
ENSMUSG00000032462	Pik3cb
ENSMUSG00000032468	Armc8
ENSMUSG00000032469	Dbr1
ENSMUSG00000032475	Nck1
ENSMUSG00000032479	Map4
ENSMUSG00000032480	Dhx30
ENSMUSG00000032492	Pth1r
ENSMUSG00000032497	Lrrfip2
ENSMUSG00000032500	Dclk3
ENSMUSG00000032501	Trib1
ENSMUSG00000032504	Pdcd6ip
ENSMUSG00000032512	Wdr48
ENSMUSG00000032513	Gorasp1
ENSMUSG00000032515	Csrnp1
ENSMUSG00000032518	Rpsa
ENSMUSG00000032526	Ss18l2
ENSMUSG00000032527	Pccb
ENSMUSG00000032528	Vipr1
ENSMUSG00000032531	Amotl2
ENSMUSG00000032536	Trak1
ENSMUSG00000032540	Abhd5
ENSMUSG00000032548	Slco2a1
ENSMUSG00000032551	1110059G10Rik
ENSMUSG00000032553	Srprb
ENSMUSG00000032554	Trf
ENSMUSG00000032560	Dnajc13
ENSMUSG00000032562	Gnai2
ENSMUSG00000032565	Nudt16
ENSMUSG00000032570	Atp2c1
ENSMUSG00000032571	Pik3r4
ENSMUSG00000032575	Manf
ENSMUSG00000032578	Cish
ENSMUSG00000032580	Rbm5

ENSMUSG00000032583	Mon1a
ENSMUSG00000032594	Ip6k1
ENSMUSG00000032599	Ip6k2
ENSMUSG00000032601	Prkar2a
ENSMUSG00000032602	Slc25a20
ENSMUSG00000032612	Usp4
ENSMUSG00000032621	Srek1
ENSMUSG00000032624	Eml4
ENSMUSG00000032633	Flcn
ENSMUSG00000032637	Atxn2l
ENSMUSG00000032641	Gpr19
ENSMUSG00000032652	Crebl2
ENSMUSG00000032673	Prorsd1
ENSMUSG00000032679	Cd59a
ENSMUSG00000032698	Lmo2
ENSMUSG00000032702	Kank1
ENSMUSG00000032712	Resf1
ENSMUSG00000032714	Syde1
ENSMUSG00000032715	Trib3
ENSMUSG00000032727	Mier3
ENSMUSG00000032733	Snx33
ENSMUSG00000032735	Ablim3
ENSMUSG00000032737	Inppl1
ENSMUSG00000032745	Gpbp1
ENSMUSG00000032754	Slc8b1
ENSMUSG00000032757	Bet1
ENSMUSG00000032763	Ilvbl
ENSMUSG00000032766	Gng11
ENSMUSG00000032776	Mctp2
ENSMUSG00000032777	Gtf3c1
ENSMUSG00000032786	Alas1
ENSMUSG00000032802	Srxn1
ENSMUSG00000032803	Cdv3
ENSMUSG00000032806	Slc10a3
ENSMUSG00000032808	Cyp2c38
ENSMUSG00000032812	Arap1
ENSMUSG00000032815	Fanca
ENSMUSG00000032826	Ank2
ENSMUSG00000032834	Pwp2
ENSMUSG00000032840	2410131K14Rik
ENSMUSG00000032846	Zswim6
ENSMUSG00000032855	Pkd1
ENSMUSG00000032860	P2ry2
ENSMUSG00000032869	Psmf1

ENSMUSG00000032870	Smap2
ENSMUSG00000032883	Acsl3
ENSMUSG00000032897	Nfyc
ENSMUSG00000032898	Fbxo21
ENSMUSG00000032902	Slc16a1
ENSMUSG00000032905	Atg12
ENSMUSG00000032913	Lrig2
ENSMUSG00000032932	Hspa13
ENSMUSG00000032952	Ap4b1
ENSMUSG00000032966	Fkbp1a
ENSMUSG00000032977	Fam207a
ENSMUSG00000033004	Mycbp2
ENSMUSG00000033014	Trim33
ENSMUSG00000033021	Gmppa
ENSMUSG00000033022	Cdo1
ENSMUSG00000033033	Calhm2
ENSMUSG00000033054	Npat
ENSMUSG00000033088	Triobp
ENSMUSG00000033096	Apmap
ENSMUSG00000033099	Nol12
ENSMUSG00000033102	Cdc14b
ENSMUSG00000033106	Slc7a6os
ENSMUSG00000033107	Rnf125
ENSMUSG00000033114	Slc35d2
ENSMUSG00000033124	Atg9a
ENSMUSG00000033128	Gga1
ENSMUSG00000033147	Slc22a15
ENSMUSG00000033149	Phldb2
ENSMUSG00000033159	Cnppd1
ENSMUSG00000033174	Mgll
ENSMUSG00000033184	Tmed7
ENSMUSG00000033216	Eefsec
ENSMUSG00000033228	Scaf11
ENSMUSG00000033237	Arid2
ENSMUSG00000033253	Szt2
ENSMUSG00000033256	Shf
ENSMUSG00000033257	Ttll4
ENSMUSG00000033272	Slc35a4
ENSMUSG00000033295	Ptprf
ENSMUSG00000033307	Mif
ENSMUSG00000033308	Dpyd
ENSMUSG00000033319	Fem1c
ENSMUSG00000033323	Ctdp1
ENSMUSG00000033326	Kdm4a

ENSMUSG00000033350	Chst2
ENSMUSG00000033352	Map2k4
ENSMUSG00000033355	Rtp4
ENSMUSG00000033382	Trappc8
ENSMUSG00000033386	Frrs1
ENSMUSG00000033396	Spg11
ENSMUSG00000033416	Gucd1
ENSMUSG00000033417	Cacul1
ENSMUSG00000033429	Mcee
ENSMUSG00000033430	Terf2ip
ENSMUSG00000033439	Trmt13
ENSMUSG00000033444	Specc1l
ENSMUSG00000033446	Lpar6
ENSMUSG00000033454	Zbtb1
ENSMUSG00000033478	Fam160b1
ENSMUSG00000033487	Fndc3a
ENSMUSG00000033499	Larp4b
ENSMUSG00000033510	Otud7a
ENSMUSG00000033533	Acsm1
ENSMUSG00000033540	Idua
ENSMUSG00000033542	Arhgef5
ENSMUSG00000033545	Znrf1
ENSMUSG00000033557	Fam20b
ENSMUSG00000033577	Myo6
ENSMUSG00000033594	Spata2l
ENSMUSG00000033596	Rfwd3
ENSMUSG00000033610	Pank1
ENSMUSG00000033618	Map3k13
ENSMUSG00000033623	Pcgf3
ENSMUSG00000033624	Pdpr
ENSMUSG00000033629	Hacd3
ENSMUSG00000033634	Nat8f2
ENSMUSG00000033671	Cep350
ENSMUSG00000033685	Ucp2
ENSMUSG00000033688	1300017J02Rik
ENSMUSG00000033705	Stard9
ENSMUSG00000033713	Foxn3
ENSMUSG00000033728	Lrrc14
ENSMUSG00000033732	Sf3b3
ENSMUSG00000033735	Spr
ENSMUSG00000033739	Fkbpl
ENSMUSG00000033751	Gadd45gip1
ENSMUSG00000033760	Rbm4b
ENSMUSG00000033767	Tmem131l

ENSMUSG00000033773	Rpap2
ENSMUSG00000033793	Atp6v1h
ENSMUSG00000033819	Ppp1r16a
ENSMUSG00000033831	Fgb
ENSMUSG00000033845	Mrpl15
ENSMUSG00000033855	Ston1
ENSMUSG00000033857	Engase
ENSMUSG00000033860	Fgg
ENSMUSG00000033863	Klf9
ENSMUSG00000033871	Ppargc1b
ENSMUSG00000033880	Lgals3bp
ENSMUSG00000033898	Cfhr2
ENSMUSG00000033909	Usp36
ENSMUSG00000033917	Gde1
ENSMUSG00000033931	Rbm34
ENSMUSG00000033938	Ndufb7
ENSMUSG00000033940	Brk1
ENSMUSG00000033943	Mga
ENSMUSG00000033953	Ppp3r1
ENSMUSG00000033955	Tnks1bp1
ENSMUSG00000033964	Zbtb41
ENSMUSG00000033965	Slc16a2
ENSMUSG00000033972	Zfp944
ENSMUSG00000033985	Tesk2
ENSMUSG00000033987	Dnah17
ENSMUSG00000034021	Pds5b
ENSMUSG00000034024	Cct2
ENSMUSG00000034037	Fgd5
ENSMUSG00000034064	Poglut1
ENSMUSG00000034066	Farp2
ENSMUSG00000034075	Zdhhc5
ENSMUSG00000034083	Ccdc174
ENSMUSG00000034088	Hdlbp
ENSMUSG00000034101	Ctnnd1
ENSMUSG00000034108	Ccs
ENSMUSG00000034109	Golim4
ENSMUSG00000034118	Tpst1
ENSMUSG00000034126	Pomt2
ENSMUSG00000034135	Sik3
ENSMUSG00000034152	Exoc3
ENSMUSG00000034157	Cipc
ENSMUSG00000034158	Lrrc58
ENSMUSG00000034160	Ogt
ENSMUSG00000034163	Zfc3h1

ENSMUSG00000034168	Irf2bpl
ENSMUSG00000034171	Faah
ENSMUSG00000034177	Rnf43
ENSMUSG00000034190	Chmp7
ENSMUSG00000034194	R3hcc1
ENSMUSG00000034201	Gas2l1
ENSMUSG00000034203	Chchd4
ENSMUSG00000034216	Vps18
ENSMUSG00000034218	Atm
ENSMUSG00000034243	Golgb1
ENSMUSG00000034247	Plekhm1
ENSMUSG00000034248	Slc25a37
ENSMUSG00000034252	Senp6
ENSMUSG00000034254	Agpat1
ENSMUSG00000034259	Exosc4
ENSMUSG00000034265	Zdhhc14
ENSMUSG00000034269	Setd5
ENSMUSG00000034271	Jdp2
ENSMUSG00000034285	Nipsnap1
ENSMUSG00000034290	Nek9
ENSMUSG00000034292	Traf3ip1
ENSMUSG00000034297	Med13
ENSMUSG00000034300	Fam53c
ENSMUSG00000034308	Sdr42e1
ENSMUSG00000034312	Iqsec1
ENSMUSG00000034343	Ube2f
ENSMUSG00000034345	Gtf2h5
ENSMUSG00000034354	Mtmr3
ENSMUSG00000034382	AI661453
ENSMUSG00000034403	Pja1
ENSMUSG00000034412	Tbc1d10a
ENSMUSG00000034422	Parp14
ENSMUSG00000034429	Zfp707
ENSMUSG00000034430	Zxdc
ENSMUSG00000034432	Cops8
ENSMUSG00000034435	Tmem30b
ENSMUSG00000034450	Gulo
ENSMUSG00000034453	Polr3b
ENSMUSG00000034456	Uroc1
ENSMUSG00000034459	Ifit1
ENSMUSG00000034471	Caskin2
ENSMUSG00000034473	Sec22a
ENSMUSG00000034484	Snx2
ENSMUSG00000034485	Uaca

ENSMUSG00000034487	Kdelc2
ENSMUSG00000034501	Pcnx4
ENSMUSG00000034509	Mad2l1bp
ENSMUSG00000034525	Ice1
ENSMUSG00000034528	Hsd17b13
ENSMUSG00000034543	Morc2a
ENSMUSG00000034557	Zfyve9
ENSMUSG00000034560	Washc4
ENSMUSG00000034563	Ccpg1
ENSMUSG00000034574	Daam1
ENSMUSG00000034584	Exph5
ENSMUSG00000034595	Ppp1r18
ENSMUSG00000034621	Gpatch8
ENSMUSG00000034636	Zyg11b
ENSMUSG00000034639	Setmar
ENSMUSG00000034640	Tiparp
ENSMUSG00000034663	Bmp2k
ENSMUSG00000034673	Pbx2
ENSMUSG00000034684	Sema3f
ENSMUSG00000034687	Fras1
ENSMUSG00000034707	Gns
ENSMUSG00000034708	Grn
ENSMUSG00000034714	Ttyh2
ENSMUSG00000034723	Tmx4
ENSMUSG00000034757	Tmub2
ENSMUSG00000034780	B3galt1
ENSMUSG00000034785	Dio1
ENSMUSG00000034789	Rab24
ENSMUSG00000034801	Sos2
ENSMUSG00000034807	Colgalt1
ENSMUSG00000034820	Cpsf7
ENSMUSG00000034826	Nup54
ENSMUSG00000034832	Tet3
ENSMUSG00000034837	Gnat1
ENSMUSG00000034845	Plvap
ENSMUSG00000034850	Tmem127
ENSMUSG00000034854	Mfsd12
ENSMUSG00000034858	Fam214a
ENSMUSG00000034867	Ankrd27
ENSMUSG00000034875	Nudt19
ENSMUSG00000034880	Mrpl34
ENSMUSG00000034881	Tbxa2r
ENSMUSG00000034889	Cactin
ENSMUSG00000034893	Cog3

ENSMUSG00000034902	Pip5k1c
ENSMUSG00000034903	Cobll1
ENSMUSG00000034926	Dhcr24
ENSMUSG00000034930	Rtkn
ENSMUSG00000034931	Dhx8
ENSMUSG00000034932	Mrpl54
ENSMUSG00000034936	Arl4d
ENSMUSG00000034940	Synrg
ENSMUSG00000034947	Tmem106a
ENSMUSG00000034951	Cog7
ENSMUSG00000034957	Cebpa
ENSMUSG00000034973	Dop1a
ENSMUSG00000034994	Eef2
ENSMUSG00000034998	Foxn2
ENSMUSG00000035004	Igsf6
ENSMUSG00000035007	Rundc1
ENSMUSG00000035011	Zbtb7a
ENSMUSG00000035031	C8a
ENSMUSG00000035041	Creb3l3
ENSMUSG00000035047	Kri1
ENSMUSG00000035069	Oma1
ENSMUSG00000035086	Becn1
ENSMUSG00000035093	Secisbp2l
ENSMUSG00000035104	Eva1a
ENSMUSG00000035105	Egln3
ENSMUSG00000035133	Arhgap5
ENSMUSG00000035139	Secisbp2
ENSMUSG00000035161	Ints6
ENSMUSG00000035168	Tanc1
ENSMUSG00000035172	Plekhh3
ENSMUSG00000035184	Fam124a
ENSMUSG00000035198	Tubg1
ENSMUSG00000035199	Arl6ip5
ENSMUSG00000035202	Lars2
ENSMUSG00000035203	Epn1
ENSMUSG00000035206	Sppl2b
ENSMUSG00000035227	Spcs2
ENSMUSG00000035235	Trim13
ENSMUSG00000035237	Lcat
ENSMUSG00000035247	Hectd1
ENSMUSG00000035248	Tut7
ENSMUSG00000035278	Plekhj1
ENSMUSG00000035310	Lin54
ENSMUSG00000035329	Fbxo33

ENSMUSG00000035342	Lzts2
ENSMUSG00000035354	Uvrag
ENSMUSG00000035367	Rmi1
ENSMUSG00000035370	Gm49322
ENSMUSG00000035372	1810055G02Rik
ENSMUSG00000035376	Hacd2
ENSMUSG00000035378	Shq1
ENSMUSG00000035382	Pcsk7
ENSMUSG00000035399	Oser1
ENSMUSG00000035401	Emsy
ENSMUSG00000035451	Foxa1
ENSMUSG00000035469	Rcbtb1
ENSMUSG00000035473	Galm
ENSMUSG00000035478	Mbd3
ENSMUSG00000035493	Tgfbi
ENSMUSG00000035504	Reep6
ENSMUSG00000035529	Prdm4
ENSMUSG00000035530	Eif1
ENSMUSG00000035540	Gc
ENSMUSG00000035545	Leng8
ENSMUSG00000035561	Aldh1b1
ENSMUSG00000035569	Ankrd11
ENSMUSG00000035572	Dcaf10
ENSMUSG00000035575	Utp6
ENSMUSG00000035578	Iqcg
ENSMUSG00000035614	Togaram1
ENSMUSG00000035620	Ric8b
ENSMUSG00000035621	Midn
ENSMUSG00000035623	Rsf1
ENSMUSG00000035637	Grhpr
ENSMUSG00000035640	Cbarp
ENSMUSG00000035666	Gtf3c4
ENSMUSG00000035671	Zswim4
ENSMUSG00000035692	Isg15
ENSMUSG00000035697	Arhgap45
ENSMUSG00000035704	Alg8
ENSMUSG00000035711	Dok3
ENSMUSG00000035726	Supt16
ENSMUSG00000035762	Tmem161b
ENSMUSG00000035764	Fbxo45
ENSMUSG00000035769	Xylb
ENSMUSG00000035770	Dync1li2
ENSMUSG00000035772	Mrps2
ENSMUSG00000035778	Ggta1

ENSMUSG00000035780	Ugt2a3
ENSMUSG00000035798	Zdhhc17
ENSMUSG00000035811	Ugt2b35
ENSMUSG00000035824	Tk2
ENSMUSG00000035828	Pim3
ENSMUSG00000035836	Ugt2b1
ENSMUSG00000035840	Lysmd3
ENSMUSG00000035842	Ddx11
ENSMUSG00000035845	Alg12
ENSMUSG00000035851	Ythdc1
ENSMUSG00000035875	AI182371
ENSMUSG00000035877	Zhx3
ENSMUSG00000035878	Hykk
ENSMUSG00000035890	Rnf126
ENSMUSG00000035914	Cd276
ENSMUSG00000035929	H2-Q4
ENSMUSG00000035941	Ibtk
ENSMUSG00000035949	Fbxw2
ENSMUSG00000035954	Dock4
ENSMUSG00000035960	Apex1
ENSMUSG00000035969	Rusc2
ENSMUSG00000035992	Fnip1
ENSMUSG00000036002	Fam214b
ENSMUSG00000036040	Adamtsl2
ENSMUSG00000036046	5031439G07Rik
ENSMUSG00000036057	Ptpn23
ENSMUSG00000036078	Sigmar1
ENSMUSG00000036083	Slc17a3
ENSMUSG00000036086	Zranb3
ENSMUSG00000036087	Slain2
ENSMUSG00000036091	Hyal3
ENSMUSG00000036093	Arl5a
ENSMUSG00000036099	Vezt
ENSMUSG00000036103	Colec12
ENSMUSG00000036104	Rab3gap1
ENSMUSG00000036106	Prr5
ENSMUSG00000036110	Slc17a2
ENSMUSG00000036114	Rpp25l
ENSMUSG00000036120	Rfxank
ENSMUSG00000036138	Acaa1a
ENSMUSG00000036155	Mgat5
ENSMUSG00000036158	Prickle1
ENSMUSG00000036160	Surf6
ENSMUSG00000036180	Gatad2a

ENSMUSG00000036181	Hist1h1c
ENSMUSG00000036197	Gxylt1
ENSMUSG00000036199	Ndufa13
ENSMUSG00000036202	Rif1
ENSMUSG00000036214	Znrd1as
ENSMUSG00000036225	Kctd1
ENSMUSG00000036241	Ube2r2
ENSMUSG00000036257	Pnpla8
ENSMUSG00000036275	9530068E07Rik
ENSMUSG00000036281	Snapc4
ENSMUSG00000036282	Naa30
ENSMUSG00000036323	Srp72
ENSMUSG00000036327	Qsox2
ENSMUSG00000036333	Kidins220
ENSMUSG00000036339	Tmem260
ENSMUSG00000036368	Rmdn2
ENSMUSG00000036371	Serbp1
ENSMUSG00000036376	Abt1
ENSMUSG00000036390	Gadd45a
ENSMUSG00000036391	Sec24a
ENSMUSG00000036398	Ppp1r11
ENSMUSG00000036427	Gpi1
ENSMUSG00000036430	Tbcc
ENSMUSG00000036432	Siah2
ENSMUSG00000036438	Calm2
ENSMUSG00000036442	Thap11
ENSMUSG00000036446	Lum
ENSMUSG00000036452	Arhgap26
ENSMUSG00000036461	Elf1
ENSMUSG00000036473	Tbc1d24
ENSMUSG00000036478	Btg1
ENSMUSG00000036492	Rnf39
ENSMUSG00000036499	Eea1
ENSMUSG00000036513	Commd2
ENSMUSG00000036534	Slc38a7
ENSMUSG00000036553	Sh3tc1
ENSMUSG00000036568	Bicral
ENSMUSG00000036570	Fxyd1
ENSMUSG00000036572	Upf3b
ENSMUSG00000036580	Spg20
ENSMUSG00000036585	Fgf1
ENSMUSG00000036591	Arhgap21
ENSMUSG00000036599	Chst12
ENSMUSG00000036606	Plxnb2

ENSMUSG00000036611	Eepd1
ENSMUSG00000036615	Rfxap
ENSMUSG00000036639	Nudt1
ENSMUSG00000036644	Tbc1d9b
ENSMUSG00000036655	Colec11
ENSMUSG00000036661	Dennd3
ENSMUSG00000036676	Tmtc3
ENSMUSG00000036687	Tmem184a
ENSMUSG00000036693	Nop14
ENSMUSG00000036707	Cab39
ENSMUSG00000036721	Zscan12
ENSMUSG00000036733	Rbm42
ENSMUSG00000036737	Oxsr1
ENSMUSG00000036748	Cuedc2
ENSMUSG00000036751	Cox6b1
ENSMUSG00000036752	Tubb4b
ENSMUSG00000036775	Decr2
ENSMUSG00000036779	Tent4b
ENSMUSG00000036781	Rps27l
ENSMUSG00000036813	Entpd8
ENSMUSG00000036817	Sun1
ENSMUSG00000036819	Jmjd4
ENSMUSG00000036822	Topors
ENSMUSG00000036840	Siah1a
ENSMUSG00000036850	Mrpl41
ENSMUSG00000036854	Hspb6
ENSMUSG00000036860	Mrpl55
ENSMUSG00000036863	Syde2
ENSMUSG00000036867	Smad6
ENSMUSG00000036885	Arhgef26
ENSMUSG00000036887	C1qa
ENSMUSG00000036893	Ehmt1
ENSMUSG00000036896	C1qc
ENSMUSG00000036898	Zfp157
ENSMUSG00000036904	Fzd8
ENSMUSG00000036905	C1qb
ENSMUSG00000036908	Unc93b1
ENSMUSG00000036918	Ttc7
ENSMUSG00000036932	Aifm1
ENSMUSG00000036934	4921524J17Rik
ENSMUSG00000036940	Kdm1a
ENSMUSG00000036941	Elac1
ENSMUSG00000036955	Kif1bp
ENSMUSG00000036957	Lrfn3

ENSMUSG00000036964	Trim17
ENSMUSG00000036975	Tmem177
ENSMUSG00000036986	Pml
ENSMUSG00000036989	Trim3
ENSMUSG00000036990	Otud4
ENSMUSG00000036992	Nxt1
ENSMUSG00000036995	Asap3
ENSMUSG00000037003	Tns2
ENSMUSG00000037013	Ss18
ENSMUSG00000037022	Mmaa
ENSMUSG00000037025	Foxa2
ENSMUSG00000037029	Zfp146
ENSMUSG00000037035	Inhbb
ENSMUSG00000037049	Smpd1
ENSMUSG00000037053	Azgp1
ENSMUSG00000037058	Paip2
ENSMUSG00000037062	Sh3glb1
ENSMUSG00000037070	Rbmxl1
ENSMUSG00000037071	Scd1
ENSMUSG00000037072	Selenof
ENSMUSG00000037075	Rnf139
ENSMUSG00000037085	Trmt12
ENSMUSG00000037089	Slc35b2
ENSMUSG00000037095	Lrg1
ENSMUSG00000037098	Rab11fip3
ENSMUSG00000037104	Socs5
ENSMUSG00000037110	Ralgapa2
ENSMUSG00000037112	Sik2
ENSMUSG00000037119	Fam91a1
ENSMUSG00000037166	Ppp1r14a
ENSMUSG00000037174	Elf2
ENSMUSG00000037190	Cyb561d2
ENSMUSG00000037197	Rbm17
ENSMUSG00000037204	Atg101
ENSMUSG00000037206	Islr
ENSMUSG00000037210	Fam193a
ENSMUSG00000037211	Spry1
ENSMUSG00000037216	Lipt1
ENSMUSG00000037221	Mospd3
ENSMUSG00000037225	Fgf2
ENSMUSG00000037236	Matr3
ENSMUSG00000037242	Clic4
ENSMUSG00000037251	Pomk
ENSMUSG00000037253	Mex3c

ENSMUSG00000037254	Itih2
ENSMUSG00000037257	Aagab
ENSMUSG00000037260	Hgsnat
ENSMUSG00000037262	Kin
ENSMUSG00000037266	Rsrp1
ENSMUSG00000037270	4932438A13Rik
ENSMUSG00000037275	Gemin5
ENSMUSG00000037278	Tmem97
ENSMUSG00000037287	Tbcel
ENSMUSG00000037295	Ldlrap1
ENSMUSG00000037316	Bag4
ENSMUSG00000037326	Capn15
ENSMUSG00000037331	Larp1
ENSMUSG00000037339	Fam53a
ENSMUSG00000037343	Taf2
ENSMUSG00000037348	Paqr7
ENSMUSG00000037364	Srrt
ENSMUSG00000037366	Pafah2
ENSMUSG00000037370	Enpp1
ENSMUSG00000037400	Atp11b
ENSMUSG00000037405	Icam1
ENSMUSG00000037410	Tbc1d2b
ENSMUSG00000037415	Ranbp10
ENSMUSG00000037434	Slc30a1
ENSMUSG00000037438	Uqcrh-ps1
ENSMUSG00000037440	Vnn1
ENSMUSG00000037443	Cep85
ENSMUSG00000037461	Ints7
ENSMUSG00000037465	Klf10
ENSMUSG00000037470	Uggt1
ENSMUSG00000037475	Thoc2
ENSMUSG00000037486	Asxl2
ENSMUSG00000037487	Ubr5
ENSMUSG00000037503	Fam168b
ENSMUSG00000037514	Pank2
ENSMUSG00000037519	Ppfia1
ENSMUSG00000037523	Mavs
ENSMUSG00000037525	Bcdin3d
ENSMUSG00000037533	Rapgef6
ENSMUSG00000037536	Fbxo34
ENSMUSG00000037541	Shank2
ENSMUSG00000037542	Aldh8a1
ENSMUSG00000037552	Plekhg2
ENSMUSG00000037553	Zdhhc18

ENSMUSG00000037573	Tob1
ENSMUSG00000037583	Nr0b2
ENSMUSG00000037601	Nme1
ENSMUSG00000037608	Bclaf1
ENSMUSG00000037621	Atoh8
ENSMUSG00000037622	Wdtc1
ENSMUSG00000037624	Kcnk2
ENSMUSG00000037638	Zbtb42
ENSMUSG00000037643	Prkci
ENSMUSG00000037646	Vps13b
ENSMUSG00000037656	Slc20a2
ENSMUSG00000037674	Rfx7
ENSMUSG00000037679	Inf2
ENSMUSG00000037686	Aspg
ENSMUSG00000037692	Ahdc1
ENSMUSG00000037697	Ddhd1
ENSMUSG00000037703	Lzts3
ENSMUSG00000037709	Fam13a
ENSMUSG00000037710	Cisd1
ENSMUSG00000037712	Fermt2
ENSMUSG00000037722	Gnpnat1
ENSMUSG00000037730	Mynn
ENSMUSG00000037731	Themis2
ENSMUSG00000037736	Limch1
ENSMUSG00000037740	Mrps26
ENSMUSG00000037742	Eef1a1
ENSMUSG00000037750	Fam222b
ENSMUSG00000037752	Xkr8
ENSMUSG00000037761	Actr5
ENSMUSG00000037773	Pced1a
ENSMUSG00000037780	Mbl1
ENSMUSG00000037791	Phf12
ENSMUSG00000037797	Adh4
ENSMUSG00000037798	Mat1a
ENSMUSG00000037820	Tgm2
ENSMUSG00000037824	Tspan14
ENSMUSG00000037826	Ppm1k
ENSMUSG00000037857	Nufip2
ENSMUSG00000037876	Jmjd1c
ENSMUSG00000037885	Stk35
ENSMUSG00000037887	Dusp8
ENSMUSG00000037894	H2afz
ENSMUSG00000037896	Rcor1
ENSMUSG00000037902	Sirpa

ENSMUSG00000037904	Ankrd9
ENSMUSG00000037905	Bri3bp
ENSMUSG00000037926	Ssh2
ENSMUSG00000037933	Bicd2
ENSMUSG00000037938	Chchd5
ENSMUSG00000037942	Crp
ENSMUSG00000037957	Wdr20
ENSMUSG00000037958	Nsrp1
ENSMUSG00000037965	Zc3h7a
ENSMUSG00000037966	Ninj1
ENSMUSG00000037992	Rara
ENSMUSG00000037993	Dhx38
ENSMUSG00000037999	Arap2
ENSMUSG00000038002	Cramp1l
ENSMUSG00000038009	Dnajc22
ENSMUSG00000038013	Wipf2
ENSMUSG00000038014	Fam120a
ENSMUSG00000038024	Dennd4c
ENSMUSG00000038025	Phf2
ENSMUSG00000038028	Tigar
ENSMUSG00000038046	Mrm3
ENSMUSG00000038055	Dexi
ENSMUSG00000038056	Kmt2c
ENSMUSG00000038058	Nod1
ENSMUSG00000038060	Dlec1
ENSMUSG00000038068	Rnf144b
ENSMUSG00000038069	Cdkn2aip
ENSMUSG00000038080	Kdm1b
ENSMUSG00000038085	Cnbd2
ENSMUSG00000038092	Hsd3b5
ENSMUSG00000038095	Sbno1
ENSMUSG00000038116	Phf20
ENSMUSG00000038121	Fam210a
ENSMUSG00000038127	Ccdc50
ENSMUSG00000038145	Snrk
ENSMUSG00000038150	Ormdl3
ENSMUSG00000038160	Atg5
ENSMUSG00000038167	Plekhg6
ENSMUSG00000038170	Pde4dip
ENSMUSG00000038175	Mylip
ENSMUSG00000038178	Slc43a2
ENSMUSG00000038181	Chpf2
ENSMUSG00000038188	Scarf1
ENSMUSG00000038193	Hand2

ENSMUSG00000038206	Fbxo8
ENSMUSG00000038212	Mfsd14b
ENSMUSG00000038213	Tapbpl
ENSMUSG00000038214	Bend3
ENSMUSG00000038217	Tlcd2
ENSMUSG00000038224	Serpinf2
ENSMUSG00000038235	F11r
ENSMUSG00000038241	Cep250
ENSMUSG00000038250	Usp38
ENSMUSG00000038260	Trpm4
ENSMUSG00000038267	Slc22a23
ENSMUSG00000038268	Ovca2
ENSMUSG00000038279	Nop2
ENSMUSG00000038290	Smg6
ENSMUSG00000038302	Afg1l
ENSMUSG00000038312	Edem2
ENSMUSG00000038323	1700066M21Rik
ENSMUSG00000038332	Sesn1
ENSMUSG00000038342	Mlxip
ENSMUSG00000038344	Txlng
ENSMUSG00000038346	Zfp384
ENSMUSG00000038366	Lasp1
ENSMUSG00000038369	Ncoa6
ENSMUSG00000038371	Sbf2
ENSMUSG00000038383	Pigu
ENSMUSG00000038384	Setd1b
ENSMUSG00000038387	Rras
ENSMUSG00000038388	Mpp6
ENSMUSG00000038393	Txnip
ENSMUSG00000038403	Hjv
ENSMUSG00000038406	Scaf1
ENSMUSG00000038415	Foxq1
ENSMUSG00000038416	Cdc16
ENSMUSG00000038418	Egr1
ENSMUSG00000038421	Fcrla
ENSMUSG00000038422	Hdhd3
ENSMUSG00000038425	Poli
ENSMUSG00000038429	Usp5
ENSMUSG00000038446	Cdc40
ENSMUSG00000038451	Spsb2
ENSMUSG00000038456	Dennd2a
ENSMUSG00000038467	Chmp4b
ENSMUSG00000038481	Cdk19
ENSMUSG00000038485	Socs7

ENSMUSG00000038495	Otud7b
ENSMUSG00000038500	Prr3
ENSMUSG00000038503	Mesd
ENSMUSG00000038506	Dcun1d2
ENSMUSG00000038508	Gdf15
ENSMUSG00000038510	Rpf2
ENSMUSG00000038520	Tbc1d17
ENSMUSG00000038521	C1s1
ENSMUSG00000038522	Mfsd4b1
ENSMUSG00000038525	Armc10
ENSMUSG00000038526	Car14
ENSMUSG00000038527	C1rl
ENSMUSG00000038533	Cbfa2t2
ENSMUSG00000038535	Zfp280d
ENSMUSG00000038539	Atf5
ENSMUSG00000038544	Inip
ENSMUSG00000038563	Efl1
ENSMUSG00000038576	Susd4
ENSMUSG00000038578	Susd1
ENSMUSG00000038582	Pptc7
ENSMUSG00000038587	Akap12
ENSMUSG00000038591	Colec10
ENSMUSG00000038594	Cep85l
ENSMUSG00000038604	Ripor1
ENSMUSG00000038611	Phrf1
ENSMUSG00000038612	Mcl1
ENSMUSG00000038615	Nfe2l1
ENSMUSG00000038618	Rassf7
ENSMUSG00000038619	Ensa
ENSMUSG00000038633	Degs1
ENSMUSG00000038641	Akr1d1
ENSMUSG00000038642	Ctss
ENSMUSG00000038648	Creb3l2
ENSMUSG00000038658	Ric1
ENSMUSG00000038696	Mapkap1
ENSMUSG00000038697	Taf5l
ENSMUSG00000038705	Gmeb2
ENSMUSG00000038708	Golga4
ENSMUSG00000038712	Mindy1
ENSMUSG00000038722	Bud31
ENSMUSG00000038729	Akap2
ENSMUSG00000038733	Wdr26
ENSMUSG00000038742	Angptl6
ENSMUSG00000038745	Nlrp6

ENSMUSG00000038754	Elovl3
ENSMUSG00000038762	Abcf1
ENSMUSG00000038764	Ptpn3
ENSMUSG00000038766	Gabpb2
ENSMUSG00000038773	Kdm3b
ENSMUSG00000038776	Ephx1
ENSMUSG00000038780	Smurf1
ENSMUSG00000038781	Stap2
ENSMUSG00000038803	Ost4
ENSMUSG00000038806	Sde2
ENSMUSG00000038828	Tmem214
ENSMUSG00000038831	Ralgps1
ENSMUSG00000038845	Phb
ENSMUSG00000038848	Ythdf1
ENSMUSG00000038855	Itpkb
ENSMUSG00000038861	Pi4kb
ENSMUSG00000038866	Zcchc2
ENSMUSG00000038871	Bpgm
ENSMUSG00000038876	Rnf146
ENSMUSG00000038880	Mrps34
ENSMUSG00000038884	A230050P20Rik
ENSMUSG00000038886	Man2a2
ENSMUSG00000038894	Irs2
ENSMUSG00000038902	Pogz
ENSMUSG00000038910	Plcl2
ENSMUSG00000038914	Dido1
ENSMUSG00000038949	Cnst
ENSMUSG00000038957	Edc3
ENSMUSG00000038976	Ppp1r9b
ENSMUSG00000038982	Bloc1s5
ENSMUSG00000038990	Cables2
ENSMUSG00000038991	Txndc5
ENSMUSG00000039016	Timm8b
ENSMUSG00000039046	Usp6nl
ENSMUSG00000039047	Pigk
ENSMUSG00000039048	Foxred1
ENSMUSG00000039050	Osbpl2
ENSMUSG00000039062	Anpep
ENSMUSG00000039065	Atpsckmt
ENSMUSG00000039067	Psmd7
ENSMUSG00000039068	Zzz3
ENSMUSG00000039069	Mtg2
ENSMUSG00000039081	Zfp503
ENSMUSG00000039087	Rreb1

ENSMUSG00000039100	6-Mar
ENSMUSG00000039115	Itga9
ENSMUSG00000039117	Taf4
ENSMUSG00000039128	Cdc123
ENSMUSG00000039145	Camk1d
ENSMUSG00000039148	Sart1
ENSMUSG00000039156	Stim2
ENSMUSG00000039159	Ube2h
ENSMUSG00000039163	Cmc1
ENSMUSG00000039164	Naif1
ENSMUSG00000039168	Dap
ENSMUSG00000039182	AW209491
ENSMUSG00000039195	1110008P14Rik
ENSMUSG00000039196	Orm1
ENSMUSG00000039197	Adk
ENSMUSG00000039205	Ciz1
ENSMUSG00000039208	Metrnl
ENSMUSG00000039210	Gpatch2
ENSMUSG00000039218	Srrm2
ENSMUSG00000039219	Arid4b
ENSMUSG00000039220	Ppp1r10
ENSMUSG00000039221	Rpl22l1
ENSMUSG00000039238	Zfp750
ENSMUSG00000039244	E130309D02Rik
ENSMUSG00000039262	Prrc2b
ENSMUSG00000039270	Megf9
ENSMUSG00000039275	Foxk2
ENSMUSG00000039285	Azi2
ENSMUSG00000039294	Cybc1
ENSMUSG00000039298	Cdk5rap2
ENSMUSG00000039304	Tnfsf10
ENSMUSG00000039308	Ndst2
ENSMUSG00000039349	C130074G19Rik
ENSMUSG00000039361	Picalm
ENSMUSG00000039367	Sec24c
ENSMUSG00000039377	Hlx
ENSMUSG00000039382	Wdr45
ENSMUSG00000039395	Mreg
ENSMUSG00000039405	Prss23
ENSMUSG00000039414	Heatr5b
ENSMUSG00000039428	Tmem135
ENSMUSG00000039438	Ttc36
ENSMUSG00000039449	Prpf18
ENSMUSG00000039450	Dcxr

ENSMUSG00000039456	Morc3
ENSMUSG00000039457	Ppl
ENSMUSG00000039458	Mtmr12
ENSMUSG00000039473	Ubn1
ENSMUSG00000039477	Tnrc18
ENSMUSG00000039483	Asb6
ENSMUSG00000039497	Dse
ENSMUSG00000039501	Znfx1
ENSMUSG00000039512	Uhrf1bp1
ENSMUSG00000039519	Cyp7b1
ENSMUSG00000039529	Atp8b1
ENSMUSG00000039536	Stau1
ENSMUSG00000039568	Ubald1
ENSMUSG00000039585	Myo9a
ENSMUSG00000039599	Fam149b
ENSMUSG00000039615	Stub1
ENSMUSG00000039620	Trmt9b
ENSMUSG00000039621	Prex1
ENSMUSG00000039623	Ap5z1
ENSMUSG00000039630	Hnrnpu
ENSMUSG00000039633	Lonrf1
ENSMUSG00000039634	Zfp189
ENSMUSG00000039637	Coro7
ENSMUSG00000039640	Mrpl12
ENSMUSG00000039646	Vasn
ENSMUSG00000039653	Baat
ENSMUSG00000039656	Rxrb
ENSMUSG00000039660	Spout1
ENSMUSG00000039662	Icmt
ENSMUSG00000039670	Oxld1
ENSMUSG00000039671	Zmynd8
ENSMUSG00000039678	Tbc1d13
ENSMUSG00000039680	Mrps6
ENSMUSG00000039701	Usp53
ENSMUSG00000039703	Nploc4
ENSMUSG00000039713	Plekhg5
ENSMUSG00000039725	Trp53rka
ENSMUSG00000039737	Prkrip1
ENSMUSG00000039740	Alg2
ENSMUSG00000039745	Htatip2
ENSMUSG00000039753	Fbxl5
ENSMUSG00000039754	Alkbh4
ENSMUSG00000039756	Dnttip2
ENSMUSG00000039759	Thap3

ENSMUSG00000039763	Dnajc28
ENSMUSG00000039768	Dnajc11
ENSMUSG00000039770	Ypel5
ENSMUSG00000039782	Cpeb2
ENSMUSG00000039783	Kmo
ENSMUSG00000039789	Zfp597
ENSMUSG00000039804	Ncoa5
ENSMUSG00000039809	Gabbr2
ENSMUSG00000039810	Zc3h10
ENSMUSG00000039826	Trub2
ENSMUSG00000039831	Arhgap29
ENSMUSG00000039835	Nhsl1
ENSMUSG00000039840	Epg5
ENSMUSG00000039841	Zfp800
ENSMUSG00000039844	Rapgef1
ENSMUSG00000039849	Pcif1
ENSMUSG00000039850	Endov
ENSMUSG00000039852	Rere
ENSMUSG00000039879	Heca
ENSMUSG00000039899	Fgl2
ENSMUSG00000039910	Cited2
ENSMUSG00000039917	Rhbdd2
ENSMUSG00000039929	Urb1
ENSMUSG00000039942	Ptger4
ENSMUSG00000039952	Dag1
ENSMUSG00000039956	Mrap
ENSMUSG00000039958	Etfbkmt
ENSMUSG00000039959	Hip1
ENSMUSG00000039960	Rhou
ENSMUSG00000039967	Zfp292
ENSMUSG00000039968	Rsbn1l
ENSMUSG00000039976	Tbc1d16
ENSMUSG00000039983	Ccdc32
ENSMUSG00000039989	Cbx4
ENSMUSG00000039990	Edrf1
ENSMUSG00000039997	Ifi203
ENSMUSG00000040006	Ginm1
ENSMUSG00000040007	Bahd1
ENSMUSG00000040010	Slc7a5
ENSMUSG00000040017	Saa4
ENSMUSG00000040018	Cox15
ENSMUSG00000040021	Lats1
ENSMUSG00000040025	Ythdf2
ENSMUSG00000040029	Ipo8

ENSMUSG00000040033	Stat2
ENSMUSG00000040043	Rbms2
ENSMUSG00000040048	Ndufb10
ENSMUSG00000040054	Baz2a
ENSMUSG00000040078	Ptges3-ps
ENSMUSG00000040093	Bmf
ENSMUSG00000040097	Flywch1
ENSMUSG00000040102	Klhl42
ENSMUSG00000040105	Plpp6
ENSMUSG00000040123	Zmym5
ENSMUSG00000040124	Gorab
ENSMUSG00000040127	Sdr9c7
ENSMUSG00000040128	Pnrc1
ENSMUSG00000040134	Rdh7
ENSMUSG00000040146	Rgl3
ENSMUSG00000040147	Maob
ENSMUSG00000040151	Hs2st1
ENSMUSG00000040167	Ikzf5
ENSMUSG00000040170	Fmo2
ENSMUSG00000040177	2310057M21Rik
ENSMUSG00000040188	Scamp2
ENSMUSG00000040213	Kyat3
ENSMUSG00000040225	Prrc2c
ENSMUSG00000040236	Trappc5
ENSMUSG00000040242	Fgfr1op2
ENSMUSG00000040253	Gbp7
ENSMUSG00000040263	Klhdc4
ENSMUSG00000040268	Plekha1
ENSMUSG00000040269	Mrps28
ENSMUSG00000040283	Btnl9
ENSMUSG00000040296	Ddx58
ENSMUSG00000040297	Suco
ENSMUSG00000040302	Rbm48
ENSMUSG00000040325	Dcaf1
ENSMUSG00000040328	Olfr56
ENSMUSG00000040350	Trim7
ENSMUSG00000040351	Ankib1
ENSMUSG00000040359	Ufl1
ENSMUSG00000040365	Trim41
ENSMUSG00000040370	Etfrf1
ENSMUSG00000040374	Pex2
ENSMUSG00000040385	Ppp1ca
ENSMUSG00000040390	Map3k10
ENSMUSG00000040396	Abhd13

ENSMUSG00000040407	Akap9
ENSMUSG00000040410	Fbxl4
ENSMUSG00000040413	Timd2
ENSMUSG00000040414	Slc25a28
ENSMUSG00000040430	Pitpnc1
ENSMUSG00000040433	Zbtb38
ENSMUSG00000040435	Ppp1r15a
ENSMUSG00000040441	Slc26a10
ENSMUSG00000040447	Spns2
ENSMUSG00000040451	Sgms1
ENSMUSG00000040459	Arglu1
ENSMUSG00000040462	Os9
ENSMUSG00000040463	Mybbp1a
ENSMUSG00000040464	Gtpbp10
ENSMUSG00000040471	Ggt6
ENSMUSG00000040479	Dgkz
ENSMUSG00000040481	Bptf
ENSMUSG00000040505	Abcg5
ENSMUSG00000040506	Ambra1
ENSMUSG00000040520	Manea
ENSMUSG00000040524	Zfp609
ENSMUSG00000040536	Necab1
ENSMUSG00000040548	Tex2
ENSMUSG00000040549	Ckap5
ENSMUSG00000040564	Apoc1
ENSMUSG00000040591	1110051M20Rik
ENSMUSG00000040594	Ranbp17
ENSMUSG00000040599	Mis12
ENSMUSG00000040605	Bace2
ENSMUSG00000040612	Ildr2
ENSMUSG00000040613	Apobec1
ENSMUSG00000040616	Tmem51
ENSMUSG00000040620	Dhx33
ENSMUSG00000040624	Plekhg1
ENSMUSG00000040640	Erc2
ENSMUSG00000040648	Ppip5k2
ENSMUSG00000040651	Tasor
ENSMUSG00000040652	Oaz2
ENSMUSG00000040659	Efhd2
ENSMUSG00000040661	Rad54l2
ENSMUSG00000040697	Dnajc16
ENSMUSG00000040701	Ap1g2
ENSMUSG00000040713	Creg1
ENSMUSG00000040714	Klc3

ENSMUSG00000040715	Rsc1a1
ENSMUSG00000040721	Zfhx2
ENSMUSG00000040725	Hnrnpul1
ENSMUSG00000040731	Eif4h
ENSMUSG00000040732	Erg
ENSMUSG00000040738	Ints8
ENSMUSG00000040747	Cd53
ENSMUSG00000040760	Appl1
ENSMUSG00000040761	Spen
ENSMUSG00000040771	Oard1
ENSMUSG00000040813	Tex264
ENSMUSG00000040818	Dennd6a
ENSMUSG00000040820	Hlcs
ENSMUSG00000040822	1700123O20Rik
ENSMUSG00000040824	Snrpd2
ENSMUSG00000040840	4930579G18Rik
ENSMUSG00000040841	Six5
ENSMUSG00000040842	Szrd1
ENSMUSG00000040843	Tiprl
ENSMUSG00000040848	Sft2d2
ENSMUSG00000040850	Psme4
ENSMUSG00000040857	Erf
ENSMUSG00000040859	Bsdc1
ENSMUSG00000040888	Gfer
ENSMUSG00000040891	Foxa3
ENSMUSG00000040904	Gm21988
ENSMUSG00000040913	Fbxw4
ENSMUSG00000040918	Slc19a2
ENSMUSG00000040943	Tet2
ENSMUSG00000040963	Asgr2
ENSMUSG00000040964	Arhgef10l
ENSMUSG00000040997	Abhd4
ENSMUSG00000041028	Ghitm
ENSMUSG00000041040	Fam117b
ENSMUSG00000041044	Lrit1
ENSMUSG00000041057	Wdr43
ENSMUSG00000041075	Fzd7
ENSMUSG00000041096	Tspyl2
ENSMUSG00000041124	Msantd4
ENSMUSG00000041130	Zfp598
ENSMUSG00000041132	N4bp2l1
ENSMUSG00000041133	Smc1a
ENSMUSG00000041135	Ripk2
ENSMUSG00000041143	Tmco4

ENSMUSG00000041161	Otud3
ENSMUSG00000041164	Zmiz2
ENSMUSG00000041168	Lonp1
ENSMUSG00000041187	Prkd2
ENSMUSG00000041203	Trir
ENSMUSG00000041215	Yeats2
ENSMUSG00000041220	Elovl6
ENSMUSG00000041225	Arhgap12
ENSMUSG00000041231	Ublcp1
ENSMUSG00000041235	Chd7
ENSMUSG00000041237	Pklr
ENSMUSG00000041238	Rbbp8
ENSMUSG00000041241	Mul1
ENSMUSG00000041258	Zfp236
ENSMUSG00000041264	Uspl1
ENSMUSG00000041278	Ttc1
ENSMUSG00000041293	Adgrf1
ENSMUSG00000041297	Cdk13
ENSMUSG00000041328	Pcf11
ENSMUSG00000041341	Atg2b
ENSMUSG00000041354	Rgl2
ENSMUSG00000041355	Ssr2
ENSMUSG00000041362	Shtn1
ENSMUSG00000041375	Ccdc9
ENSMUSG00000041378	Cldn5
ENSMUSG00000041406	BC055324
ENSMUSG00000041408	Wapl
ENSMUSG00000041415	Dicer1
ENSMUSG00000041417	Pik3r1
ENSMUSG00000041444	Arhgap32
ENSMUSG00000041445	Mmrn2
ENSMUSG00000041459	Tardbp
ENSMUSG00000041471	Shld2
ENSMUSG00000041483	Zfp281
ENSMUSG00000041540	Sox5
ENSMUSG00000041548	Hspb8
ENSMUSG00000041570	Camsap2
ENSMUSG00000041571	Selenow
ENSMUSG00000041577	Prelp
ENSMUSG00000041598	Cdc42ep4
ENSMUSG00000041629	Fam104a
ENSMUSG00000041632	Mrps27
ENSMUSG00000041638	Gcn1l1
ENSMUSG00000041645	Ddx24

ENSMUSG00000041671	Pyroxd1
ENSMUSG00000041684	Bivm
ENSMUSG00000041698	Slco1a1
ENSMUSG00000041702	Btbd7
ENSMUSG00000041716	Gm20604
ENSMUSG00000041720	Pi4ka
ENSMUSG00000041733	Coq5
ENSMUSG00000041740	Rnf10
ENSMUSG00000041747	Utp15
ENSMUSG00000041757	Plekha6
ENSMUSG00000041762	Gpr155
ENSMUSG00000041769	Ppp2r2d
ENSMUSG00000041773	Enc1
ENSMUSG00000041774	Ydjc
ENSMUSG00000041775	Mapk1ip1
ENSMUSG00000041777	Cir1
ENSMUSG00000041779	Tram2
ENSMUSG00000041781	Cpsf2
ENSMUSG00000041798	Gck
ENSMUSG00000041815	Poldip3
ENSMUSG00000041827	Oasl1
ENSMUSG00000041828	Abca8a
ENSMUSG00000041845	Rhod
ENSMUSG00000041846	Ppp4r3a
ENSMUSG00000041849	Card6
ENSMUSG00000041852	Tcf20
ENSMUSG00000041879	Ipo9
ENSMUSG00000041891	Lman1
ENSMUSG00000041915	Ammecr1l
ENSMUSG00000041920	Slc16a6
ENSMUSG00000041930	Fam222a
ENSMUSG00000041935	AW549877
ENSMUSG00000041959	S100a10
ENSMUSG00000041961	Znrf3
ENSMUSG00000041975	Mettl8
ENSMUSG00000041992	Rapgef5
ENSMUSG00000041995	Zbed3
ENSMUSG00000041997	Tlk1
ENSMUSG00000042010	Acacb
ENSMUSG00000042041	2010003K11Rik
ENSMUSG00000042042	Csgalnact2
ENSMUSG00000042063	Zfp386
ENSMUSG00000042066	Tmcc2
ENSMUSG00000042079	Hnrnpf

ENSMUSG00000042082	Arsb
ENSMUSG00000042099	Kank3
ENSMUSG00000042102	Dmgdh
ENSMUSG00000042105	Inpp5f
ENSMUSG00000042118	Bhmt2
ENSMUSG00000042121	Ssh1
ENSMUSG00000042129	Rassf4
ENSMUSG00000042133	Ppig
ENSMUSG00000042138	Msantd2
ENSMUSG00000042148	Cox10
ENSMUSG00000042178	Armc5
ENSMUSG00000042185	Nfrkb
ENSMUSG00000042190	Cmklr1
ENSMUSG00000042202	Slc35e2
ENSMUSG00000042207	Kdm5b
ENSMUSG00000042211	Fbxo38
ENSMUSG00000042213	Zfand4
ENSMUSG00000042228	Lyn
ENSMUSG00000042229	Rabif
ENSMUSG00000042251	Pm20d1
ENSMUSG00000042275	Pelo
ENSMUSG00000042284	Itga1
ENSMUSG00000042289	Hsd3b7
ENSMUSG00000042292	Mrtfa
ENSMUSG00000042293	Gm5617
ENSMUSG00000042298	Ttc19
ENSMUSG00000042302	Ehbp1
ENSMUSG00000042305	Tmem183a
ENSMUSG00000042308	Setd1a
ENSMUSG00000042312	S100a13
ENSMUSG00000042323	Pbrm1
ENSMUSG00000042328	Hps4
ENSMUSG00000042348	Arl15
ENSMUSG00000042350	Arel1
ENSMUSG00000042354	Gnl3
ENSMUSG00000042363	Lgalsl
ENSMUSG00000042364	Snx18
ENSMUSG00000042380	Smim12
ENSMUSG00000042396	Rbm7
ENSMUSG00000042406	Atf4
ENSMUSG00000042419	Nfkbil1
ENSMUSG00000042423	Fbrs
ENSMUSG00000042429	Adora1
ENSMUSG00000042444	Mindy2

ENSMUSG00000042460	C1galt1
ENSMUSG00000042476	Abcb4
ENSMUSG00000042485	Mustn1
ENSMUSG00000042487	Leo1
ENSMUSG00000042492	Tbc1d10b
ENSMUSG00000042500	Ago4
ENSMUSG00000042502	Cd2bp2
ENSMUSG00000042505	Sdhaf3
ENSMUSG00000042507	Elmsan1
ENSMUSG00000042510	AA986860
ENSMUSG00000042541	Sem1
ENSMUSG00000042548	Asxl1
ENSMUSG00000042557	Sin3a
ENSMUSG00000042558	Adprhl2
ENSMUSG00000042589	Cux2
ENSMUSG00000042594	Sh2b3
ENSMUSG00000042599	Kdm7a
ENSMUSG00000042608	Stk40
ENSMUSG00000042613	Pbxip1
ENSMUSG00000042622	Maff
ENSMUSG00000042625	Safb2
ENSMUSG00000042628	Zfyve1
ENSMUSG00000042642	Flad1
ENSMUSG00000042650	Alkbh5
ENSMUSG00000042659	Arrdc4
ENSMUSG00000042660	Wdr55
ENSMUSG00000042675	Ypel3
ENSMUSG00000042680	Garem1
ENSMUSG00000042688	Mapk6
ENSMUSG00000042699	Dhx9
ENSMUSG00000042712	Tceal9
ENSMUSG00000042719	Naa25
ENSMUSG00000042742	Bmt2
ENSMUSG00000042744	Hectd4
ENSMUSG00000042745	Id1
ENSMUSG00000042790	Rnf214
ENSMUSG00000042810	Krba1
ENSMUSG00000042812	Foxf1
ENSMUSG00000042814	Mcts2
ENSMUSG00000042851	Zc3h6
ENSMUSG00000042874	D930007J09Rik
ENSMUSG00000042903	Foxo4
ENSMUSG00000042978	Sbk1
ENSMUSG00000042988	Notum

ENSMUSG00000042992	Borcs5
ENSMUSG00000042997	Nhlrc3
ENSMUSG00000043013	Onecut1
ENSMUSG00000043019	Edem3
ENSMUSG00000043059	Zfp513
ENSMUSG00000043067	Dpy19l1
ENSMUSG00000043085	Tmem82
ENSMUSG00000043090	Zfp866
ENSMUSG00000043091	Tuba1c
ENSMUSG00000043099	Hic1
ENSMUSG00000043140	Tmem186
ENSMUSG00000043154	Ppp2r3a
ENSMUSG00000043190	Rfesd
ENSMUSG00000043241	Upf2
ENSMUSG00000043252	Tmem64
ENSMUSG00000043257	Pigv
ENSMUSG00000043279	Trim56
ENSMUSG00000043284	Tmem11
ENSMUSG00000043295	Gm16373
ENSMUSG00000043311	D17H6S53E
ENSMUSG00000043336	Filip1l
ENSMUSG00000043391	2510009E07Rik
ENSMUSG00000043411	Usp48
ENSMUSG00000043415	Otud1
ENSMUSG00000043421	Hilpda
ENSMUSG00000043424	Eif3j2
ENSMUSG00000043432	Leng9
ENSMUSG00000043439	Epop
ENSMUSG00000043445	Pgp
ENSMUSG00000043483	Gm6863
ENSMUSG00000043535	Setx
ENSMUSG00000043587	Pxylp1
ENSMUSG00000043633	Fam221b
ENSMUSG00000043681	Fam25c
ENSMUSG00000043683	Fem1a
ENSMUSG00000043687	1190005I06Rik
ENSMUSG00000043702	Pde12
ENSMUSG00000043733	Ptpn11
ENSMUSG00000043789	Vwce
ENSMUSG00000043831	Lysmd4
ENSMUSG00000043881	Kbtbd7
ENSMUSG00000043885	Slc36a4
ENSMUSG00000043895	S1pr2
ENSMUSG00000043940	Wdfy3

ENSMUSG00000043962	Thrap3
ENSMUSG00000043964	Orai3
ENSMUSG00000043991	Pura
ENSMUSG00000043998	Mgat2
ENSMUSG00000044005	Gls2
ENSMUSG00000044018	Mrpl50
ENSMUSG00000044026	Slc35g1
ENSMUSG00000044030	Irf2bpl
ENSMUSG00000044033	Ccdc141
ENSMUSG00000044037	Als2cl
ENSMUSG00000044066	Cep68
ENSMUSG00000044068	Zrsr1
ENSMUSG00000044080	S100a1
ENSMUSG00000044098	Rsbn1
ENSMUSG00000044134	Pheta1
ENSMUSG00000044147	Arf6
ENSMUSG00000044167	Foxo1
ENSMUSG00000044197	Gpr146
ENSMUSG00000044206	Vsig4
ENSMUSG00000044224	Dnajc21
ENSMUSG00000044229	Nxpe4
ENSMUSG00000044252	Osbpl1a
ENSMUSG00000044254	Pcsk9
ENSMUSG00000044268	Gm4895
ENSMUSG00000044279	Crb3
ENSMUSG00000044285	Gm1821
ENSMUSG00000044308	Ubr3
ENSMUSG00000044339	Alkbh2
ENSMUSG00000044340	Phlpp1
ENSMUSG00000044345	Marveld1
ENSMUSG00000044361	BC024139
ENSMUSG00000044393	Dsg2
ENSMUSG00000044433	Camsap3
ENSMUSG00000044447	Dock5
ENSMUSG00000044452	Zfp507
ENSMUSG00000044456	Rin3
ENSMUSG00000044465	Fam160a2
ENSMUSG00000044468	Tent5c
ENSMUSG00000044477	Zfand3
ENSMUSG00000044496	2510039O18Rik
ENSMUSG00000044501	Zfp758
ENSMUSG00000044502	Bod1
ENSMUSG00000044548	Dact1
ENSMUSG00000044562	Rasip1

ENSMUSG00000044573	Acp1
ENSMUSG00000044617	Zbtb39
ENSMUSG00000044641	Pard6b
ENSMUSG00000044674	Fzd1
ENSMUSG00000044676	Zfp612
ENSMUSG00000044700	Tmem201
ENSMUSG00000044709	Gemin7
ENSMUSG00000044715	Gskip
ENSMUSG00000044749	Abca6
ENSMUSG00000044763	Trmt10c
ENSMUSG00000044768	D1Ertd622e
ENSMUSG00000044783	Hjurp
ENSMUSG00000044786	Zfp36
ENSMUSG00000044788	Fads6
ENSMUSG00000044791	Setd2
ENSMUSG00000044813	Shb
ENSMUSG00000044881	Coa4
ENSMUSG00000044950	Pwwp2a
ENSMUSG00000044982	Sft2d3
ENSMUSG00000044986	Tst
ENSMUSG00000044991	Shld1
ENSMUSG00000045005	Fzd5
ENSMUSG00000045038	Prkce
ENSMUSG00000045039	Megf8
ENSMUSG00000045087	S1pr5
ENSMUSG00000045092	S1pr1
ENSMUSG00000045094	Arhgef37
ENSMUSG00000045095	Magi1
ENSMUSG00000045098	Kmt5b
ENSMUSG00000045103	Dmd
ENSMUSG00000045107	Saysd1
ENSMUSG00000045176	Borcs6
ENSMUSG00000045180	Shroom2
ENSMUSG00000045193	Cirbp
ENSMUSG00000045210	Vcpip1
ENSMUSG00000045216	Hs6st1
ENSMUSG00000045237	1110012L19Rik
ENSMUSG00000045248	Med26
ENSMUSG00000045251	Zfp688
ENSMUSG00000045252	Zfp574
ENSMUSG00000045268	Zfp691
ENSMUSG00000045287	Rtn4rl1
ENSMUSG00000045294	Insig1
ENSMUSG00000045302	Preb

ENSMUSG00000045312	Lhfpl2
ENSMUSG00000045316	Fahd1
ENSMUSG00000045374	Wdr81
ENSMUSG00000045377	Tmem88
ENSMUSG00000045392	Olfr1033
ENSMUSG00000045410	Akr1e1
ENSMUSG00000045411	2410002F23Rik
ENSMUSG00000045414	Dipk2a
ENSMUSG00000045427	Hnrnph2
ENSMUSG00000045438	Cox19
ENSMUSG00000045441	Gprin3
ENSMUSG00000045455	Gm9797
ENSMUSG00000045482	Trrap
ENSMUSG00000045503	Sys1
ENSMUSG00000045538	Ddx28
ENSMUSG00000045551	Fpr1
ENSMUSG00000045576	St7l
ENSMUSG00000045594	Glb1
ENSMUSG00000045598	Zfp553
ENSMUSG00000045624	Esf1
ENSMUSG00000045636	Mtus1
ENSMUSG00000045639	Zfp629
ENSMUSG00000045658	Pid1
ENSMUSG00000045664	Cdc42ep2
ENSMUSG00000045665	Mfsd5
ENSMUSG00000045671	Spred2
ENSMUSG00000045691	Thtpa
ENSMUSG00000045730	Adrb2
ENSMUSG00000045752	Tssc4
ENSMUSG00000045763	Basp1
ENSMUSG00000045767	B230219D22Rik
ENSMUSG00000045775	Slc16a5
ENSMUSG00000045795	Whamm
ENSMUSG00000045799	Gm9800
ENSMUSG00000045817	Zfp36l2
ENSMUSG00000045854	Lyrm2
ENSMUSG00000045930	Clec14a
ENSMUSG00000045932	Ifit2
ENSMUSG00000045948	Mrps12
ENSMUSG00000045954	Cavin2
ENSMUSG00000045962	Wnk1
ENSMUSG00000045968	Teddm2
ENSMUSG00000045969	Ing1
ENSMUSG00000045973	Slc25a51

ENSMUSG00000045975	C2cd2
ENSMUSG00000045980	Tmem104
ENSMUSG00000045983	Eif4g1
ENSMUSG00000045991	Onecut2
ENSMUSG00000046010	Zfp830
ENSMUSG00000046020	Pofut1
ENSMUSG00000046027	Stard5
ENSMUSG00000046034	Otulin
ENSMUSG00000046062	Ppp1r15b
ENSMUSG00000046070	Igfals
ENSMUSG00000046138	9930021J03Rik
ENSMUSG00000046185	Zfp84
ENSMUSG00000046201	Scaf8
ENSMUSG00000046229	Scand1
ENSMUSG00000046280	She
ENSMUSG00000046311	Zfp62
ENSMUSG00000046312	Myorg
ENSMUSG00000046318	Ccbe1
ENSMUSG00000046324	Ermp1
ENSMUSG00000046329	Slc25a23
ENSMUSG00000046341	Gm11223
ENSMUSG00000046351	Zfp322a
ENSMUSG00000046352	Gjb2
ENSMUSG00000046402	Rbp1
ENSMUSG00000046404	Yod1
ENSMUSG00000046447	Camk2n1
ENSMUSG00000046470	Sox18
ENSMUSG00000046516	Cox17
ENSMUSG00000046519	Golph3l
ENSMUSG00000046541	Zfp526
ENSMUSG00000046546	Fam43a
ENSMUSG00000046562	Unc119b
ENSMUSG00000046573	Lyrm4
ENSMUSG00000046574	Prr12
ENSMUSG00000046580	Gm7862
ENSMUSG00000046598	Bdh1
ENSMUSG00000046668	Cxxc5
ENSMUSG00000046675	Tmem251
ENSMUSG00000046679	C87436
ENSMUSG00000046688	Tifa
ENSMUSG00000046691	Chtf8
ENSMUSG00000046707	Csnk2a2
ENSMUSG00000046721	Rpl14-ps1
ENSMUSG00000046722	Cdc42se1

ENSMUSG00000046753	Ccdc66
ENSMUSG00000046756	Mrps7
ENSMUSG00000046761	Fam83h
ENSMUSG00000046785	Epm2aip1
ENSMUSG00000046791	Riox1
ENSMUSG00000046794	Ppp1r3b
ENSMUSG00000046798	Cldn12
ENSMUSG00000046805	Mpeg1
ENSMUSG00000046806	Cyren
ENSMUSG00000046811	Gltpd2
ENSMUSG00000046814	Gchfr
ENSMUSG00000046822	Slc39a3
ENSMUSG00000046836	Brox
ENSMUSG00000046841	Ckap4
ENSMUSG00000046862	Pramef8
ENSMUSG00000046879	Irgm1
ENSMUSG00000046897	Zfp740
ENSMUSG00000046909	Tefm
ENSMUSG00000046916	Myct1
ENSMUSG00000046942	Mageb16
ENSMUSG00000046947	Adck2
ENSMUSG00000046949	Nqo2
ENSMUSG00000046959	Slc26a1
ENSMUSG00000046962	Zbtb21
ENSMUSG00000046982	Tshz1
ENSMUSG00000046985	Tapt1
ENSMUSG00000046994	Mars2
ENSMUSG00000047022	Mipol1
ENSMUSG00000047030	Spata2
ENSMUSG00000047036	Zfp445
ENSMUSG00000047044	D030056L22Rik
ENSMUSG00000047067	Dusp28
ENSMUSG00000047084	Ngrn
ENSMUSG00000047109	Cldn14
ENSMUSG00000047123	Ticam1
ENSMUSG00000047126	Cltc
ENSMUSG00000047141	Zfp654
ENSMUSG00000047153	Khnyn
ENSMUSG00000047180	Neurl3
ENSMUSG00000047213	Ythdf3
ENSMUSG00000047228	A2ml1
ENSMUSG00000047230	Cldn2
ENSMUSG00000047250	Ptgs1
ENSMUSG00000047260	Emc6

ENSMUSG00000047370	Gm7367
ENSMUSG00000047371	Zfp768
ENSMUSG00000047379	B4gat1
ENSMUSG00000047388	Atmin
ENSMUSG00000047409	Ctdspl
ENSMUSG00000047412	Zbtb44
ENSMUSG00000047417	Rexo1
ENSMUSG00000047423	AI837181
ENSMUSG00000047434	Xxylt1
ENSMUSG00000047446	Arl4a
ENSMUSG00000047459	Dynlrb1
ENSMUSG00000047492	Inhbe
ENSMUSG00000047496	Rnf152
ENSMUSG00000047507	Baiap3
ENSMUSG00000047514	Tspyl1
ENSMUSG00000047539	Fbxo28
ENSMUSG00000047547	Cltb
ENSMUSG00000047604	Frat2
ENSMUSG00000047613	A430005L14Rik
ENSMUSG00000047631	Apof
ENSMUSG00000047635	2810006K23Rik
ENSMUSG00000047638	Nr1h4
ENSMUSG00000047648	Fbxo30
ENSMUSG00000047649	Cd3eap
ENSMUSG00000047719	Ubiad1
ENSMUSG00000047721	Bola2
ENSMUSG00000047728	Ly6g2
ENSMUSG00000047731	Wbp11
ENSMUSG00000047735	Samd9l
ENSMUSG00000047777	Phf13
ENSMUSG00000047793	Sned1
ENSMUSG00000047797	Gjb1
ENSMUSG00000047804	Akap10
ENSMUSG00000047822	Angptl8
ENSMUSG00000047824	Pygo2
ENSMUSG00000047832	Cdca4
ENSMUSG00000047867	Gimap6
ENSMUSG00000047875	Gpr157
ENSMUSG00000047879	Usp14
ENSMUSG00000047881	Rell1
ENSMUSG00000047888	Tnrc6b
ENSMUSG00000047905	Gm8566
ENSMUSG00000047907	Tshz2
ENSMUSG00000047909	Ankrd16

ENSMUSG00000047921	Trappc9
ENSMUSG00000047945	Marcksl1
ENSMUSG00000047963	Stbd1
ENSMUSG00000048000	Gigyf2
ENSMUSG00000048027	Rgmb
ENSMUSG00000048039	Isg20l2
ENSMUSG00000048058	Ldlrad3
ENSMUSG00000048076	Arf1
ENSMUSG00000048106	4632415L05Rik
ENSMUSG00000048109	Rbm15
ENSMUSG00000048118	Arid4a
ENSMUSG00000048120	Entpd1
ENSMUSG00000048154	Kmt2d
ENSMUSG00000048175	Asb8
ENSMUSG00000048217	Nags
ENSMUSG00000048218	Amigo2
ENSMUSG00000048234	Rnf149
ENSMUSG00000048249	Crebrf
ENSMUSG00000048264	Dip2c
ENSMUSG00000048271	Rbm33
ENSMUSG00000048277	Syngr2
ENSMUSG00000048310	Pskh1
ENSMUSG00000048334	Gm8258
ENSMUSG00000048351	Coa7
ENSMUSG00000048368	Omd
ENSMUSG00000048371	Pdp2
ENSMUSG00000048376	F2r
ENSMUSG00000048379	Socs4
ENSMUSG00000048429	Timm29
ENSMUSG00000048486	Fitm2
ENSMUSG00000048489	Depp1
ENSMUSG00000048490	Nrip1
ENSMUSG00000048495	Tyw5
ENSMUSG00000048520	Fbxl13
ENSMUSG00000048546	Tob2
ENSMUSG00000048578	Mlec
ENSMUSG00000048581	E130311K13Rik
ENSMUSG00000048696	Mex3d
ENSMUSG00000048701	Ccdc6
ENSMUSG00000048706	Lurap1l
ENSMUSG00000048709	Gm8666
ENSMUSG00000048755	Mcat
ENSMUSG00000048756	Foxo3
ENSMUSG00000048772	Tmem53

ENSMUSG00000048787	Dcun1d3
ENSMUSG00000048799	Cep120
ENSMUSG00000048826	Dact2
ENSMUSG00000048827	Pkd1l3
ENSMUSG00000048832	Vps37c
ENSMUSG00000048833	Slc39a9
ENSMUSG00000048856	Slc25a47
ENSMUSG00000048874	Phf3
ENSMUSG00000048878	Hexim1
ENSMUSG00000048897	Zfp710
ENSMUSG00000048911	Rnf24
ENSMUSG00000048920	Fkrp
ENSMUSG00000048924	Ccdc125
ENSMUSG00000048938	Nr1h5
ENSMUSG00000048970	C1galt1c1
ENSMUSG00000049044	Rapgef4
ENSMUSG00000049047	Armcx3
ENSMUSG00000049086	Bmyc
ENSMUSG00000049090	Zadh2
ENSMUSG00000049091	Sephs2
ENSMUSG00000049106	Dcaf5
ENSMUSG00000049115	Agtr1a
ENSMUSG00000049152	Ugt3a2
ENSMUSG00000049164	Zfp518a
ENSMUSG00000049231	Gm12497
ENSMUSG00000049232	Tigd2
ENSMUSG00000049287	Iba57
ENSMUSG00000049295	Zfp219
ENSMUSG00000049300	Prmt6
ENSMUSG00000049305	Ccdc71
ENSMUSG00000049323	Smcr8
ENSMUSG00000049327	Kmt5a
ENSMUSG00000049339	Retreg2
ENSMUSG00000049354	Dcaf7
ENSMUSG00000049396	Gemin4
ENSMUSG00000049401	Ogfr
ENSMUSG00000049421	Zfp260
ENSMUSG00000049422	Chchd10
ENSMUSG00000049470	Aff4
ENSMUSG00000049489	Ccnq
ENSMUSG00000049502	Dtx3l
ENSMUSG00000049516	Spty2d1
ENSMUSG00000049521	Cdc42ep1
ENSMUSG00000049550	Clip1

ENSMUSG00000049553	Polr1a
ENSMUSG00000049555	Tmie
ENSMUSG00000049577	Zfpm1
ENSMUSG00000049580	Tsku
ENSMUSG00000049643	2310022A10Rik
ENSMUSG00000049657	Zbtb5
ENSMUSG00000049659	Aftph
ENSMUSG00000049672	Zbtb14
ENSMUSG00000049680	Urgcp
ENSMUSG00000049686	Orai1
ENSMUSG00000049734	Trex1
ENSMUSG00000049739	Zfp646
ENSMUSG00000049751	Rpl36al
ENSMUSG00000049764	Zfp280b
ENSMUSG00000049775	Tmsb4x
ENSMUSG00000049791	Fzd4
ENSMUSG00000049792	Bag5
ENSMUSG00000049800	Sertad2
ENSMUSG00000049807	Arhgap23
ENSMUSG00000049858	Suox
ENSMUSG00000049866	Arl4c
ENSMUSG00000049878	Rlf
ENSMUSG00000049892	Rasd1
ENSMUSG00000049922	Slc35c1
ENSMUSG00000049932	H2afx
ENSMUSG00000049940	Pgrmc2
ENSMUSG00000049950	Rpp38
ENSMUSG00000049969	Plekhf2
ENSMUSG00000049971	Glt1d1
ENSMUSG00000050029	Rap2c
ENSMUSG00000050052	Tdrp
ENSMUSG00000050069	Grem2
ENSMUSG00000050088	1600012H06Rik
ENSMUSG00000050144	Slc25a44
ENSMUSG00000050148	Ubqln2
ENSMUSG00000050174	Nudt6
ENSMUSG00000050188	Lsm10
ENSMUSG00000050199	Lgr4
ENSMUSG00000050212	Eva1b
ENSMUSG00000050213	Snip1
ENSMUSG00000050229	Pigm
ENSMUSG00000050234	Gja4
ENSMUSG00000050310	Rictor
ENSMUSG00000050373	Snx21

ENSMUSG00000050390	C77080
ENSMUSG00000050423	Ppp1r3g
ENSMUSG00000050428	Fbxo46
ENSMUSG00000050440	Hamp
ENSMUSG00000050445	Cyp8b1
ENSMUSG00000050493	Fam167b
ENSMUSG00000050530	Fam171a1
ENSMUSG00000050541	Adra1b
ENSMUSG00000050552	Lamtor4
ENSMUSG00000050555	Hyls1
ENSMUSG00000050565	Tor1aip2
ENSMUSG00000050567	Maml1
ENSMUSG00000050608	Micos10
ENSMUSG00000050619	Zscan29
ENSMUSG00000050628	Ubald2
ENSMUSG00000050705	2310061I04Rik
ENSMUSG00000050721	Plekho2
ENSMUSG00000050730	Arhgap42
ENSMUSG00000050732	Vamp8
ENSMUSG00000050777	Tmem37
ENSMUSG00000050796	B3galt6
ENSMUSG00000050812	Ecpas
ENSMUSG00000050846	Zfp623
ENSMUSG00000050854	Tmem125
ENSMUSG00000050860	Phospho1
ENSMUSG00000050890	Pdik1l
ENSMUSG00000050900	Gm7327
ENSMUSG00000050910	Cdr2l
ENSMUSG00000050931	Sgms2
ENSMUSG00000050947	Amigo1
ENSMUSG00000050953	Gja1
ENSMUSG00000050973	Gdpgp1
ENSMUSG00000050974	Gm9847
ENSMUSG00000051041	Olfml1
ENSMUSG00000051043	Gprc5c
ENSMUSG00000051065	Mb21d2
ENSMUSG00000051124	Gimap9
ENSMUSG00000051147	Nat2
ENSMUSG00000051149	Adnp
ENSMUSG00000051169	Rpusd3
ENSMUSG00000051238	Swsap1
ENSMUSG00000051285	Pcmtd1
ENSMUSG00000051306	Usp42
ENSMUSG00000051319	Mtln

ENSMUSG00000051339	2900026A02Rik
ENSMUSG00000051346	Spryd4
ENSMUSG00000051355	Commd1
ENSMUSG00000051359	Ncald
ENSMUSG00000051375	Pcdh1
ENSMUSG00000051390	Zbtb22
ENSMUSG00000051391	Ywhag
ENSMUSG00000051403	Ppp1r37
ENSMUSG00000051427	Ccdc157
ENSMUSG00000051439	Cd14
ENSMUSG00000051451	Crebzf
ENSMUSG00000051469	Zfp24
ENSMUSG00000051483	Cbr1
ENSMUSG00000051495	Irf2bp2
ENSMUSG00000051510	Mafg
ENSMUSG00000051518	Rps19bp1
ENSMUSG00000051548	Gm6365
ENSMUSG00000051550	Zfp579
ENSMUSG00000051579	Tceal8
ENSMUSG00000051586	Mical3
ENSMUSG00000051615	Rap2a
ENSMUSG00000051639	Gm5812
ENSMUSG00000051650	B3gnt2
ENSMUSG00000051652	Lrrc3
ENSMUSG00000051671	Coa6
ENSMUSG00000051674	Dcun1d4
ENSMUSG00000051695	Pcbp1
ENSMUSG00000051716	Apon
ENSMUSG00000051817	Sox12
ENSMUSG00000051853	Arf3
ENSMUSG00000051910	Sox6
ENSMUSG00000051977	Prdm9
ENSMUSG00000052031	Tagap1
ENSMUSG00000052033	Pfdn4
ENSMUSG00000052040	Klf13
ENSMUSG00000052056	Zfp217
ENSMUSG00000052085	Dock8
ENSMUSG00000052117	D630039A03Rik
ENSMUSG00000052137	Rbm12b2
ENSMUSG00000052144	Ppp4r2
ENSMUSG00000052151	Plpp2
ENSMUSG00000052155	Acvr2a
ENSMUSG00000052214	Opa3
ENSMUSG00000052253	Zfp622

ENSMUSG00000052296	Ppp6r1
ENSMUSG00000052302	Tbc1d30
ENSMUSG00000052305	Hbb-bs
ENSMUSG00000052310	Slc39a1
ENSMUSG00000052384	Nrros
ENSMUSG00000052387	Trpm3
ENSMUSG00000052392	Acot4
ENSMUSG00000052395	Rft1
ENSMUSG00000052403	Fcnaos
ENSMUSG00000052406	Rexo4
ENSMUSG00000052414	Gm28047
ENSMUSG00000052419	2610001J05Rik
ENSMUSG00000052428	Tmco1
ENSMUSG00000052435	Cebpe
ENSMUSG00000052446	Zfp961
ENSMUSG00000052456	Asna1
ENSMUSG00000052504	Epha3
ENSMUSG00000052512	Nav2
ENSMUSG00000052520	Cyp2j5
ENSMUSG00000052539	Magi3
ENSMUSG00000052562	Slc22a30
ENSMUSG00000052593	Adam17
ENSMUSG00000052595	A1cf
ENSMUSG00000052609	Plekhg3
ENSMUSG00000052656	Rnf103
ENSMUSG00000052673	Gm9887
ENSMUSG00000052681	Rap1b
ENSMUSG00000052684	Jun
ENSMUSG00000052707	Tnrc6a
ENSMUSG00000052712	BC004004
ENSMUSG00000052738	Suclg1
ENSMUSG00000052751	Repin1
ENSMUSG00000052763	Zfp212
ENSMUSG00000052812	Atad2b
ENSMUSG00000052825	Gm9892
ENSMUSG00000052837	Junb
ENSMUSG00000052906	Ubxn8
ENSMUSG00000052914	Cyp2j6
ENSMUSG00000052915	Msl1
ENSMUSG00000052920	Prkg1
ENSMUSG00000052921	Arhgef15
ENSMUSG00000052928	Ctif
ENSMUSG00000052934	Fbxo31
ENSMUSG00000052957	Gas1

ENSMUSG00000053012	Krcc1
ENSMUSG00000053094	Tmem248
ENSMUSG00000053110	Yap1
ENSMUSG00000053113	Socs3
ENSMUSG00000053119	Chmp3
ENSMUSG00000053128	Rnf26
ENSMUSG00000053175	Bcl3
ENSMUSG00000053205	Styx
ENSMUSG00000053289	Ddx10
ENSMUSG00000053293	Pom121
ENSMUSG00000053329	Gatd3a
ENSMUSG00000053334	Ficd
ENSMUSG00000053347	Zfp943
ENSMUSG00000053411	Cbx7
ENSMUSG00000053436	Mapk14
ENSMUSG00000053469	Tg
ENSMUSG00000053470	Kdm3a
ENSMUSG00000053477	Tcf4
ENSMUSG00000053510	Nrd1
ENSMUSG00000053536	Cstf2t
ENSMUSG00000053553	3110082I17Rik
ENSMUSG00000053559	Smagp
ENSMUSG00000053560	Ier2
ENSMUSG00000053581	Zfand2a
ENSMUSG00000053588	A730061H03Rik
ENSMUSG00000053600	Zfp472
ENSMUSG00000053613	Notumos
ENSMUSG00000053617	Sh3pxd2a
ENSMUSG00000053646	Plxnb1
ENSMUSG00000053684	BC048403
ENSMUSG00000053693	Mast1
ENSMUSG00000053714	4732471J01Rik
ENSMUSG00000053716	Dusp7
ENSMUSG00000053740	Gm6457
ENSMUSG00000053749	Gm9920
ENSMUSG00000053754	Chd8
ENSMUSG00000053768	Chchd3
ENSMUSG00000053799	Exoc6
ENSMUSG00000053801	Grwd1
ENSMUSG00000053835	H2-T24
ENSMUSG00000053838	Nudcd3
ENSMUSG00000053841	Txlna
ENSMUSG00000053846	Lipg
ENSMUSG00000053870	Fpgt

ENSMUSG00000053877	Srcap
ENSMUSG00000053886	Sh2d4a
ENSMUSG00000053907	Mat2a
ENSMUSG00000053929	Cyhr1
ENSMUSG00000053931	Cnn3
ENSMUSG00000053950	Adnp2
ENSMUSG00000054008	Ndst1
ENSMUSG00000054027	Nt5dc3
ENSMUSG00000054051	Ercc6
ENSMUSG00000054072	Iigp1
ENSMUSG00000054150	Syne3
ENSMUSG00000054191	Klf1
ENSMUSG00000054199	Gon4l
ENSMUSG00000054263	Lifr
ENSMUSG00000054364	Rhob
ENSMUSG00000054381	Zfp747
ENSMUSG00000054387	Mdm4
ENSMUSG00000054404	Slfn5
ENSMUSG00000054405	Dnajc8
ENSMUSG00000054414	Slc30a7
ENSMUSG00000054422	Fabp1
ENSMUSG00000054426	A930005H10Rik
ENSMUSG00000054428	Atpif1
ENSMUSG00000054434	Tmem120b
ENSMUSG00000054435	Gimap4
ENSMUSG00000054452	Aes
ENSMUSG00000054455	Vapb
ENSMUSG00000054469	Lclat1
ENSMUSG00000054477	Kcnn2
ENSMUSG00000054484	Tmem62
ENSMUSG00000054499	Dedd2
ENSMUSG00000054509	Parp4
ENSMUSG00000054520	Sh3bp2
ENSMUSG00000054545	Ugt1a6a
ENSMUSG00000054604	Cggbp1
ENSMUSG00000054611	Kdm2a
ENSMUSG00000054619	Mettl7a1
ENSMUSG00000054630	Ugt2b5
ENSMUSG00000054648	Zfp869
ENSMUSG00000054676	1600014C10Rik
ENSMUSG00000054693	Adam10
ENSMUSG00000054715	Zscan22
ENSMUSG00000054716	Zfp771
ENSMUSG00000054720	Lrrc8c

ENSMUSG00000054723	Vmac
ENSMUSG00000054733	Msra
ENSMUSG00000054757	Akr1c20
ENSMUSG00000054766	Set
ENSMUSG00000054792	Klhl18
ENSMUSG00000054808	Actn4
ENSMUSG00000054814	Usp46
ENSMUSG00000054823	Nsd3
ENSMUSG00000054827	Cyp2c50
ENSMUSG00000054843	Atrnl1
ENSMUSG00000054855	Rnd1
ENSMUSG00000054874	Pcnx3
ENSMUSG00000054889	Dsp
ENSMUSG00000054920	Klhl5
ENSMUSG00000055024	Ep300
ENSMUSG00000055041	Commd5
ENSMUSG00000055053	Nfic
ENSMUSG00000055065	Ddx17
ENSMUSG00000055148	Klf2
ENSMUSG00000055172	C1ra
ENSMUSG00000055200	Sertad3
ENSMUSG00000055204	Ankrd17
ENSMUSG00000055239	Kcmf1
ENSMUSG00000055301	Adh7
ENSMUSG00000055302	Mrfap1
ENSMUSG00000055322	Tns1
ENSMUSG00000055435	Maf
ENSMUSG00000055436	Srsf11
ENSMUSG00000055491	Pprc1
ENSMUSG00000055538	Zcchc24
ENSMUSG00000055632	Hmcn2
ENSMUSG00000055652	Klhl25
ENSMUSG00000055670	Zzef1
ENSMUSG00000055681	Cope
ENSMUSG00000055737	Ghr
ENSMUSG00000055760	Gemin6
ENSMUSG00000055763	Rybp-ps
ENSMUSG00000055771	Gm7936
ENSMUSG00000055782	Abcd2
ENSMUSG00000055818	A230083G16Rik
ENSMUSG00000055835	Zfp1
ENSMUSG00000055839	Elob
ENSMUSG00000055862	Izumo4
ENSMUSG00000055866	Per2

ENSMUSG00000055897	Ppp4r1l-ps
ENSMUSG00000055912	Tmem150a
ENSMUSG00000055980	Irs1
ENSMUSG00000055991	Zkscan5
ENSMUSG00000056014	A430033K04Rik
ENSMUSG00000056050	Mia3
ENSMUSG00000056069	Otulinl
ENSMUSG00000056076	Eif3b
ENSMUSG00000056091	St3gal5
ENSMUSG00000056116	H2-T22
ENSMUSG00000056121	Fez2
ENSMUSG00000056148	Rdh9
ENSMUSG00000056153	Socs6
ENSMUSG00000056167	Cnot10
ENSMUSG00000056204	Pgpep1
ENSMUSG00000056216	Cebpg
ENSMUSG00000056305	Usp39
ENSMUSG00000056313	Tcim
ENSMUSG00000056342	Usp34
ENSMUSG00000056383	AI987944
ENSMUSG00000056429	Tgoln1
ENSMUSG00000056492	Adgrf5
ENSMUSG00000056493	Foxk1
ENSMUSG00000056501	Cebpb
ENSMUSG00000056515	Rab31
ENSMUSG00000056537	Rlim
ENSMUSG00000056579	Tug1
ENSMUSG00000056608	Chd9
ENSMUSG00000056612	Ppp1r14b
ENSMUSG00000056629	Fkbp2
ENSMUSG00000056643	Chst13
ENSMUSG00000056692	Ilrun
ENSMUSG00000056708	Ier5
ENSMUSG00000056749	Nfil3
ENSMUSG00000056770	Setd3
ENSMUSG00000056836	Gm6851
ENSMUSG00000056917	Sipa1
ENSMUSG00000056938	Acbd4
ENSMUSG00000056952	Tatdn2
ENSMUSG00000056962	Jmjd6
ENSMUSG00000056978	Hamp2
ENSMUSG00000056999	Ide
ENSMUSG00000057037	Cfhr1
ENSMUSG00000057069	Ero1lb

ENSMUSG00000057074	Ces1g
ENSMUSG00000057101	Zfp180
ENSMUSG00000057103	Nat8f1
ENSMUSG00000057130	Txnl4a
ENSMUSG00000057133	Chd6
ENSMUSG00000057134	Ado
ENSMUSG00000057176	Ccdc189
ENSMUSG00000057177	Gsk3a
ENSMUSG00000057228	Aadat
ENSMUSG00000057229	Dmac2
ENSMUSG00000057230	Aak1
ENSMUSG00000057265	Bbof1
ENSMUSG00000057342	Sphk2
ENSMUSG00000057363	Uxs1
ENSMUSG00000057367	Birc2
ENSMUSG00000057388	Mrpl18
ENSMUSG00000057400	Ces1c
ENSMUSG00000057411	Fam173a
ENSMUSG00000057497	Fam136a
ENSMUSG00000057522	Spop
ENSMUSG00000057530	Ece1
ENSMUSG00000057531	Dtnbp1
ENSMUSG00000057551	Zfp317
ENSMUSG00000057554	Lgals8
ENSMUSG00000057561	Eif1a
ENSMUSG00000057572	Zbtb8os
ENSMUSG00000057594	Arl16
ENSMUSG00000057637	Prdm2
ENSMUSG00000057667	Bloc1s3
ENSMUSG00000057672	Pkn1
ENSMUSG00000057691	Zfp746
ENSMUSG00000057789	Bak1
ENSMUSG00000057880	Abat
ENSMUSG00000057963	Itpk1
ENSMUSG00000057982	Zfp809
ENSMUSG00000058022	Adtrp
ENSMUSG00000058056	Palld
ENSMUSG00000058073	Gm11189
ENSMUSG00000058126	Tpm3-rs7
ENSMUSG00000058135	Gstm1
ENSMUSG00000058152	Chsy3
ENSMUSG00000058207	Serpina3k
ENSMUSG00000058230	Arhgap35
ENSMUSG00000058239	Usf2

ENSMUSG00000058291	Zfp68
ENSMUSG00000058298	Mcm9
ENSMUSG00000058301	Upf1
ENSMUSG00000058318	Phf21a
ENSMUSG00000058388	Phtf1
ENSMUSG00000058396	Gpr182
ENSMUSG00000058446	Znrf2
ENSMUSG00000058454	Dhcr7
ENSMUSG00000058486	Wdr91
ENSMUSG00000058569	Tmed9
ENSMUSG00000058594	Fbh1
ENSMUSG00000058620	Adra2b
ENSMUSG00000058624	Gda
ENSMUSG00000058638	Zfp110
ENSMUSG00000058655	Eif4b
ENSMUSG00000058672	Tubb2a
ENSMUSG00000058690	Ccser2
ENSMUSG00000058709	Egln2
ENSMUSG00000058748	Zfp958
ENSMUSG00000058756	Thra
ENSMUSG00000058761	Rnf169
ENSMUSG00000058833	Rex1bd
ENSMUSG00000058835	Abi1
ENSMUSG00000058883	Zfp708
ENSMUSG00000058905	Gm10051
ENSMUSG00000058921	Slc10a5
ENSMUSG00000058922	Gm10052
ENSMUSG00000058927	Gm10053
ENSMUSG00000058952	Cfi
ENSMUSG00000058979	Hdhd5
ENSMUSG00000058997	Vwa8
ENSMUSG00000059013	Sh2d3c
ENSMUSG00000059031	Olfr482
ENSMUSG00000059040	Eno1b
ENSMUSG00000059058	Tma7-ps
ENSMUSG00000059142	Zfp945
ENSMUSG00000059183	Mtfmt
ENSMUSG00000059195	Gm12715
ENSMUSG00000059208	Hnrnpm
ENSMUSG00000059237	Ppp1r2-ps4
ENSMUSG00000059263	Usp47
ENSMUSG00000059273	Zc3h4
ENSMUSG00000059278	Naa38
ENSMUSG00000059316	Slc27a4

ENSMUSG00000059325	Hopx
ENSMUSG00000059409	Ppp2r5d
ENSMUSG00000059434	Gckr
ENSMUSG00000059436	Max
ENSMUSG00000059456	Ptk2b
ENSMUSG00000059475	Zfp426
ENSMUSG00000059479	B3gnt8
ENSMUSG00000059481	Plg
ENSMUSG00000059486	Kbtbd2
ENSMUSG00000059495	Arhgef12
ENSMUSG00000059498	Fcgr3
ENSMUSG00000059518	Znhit1
ENSMUSG00000059555	Tor4a
ENSMUSG00000059588	Calcrl
ENSMUSG00000059689	Zfp637
ENSMUSG00000059772	Slx1b
ENSMUSG00000059811	Atl2
ENSMUSG00000059820	Nkapd1
ENSMUSG00000059824	Dbp
ENSMUSG00000059851	Kmt5c
ENSMUSG00000059878	Zfp422
ENSMUSG00000059883	Irak4
ENSMUSG00000059890	Ube4a
ENSMUSG00000059895	Ptp4a3
ENSMUSG00000059908	Mug1
ENSMUSG00000059920	4930453N24Rik
ENSMUSG00000059923	Grb2
ENSMUSG00000059939	9430015G10Rik
ENSMUSG00000059981	Taok2
ENSMUSG00000059995	Atxn7l3
ENSMUSG00000060002	Chpt1
ENSMUSG00000060012	Kif13b
ENSMUSG00000060143	Gm10076
ENSMUSG00000060149	BC002059
ENSMUSG00000060152	Pop5
ENSMUSG00000060166	Zdhhc8
ENSMUSG00000060181	Slc35e3
ENSMUSG00000060244	Alyref2
ENSMUSG00000060261	Gtf2i
ENSMUSG00000060317	Acnat2
ENSMUSG00000060373	Hnrnpc
ENSMUSG00000060376	Bckdha
ENSMUSG00000060377	Rpl36a-ps1
ENSMUSG00000060407	Cyp2a12

ENSMUSG00000060427	Zfp868
ENSMUSG00000060429	Sntb1
ENSMUSG00000060459	Kng2
ENSMUSG00000060467	Gm10080
ENSMUSG00000060470	Adgrg3
ENSMUSG00000060475	Wtap
ENSMUSG00000060477	Irak2
ENSMUSG00000060510	Zfp266
ENSMUSG00000060512	0610040J01Rik
ENSMUSG00000060519	Tor3a
ENSMUSG00000060550	H2-Q7
ENSMUSG00000060591	Ifitm2
ENSMUSG00000060613	Cyp2c70
ENSMUSG00000060657	Marf1
ENSMUSG00000060708	Bloc1s4
ENSMUSG00000060727	Gm12571
ENSMUSG00000060733	Ipmk
ENSMUSG00000060802	B2m
ENSMUSG00000060807	Serpina6
ENSMUSG00000060938	Rpl26
ENSMUSG00000060992	Copz1
ENSMUSG00000061024	Rrs1
ENSMUSG00000061062	Gm10093
ENSMUSG00000061079	Zfp143
ENSMUSG00000061118	Dnajc30
ENSMUSG00000061130	Ppm1b
ENSMUSG00000061136	Prpf40a
ENSMUSG00000061175	Fnip2
ENSMUSG00000061232	H2-K1
ENSMUSG00000061244	Exoc5
ENSMUSG00000061288	Taok3
ENSMUSG00000061306	Slc38a10
ENSMUSG00000061353	Cxcl12
ENSMUSG00000061360	Phf5a
ENSMUSG00000061374	Fiz1
ENSMUSG00000061410	Zcchc14
ENSMUSG00000061436	Hipk2
ENSMUSG00000061455	Stx17
ENSMUSG00000061589	Dot1l
ENSMUSG00000061650	Med9
ENSMUSG00000061689	Dlgap4
ENSMUSG00000061731	Ext1
ENSMUSG00000061740	Cyp2d22
ENSMUSG00000061751	Kalrn

ENSMUSG00000061755	Bod1l
ENSMUSG00000061759	Armt1
ENSMUSG00000061808	Ttr
ENSMUSG00000061887	Ssbp3
ENSMUSG00000061897	Gm14292
ENSMUSG00000061947	Serpina10
ENSMUSG00000061950	Ppp4r1
ENSMUSG00000061959	Ces1e
ENSMUSG00000061979	Rcc1l
ENSMUSG00000061981	Flot2
ENSMUSG00000062006	Rpl34
ENSMUSG00000062078	Qk
ENSMUSG00000062081	Gm6055
ENSMUSG00000062098	Btbd3
ENSMUSG00000062116	Zfp954
ENSMUSG00000062127	Cttnbp2nl
ENSMUSG00000062181	Ces3b
ENSMUSG00000062202	Btbd9
ENSMUSG00000062203	Gspt1
ENSMUSG00000062232	Rapgef2
ENSMUSG00000062234	Gak
ENSMUSG00000062300	Nectin2
ENSMUSG00000062353	Gm15772
ENSMUSG00000062376	Borcs7
ENSMUSG00000062480	Acat3
ENSMUSG00000062488	Ifit3b
ENSMUSG00000062515	Fabp4
ENSMUSG00000062554	Gm12751
ENSMUSG00000062580	Timm17a
ENSMUSG00000062604	Srpk2
ENSMUSG00000062611	Rps3a2
ENSMUSG00000062619	2310039H08Rik
ENSMUSG00000062822	4833420G17Rik
ENSMUSG00000062866	Phactr2
ENSMUSG00000062901	Klhl24
ENSMUSG00000062908	Acadm
ENSMUSG00000062937	Mtap
ENSMUSG00000062949	Atp11c
ENSMUSG00000062960	Kdr
ENSMUSG00000062963	Ufc1
ENSMUSG00000062980	Cped1
ENSMUSG00000063015	Ccni
ENSMUSG00000063019	Manbal
ENSMUSG00000063049	Ing2

ENSMUSG00000063060	Sox7
ENSMUSG00000063065	Mapk3
ENSMUSG00000063077	Kif1b
ENSMUSG00000063108	Zfp26
ENSMUSG00000063171	Rps4l
ENSMUSG00000063179	Pstk
ENSMUSG00000063232	Serpina11
ENSMUSG00000063235	Ptpmt1
ENSMUSG00000063236	1110038F14Rik
ENSMUSG00000063268	Parp10
ENSMUSG00000063273	Naa15
ENSMUSG00000063281	Zfp35
ENSMUSG00000063358	Mapk1
ENSMUSG00000063362	Alg11
ENSMUSG00000063382	Bcl9l
ENSMUSG00000063388	BC023105
ENSMUSG00000063406	Tmed5
ENSMUSG00000063415	Cyp26b1
ENSMUSG00000063439	B9d2
ENSMUSG00000063450	Syne2
ENSMUSG00000063488	Zkscan7
ENSMUSG00000063511	Snrnp70
ENSMUSG00000063550	Nup98
ENSMUSG00000063659	Zbtb18
ENSMUSG00000063683	Glyat
ENSMUSG00000063727	Tnfrsf11b
ENSMUSG00000063730	Hsd3b2
ENSMUSG00000063760	Rnf217
ENSMUSG00000063765	Chadl
ENSMUSG00000063785	Utp14a
ENSMUSG00000063787	Chchd1
ENSMUSG00000063801	Ap3s2
ENSMUSG00000063838	Cdc42ep5
ENSMUSG00000063849	Ppcdc
ENSMUSG00000063870	Chd4
ENSMUSG00000063882	Uqcrh
ENSMUSG00000063888	Rpl7l1
ENSMUSG00000063889	Crem
ENSMUSG00000063897	CAAA01118383.1
ENSMUSG00000063919	Srrm4
ENSMUSG00000063931	Pepd
ENSMUSG00000063952	Brpf3
ENSMUSG00000064030	Pym1
ENSMUSG00000064068	Mtx1

ENSMUSG00000064105	Cnnm2
ENSMUSG00000064145	Arih2
ENSMUSG00000064181	Rab3ip
ENSMUSG00000064208	Gm10145
ENSMUSG00000064210	Ano6
ENSMUSG00000064215	Ifi27
ENSMUSG00000064225	Paqr9
ENSMUSG00000064262	Gimap8
ENSMUSG00000064280	Ccdc146
ENSMUSG00000064289	Tank
ENSMUSG00000064294	Aox3
ENSMUSG00000064302	Clasp1
ENSMUSG00000064345	mt-Nd2
ENSMUSG00000064363	mt-Nd4
ENSMUSG00000064367	mt-Nd5
ENSMUSG00000064368	mt-Nd6
ENSMUSG00000064370	mt-Cytb
ENSMUSG00000065979	Cpped1
ENSMUSG00000066026	Dhrs3
ENSMUSG00000066037	Hnrnpr
ENSMUSG00000066042	Med18
ENSMUSG00000066043	Phactr4
ENSMUSG00000066071	Cyp4a12a
ENSMUSG00000066148	Prpf4
ENSMUSG00000066151	Fkbp15
ENSMUSG00000066152	Slc31a2
ENSMUSG00000066175	2510046G10Rik
ENSMUSG00000066232	Ipo7
ENSMUSG00000066233	Tmem42
ENSMUSG00000066235	Pomgnt2
ENSMUSG00000066278	Vps37b
ENSMUSG00000066306	Numa1
ENSMUSG00000066319	Rtp3
ENSMUSG00000066324	Impad1
ENSMUSG00000066357	Wdr6
ENSMUSG00000066406	Akap13
ENSMUSG00000066415	Msl2
ENSMUSG00000066440	Zfyve26
ENSMUSG00000066441	Rdh11
ENSMUSG00000066554	Gm10167
ENSMUSG00000066568	Lsm14a
ENSMUSG00000066571	4931406P16Rik
ENSMUSG00000066632	Pgk1-rs7
ENSMUSG00000066637	Ttc32

ENSMUSG00000066647	Gm5113
ENSMUSG00000066687	Zbtb16
ENSMUSG00000066735	Vkorc1l1
ENSMUSG00000066798	Zbtb6
ENSMUSG00000066839	Ecsit
ENSMUSG00000066892	Fbxl12
ENSMUSG00000067071	Hes6
ENSMUSG00000067150	Xpo5
ENSMUSG00000067199	Frat1
ENSMUSG00000067203	H2-K2
ENSMUSG00000067219	Nipal1
ENSMUSG00000067225	Cyp2c54
ENSMUSG00000067279	Ppp1r3c
ENSMUSG00000067365	Tmem128
ENSMUSG00000067367	Lyar
ENSMUSG00000067424	Zfp563
ENSMUSG00000067722	BC003965
ENSMUSG00000067787	Blcap
ENSMUSG00000067825	Pex26
ENSMUSG00000067835	Gm14661
ENSMUSG00000067869	Tcea1-ps1
ENSMUSG00000067873	Htatsf1
ENSMUSG00000067889	Sptbn2
ENSMUSG00000067924	Rtl8b
ENSMUSG00000067925	Rtl8a
ENSMUSG00000067931	Zfp948
ENSMUSG00000067942	Zfp160
ENSMUSG00000067995	Gtf2f2
ENSMUSG00000068011	Mkrn2os
ENSMUSG00000068015	Lrch1
ENSMUSG00000068036	Afdn
ENSMUSG00000068039	Tcp1
ENSMUSG00000068040	Tm9sf4
ENSMUSG00000068114	Ccdc134
ENSMUSG00000068141	Gm10232
ENSMUSG00000068184	Ndufaf2
ENSMUSG00000068205	Macrod2
ENSMUSG00000068246	Apol9b
ENSMUSG00000068264	Ap5s1
ENSMUSG00000068267	Cenpb
ENSMUSG00000068284	Usf3
ENSMUSG00000068290	Ddrgk1
ENSMUSG00000068328	Aup1
ENSMUSG00000068329	Htra2

ENSMUSG00000068391	Chrac1
ENSMUSG00000068394	Cep152
ENSMUSG00000068463	B630019A10Rik
ENSMUSG00000068551	Zfp467
ENSMUSG00000068566	Myadm
ENSMUSG00000068663	Clec16a
ENSMUSG00000068706	Gm10250
ENSMUSG00000068732	Tmem167b
ENSMUSG00000068739	Sars
ENSMUSG00000068762	Gstm6
ENSMUSG00000068882	Ssb
ENSMUSG00000068917	Clk2
ENSMUSG00000069114	Zbtb10
ENSMUSG00000069206	Zfp874a
ENSMUSG00000069208	Zfp825
ENSMUSG00000069237	Fam8a1
ENSMUSG00000069324	Gm5096
ENSMUSG00000069456	Rdh16
ENSMUSG00000069495	Epc2
ENSMUSG00000069516	Lyz2
ENSMUSG00000069520	Tmem19
ENSMUSG00000069539	Scyl2
ENSMUSG00000069565	Dazap1
ENSMUSG00000069601	Ank3
ENSMUSG00000069633	Pex11g
ENSMUSG00000069662	Marcks
ENSMUSG00000069729	Arid1b
ENSMUSG00000069755	Zfp125
ENSMUSG00000069763	Tmem100
ENSMUSG00000069792	Wfdc17
ENSMUSG00000069793	Slfn9
ENSMUSG00000069808	Fam57a
ENSMUSG00000069833	Ahnak
ENSMUSG00000069844	Sco1
ENSMUSG00000069874	Irgm2
ENSMUSG00000069895	Atxn1l
ENSMUSG00000069913	Anp32-ps
ENSMUSG00000069922	Ces3a
ENSMUSG00000070002	Ell
ENSMUSG00000070044	Fam149a
ENSMUSG00000070047	Fat1
ENSMUSG00000070056	Mfhas1
ENSMUSG00000070284	Gmppb
ENSMUSG00000070319	Eif3g

ENSMUSG00000070327	Rnf213
ENSMUSG00000070343	Gm10288
ENSMUSG00000070354	Evi2
ENSMUSG00000070407	Hs3st3b1
ENSMUSG00000070420	Zscan25
ENSMUSG00000070426	Rnf121
ENSMUSG00000070462	Tlnrd1
ENSMUSG00000070473	Cldn3
ENSMUSG00000070520	Nsmce3
ENSMUSG00000070544	Top1
ENSMUSG00000070594	Gm4788
ENSMUSG00000070661	Rnf186
ENSMUSG00000070690	5830473C10Rik
ENSMUSG00000070697	Utp3
ENSMUSG00000070704	Ugt2b36
ENSMUSG00000070705	Eid2b
ENSMUSG00000070729	Gm12966
ENSMUSG00000070733	Fryl
ENSMUSG00000070780	Rbm47
ENSMUSG00000070808	Bicra
ENSMUSG00000070923	Klhl9
ENSMUSG00000070934	Rraga
ENSMUSG00000070939	Tgfbrap1
ENSMUSG00000070972	Dnajc25
ENSMUSG00000070985	Acnat1
ENSMUSG00000071054	Safb
ENSMUSG00000071074	Yipf3
ENSMUSG00000071076	Jund
ENSMUSG00000071078	Nr2c2ap
ENSMUSG00000071141	Rpl36a-ps3
ENSMUSG00000071178	Serpina1b
ENSMUSG00000071180	Smim15
ENSMUSG00000071204	Gm10319
ENSMUSG00000071226	Cecr2
ENSMUSG00000071256	Zfp213
ENSMUSG00000071267	Zfp942
ENSMUSG00000071335	Mfsd4b3-ps
ENSMUSG00000071350	Setdb2
ENSMUSG00000071359	Tbpl1
ENSMUSG00000071369	Map3k5
ENSMUSG00000071379	Hpcal1
ENSMUSG00000071477	Zfp777
ENSMUSG00000071497	Nutf2-ps1
ENSMUSG00000071533	Pcnp

ENSMUSG00000071551	Akr1c19
ENSMUSG00000071632	2510002D24Rik
ENSMUSG00000071633	Gm4952
ENSMUSG00000071637	Cebpd
ENSMUSG00000071644	Eef1g
ENSMUSG00000071645	Tut1
ENSMUSG00000071649	B3gat3
ENSMUSG00000071650	Ganab
ENSMUSG00000071652	Ints5
ENSMUSG00000071653	1810009A15Rik
ENSMUSG00000071654	Uqcc3
ENSMUSG00000071655	Ubxn1
ENSMUSG00000071659	Hnrnpul2
ENSMUSG00000071660	Ttc9c
ENSMUSG00000071711	Mpst
ENSMUSG00000071757	Zhx2
ENSMUSG00000071856	Mcc
ENSMUSG00000071867	Gm6304
ENSMUSG00000072115	Ang
ENSMUSG00000072294	Klf12
ENSMUSG00000072494	Ppp1r3e
ENSMUSG00000072501	Phf20l1
ENSMUSG00000072568	Fam84b
ENSMUSG00000072582	Ptrh2
ENSMUSG00000072596	Ear2
ENSMUSG00000072620	Slfn2
ENSMUSG00000072625	Gdf2
ENSMUSG00000072664	Ugt3a1
ENSMUSG00000072673	Gm10392
ENSMUSG00000072704	Smim10l1
ENSMUSG00000072770	Acrbp
ENSMUSG00000072809	9330160F10Rik
ENSMUSG00000072825	Cep170b
ENSMUSG00000072849	Serpina1e
ENSMUSG00000072941	Sod3
ENSMUSG00000072949	Acot1
ENSMUSG00000072978	Gm5830
ENSMUSG00000073002	Vamp5
ENSMUSG00000073067	9130019P16Rik
ENSMUSG00000073139	Tmem185a
ENSMUSG00000073144	4930599N23Rik
ENSMUSG00000073147	5031425E22Rik
ENSMUSG00000073155	1810058I24Rik
ENSMUSG00000073409	H2-Q6

ENSMUSG00000073411	H2-D1
ENSMUSG00000073436	Eme2
ENSMUSG00000073490	Ifi207
ENSMUSG00000073555	Gm4951
ENSMUSG00000073563	Csnk1g3
ENSMUSG00000073565	Prr16
ENSMUSG00000073600	Prob1
ENSMUSG00000073616	Cops9
ENSMUSG00000073639	Rab18
ENSMUSG00000073647	Gm10557
ENSMUSG00000073682	Gm10563
ENSMUSG00000073684	Faap20
ENSMUSG00000073700	Klhl21
ENSMUSG00000073725	Lmbrd1
ENSMUSG00000073731	AI507597
ENSMUSG00000073775	Kti12
ENSMUSG00000073778	Faddos
ENSMUSG00000073830	Mup14
ENSMUSG00000073834	Mup11
ENSMUSG00000073842	Mup7
ENSMUSG00000073910	Mob3b
ENSMUSG00000073940	Hbb-bt
ENSMUSG00000073982	Rhog
ENSMUSG00000073988	Ttpa
ENSMUSG00000074030	Exoc8
ENSMUSG00000074052	BC048644
ENSMUSG00000074063	Osgin1
ENSMUSG00000074064	Mlycd
ENSMUSG00000074102	Rbm15b
ENSMUSG00000074129	Rpl13a
ENSMUSG00000074141	Il4i1
ENSMUSG00000074151	Nlrc5
ENSMUSG00000074166	AW146154
ENSMUSG00000074170	Plekhf1
ENSMUSG00000074182	Znhit6
ENSMUSG00000074194	Zfp791
ENSMUSG00000074207	Adh1
ENSMUSG00000074211	Sdhaf1
ENSMUSG00000074213	Gm10642
ENSMUSG00000074221	Zfp568
ENSMUSG00000074247	Dda1
ENSMUSG00000074264	Amy1
ENSMUSG00000074272	Ceacam1
ENSMUSG00000074284	Gm10658

ENSMUSG00000074305	Peak1
ENSMUSG00000074336	Apoc4
ENSMUSG00000074373	Gm10680
ENSMUSG00000074398	Gm15441
ENSMUSG00000074405	Zfp865
ENSMUSG00000074406	Zfp628
ENSMUSG00000074457	S100a16
ENSMUSG00000074465	Gm10701
ENSMUSG00000074491	Clec4g
ENSMUSG00000074506	Gm10705
ENSMUSG00000074529	Zfp972
ENSMUSG00000074576	Mocs3
ENSMUSG00000074582	Arfgef2
ENSMUSG00000074622	Mafb
ENSMUSG00000074639	Rdh16f2
ENSMUSG00000074643	Cpne1
ENSMUSG00000074646	6430550D23Rik
ENSMUSG00000074649	BC029722
ENSMUSG00000074656	Eif2s2
ENSMUSG00000074743	Thbd
ENSMUSG00000074746	Pdzd8
ENSMUSG00000074748	Atxn7l3b
ENSMUSG00000074749	Kiz
ENSMUSG00000074754	Smim26
ENSMUSG00000074780	Anapc15-ps
ENSMUSG00000074794	Arrdc3
ENSMUSG00000074807	Gm10762
ENSMUSG00000074811	Hps6
ENSMUSG00000074829	2010315B03Rik
ENSMUSG00000074846	Gm10774
ENSMUSG00000074874	Ctla2b
ENSMUSG00000074890	Lcmt2
ENSMUSG00000074896	Ifit3
ENSMUSG00000074909	Ranbp6
ENSMUSG00000074918	Inafm2
ENSMUSG00000074922	Fam122a
ENSMUSG00000074994	Qser1
ENSMUSG00000075000	Nrbf2
ENSMUSG00000075010	AW112010
ENSMUSG00000075014	Gm10800
ENSMUSG00000075040	Zfp408
ENSMUSG00000075054	Yae1d1
ENSMUSG00000075227	Znhit2
ENSMUSG00000075232	Amd1

ENSMUSG00000075254	Heg1
ENSMUSG00000075273	Ttc30b
ENSMUSG00000075327	Zbtb2
ENSMUSG00000075419	Dolk
ENSMUSG00000075467	Dnlz
ENSMUSG00000075486	Commd6
ENSMUSG00000075517	Cyp2d37-ps
ENSMUSG00000075543	Urad
ENSMUSG00000075576	Gm12359
ENSMUSG00000075590	Nrbp2
ENSMUSG00000075595	Zfp652
ENSMUSG00000075700	Selenot
ENSMUSG00000075701	Selenos
ENSMUSG00000075703	Selenoi
ENSMUSG00000075705	Msrb1
ENSMUSG00000076138	Gm26377
ENSMUSG00000076435	Acsf2
ENSMUSG00000076441	Ass1
ENSMUSG00000076609	Igkc
ENSMUSG00000077450	Rab11b
ENSMUSG00000078139	AK157302
ENSMUSG00000078143	Gm17344
ENSMUSG00000078173	Lenep
ENSMUSG00000078184	Rbm8a2
ENSMUSG00000078234	Klhdc7a
ENSMUSG00000078249	Hmga1b
ENSMUSG00000078317	F8a
ENSMUSG00000078348	Sf3b5
ENSMUSG00000078350	Smim1
ENSMUSG00000078429	Ctdsp2
ENSMUSG00000078489	Gm17106
ENSMUSG00000078515	Ddi2
ENSMUSG00000078570	1110065P20Rik
ENSMUSG00000078580	E430018J23Rik
ENSMUSG00000078584	AU022252
ENSMUSG00000078588	Ccdc24
ENSMUSG00000078622	Ccdc47
ENSMUSG00000078650	G6pc
ENSMUSG00000078652	Psme3
ENSMUSG00000078670	Fam174b
ENSMUSG00000078671	Chd2
ENSMUSG00000078674	Mup18
ENSMUSG00000078675	Mup16
ENSMUSG00000078676	Casc3

ENSMUSG00000078684	5830417I10Rik
ENSMUSG00000078695	Cisd3
ENSMUSG00000078713	Tomm5
ENSMUSG00000078771	Evi2a
ENSMUSG00000078786	BC024978
ENSMUSG00000078789	Dph1
ENSMUSG00000078812	Eif5a
ENSMUSG00000078817	Nlrp12
ENSMUSG00000078851	Hist3h2a
ENSMUSG00000078853	Igtp
ENSMUSG00000078920	Ifi47
ENSMUSG00000078923	Ube2v1
ENSMUSG00000078931	Pdf
ENSMUSG00000078941	Ak6
ENSMUSG00000078964	Ces1b
ENSMUSG00000079003	Samd1
ENSMUSG00000079012	Serpina3m
ENSMUSG00000079020	Slc45a4
ENSMUSG00000079036	Alkbh1
ENSMUSG00000079037	Prnp
ENSMUSG00000079043	Fastkd5
ENSMUSG00000079045	Prox1os
ENSMUSG00000079057	Cyp4v3
ENSMUSG00000079076	Gm3086
ENSMUSG00000079083	Jrkl
ENSMUSG00000079104	Prps1l3
ENSMUSG00000079111	Kdelr2
ENSMUSG00000079140	Gm6374
ENSMUSG00000079144	A130010J15Rik
ENSMUSG00000079184	Mphosph8
ENSMUSG00000079215	Zfp664
ENSMUSG00000079225	Gm9531
ENSMUSG00000079227	Ccr5
ENSMUSG00000079293	Clec7a
ENSMUSG00000079316	Rab9
ENSMUSG00000079334	Naa80
ENSMUSG00000079362	Gm43302
ENSMUSG00000079418	Atg4a
ENSMUSG00000079426	Arpc4
ENSMUSG00000079469	Pigb
ENSMUSG00000079477	Rab7
ENSMUSG00000079478	Ssscа1
ENSMUSG00000079484	Phyhd1
ENSMUSG00000079487	Med12

ENSMUSG00000079505	Gm11131
ENSMUSG00000079509	Zfx
ENSMUSG00000079511	Gm42688
ENSMUSG00000079557	2-Mar
ENSMUSG00000079562	Maea
ENSMUSG00000079563	Pglyrp2
ENSMUSG00000079605	Zbtb9
ENSMUSG00000079677	Fdx1l
ENSMUSG00000079941	Gm11273
ENSMUSG00000080058	Gm11175
ENSMUSG00000080268	Brms1
ENSMUSG00000080772	Gm12543
ENSMUSG00000080775	Gm6368
ENSMUSG00000080811	Gm14513
ENSMUSG00000080893	Gm15920
ENSMUSG00000080896	Gm14567
ENSMUSG00000080902	Ywhaq-ps3
ENSMUSG00000081049	Rps24-ps3
ENSMUSG00000081094	Rpl19-ps11
ENSMUSG00000081113	Gm7308
ENSMUSG00000081227	Gm13082
ENSMUSG00000081228	Gm16089
ENSMUSG00000081302	Gm12020
ENSMUSG00000081413	Uqcrh-ps2
ENSMUSG00000081583	Gm14769
ENSMUSG00000081603	Gm14681
ENSMUSG00000081700	Atp5k-ps2
ENSMUSG00000081729	Hspa9-ps1
ENSMUSG00000081731	Calr-ps
ENSMUSG00000081788	Gm5898
ENSMUSG00000081792	Anp32b-ps1
ENSMUSG00000081824	BC002163
ENSMUSG00000081871	Gm11488
ENSMUSG00000081888	Spcs2-ps
ENSMUSG00000081988	Gm15746
ENSMUSG00000082002	Gm6568
ENSMUSG00000082120	Gm15720
ENSMUSG00000082145	Gm12312
ENSMUSG00000082189	Gm4784
ENSMUSG00000082194	Gm12444
ENSMUSG00000082272	Gm11675
ENSMUSG00000082319	Gm8822
ENSMUSG00000082454	Gm12183
ENSMUSG00000082491	Gm5909

ENSMUSG00000082530	Gm12168
ENSMUSG00000082534	Gm13712
ENSMUSG00000082674	Gm11914
ENSMUSG00000082691	Dynlt1-ps1
ENSMUSG00000082848	Gm16066
ENSMUSG00000082872	Gm15773
ENSMUSG00000082876	Gm11889
ENSMUSG00000082878	Gm12583
ENSMUSG00000082925	Gm13135
ENSMUSG00000082938	Gm2810
ENSMUSG00000082998	Gm11221
ENSMUSG00000083012	Fam220a
ENSMUSG00000083093	Gm15385
ENSMUSG00000083179	Gm12693
ENSMUSG00000083240	Gm13453
ENSMUSG00000083270	Gm13498
ENSMUSG00000083364	Llph-ps2
ENSMUSG00000083367	Gm8806
ENSMUSG00000083396	Gm15542
ENSMUSG00000083692	Gm9575
ENSMUSG00000083700	Gm2986
ENSMUSG00000083736	Gm6039
ENSMUSG00000083798	Gm14584
ENSMUSG00000083820	Ndufs6b
ENSMUSG00000083844	Ube2d-ps
ENSMUSG00000083992	Gm11478
ENSMUSG00000084067	Gm14269
ENSMUSG00000084094	Gm13961
ENSMUSG00000084127	Gm13169
ENSMUSG00000084128	Esrp2
ENSMUSG00000084159	Gm12696
ENSMUSG00000084260	Gm12844
ENSMUSG00000084300	Gm6954
ENSMUSG00000084304	Gm6142
ENSMUSG00000084761	Gm12406
ENSMUSG00000084808	9430091E24Rik
ENSMUSG00000084835	Gm12352
ENSMUSG00000084839	Gm14097
ENSMUSG00000084883	Ccdc85c
ENSMUSG00000084910	C630043F03Rik
ENSMUSG00000084981	Gm15962
ENSMUSG00000084983	Gm11789
ENSMUSG00000084997	Gm15471
ENSMUSG00000085012	Gm15492

ENSMUSG00000085028	Slc2a4rg-ps
ENSMUSG00000085148	Mir22hg
ENSMUSG00000085172	Gm6542
ENSMUSG00000085185	BC028777
ENSMUSG00000085208	Brip1os
ENSMUSG00000085236	2610206C17Rik
ENSMUSG00000085289	Gm15337
ENSMUSG00000085337	Gm15964
ENSMUSG00000085376	Gm14508
ENSMUSG00000085438	1700020I14Rik
ENSMUSG00000085465	Gm15347
ENSMUSG00000085505	Gm12868
ENSMUSG00000085587	Gm14493
ENSMUSG00000085595	Gm16090
ENSMUSG00000085642	3110053B16Rik
ENSMUSG00000085664	Atxn7l1os2
ENSMUSG00000085667	Gm12992
ENSMUSG00000085711	Gm15163
ENSMUSG00000085714	Gm13008
ENSMUSG00000085720	Gm7854
ENSMUSG00000085788	4930470G03Rik
ENSMUSG00000085793	Lin52
ENSMUSG00000085795	Zfp703
ENSMUSG00000085818	Gm13267
ENSMUSG00000085826	Gm15638
ENSMUSG00000085875	Gm12905
ENSMUSG00000085882	2610507I01Rik
ENSMUSG00000085885	Gm11906
ENSMUSG00000085890	Tnfsf13os
ENSMUSG00000085913	Gm15601
ENSMUSG00000085962	Gm16984
ENSMUSG00000085971	Gm15411
ENSMUSG00000086043	Gm12473
ENSMUSG00000086047	9530046B11Rik
ENSMUSG00000086090	Gm12531
ENSMUSG00000086141	9030622O22Rik
ENSMUSG00000086184	Gm12764
ENSMUSG00000086219	Srrm4os
ENSMUSG00000086245	Gm16170
ENSMUSG00000086269	Tmcc3os
ENSMUSG00000086296	D030055H07Rik
ENSMUSG00000086330	1700007J10Rik
ENSMUSG00000086332	4930480G23Rik
ENSMUSG00000086341	Gm15932

ENSMUSG00000086390	1810019D21Rik
ENSMUSG00000086392	Mccc1os
ENSMUSG00000086401	Gm15559
ENSMUSG00000086407	Gm14123
ENSMUSG00000086414	Gm15645
ENSMUSG00000086459	1700030C12Rik
ENSMUSG00000086514	Gm11747
ENSMUSG00000086582	Gm16272
ENSMUSG00000086583	Gm15500
ENSMUSG00000086593	Gm16548
ENSMUSG00000086594	Nudt12os
ENSMUSG00000086610	Gm15408
ENSMUSG00000086682	Gm16023
ENSMUSG00000086693	A730081D07Rik
ENSMUSG00000086697	4933427G23Rik
ENSMUSG00000086714	0610009E02Rik
ENSMUSG00000086717	Gm15655
ENSMUSG00000086724	Gm12833
ENSMUSG00000086725	A630052C17Rik
ENSMUSG00000086728	Man2c1os
ENSMUSG00000086740	Gm17029
ENSMUSG00000086765	Gm11827
ENSMUSG00000086779	Gm13562
ENSMUSG00000086782	E130102H24Rik
ENSMUSG00000086784	Isoc2a
ENSMUSG00000086825	Gm15675
ENSMUSG00000086839	Gm11973
ENSMUSG00000086914	Gm16124
ENSMUSG00000086922	Gm13835
ENSMUSG00000086929	Gm11788
ENSMUSG00000086942	Gm15489
ENSMUSG00000086949	Gm13066
ENSMUSG00000087006	Gm13889
ENSMUSG00000087097	4930586N03Rik
ENSMUSG00000087109	4930474H06Rik
ENSMUSG00000087141	Plcxd2
ENSMUSG00000087240	Gm15976
ENSMUSG00000087327	Gm15884
ENSMUSG00000087331	1810021B22Rik
ENSMUSG00000087338	Scpep1os
ENSMUSG00000087339	Gm11586
ENSMUSG00000087341	0610040F04Rik
ENSMUSG00000087352	Gm12999
ENSMUSG00000087356	Gm13856

ENSMUSG00000087367	Gm15491
ENSMUSG00000087370	Tmem170b
ENSMUSG00000087382	Ctcflos
ENSMUSG00000087385	Frg2f1
ENSMUSG00000087400	Gm15270
ENSMUSG00000087412	Gm15501
ENSMUSG00000087436	Gm16156
ENSMUSG00000087439	Gm15788
ENSMUSG00000087515	Gm13554
ENSMUSG00000087516	Tbx3os1
ENSMUSG00000087526	Gm15738
ENSMUSG00000087528	9830144P21Rik
ENSMUSG00000087541	Hopxos
ENSMUSG00000087590	Epb41l4aos
ENSMUSG00000087610	Gm16253
ENSMUSG00000087612	A230005M16Rik
ENSMUSG00000087640	Gm14798
ENSMUSG00000087679	Tmem250-ps
ENSMUSG00000087687	Pet100
ENSMUSG00000087691	Gm15674
ENSMUSG00000087700	Gm15283
ENSMUSG00000089628	Gm9727
ENSMUSG00000089635	Gm16559
ENSMUSG00000089671	Gm16537
ENSMUSG00000089675	Ugt1a8
ENSMUSG00000089678	Agxt2
ENSMUSG00000089704	Galnt2
ENSMUSG00000089739	Gm20431
ENSMUSG00000089762	Ier5l
ENSMUSG00000089766	Gm16538
ENSMUSG00000089824	Rbm12
ENSMUSG00000089838	Gm2962
ENSMUSG00000089865	Gm44503
ENSMUSG00000089872	Rps6kc1
ENSMUSG00000089875	Etohd2
ENSMUSG00000089876	Tmem102
ENSMUSG00000089935	Gm16343
ENSMUSG00000089943	Ugt1a5
ENSMUSG00000089945	Pakap
ENSMUSG00000089956	Gm16553
ENSMUSG00000089960	Ugt1a1
ENSMUSG00000089961	Gm16567
ENSMUSG00000089983	2010320O07Rik
ENSMUSG00000089984	Fbxo24

ENSMUSG00000089989	Gm45713
ENSMUSG00000090019	Gimap1
ENSMUSG00000090035	Galnt4
ENSMUSG00000090038	Gm16573
ENSMUSG00000090083	Rnf8
ENSMUSG00000090117	Gm16541
ENSMUSG00000090118	Gm16163
ENSMUSG00000090119	Gm8185
ENSMUSG00000090124	Ugt1a7c
ENSMUSG00000090136	Gm10177
ENSMUSG00000090145	Ugt1a6b
ENSMUSG00000090157	Gm16534
ENSMUSG00000090165	Ugt1a10
ENSMUSG00000090171	Ugt1a2
ENSMUSG00000090175	Ugt1a9
ENSMUSG00000090207	4930524O07Rik
ENSMUSG00000090208	Gm15851
ENSMUSG00000090213	Tmem189
ENSMUSG00000090247	Bloc1s1
ENSMUSG00000090272	Mndal
ENSMUSG00000090306	Adh6-ps1
ENSMUSG00000090357	Gm17077
ENSMUSG00000090389	Cdv3-ps
ENSMUSG00000090394	4930523C07Rik
ENSMUSG00000090406	Gm17058
ENSMUSG00000090523	Gypc
ENSMUSG00000090570	Gm17041
ENSMUSG00000090608	Gm17200
ENSMUSG00000090622	A930033H14Rik
ENSMUSG00000090623	Cfhr3
ENSMUSG00000090625	Gm20721
ENSMUSG00000090639	Gm20425
ENSMUSG00000090861	Arf4os
ENSMUSG00000090863	A530084C06Rik
ENSMUSG00000090877	Hspa1b
ENSMUSG00000090935	Synj2bp
ENSMUSG00000090942	F830016B08Rik
ENSMUSG00000090946	Ccdc71l
ENSMUSG00000090952	Gm17251
ENSMUSG00000090958	Lrrc32
ENSMUSG00000090996	Gm20458
ENSMUSG00000091041	Gm17720
ENSMUSG00000091050	9330020H09Rik
ENSMUSG00000091058	Gm17538

ENSMUSG00000091144	Phf11c
ENSMUSG00000091154	Proscos
ENSMUSG00000091228	Gm20390
ENSMUSG00000091237	Gm17114
ENSMUSG00000091264	Smim13
ENSMUSG00000091306	Gm17606
ENSMUSG00000091329	1700112D23Rik
ENSMUSG00000091337	Eid1
ENSMUSG00000091387	Gcnt4
ENSMUSG00000091390	Gm17168
ENSMUSG00000091514	Gm17484
ENSMUSG00000091586	Cyp4f17
ENSMUSG00000091623	Gm17092
ENSMUSG00000091780	Sco2
ENSMUSG00000091811	Inafm1
ENSMUSG00000091825	Gm5778
ENSMUSG00000091845	Rpl36-ps12
ENSMUSG00000091896	Ube2d2a
ENSMUSG00000091931	Gon7
ENSMUSG00000091985	Gm17354
ENSMUSG00000091993	B930036N10Rik
ENSMUSG00000092006	Gm17139
ENSMUSG00000092008	Cyp2c69
ENSMUSG00000092021	Gbp11
ENSMUSG00000092075	Serpina4-ps1
ENSMUSG00000092118	Fancf
ENSMUSG00000092187	Gm20457
ENSMUSG00000092220	Gm20528
ENSMUSG00000092232	Gm20521
ENSMUSG00000092241	Gm20522
ENSMUSG00000092274	Neat1
ENSMUSG00000092283	Gm20412
ENSMUSG00000092329	Gm20388
ENSMUSG00000092341	Malat1
ENSMUSG00000092345	Gm20503
ENSMUSG00000092356	Gm20532
ENSMUSG00000092360	Gm20441
ENSMUSG00000092364	Gm20476
ENSMUSG00000092368	A930015D03Rik
ENSMUSG00000092386	Gm20536
ENSMUSG00000092417	Gpank1
ENSMUSG00000092470	Gm20518
ENSMUSG00000092534	Pagr1b
ENSMUSG00000092541	Gm20537

ENSMUSG00000092544	Gm20422
ENSMUSG00000092545	Gm20319
ENSMUSG00000092558	Med20
ENSMUSG00000092592	Gm20449
ENSMUSG00000092595	Gm20427
ENSMUSG00000092655	Gm25238
ENSMUSG00000092686	Gm25745
ENSMUSG00000093445	Lrch4
ENSMUSG00000093452	Zfhx2os
ENSMUSG00000093485	Gm20708
ENSMUSG00000093545	Gm5871
ENSMUSG00000093575	Gm20695
ENSMUSG00000093662	Gm20649
ENSMUSG00000093674	Rpl41
ENSMUSG00000093701	Gm21970
ENSMUSG00000093752	Gm20716
ENSMUSG00000093753	Gm28577
ENSMUSG00000093772	4931403E22Rik
ENSMUSG00000093916	Gm379
ENSMUSG00000093930	Hmgcs1
ENSMUSG00000093938	Evi2b
ENSMUSG00000093989	Rnasek
ENSMUSG00000094090	Gm8494
ENSMUSG00000094365	Gm21982
ENSMUSG00000094410	Gm38394
ENSMUSG00000094437	Gm9830
ENSMUSG00000094439	Gm21969
ENSMUSG00000094441	Zfp955a
ENSMUSG00000094483	Purb
ENSMUSG00000094638	Gm21972
ENSMUSG00000094724	Rnaset2b
ENSMUSG00000094786	Gm14403
ENSMUSG00000094793	Mup12
ENSMUSG00000094806	Cyp2d10
ENSMUSG00000094814	Gm21973
ENSMUSG00000094870	Zfp131
ENSMUSG00000095098	Ccdc85b
ENSMUSG00000095325	Zfp870
ENSMUSG00000095440	Fignl2
ENSMUSG00000095464	Gm21987
ENSMUSG00000095567	Noc2l
ENSMUSG00000095687	Rnaset2a
ENSMUSG00000095913	Gm21844
ENSMUSG00000096002	Vmn2r53

ENSMUSG00000096054	Syne1
ENSMUSG00000096056	Gm21986
ENSMUSG00000096199	Ptrhd1
ENSMUSG00000096210	H1f0
ENSMUSG00000096351	Samd11
ENSMUSG00000096370	Gm21992
ENSMUSG00000096458	Moap1
ENSMUSG00000096544	Gm4617
ENSMUSG00000096606	Tpbgl
ENSMUSG00000096687	Mfsd4b4
ENSMUSG00000096740	Lbhd1
ENSMUSG00000096764	Gm21985
ENSMUSG00000096842	Gm10736
ENSMUSG00000096910	Zfp955b
ENSMUSG00000096942	Rps19-ps6
ENSMUSG00000096958	Gm26573
ENSMUSG00000096971	4930556M19Rik
ENSMUSG00000096972	Gm26883
ENSMUSG00000096981	Gm16845
ENSMUSG00000096990	Gm26790
ENSMUSG00000097000	Gm17435
ENSMUSG00000097006	9530082P21Rik
ENSMUSG00000097008	Gm26860
ENSMUSG00000097042	Gm17491
ENSMUSG00000097051	Gm26836
ENSMUSG00000097062	Gm17586
ENSMUSG00000097099	Gm9917
ENSMUSG00000097101	1810034E14Rik
ENSMUSG00000097121	D130020L05Rik
ENSMUSG00000097122	Gm26624
ENSMUSG00000097125	Gm26885
ENSMUSG00000097142	Gm26778
ENSMUSG00000097148	Gm3839
ENSMUSG00000097167	Gm16740
ENSMUSG00000097168	C230088H06Rik
ENSMUSG00000097197	9530027J09Rik
ENSMUSG00000097213	Gm26745
ENSMUSG00000097219	Gm26551
ENSMUSG00000097221	1810049J17Rik
ENSMUSG00000097234	Gm26518
ENSMUSG00000097245	Gm5421
ENSMUSG00000097253	Gm26770
ENSMUSG00000097275	Gm26648
ENSMUSG00000097286	Gm26684

ENSMUSG00000097321	1700028E10Rik
ENSMUSG00000097328	Tnfsf12
ENSMUSG00000097331	F420014N23Rik
ENSMUSG00000097353	A430046D13Rik
ENSMUSG00000097380	Gm26816
ENSMUSG00000097381	A230087F16Rik
ENSMUSG00000097397	Gm16861
ENSMUSG00000097410	Gm26668
ENSMUSG00000097417	Gm26669
ENSMUSG00000097426	Gm8941
ENSMUSG00000097433	Gm26781
ENSMUSG00000097439	Gm16754
ENSMUSG00000097457	2310031A07Rik
ENSMUSG00000097469	Gm26733
ENSMUSG00000097479	Gm26582
ENSMUSG00000097517	Gm26695
ENSMUSG00000097518	Gm26694
ENSMUSG00000097533	Gm26590
ENSMUSG00000097537	2610020C07Rik
ENSMUSG00000097554	Gm26825
ENSMUSG00000097560	Gm26904
ENSMUSG00000097564	C430014B12Rik
ENSMUSG00000097577	6230400D17Rik
ENSMUSG00000097590	Gm26675
ENSMUSG00000097605	9430098F02Rik
ENSMUSG00000097614	Gm26508
ENSMUSG00000097625	Gm26561
ENSMUSG00000097640	Gm20033
ENSMUSG00000097644	Gm26862
ENSMUSG00000097654	Gm26714
ENSMUSG00000097658	Gm16755
ENSMUSG00000097690	4930505N22Rik
ENSMUSG00000097691	9030616G12Rik
ENSMUSG00000097724	Gm26850
ENSMUSG00000097748	Gm26533
ENSMUSG00000097750	Gm4673
ENSMUSG00000097762	4732463B04Rik
ENSMUSG00000097799	Gm26899
ENSMUSG00000097811	2810425M01Rik
ENSMUSG00000097815	Gm26809
ENSMUSG00000097852	4933405D12Rik
ENSMUSG00000097867	Lppos
ENSMUSG00000097887	Gm26542
ENSMUSG00000097893	1700034P13Rik

ENSMUSG00000097913	Gm26837
ENSMUSG00000097919	Gm27021
ENSMUSG00000097930	C330002G04Rik
ENSMUSG00000098066	Gm26944
ENSMUSG00000098134	Rnf113a2
ENSMUSG00000098140	Gm26938
ENSMUSG00000098188	Sowahc
ENSMUSG00000098282	Mir6936
ENSMUSG00000098284	A330093E20Rik
ENSMUSG00000098302	Gm28039
ENSMUSG00000098306	Gm28040
ENSMUSG00000098330	Gtpbp4-ps1
ENSMUSG00000098371	Gm28037
ENSMUSG00000098374	Gm28043
ENSMUSG00000098387	Pet117
ENSMUSG00000098503	Gm27937
ENSMUSG00000098530	Gm28051
ENSMUSG00000098557	Kctd12
ENSMUSG00000098573	Gm27232
ENSMUSG00000098650	Gm28048
ENSMUSG00000098708	Gm27252
ENSMUSG00000098739	Gm27151
ENSMUSG00000098754	Prn
ENSMUSG00000098794	Gm28038
ENSMUSG00000098912	1500004A13Rik
ENSMUSG00000098923	Tmem185b
ENSMUSG00000098950	Gm28036
ENSMUSG00000099009	Rdh16f1
ENSMUSG00000099041	Gm28035
ENSMUSG00000099083	Atf7
ENSMUSG00000099146	0610031O16Rik
ENSMUSG00000099227	Mir8114
ENSMUSG00000099329	Gm28052
ENSMUSG00000099377	Gm6159
ENSMUSG00000099397	Gm7809
ENSMUSG00000099492	Gm5525
ENSMUSG00000099519	Gm29253
ENSMUSG00000099568	Gm28513
ENSMUSG00000099681	1700052K11Rik
ENSMUSG00000099706	Gm29536
ENSMUSG00000099707	Gm8883
ENSMUSG00000099803	Gm28863
ENSMUSG00000099843	Gm7160
ENSMUSG00000099851	Gm29542

ENSMUSG00000099858	Gm6652
ENSMUSG00000099881	2810013P06Rik
ENSMUSG00000099902	Gm12115
ENSMUSG00000099908	Gm28539
ENSMUSG00000099913	Gm28551
ENSMUSG00000099966	2810402E24Rik
ENSMUSG00000100017	2410022M11Rik
ENSMUSG00000100037	Gm29103
ENSMUSG00000100094	1810008I18Rik
ENSMUSG00000100113	Gm28592
ENSMUSG00000100162	Gm20687
ENSMUSG00000100235	Gm28557
ENSMUSG00000100548	Gm29585
ENSMUSG00000100594	2810414N06Rik
ENSMUSG00000100691	2010320M18Rik
ENSMUSG00000100801	Gm15459
ENSMUSG00000100927	Gm28536
ENSMUSG00000100954	Gm10138
ENSMUSG00000100969	1700030N03Rik
ENSMUSG00000101013	A630072M18Rik
ENSMUSG00000101014	Gm28289
ENSMUSG00000101033	Gm18776
ENSMUSG00000101133	Gm29050
ENSMUSG00000101152	Gm28305
ENSMUSG00000101166	Gm28496
ENSMUSG00000101304	Plet1os
ENSMUSG00000101397	Mug-ps1
ENSMUSG00000101514	Gm5524
ENSMUSG00000101585	1600010M07Rik
ENSMUSG00000101586	Gm29017
ENSMUSG00000101589	Rbm6-ps1
ENSMUSG00000101628	Gm28177
ENSMUSG00000101645	Gm28635
ENSMUSG00000101678	Gm29609
ENSMUSG00000101799	9330175M20Rik
ENSMUSG00000101845	Gm28198
ENSMUSG00000101856	1700096K18Rik
ENSMUSG00000101904	Gm29427
ENSMUSG00000101970	1810026B05Rik
ENSMUSG00000102014	2900009J06Rik
ENSMUSG00000102049	Zbed6
ENSMUSG00000102060	1700061E17Rik
ENSMUSG00000102095	C730036E19Rik
ENSMUSG00000102101	Zbtb11os1

ENSMUSG00000102349	Gm37376
ENSMUSG00000102353	Gm38345
ENSMUSG00000102422	Iqschfp
ENSMUSG00000102558	Gm37571
ENSMUSG00000102564	Gm37035
ENSMUSG00000102644	Thap6
ENSMUSG00000102725	Gm37939
ENSMUSG00000102805	Gm37240
ENSMUSG00000102824	Pdcd5-ps
ENSMUSG00000102869	2900097C17Rik
ENSMUSG00000102976	Zc3h11a
ENSMUSG00000103041	Gm37305
ENSMUSG00000103392	Gm38303
ENSMUSG00000103393	Gm38304
ENSMUSG00000103421	Goltla
ENSMUSG00000103502	9330121J05Rik
ENSMUSG00000103548	Gm37670
ENSMUSG00000103649	Gm37768
ENSMUSG00000103651	Gm37206
ENSMUSG00000103676	Gm38204
ENSMUSG00000103922	Gm6123
ENSMUSG00000104245	Gm38155
ENSMUSG00000104443	4932442E05Rik
ENSMUSG00000104444	Gm33051
ENSMUSG00000104448	Gm37256
ENSMUSG00000104491	Gm32340
ENSMUSG00000104523	Gm37335
ENSMUSG00000104664	Gm35570
ENSMUSG00000104713	Gbp6
ENSMUSG00000104761	Gm43511
ENSMUSG00000104882	Gm43096
ENSMUSG00000105053	Gm43064
ENSMUSG00000105055	Gm43079
ENSMUSG00000105096	Gbp10
ENSMUSG00000105103	Gm43191
ENSMUSG00000105338	Gm43802
ENSMUSG00000105339	Gm42457
ENSMUSG00000105391	Gm43401
ENSMUSG00000105646	Gm30211
ENSMUSG00000105703	Gm43305
ENSMUSG00000105704	Gm43055
ENSMUSG00000105787	Gm8099
ENSMUSG00000105813	Gm42928
ENSMUSG00000105835	Gm43552

ENSMUSG00000105841	Mir3960
ENSMUSG00000105852	Gm42890
ENSMUSG00000106190	Gm20768
ENSMUSG00000106294	Gm8539
ENSMUSG00000106352	5033403H07Rik
ENSMUSG00000106418	Gm44397
ENSMUSG00000106447	Gm42957
ENSMUSG00000106526	Gm42604
ENSMUSG00000106538	Gm30301
ENSMUSG00000106797	Gm42936
ENSMUSG00000106831	Ube2n-ps1
ENSMUSG00000106847	Peg13
ENSMUSG00000106863	Gm42109
ENSMUSG00000106864	Gtf3c2
ENSMUSG00000106889	Gm7463
ENSMUSG00000107002	0610012G03Rik
ENSMUSG00000107023	Gm42715
ENSMUSG00000107068	Gm42742
ENSMUSG00000107071	Gm42420
ENSMUSG00000107086	Gm43808
ENSMUSG00000107176	Gm9794
ENSMUSG00000107191	Gm43579
ENSMUSG00000107283	Mpv17
ENSMUSG00000107478	Gm45234
ENSMUSG00000107482	Etfbl
ENSMUSG00000107539	Gm44790
ENSMUSG00000107588	Gm45021
ENSMUSG00000107596	Gm18913
ENSMUSG00000107747	Gm5881
ENSMUSG00000107789	Gm44965
ENSMUSG00000107799	Gm38825
ENSMUSG00000107802	1700126G02Rik
ENSMUSG00000107838	Gm45769
ENSMUSG00000107881	Gm44250
ENSMUSG00000107928	Gm45140
ENSMUSG00000107999	Gm44123
ENSMUSG00000108034	4930557K07Rik
ENSMUSG00000108112	Gm45193
ENSMUSG00000108126	Gm43909
ENSMUSG00000108282	Gm44317
ENSMUSG00000108332	D530033B14Rik
ENSMUSG00000108518	Gm5587
ENSMUSG00000108546	Gm44662
ENSMUSG00000108563	Gm44686

ENSMUSG00000108621	Gm37494
ENSMUSG00000108702	Gm9333
ENSMUSG00000108793	Gm45213
ENSMUSG00000108806	Gm44729
ENSMUSG00000108815	Gm49388
ENSMUSG00000108822	Gm44787
ENSMUSG00000108874	Gm9711
ENSMUSG00000108955	Gm44775
ENSMUSG00000109061	Gm49320
ENSMUSG00000109093	Gm19950
ENSMUSG00000109129	Gm44973
ENSMUSG00000109168	Gm44709
ENSMUSG00000109222	Gm10297
ENSMUSG00000109274	Gm45133
ENSMUSG00000109291	Gm2814
ENSMUSG00000109350	Gm44805
ENSMUSG00000109428	Gm45698
ENSMUSG00000109448	Gm45224
ENSMUSG00000109455	Gm44710
ENSMUSG00000109482	Gm4756
ENSMUSG00000109484	Gm44870
ENSMUSG00000109493	Gm45208
ENSMUSG00000109511	Nup62
ENSMUSG00000109628	BC024386
ENSMUSG00000109644	0610005C13Rik
ENSMUSG00000109764	Klkb1
ENSMUSG00000109799	Gm45515
ENSMUSG00000109894	Gm5904
ENSMUSG00000109901	Chmp1b
ENSMUSG00000109926	Gm45808
ENSMUSG00000109941	Exosc6
ENSMUSG00000109954	Gm45371
ENSMUSG00000109984	Gm38948
ENSMUSG00000110040	Gm49369
ENSMUSG00000110104	Gm45717
ENSMUSG00000110136	Gm45785
ENSMUSG00000110195	Pde2a
ENSMUSG00000110206	Flt3l
ENSMUSG00000110277	Gm45871
ENSMUSG00000110353	Gm33543
ENSMUSG00000110386	Gm42031
ENSMUSG00000110440	Gm45894
ENSMUSG00000110505	Gm45842
ENSMUSG00000110537	Gm4316

ENSMUSG00000110567	Gm45703
ENSMUSG00000110588	Gm45774
ENSMUSG00000110615	Gm45890
ENSMUSG00000110617	Gm5910
ENSMUSG00000110631	Gm42047
ENSMUSG00000110704	Gm45732
ENSMUSG00000110715	Gm5131
ENSMUSG00000110750	Gm48702
ENSMUSG00000110755	BC049987
ENSMUSG00000110803	Gm20275
ENSMUSG00000111128	Gm49338
ENSMUSG00000111133	Gm5831
ENSMUSG00000111312	Gm47205
ENSMUSG00000111368	Gm47889
ENSMUSG00000111375	Btbd8
ENSMUSG00000111403	Gm47544
ENSMUSG00000111409	Gm49380
ENSMUSG00000111447	Gm48249
ENSMUSG00000111471	Gm47350
ENSMUSG00000111497	Gm38431
ENSMUSG00000111654	Gm47077
ENSMUSG00000111692	Gm49373
ENSMUSG00000111713	Gm20234
ENSMUSG00000111789	Gm49323
ENSMUSG00000111795	Gm47620
ENSMUSG00000111842	Gm49318
ENSMUSG00000112006	Gm48633
ENSMUSG00000112109	Gm30906
ENSMUSG00000112129	Pbld1
ENSMUSG00000112189	C730027H18Rik
ENSMUSG00000112241	Gm49325
ENSMUSG00000112249	Gm30262
ENSMUSG00000112302	Gm48226
ENSMUSG00000112336	Gm47766
ENSMUSG00000112366	C730027H18Rik
ENSMUSG00000112397	Gm10824
ENSMUSG00000112426	Gm47200
ENSMUSG00000112429	Gm47093
ENSMUSG00000112437	Gm35188
ENSMUSG00000112510	Gm47566
ENSMUSG00000112515	Gm4928
ENSMUSG00000112550	Gm6627
ENSMUSG00000112599	Gm47719
ENSMUSG00000112709	Gm19395

ENSMUSG00000112758	Gm36172
ENSMUSG00000112774	Gm36041
ENSMUSG00000112796	Gm40770
ENSMUSG00000112873	Gm48877
ENSMUSG00000112909	Gm10120
ENSMUSG00000113030	Gm48778
ENSMUSG00000113061	Gm11361
ENSMUSG00000113063	Gm34667
ENSMUSG00000113149	Gm49383
ENSMUSG00000113475	Gm49366
ENSMUSG00000113512	Gm9063
ENSMUSG00000113521	Gm29787
ENSMUSG00000113543	Gm36264
ENSMUSG00000113786	Gm49384
ENSMUSG00000113949	Scamp4
ENSMUSG00000114046	Snapc1l
ENSMUSG00000114066	Gm9202
ENSMUSG00000114113	Gm47639
ENSMUSG00000114138	Gm36423
ENSMUSG00000114255	Gm10734
ENSMUSG00000114369	Gm41077
ENSMUSG00000114432	Gm49391
ENSMUSG00000114433	Gm48488
ENSMUSG00000114447	Gm47636
ENSMUSG00000114470	Gm49395
ENSMUSG00000114487	Gm49393
ENSMUSG00000114516	Gm46440
ENSMUSG00000114635	Gm49392
ENSMUSG00000114638	Gm31834
ENSMUSG00000114715	Gm34471
ENSMUSG00000114763	Gm49354
ENSMUSG00000114828	AI463229
ENSMUSG00000114922	Ppifos
ENSMUSG00000114934	Gm48342
ENSMUSG00000114942	Gm49361
ENSMUSG00000115007	5830448L01Rik
ENSMUSG00000115018	AL732309.1
ENSMUSG00000115022	Gm49387
ENSMUSG00000115049	Gm49172
ENSMUSG00000115074	Ndor1
ENSMUSG00000115154	Gm49067
ENSMUSG00000115219	Eef1akmt4
ENSMUSG00000115284	Gm34678
ENSMUSG00000115293	Gm49333

ENSMUSG00000115299	Gm49168
ENSMUSG00000115312	Gm8518
ENSMUSG00000115338	Pnp
ENSMUSG00000115388	Eppk1
ENSMUSG00000115423	AL731706.1
ENSMUSG00000115431	Gm3219
ENSMUSG00000115463	Gm49356
ENSMUSG00000115730	Gm7459
ENSMUSG00000115794	Gm49101
ENSMUSG00000115852	AC169509.1
ENSMUSG00000115867	Gm17753
ENSMUSG00000115919	Gm31583
ENSMUSG00000116016	Gm49496
ENSMUSG00000116024	Gm49527
ENSMUSG00000116075	Gm49476
ENSMUSG00000116121	Gm49486
ENSMUSG00000116130	Gm49431
ENSMUSG00000116138	C030006K11Rik
ENSMUSG00000116207	Nnt
ENSMUSG00000116227	Gm49513
ENSMUSG00000116262	Gm49544
ENSMUSG00000116275	Zc3h11a
ENSMUSG00000116347	Gm49416
ENSMUSG00000116348	Gm18722
ENSMUSG00000116354	Cyp2d38-ps
ENSMUSG00000116378	Gcat
ENSMUSG00000116508	Gm49463
ENSMUSG00000116564	Riok2
ENSMUSG00000116589	Gm31323
ENSMUSG00000116618	Gm49719
ENSMUSG00000116718	Gm49668
ENSMUSG00000116757	Gm4786
ENSMUSG00000116831	Gm30505
ENSMUSG00000116840	Gm21691
ENSMUSG00000116876	Gm49721
ENSMUSG00000116925	Gm49776
ENSMUSG00000116927	Gm30881
ENSMUSG00000116930	Gm49711
ENSMUSG00000116933	Atp5o
ENSMUSG00000116949	Gm7177
ENSMUSG00000116988	Gm49673
ENSMUSG00000116995	Gm21926
ENSMUSG00000117050	1700023B13Rik
ENSMUSG00000117284	Gm7072

ENSMUSG00000117310	Ptp4a1
ENSMUSG00000117322	6330415G19Rik
ENSMUSG00000117338	Gm49804
ENSMUSG00000117393	Gm36279
ENSMUSG00000117425	Gm7464
ENSMUSG00000117450	4933424G05Rik
ENSMUSG00000117458	Gm6552
ENSMUSG00000117465	Gm49980
ENSMUSG00000117485	Gm19696
ENSMUSG00000117621	Hspe1-rs1
ENSMUSG00000117694	Gm28285
ENSMUSG00000117732	Hdhd2
ENSMUSG00000117739	AC132452.1
ENSMUSG00000117748	Chtf8
ENSMUSG00000117757	AC140336.1
ENSMUSG00000117771	AC122861.1
ENSMUSG00000117780	AC127342.1
ENSMUSG00000117789	AC124502.3
ENSMUSG00000117819	Ggnbp1
ENSMUSG00000117839	AC109138.6
ENSMUSG00000117841	AC141471.2
ENSMUSG00000117862	AC115768.2
ENSMUSG00000117874	AC121821.1
ENSMUSG00000117876	AC121874.1
ENSMUSG00000117924	Tmem223
ENSMUSG00000117939	AC110212.1
ENSMUSG00000117942	AC115631.1
ENSMUSG00000117959	AC122861.4
ENSMUSG00000117975	Itprip
ENSMUSG00000117981	AC117612.2
ENSMUSG00000118007	AC144621.2
ENSMUSG00000118030	AC121821.2
ENSMUSG00000118052	AC148174.2
ENSMUSG00000118061	AC131065.2
ENSMUSG00000118077	AC134563.1
ENSMUSG00000118124	AC129217.1
ENSMUSG00000118125	Sssca1
ENSMUSG00000118135	AC134908.4
ENSMUSG00000118155	AC121973.1
ENSMUSG00000118168	AC102159.1
ENSMUSG00000118193	Arhgap26
ENSMUSG00000118251	AC120159.1
ENSMUSG00000118267	G630055G22Rik
ENSMUSG00000118332	Fam220a

ENSMUSG00000118341	AC174471.1
ENSMUSG00000118353	AC124513.1
ENSMUSG00000118361	AC132320.1
ENSMUSG00000118362	Gm19466
