# Supplementary material for: The methyltransferase METTL3 negatively regulates nonalcoholic steatohepatitis (NASH) progression
Source: Nat Commun. 2021 Dec 10;12:7213. doi: 10.1038/s41467-021-27539-3 (PMC8664922; doi:10.1038/s41467-021-27539-3)
Supplement: Supplementary file 2 — Reporting Summary [file 41467_2021_27539_MOESM2_ESM.pdf]

nature research

Corresponding author(s): Zheng Chen

Last updated by author(s): Nov 2, 2021

Reporting Summary

Nature Research wishes to improve the reproducibility of the work that we publish. This form provides structure for consistency and transparency in reporting. For further information on Nature Research policies, see our Editorial Policies and the Editorial Policy Checklist.

Statistics

For all statistical analyses, confirm that the following items are present in the figure legend, table legend, main text, or Methods section.

n/a	Confirmed	
☐	☒	The exact sample size (*n*) for each experimental group/condition, given as a discrete number and unit of measurement
☐	☒	A statement on whether measurements were taken from distinct samples or whether the same sample was measured repeatedly
☐	☒	The statistical test(s) used AND whether they are one- or two-sided<br>*Only common tests should be described solely by name; describe more complex techniques in the Methods section.*
☒	☐	A description of all covariates tested
☐	☒	A description of any assumptions or corrections, such as tests of normality and adjustment for multiple comparisons
☐	☒	A full description of the statistical parameters including central tendency (e.g. means) or other basic estimates (e.g. regression coefficient) AND variation (e.g. standard deviation) or associated estimates of uncertainty (e.g. confidence intervals)
☐	☒	For null hypothesis testing, the test statistic (e.g. *F*, *t*, *r*) with confidence intervals, effect sizes, degrees of freedom and *P* value noted<br>*Give P values as exact values whenever suitable.*
☒	☐	For Bayesian analysis, information on the choice of priors and Markov chain Monte Carlo settings
☒	☐	For hierarchical and complex designs, identification of the appropriate level for tests and full reporting of outcomes
☒	☐	Estimates of effect sizes (e.g. Cohen's *d*, Pearson's *r*), indicating how they were calculated

Our web collection on statistics for biologists contains articles on many of the points above.

Software and code

Policy information about availability of computer code

Data collection	RNA-seq, ATAC-seq and m6ARIP-seq analysis: Hisat2 version 2.0.4; HTSeq version 0.9.1; MACS2 Version 2.1.0; skewer Version: 0.1.126; 0.2.2, BWA Version 0.7.12; PeakAnnotator version 7.4; GOseq R package version 1.26.0; HOMER version 4.9.1; exomePeak R package version 2.16.0. qPCR: LightCycler 480 Software 1.5.1.
Data analysis	GraphPad Prism 6.02; ImageJ version 1.39f

For manuscripts utilizing custom algorithms or software that are central to the research but not yet described in published literature, software must be made available to editors and reviewers. We strongly encourage code deposition in a community repository (e.g. GitHub). See the Nature Research guidelines for submitting code & software for further information.

Data

Policy information about availability of data

All manuscripts must include a data availability statement. This statement should provide the following information, where applicable:
- Accession codes, unique identifiers, or web links for publicly available datasets
- A list of figures that have associated raw data
- A description of any restrictions on data availability

The ATAC-seq and RNA-seq data generated in this study have been deposited in the GEO database under accession code GSE141325 [https://www.ncbi.nlm.nih.gov/geo/query/acc.cgi?acc=GSE141325]. The m6ARIP-seq data generated in this study have been deposited in the GEO database under accession code GSE142835 [https://www.ncbi.nlm.nih.gov/geo/query/acc.cgi?acc=GSE142835]. The m6ARIP-seq data generated in this study are provided in the Supplementary Information/Source Data file. The published RNA-seq data from WT VS db/db mouse livers and NC VS NASH mouse livers used in this study are available in the GEO database under accession code GSE43314 and GSE119340 [https://www.ncbi.nlm.nih.gov/geo/query/acc.cgi?acc=GSE43314; https://www.ncbi.nlm.nih.gov/geo/query/acc.cgi?acc=GSE119340] .

The source data underlying Figs. 1a-i, 2a-q, 3a-d, 4a-d, 5b-g, 6a-f, 7b-l, 8a,d-h, and 9a-j and Supplementary Figs. 1a-n, 2a-h, 3b-f, 8a-b, 9, 10a-b, and 11 are provided as a Source Data file.

Field-specific reporting

Please select the one below that is the best fit for your research. If you are not sure, read the appropriate sections before making your selection.

- [x] Life sciences
- [ ] Behavioural & social sciences
- [ ] Ecological, evolutionary & environmental sciences

For a reference copy of the document with all sections, see nature.com/documents/nr-reporting-summary-flat.pdf

Life sciences study design

All studies must disclose on these points even when the disclosure is negative.

Sample size	The sample sizes for this study were chosen based on generally expected variations of metabolic parameters and typical sample sizes for metabolic studies documented in literature(References: PMID: 24995979; 22315325; 22581287; 27131369; 27871061; 32245957 and 30613274). Sample numbers were described in the Figure legends.
Data exclusions	No data were excluded from the analyses.
Replication	All the cell culture experiments were repeated, at least, three times and reproduced. More than three independent mice were used for animal experiments. ATAC-sequencing, RNA-sequencing and m6ARIP-seqencing were performed once from pooled tissue or RNA samples, but three to ten independent samples were further validated by alternative approaches, such as ChIP-qPCR and qRT-PCR. Western blotting data were confirmed by three to four independent samples.
Randomization	Mice with different genotypes were randomly divided into different experimental groups. Cells were grown under the same conditions and randomly allocated into different treatment groups without any bias.
Blinding	The H&E staining experiments were performed by the authors who were blinded to the experimental groups. ATAC-sequencing, RNA sequencing, m6ARIP-sequencing, library constructions, and RNA sequencing alignments were performed by technical staffs at Novogene who were blinded to the experimental groups. Blinding was not relevant to the other experiments in mice or cells because mice or cells had to be genotyped by PCR.

Reporting for specific materials, systems and methods

We require information from authors about some types of materials, experimental systems and methods used in many studies. Here, indicate whether each material, system or method listed is relevant to your study. If you are not sure if a list item applies to your research, read the appropriate section before selecting a response.

Materials & experimental systems

n/a	Involved in the study	
[ ]	[x]	Antibodies
[ ]	[x]	Eukaryotic cell lines
[x]	[ ]	Palaeontology and archaeology
[ ]	[x]	Animals and other organisms
[ ]	[x]	Human research participants
[x]	[ ]	Clinical data
[x]	[ ]	Dual use research of concern

Methods

n/a	Involved in the study	
[x]	[ ]	ChIP-seq
[x]	[ ]	Flow cytometry
[x]	[ ]	MRI-based neuroimaging

Antibodies

Antibodies used	Antibodies: METTL3 Cell Signaling Technology 96391 D2160; Flag Sigma F1804; Brdu Proteintech 66241-1-Ig 1B10E12; CD36 Proteintech 18836-1-AP; CCL2 Proteintech 1B9F7; β-actin Proteintech 60008-1-Ig 7D2C10; CDK9 Proteintech 11705-1-AP; Lamin B1 Proteintech 12987-1-AP; pCDK9 Cell Signaling Technology 2549; Phosphoserine Sigma AB1603; Caspase3 Cell Signaling Technology 9662; Myc Proteintech 16286-1-AP; HDAC1 Cell Signaling Technology 5356 10E2; HDAC2 Cell Signaling Technology 5113 3F3; Acetyl-Histone H3 (Lys9) Jingjie PTM BioLab PTM-112; Acetyl-Histone H3 (Lys27) Cell Signaling Technology 4353; Tubulin Santa cruz sc-5286 B-7; HDAC1 Proteintech 10197-1-AP; HDAC2 Proteintech 12922-3-AP; Rat IgG2b Proteintech 65211-1-Ig; Mouse IgA Bioss bs-0774P; CD36 Abcam ab23680 JC63.1; CCL2 R&D Systems MAB479500 Clone # 123616; Normal rabbit IgG Cell Signaling Technology 2729.
Validation	All commercial antibodies have been verified and used in multiple previous publications as shown below.<br>METTL3 CST Cat#96391 D2I6O https://www.cst-c.com.cn/products/primary-antibodies/mettl3-d2i6o-rabbit-mab/96391<br>Species specificity: Human, mouse, rat, monkey<br>Applications: WB, IP<br>Publications: PMID:31167133, 30212448, 32245957

Brdu Proteintech Cat#66241-1-Ig 1B10E12 https://www.ptgcn.com/products/BrDu-Antibody-66241-1-Ig.htm
Species specificity: Human, mouse, rat
Applications: IF, IHC,ELISA
Publications: PMID: 26476374,27884164,27540765

CD36 Proteintech Cat#18836-1-AP https://www.ptgcn.com/products/CD36-Antibody-18836-1-AP.htm
Species specificity: Human, mouse, hamster
Applications: FC, IHC, WB,ELISA
Publications: PMID: 30233583,29664631,29025707

CCL2 Proteintech Cat#66272-1-Ig 1B9F7 https://www.ptgcn.com/products/Mcp1-Antibody-66272-1-Ig.htm
Species specificity: Mouse
Applications:IHC, WB,ELISA
Publications: PMID: 26978584,28943249,28129426

β-actin Proteintech Cat#60008-1-Ig 7D2C10 https://www.ptgcn.com/products/ACTB-Antibody-60008-1-Ig.htm
Species specificity: Human, mouse, rat, pig, plant, Zebrafish
Applications: FC, IF, IHC, WB,ELISA
Publications: PMID: 18667751,19699238,18596218

CDK9 Proteintech Cat#11705-1-AP https://www.ptgcn.com/products/CDK9-Antibody-11705-1-AP.htm
Species specificity: Human
Applications: IHC, WB
Publications: PMID: 27315790,28474697

Lamin B1 Proteintech Cat#12987-1-AP https://www.ptgcn.com/products/LMNB1-Antibody-12987-1-AP.htm
Species specificity: Human, mouse, rat
Applications: ChIP, IF, IHC, WB
Publications: PMID: 20132211,22745163,23146752

pCDK9 CST Cat#2549 https://www.cellsignal.cn/products/primary-antibodies/phospho-cdk9-thr186-antibody/2549?site-search-type=Products&N=4294956287&Ntt=2549&fromPage=plp&_requestid=556627
Species specificity: Human, mouse, rat, monkey
Applications: WB
Publications: PMID: 31848275,31101827,29792310

Phosphoserine Sigma Cat#AB1603 https://www.sigmaaldrich.cn/CN/zh/product/mm/ab1603?context=product
Species specificity: All
Applications: ELISA, WB, IP, IHC
Publications: PMID: 11181844,12960006,15514089

Flag Sigma Cat#F1804 m2 https://www.sigmaaldrich.cn/CN/zh/product/sigma/f1804?context=product
Species specificity: All
Applications: WB, IP, IHC, IF, ICC
Publications: PMID: 28053121,28328949,27172195

Caspase3 CST Cat#9662 https://www.cellsignal.cn/products/primary-antibodies/caspase-3-antibody/9662?site-search-type=Products&N=4294956287&Ntt=9662&fromPage=plp
Species specificity: Human, mouse, rat, monkey
Applications: WB, IP, IHC
Publications: PMID: 31971852,32195376,32235878

Myc Proteintech Cat#16286-1-AP https://www.ptgcn.com/products/MYC-tag-Antibody-16286-1-AP.htm
Species specificity: Human, pig
Applications:IF, IP, WB,ELISA
Publications: PMID: 25906440,28069035,28598244

HDAC1 CST Cat#5356 10E2 https://www.cellsignal.cn/products/primary-antibodies/hdac1-10e2-mouse-mab/5356?site-search-type=Products&N=4294956287&Ntt=5356&fromPage=plp&_requestid=558238
Species specificity: Human, mouse, rat, monkey
Applications: WB, IP
Publications: PMID: 31924750,31885815,31849603

HDAC2 CST Cat#5113 3F3 https://www.cellsignal.cn/products/primary-antibodies/hdac2-3f3-mouse-mab/5113?site-search-type=Products&N=4294956287&Ntt=5113&fromPage=plp&_requestid=558365
Species specificity: Human, mouse, rat, monkey
Applications: WB, IP, IF
Publications: PMID: 31799598,31924750,31849603

Acetyl-Histone H3 (Lys9) Jingjie PTM BioLab Cat#PTM-112 http://www.ptm-biolab.com.cn/productDetail.html?id=5656
Species specificity: Human, mouse, rat
Applications: WB, CHIP
Publications: PMID: 28429772, 25160476, 23909948

Acetyl-Histone H3 (Lys27) CST Cat#4353 https://www.cellsignal.cn/products/primary-antibodies/acetyl-histone-h3-lys27-antibody/4353?site-search-type=Products&N=4294956287&Ntt=4353&fromPage=plp&_requestid=558793
Species specificity: Human, mouse, rat, monkey
Applications: WB, IP, CHIP
Publications:PMID: 33397936, 32807777, 32025238

Tubulin Santa cruz Cat#sc-5286 B-7 https://www.scbt.com/zh/p/alpha-tubulin-antibody-b-7;jsessionid=Wa7VUc80f-fcWsAzlkec3W5PWv8SzKuYFJhw7AMBzcxfy9hKbmKf!-98738317
Species specificity: Human, mouse, rat, canine, bovine and porcine
Applications: WB, IP, IF, IHC, FCM, ELISA
Publications: PMID: 30755469; 31499479; 30810354

HDAC1 Proteintech 10197-1-AP https://www.ptgcn.com/products/HDAC1-Antibody-10197-1-AP.htm
Species specificity: Human, mouse, rat
Applications: FC, IF, IHC, IP, WB, ELISA
Publications: PMID: 31040183; 32409664; 32951004

HDAC2 Proteintech 12922-3-AP https://www.ptgcn.com/products/HDAC2-Antibody-12922-3-AP.htm
Species specificity: Human, mouse, rat
Applications: FC, IF, IHC, IP, WB, ELISA
Publications: PMID: 31657882; 25486475; 29988076

Rat IgG2b Isotype Control Proteintech Cat#65211-1-Ig LTF-2 https://www.ptgcn.com/products/IgG2b-Isotype-Control-Antibody-65211-1-Ig.htm
Species specificity:
Applications: FC, Isotype Control

Mouse IgA Bioss Cat#bs-0774P http://www.bioss.com.cn/prolook_03.asp?id=AF08169606013892&pro37=5
Species specificity:
Applications: Isotype Control

CD36 Abcam Cat#ab23680 JC63.1 https://www.abcam.cn/cd36-antibody-jc631-ab23680.html
Species specificity: Mouse
• Applications: FC, Neutralization(30728288)
Publications: PMID: 32850342, 33940547, 32198214, 30728288

CCL2 R&D Systems Cat#MAB479500 Clone # 123616 https://www.rndsystems.com/cn/products/mouse-ccl2-je-mcp-1-antibody-123616_mab479
Species specificity: Mouse
Applications: Neutralization
Publications: PMID: 27158906, 22851704, 33238115

Normal rabbit IgG CST Cat#2729 https://www.cellsignal.cn/products/primary-antibodies/normal-rabbit-igg/2729?site-search-type=Products&N=4294956287&Ntt=2729&fromPage=plp&_requestid=588923
Species specificity:
Applications: Isotype Control
Publications: PMID: 34172044, 34168130, 34158624

Eukaryotic cell lines

Policy information about cell lines

Cell line source(s)	HEK293T ATCC CRL3216
Authentication	The cell line has not been authenticated recently.
Mycoplasma contamination	The cell line was tested negative for mycoplasma contamination.
Commonly misidentified lines (See ICLAC register)	There is no ICLAC line used in this study.

Animals and other organisms

Policy information about studies involving animals; ARRIVE guidelines recommended for reporting animal research

Laboratory animals	Male mice studied ranged between 8 to 20 weeks of age. All mice were in C57BL/6 background. Animals were kept under controlled light (12hour light and 12hour dark cycle), temperature (24 ± 2°C) and humidity (50% ± 10%) conditions. The age, strain and housing temperature of mice were described in 'Methods' and relevant 'Figure Legends' of the manuscript.
Wild animals	This study did not involve wild animals.
Field-collected samples	This study did not involve samples collected from the field.
Ethics oversight	Animal experiments were carried out in strict accordance with the Guide for the Care and Use of Laboratory Animals published by the US National Institutes of Health (NIH publication No. 85-23, revised 1996) and approved by the Institutional Animal Care and Use Committee or Animal Experimental Ethics Committee of Harbin Institute of Technology (HIT/IACUC). The permit number was IACUC-2018004.

Note that full information on the approval of the study protocol must also be provided in the manuscript.

Human research participants

Policy information about studies involving human research participants

Population characteristics

The clinical information and histologic features of subjects included in this study was shown in Supplementary Table 1.
The information was also shown below.

	Non-steatosis	NASH
All	9	10
Male Gender	3(33.3%)	8(80%)
Age (years)	41.78±4.36	39.6±3.63
BMI(kg/m2)	21.83±0.85	23.03±0.4
ALT(U/L)	20±4.49	131.2±43.24
AST(U/L)	20±1.55	65.3±17.57
Cholesterol (mM)	3.95±0.25	4.97±0.5
Triglycerides (mM)	2.09±0.45	2.07±0.46
HDL(mM)	1.65±0.27	1.38±0.2
LDL(mM)	2.22±0.31	2.96±0.47
FBG(mM)	4.47±0.15	7.51±1.49
Steatosis grade (1/2/3)	0	3/4/3
Lobular inflammation (1/2/3)	0	3/5/2
Ballooning (0/1/2)	0	6/2/2
Fibrosis (0/1)	0	3/7

Recruitment	All the participants in this study were enrolled between March 2018-March 2020 at the 3rd affiliated hospital of Sun Yat-sen university. No expect biases, including self-selection bias was expected from patient samples. The investigation conforms to the principles that are outlined in the Declaration of Helsinki regarding the use of human tissues.
Ethics oversight	The present study were approved by the Research Ethics Committee of the Third Affiliated Hospital of Sun Yat-sen University

Note that full information on the approval of the study protocol must also be provided in the manuscript.
